# Supplementary material for: Efficient palladium-catalyzed electrocarboxylation enables late-stage carbon isotope labelling
Source: Nat Commun. 2024 Mar 22;15:2592. doi: 10.1038/s41467-024-46820-9 (PMC10959938; doi:10.1038/s41467-024-46820-9)
Supplement: Supplementary file 1 — Supplementary Information [file 41467_2024_46820_MOESM1_ESM.pdf]

Supplementary Information

Palladium-catalysed electrocarboxylation enables late-stage radiolabelling with near-stoichiometric $^{14}CO_2$

Gabriel M. F. Batista[1], Ruth Ebenbauer[1], Craig Day[1], Jonas Bergare[2], Karoline T. Neumann[1], Kathrin H. Hopmann[3], Charles S. Elmore[2], Alonso Rosas-Hernández[1*], and Troels Skrydstrup[1*]

[1]Carbon Dioxide Activation Center (CADIAC), Interdisciplinary Nanoscience Center, Department of Chemistry, Aarhus University, Gustav Wieds Vej 14, 8000 Aarhus C, Denmark. [2]Early Chemical Development, Pharmaceutical Sciences R&D AstraZeneca, 43150 Gothenburg, Sweden. [3]Department of Chemistry, UiT - The Arctic University of Norway, N-9037 Tromsø, Norway.

*Corresponding author. Email: ts@chem.au.dk, arosas@chem.au.dk

Table of Contents

1. GENERAL INFORMATION 3

1.1 GENERAL METHODS: 3

1.2 HANDLING OF SULFURYL FLUORIDE: 3

2 OPTIMIZATION OF REACTION CONDITIONS 4

2.1 GENERAL PROCEDURE FOR THE LIGAND OPTIMIZATION UNDER PHOTOREDOX CONDITIONS AND OPTIMIZATION DISCUSSION 4

2.2 GENERAL PROCEDURE FOR THE OPTIMIZATION OF THE ELECTROCARBOXYLATION AND OPTIMIZATION DISCUSSION 5

2.3 GENERAL PROCEDURE FOR THE OPTIMIZATION OF THE CARBON ISOTOPE LABELLING: 9

3 ATTEMPTS WITH PUBLISHED CATALYTIC REDUCTIVE CARBOXYLATION METHODS: 12

3.1 PROCEDURE FOR THE TRIAL WITH MARTIN[4] CONDITIONS WITH NEAR-STOICHIOMETRIC CO_2: 12

3.2 PROCEDURE FOR THE TRIAL WITH TSUJI[5] CONDITIONS WITH NEAR-STOICHIOMETRIC CO_2: 13

3.3 PROCEDURE FOR THE TRIAL WITH MEI[6] CONDITIONS WITH NEAR-STOICHIOMETRIC CO_2: 13

3.4 PROCEDURE FOR THE TRIAL WITH IWASAWA[7,8] CONDITIONS WITH NEAR-STOICHIOMETRIC CO_2: 14

3.5 PROCEDURE FOR THE TRIAL WITH JANA[9] CONDITIONS WITH NEAR-STOICHIOMETRIC CO_2: 14

3.6 PROCEDURE FOR THE TRIAL WITH KÖNIG[10] CONDITIONS WITH NEAR-STOICHIOMETRIC CO_2: 15

3.7 PROCEDURE FOR THE TRIAL WITH YU[11] CONDITIONS WITH NEAR-STOICHIOMETRIC CO_2: 15

3.8 PROCEDURE FOR THE TRIAL WITH WANG[12] CONDITIONS WITH NEAR-STOICHIOMETRIC CO_2: 16

3.9 PROCEDURE FOR THE TRIAL WITH ACKERMANN[13] CONDITIONS WITH NEAR-STOICHIOMETRIC CO_2: 17

4 MECHANISTIC INVESTIGATION (EXPERIMENTAL AND DFT) 18

4.1 COMPUTATIONAL DETAILS: 18

4.1.1 DFT data: 19

4.1.2 Imaginary frequencies for the transition states: 21
4.1.3 Computed energies: 21
4.1.4 Intrinsic reaction coordinate (IRC): 24
4.2 General Procedure for cyclic voltammetry data: 28
4.2.1 Cyclic voltammetry data analysis: 28
4.3 Electron donor study: 36
4.4 Organometallic Synthesis 36
4.4.1 Synthesis of (BINAP)Pd(o-tolyl)(Br) complex 32 36
4.4.2 Synthesis of (BINAP)Pd(phenyl)(Br) 37
4.5 Mechanistic Experiments 38
4.5.1 Reaction of (BINAP)Pd(o-tolyl)(Br) with CO_2 38
4.5.2 Cyclic voltammetry of complex 32: 39
4.5.3 Monitoring the catalytic reaction under constant potential: 40
4.5.4 Changing reaction parameters of standard catalytic reaction under constant potential: 40
4.6 Further screening of reaction conditions after mechanistic study 41
4.6.1 General Procedure for the optimization of the amide-free solvent conditions: 42
5 SYNTHESIS OF STARTING MATERIALS 43
5.1 Fluorosulfation reactions: 43
5.1.1 General Procedure I 44
5.1.2 General Procedure II 44
5.1.3 General Procedure III 44
5.2 Synthesis of aryl bromides 55
6 SYNTHESIS OF ARYL CARBOXYLIC ACIDS 57
6.1 General Procedure for the electrocarboxylation of aryl bromides and aryl fluorosulfates with $^{12}CO_2$ (1 atm) and ascorbic acid as electron donor (General procedure A) 57
6.2 General Procedure for the electrocarboxylation of aryl bromides and aryl fluorosulfates with $^{12}CO_2$ (1 atm) and ethanol as electron donor (General procedure B). 57
6.3 General Procedure for the electrocarboxylation of aryl bromides and aryl fluorosulfates with CO_2 (1.5 equiv.) (General procedure C). 58
6.4 General Procedure for the electrocarboxylation of aryl bromides and aryl fluorosulfates with CO_2 (1 atm) and acetonitrile as solvent (General procedure D). 59
6.5 Faradaic efficiency: 61
6.6 Synthesized carboxylic acids: 63
7 NMR SPECTRA 89
8 REFERENCES 154

1. General Information

1.1 General Methods:

The glovebox is under an argon atmosphere. All commercial chemicals were purchased from Sigma-Aldrich, Tokyo Chemical Industry (TCI) or Strem Chemicals and used as received without further purification, unless otherwise noted. Barium carbonate (13C) was purchased from Cambridge isotope laboratories while barium carbonate (14C) was purchased from moravek. Flash column chromatography was carried out on silica gel 60 (230-400 mesh) with the described eluents. The chemical shifts in NMR data are reported in ppm relative to solvent residual peak (for ^{1}H and ^{13}C NMR), and the coupling patterns in the NMR spectra are abbreviated as follows: s = singlet, d = doublet, t = triplet, q = quartet, quint = quintuplet, sext = sextet, sep = septet, m = multiplet, dd = double doublet, dt = double triplet, ddd = double-double doublet. The NMR for each synthesized compound is reported as follows: (multiplicity; coupling constant(s) in Hz; integration). HRMS spectra were recorded on an LC TOF (ES) apparatus. Cyclic voltammetry measurements and constant potential reactions were recorded on a CH Instruments Electrochemical Analyzer Model CHI600E. For the constant potential reactions, the same potentiostat was used, and for the constant current reactions the in-house made Electroware was used.[1] The reference electrode (LF-1.6-100, Leak-Free Reference Electrode. 1.6 mm OD, 100 mm barrel from Innovative Instruments) was used without any modifications. The carbon paper electrodes (Toray 090 carbon paper wet-proofed) were cut to the necessary size with a sharp knife. The electrode holders, metallic electrodes, and glassware were made in-house, photos and further specifications are detailed below.

1.2 Handling of Sulfuryl Fluoride:

All reactions with Sulfuryl Fluoride were performed in a two-chamber glassware, in which gaseous SO_2F_2 was released (from 1,1'-sulfonylbis(1*H*-imidazole) (SDI) and KF in TFA at room temperature) in one of the chambers and utilized in the other.[2] The two-chamber glassware (COWare®) is composed of two thick wall glass vials connected by a hollow glass tube to allow gas-transfer. The chambers are sealed with a screw cap and a Teflon® coated silicone seal. Precise conditions and amounts are further described in the general procedures for aryl fluorosulfates synthesis.
WARNING: Glassware under pressure!

- Glassware should always be examined for damages or scratches.
- One must abide to all laboratory safety procedures and always work behind a safety shield when working with pressurized glassware.
- COware should under no circumstances be operated above 60 psi (5 bar).

2 Optimization of reaction conditions

2.1 General Procedure for the ligand optimization under photoredox conditions and optimization discussion

Inside the glovebox, a pressure tube glassware (Supplementary Figure 1) was charged with base, additives, photocatalyst, palladium precursor, and ligand. To that, 0.5 mL of solvent was added, followed by the addition of the sacrificial reductant (by volume with a micropipette) and the substrate (by weighting directly inside the glassware), and another 0.5 mL of solvent was added, rinsing the walls of the glassware. The glassware was closed and taken outside the glovebox, where it was cooled down with ice and connected to a vacuum line (Supplementary Figure 1) that is connected to a CO_2 cylinder. The atmosphere was changed 3 times with the application of vacuum until the solution started bubbling and leaving under a CO_2 atmosphere for 1 minute. Then it was displaced at the top of a stirring plate at a 5 cm distance to a Kessil LED A160WE Tuna Blue 40 W and allowed to stir overnight. Once the reaction was done it is quenched with 1M HCl and extracted 4 times with EtOAc, the combined organic phases were dried with magnesium sulfate and concentrated under reduced pressure. To this 1,3,5-trimethoxybenzene was added and ^{1}H crude NMR was recorded.

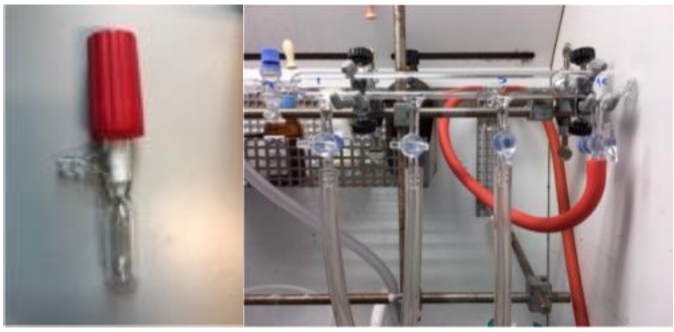

Supplementary Figure 1 Glassware used for the photoredox reactions.

Initially, a ligand screening was performed under photoredox conditions, (Supplementary Figure 2). In these experiments, BINAP, along with tBuXPhos and BrettPhos gave the best results, with BINAP presenting the highest yield and selectivity towards the desired product. Since BINAP is a less electron-rich phosphine compared to the other two ligands, the oxidative addition complex should have a lower reduction potential, thus allowing it to be applied for electron-poor substrates; the described advantages of BINAP in comparison to tBuXPhos and BrettPhos led us to continue with BINAP as the ligand of choice. Under photoredox conditions, Iwasawa has shown that a higher proton concentration in the medium leads to greater amounts of the side product **S1**. This can be partially avoided with the use of bases, but even with 3 equivalents of cesium carbonate, 37% of product **S1** can still be observed. In an electrochemical setup, it is possible to separate the reduction and oxidation reactions and thus control the proton concentration

near the palladium catalyst, which led us to transpose the BINAP-palladium catalytic system to an electrochemical setup.

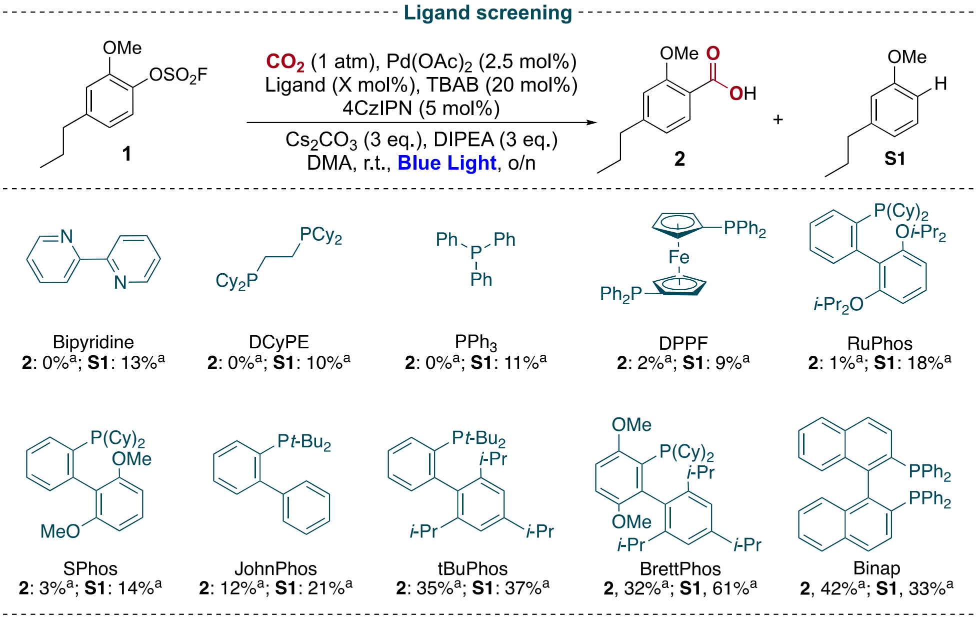

Supplementary Figure 2 Ligand screening.

[a] NMR yield calculated with 1,3,5-trimethoxybenzene as internal standard

2.2 General Procedure for the optimization of the electrocarboxylation and optimization discussion

Outside the glovebox the electrodes were prepared, a 2x1 cm piece of carbon paper (Toray paper 090 waterproofed) was attached to an aluminum holder (Supplementary Figure 1). In order to define the available area, the electrode was covered using Teflon® tape to leave an area of 2 cm^2 available (1x1 cm surface area with both sides uncovered), this was pierced through a Teflon® inlay and taken inside the glovebox together with the oven dried (120 °C) glassware. Inside the glovebox, the cathodic chamber was charged with palladium precursor, electrolyte, and additive. The anodic chamber was charged with electrolyte, additive, and sacrificial reductant. Solvent was added first to the anodic chamber followed by the cathodic chamber (2 mL of DMF to the anodic chamber followed by 2 mL in the cathodic chamber), then 2-methoxy-4-propylphenyl sulfurofluoridate (99.3 mg, 0.4 mmol) was weighted directly inside the cathodic chamber and the exact mass noted down. After the extra 1mL of solvent left is used to rinse the walls of the cathodic chamber and the solvent is leveled at the anodic side (total of 3 mL of DMF for each chamber). The electrodes were rinsed with dry DMF before being introduced into the solution, afterwards the reaction flask was sealed, and taken outside the glovebox. Once outside the glovebox the reaction is allowed to stir until everything is soluble. Afterwards, the atmosphere of the reaction was exchanged with 3 cycles of vacuum followed by leaving under 1 atm of CO_2 (1 minute each time) using a needle attached to a vacuum line. This was followed by attaching the electrodes to a potentiostat

(reactions with constant potential) or the Electroware (reactions with constant current), and 2.2 mol of electrons were allowed to pass (or until the current passing was below 5% of the initial current) (Supplementary Figure 3). Once the reaction was done it is quenched with 1M HCl and extracted 4 times with EtOAc, the combined organic phases were dried with magnesium sulfate and concentrated under reduced pressure. To this 1,3,5-trimethoxybenzene was added and ^{1}H crude NMR was recorded.

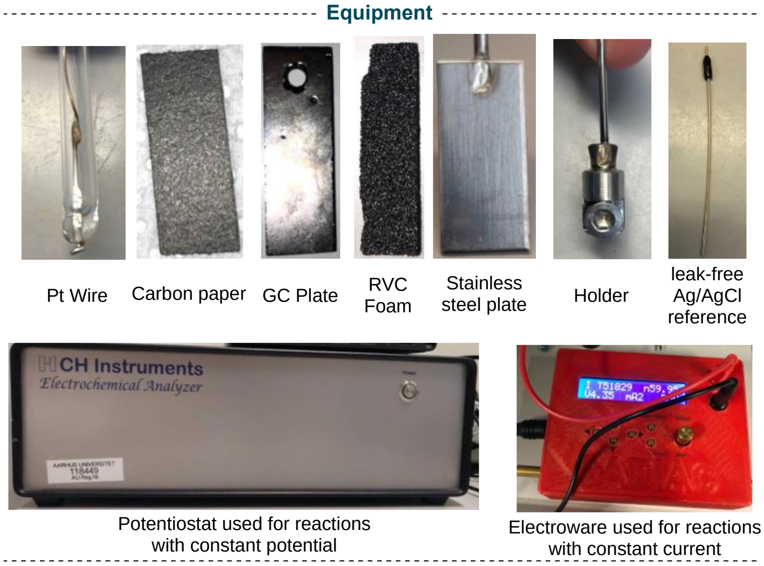

Supplementary Figure 3 Pictures of the used equipment for the reaction screening

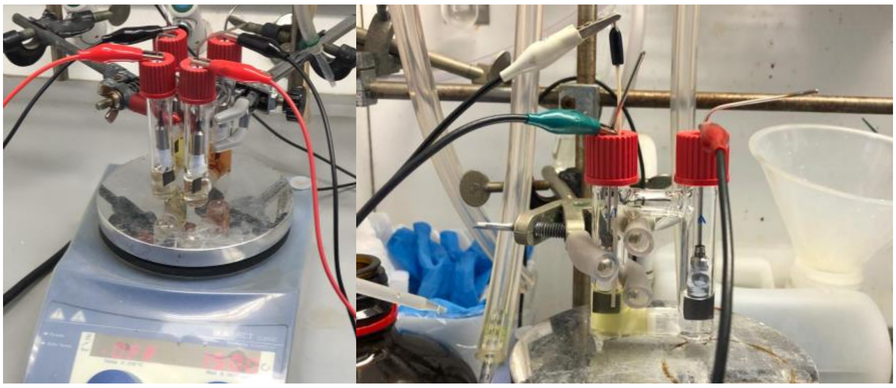

Supplementary Figure 4 Set up used for screening the reaction conditions

Using the result from the ligand optimization that BINAP is the best ligand for the carboxylation of substrate 1, we attempted the feasibility of transposing this catalytic system to an electrochemical setup (Supplementary Figure 5). This could have a major advantage compared to the photoredox setup due to the possibility of separating the reaction chambers, and thus decreasing the proton concentration in the carboxylation reaction. This was attempted using BINAPPdG4 as the pre-catalyst where we could get a proof of concept that using an electrochemical setup with a constant potential of -1.8 (vs Ag/AgCl) was feasible. The addition of lithium bromide was attempted to see if

lithium could be used as a Lewis acid to aid in the CO_2 activation, this increased the yield to 78% and up to >95% using more reductive conditions. Attempts to use a smaller concentration led to a decreased yield of 37%. With these initial conditions, the catalyst loading could not be reduced to 1 mol%, and thus the reaction was attempted with either lithium tetrafluoroborate or tetrabutylammonium bromide to observe what was the separate effect of the lithium bromide additive. With this, it was possible to observe that lithium has a negative effect on the reaction while the presence of bromides is beneficial. With this in mind the use of the commercial pre-ligated catalyst (BINAP)$PdCl_2$ was attempted, and demonstrated to be optimal.

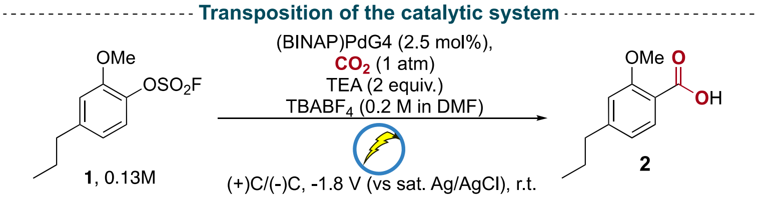

Entry	Change	Yield[a] **2** (%)
1	none	48
2	Addition of LiBr (1.1 equiv)	78
3	As entry 2 and -2.0 V (vs sat. Ag/AgCl)	>95
4	As entry 3 but [1] = 0.1 M	38
5	(BINAP)PdG4 (1.0 mol%)	20
6	$LiBF_4$ (1.1 equiv.) i.o. LiBr	18
7	no LiBr, TBABr (0.2 M in DMF) electrolyte in cathodic chamber	>95
8	(BINAP)$PdCl_2$ (2.5 mol%) as pre catalyst, no LiBr	>95

Supplementary Figure 5 Transposition of the photoredox-mediated catalytic system to an electrochemical setup

[a] NMR yield calculated with 1,3,5-trimethoxybenzene as internal standard

Although good yields could already be obtained there were still a few parameters we wanted to optimize to ensure a more environmentally friendly reaction condition. First the replacement of triethylamine for ascorbic acid was shown to lead to comparable yields. Furthermore, due to the simplicity and low cost of the electroware galvanostat compared to a potentiostat we wanted to ensure that the reaction could be run using constant current. The average current for entry 2 Sup. Fig. 6 (-4 mA) was used and provided the carboxylic acid in the same yield. With this result in mind, we once again attempted to decrease the catalyst loading, now it was possible to achieve comparable yields with only 1 mol%. Further lowering the catalyst loading was not successful, even when trying to increase the reaction concentration, the use of 0.5 mol% of catalyst led to 30% lower yield.

Catalyst loading and change to Ascorbic acid

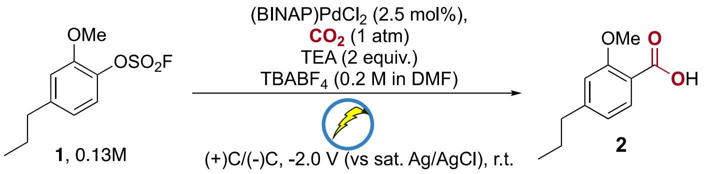

Entry	Change	Yield[a] **2** (%)
1	none	>95
2	Ascorbic Acid (2 equiv.) i.o. TEA	>95
3	A.A. (2 equiv.), -4 mA i.o. -2.0 V	>95
4	A.A. (2 equiv.), (BINAP)$PdCl_2$ (1.0 mol%) and -4 mA	93
5	A.A. (2 equiv.), (BINAP)$PdCl_2$ (0.5 mol%) and -4 mA	65
6	A.A. (2 equiv.), (BINAP)$PdCl_2$ (0.1 mol%) and -4 mA	n.r.
7	A.A. (2 equiv.), (BINAP)$PdCl_2$ (0.5 mol%) and -4 mA, [**S1**]=0.2 M	66
8	A.A. (2 equiv.), (BINAP)$PdCl_2$ (0.5 mol%) and -4 mA, [**S1**]=0.3 M	52

Supplementary Figure 6 Screening of catalyst loading and change from triethylamine (TEA) to ascorbic acid (A.A.)

[a] NMR yield calculated with 1,3,5-trimethoxybenzene as internal standard

The solvent for the reaction was also optimized, but the use of DMF could not be avoided, although an initial 55% yield was obtained with acetonitrile, it is still poor compared to 93% obtained in DMF (Supplementary Figure 6). The exchange of tetrabutylammonium tetrafluoroborate for other electrolytes was attempted but led to lower yields (Sup. Fig. 7).

Solvent and electrolyte

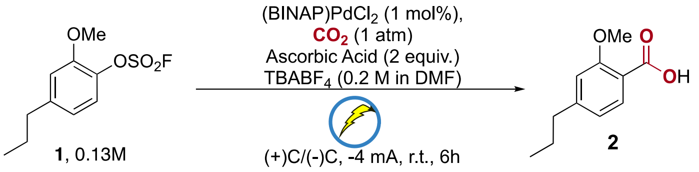

Entry	Change	Yield[a] **2** (%)
1	none	93
2	2-MeTHF i.o. DMF	n.r.
3	ACN i.o. DMF	55
4	NaI (0.2 M in DMF) i.o. $TBABF_4$	n.r.
5	KI (0.2 M in DMF) i.o. $TBABF_4$	n.r.
6	$NaBF_4$ (0.2 M in DMF) i.o. $TBABF_4$	n.r.
7	TMABF4 (0.2 M in DMF) i.o. $TBABF_4$	54
8	TEACl (0.2 M in DMF) i.o. $TBABF_4$	67

Supplementary Figure 7 Screening of solvent and electrolyte

[a] NMR yield calculated with 1,3,5-trimethoxybenzene as internal standard

Although the use of ascorbic acid represents a renewable feedstock in comparison to triethylamine, we decided to further investigate if other additives can be used in the anodic chamber. In the described electrochemical setup, the potential in the counter electrode will increase until a compound in the anodic chamber is oxidized. Thus, either an additive or the solvent must be oxidized to provide the electrons for the reaction of interest in the cathodic chamber. It is of utmost importance that the oxidation rate does not limit the potential at the working electrode during the constant-current electrolyzes in the two-electrode set-up. If the necessary potential to drive the oxidation reaction at the required current is too high, the potential at the working electrode might not be negative enough to reduce the key palladium intermediary with appreciable rates, leading to low yields and undesired side products. The absence of an additive led to lower reaction yields (37%), demonstrating that the direct oxidation of the solvent under the reaction conditions requires a higher oxidation potential, whereas the addition of ethanol was shown to facilitate this process, obtaining yields of 87%.

Reductant screening

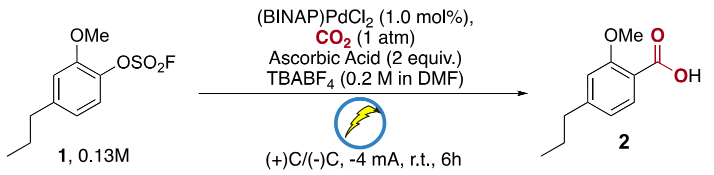

Entry	Change	Yield[a] **2** (%)
1	none	93
2	Dry ethanol (3 equiv.)	63
3	Dry ethanol (4 equiv.)	92
4	Dry ethanol (5 equiv.)	91
5	Dry ethanol (11 equiv.)	93
6	96% ethanol (4 equiv.)	87
7	96% ethanol (4 equiv.) and outside the GB	87
8	Glucose (2 equiv.) as electron donor	0
9	No reductant in the anodic chamber	37

Supplementary Figure 8 Electron donor optimization

[a] NMR yield calculated with 1,3,5-trimethoxybenzene as internal standard

2.3 General Procedure for the optimization of the Carbon isotope labelling:

Outside the glovebox, the electrodes were prepared: a 2x1 cm piece of carbon paper (Toray carbon paper 090 wet-proofed) was attached to an aluminum holder (Supplementary Figure 1). In order to define the available area the electrode was covered using Teflon® tape to leave an area of 2 cm^2 available (1x1 cm surface area with both sides uncovered), this was pierced through a Teflon® inlay and taken inside the glovebox together with the oven dried (120 °C) glassware. Inside the glovebox, the gas release chamber was charged with $BaCO_3$ (118 mg, 0.6 mmol, 1.5 equiv.) and camphorsulfonic acid (279 mg, 1.2 mmol, 3 equiv.).[3] The cathodic chamber was charged with (BINAP)$PdCl_2$ (3.2 mg, 1 mol%), and $TBABF_4$ (198 mg). The anodic chamber was charged with ascorbic acid (141mg, 2 equivalents) and $TBABF_4$ (198 mg). Solvent was

added first to the anodic chamber followed by the cathodic chamber (2 mL of DMF to the anodic chamber followed by 2 mL in the cathodic chamber), then 2-methoxy-4-propylphenyl sulfurofluoridate (99.3 mg, 0.4 mmol, 1 equiv.) was weighted directly inside the cathodic chamber and the exact mass noted down. After the extra 1 mL of solvent left is used to rinse the walls of the cathodic chamber and the solvent is leveled at the anodic side (total of 3 mL of DMF for each chamber). The electrodes were rinsed with dry DMF before introducing them into the solution, and closure of the reaction flask. After the anodic and cathodic chamber were closed, organic solvent was added to the third chamber followed by layering with water to form a biphasic mixture that was then quickly closed (to avoid losing CO_2 pressure). Once outside the glovebox, the reaction is allowed to stir for 5 minutes for the gas release to occur, after which a constant current (-4 mA) is applied for 2.2 mols of electrons (85 Coulombs). Once the reaction is completed it is quenched with 1 M HCl and extracted 4 times with EtOAc, the combined organic phases were dried with magnesium sulfate and concentrated under reduced pressure. To this 1,3,5-trimethoxybenzene was added and crude ^{1}H NMR spectrum was recorded. For the reaction setup outside the glovebox, the atmosphere inside the three-chamber glassware was exchanged for inert gas (argon or nitrogen) with the use of the Schlenk glassware. In the case the reaction was set up outside the glovebox the insertion of solvent in the third chamber is done with a syringe (the solvents should be layered inside the syringe).

With the optimized reaction conditions using 1 atmosphere of CO_2, we attempted to corroborate that the system was still very selective towards the carboxylic acid under low concentrations of CO_2 (Supplementary Figure 9). We first attempted to use the protons formed at the anodic chamber to react them directly with barium carbonate for CO_2 release. This was attempted with a simple divide cell as demonstrated in Supplementary Figure 9 (top right picture), with the addition of barium carbonate directly in the anodic chamber. This reaction failed, but since we still had the possibility of using ascorbic acid as the electron donor, we attempted to use it as both an electron donor for the reduction and a proton donor for the CO_2 release in the same glassware, but this was also a failed attempt. With this, we decided to use an additional third chamber that can be used specifically for the CO_2 release from barium carbonate and camphorsulfonic acid as demonstrated in Supplementary Figure 9 (bottom right picture). Analysis of the best solvent mixture for the gas release was also done, with layering ethylene glycol in water giving the best result (entry 6).

It is important to state that both glasswares have an upper gas bridge in between the anodic and cathodic chambers to avoid different overhead pressures inside the H-cell and prevent solvent exchange between chambers. For the case with the near stoichiometric CO_2 release, the cathodic chamber was chosen to be the one closer to the gas release chamber, as demonstrated in Supplementary Figure 10 and 11.

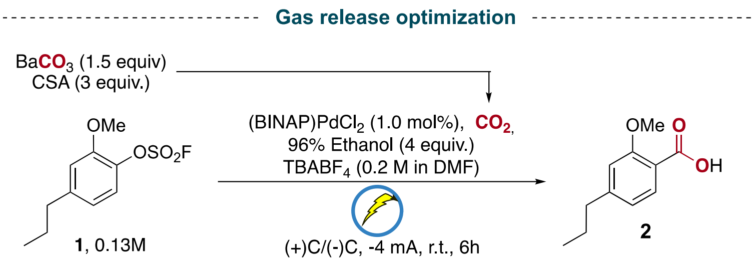

Entry	Change	Yield[a] **2** (%)
1	none	n.r.[b]
2	Ascorbic acid (2 equiv.) as electron donor	n.r.[b]
3	Ethylene Glycol:Water (4:1) in third chamber	85[c]
4	DMF:Water (4:1) in third chamber	80[c]
5	DCM:Water (2:1) in third chamber	42[c]
6	Ethylene Glycol:Water (2:1) in third chamber	85[c]

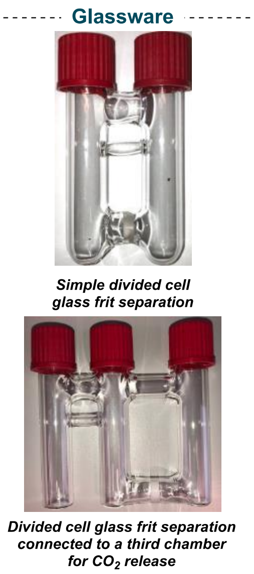

Supplementary Figure 9 Glassware and solvent screening for gas release.

[a] NMR yield calculated with 1,3,5-trimethoxybenzene as internal standard. [b] Reaction performed with simple divided cell (two-chamber electro glassware top figure). [c] Reaction performed with three-chamber electro glassware (lower figure).

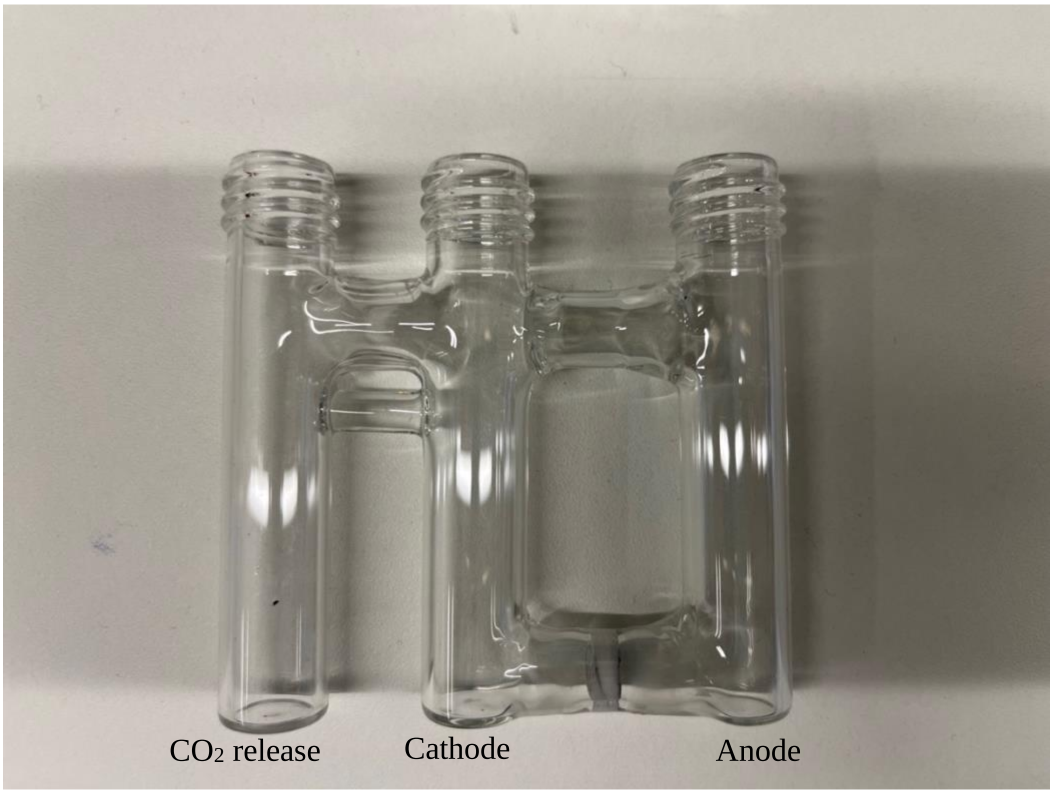

Supplementary Figure 10 Three-chamber glassware used for the stoichiometric release of CO_2 for electrocarboxylation

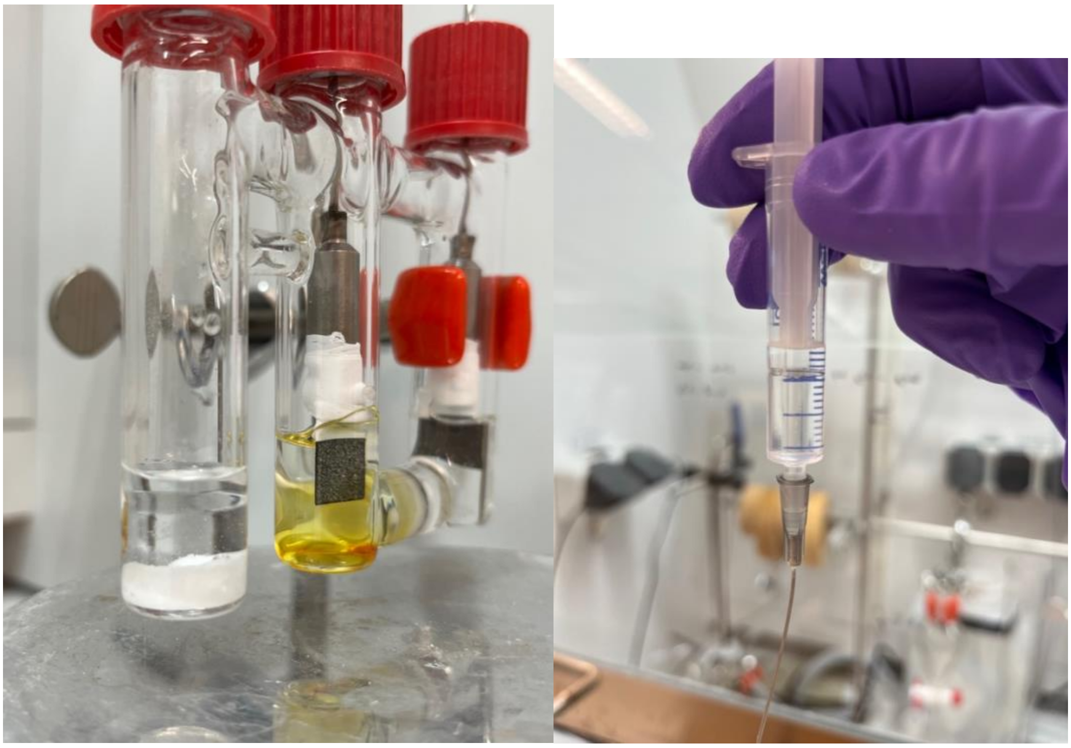

Supplementary Figure 11 Photos of layered solvents in the syringe and inside the gas release chamber.

3 Attempts with published catalytic reductive carboxylation methods:

Published catalytic reductive carboxylation (CRC) reactions rely on the use of atmospheric (or higher) pressures of CO_2. In order to observe if these reactions operate at lower pressures, the published methods were performed in a two-chamber setup with 1.5 equivalents of CO_2.

3.1 Procedure for the trial with Martin[4] conditions with near-stoichiometric CO_2:

A flame-dried two-chamber glassware (20 mL total volume) was taken inside the glovebox and the gas release chamber was charged with barium carbonate (148 mg, 1.5 equiv.) and camphorsulfonic acid (348.5 mg, 3 equiv.). The reaction chamber was charged with palladium acetate (5.5 mg, 5 mol%), *t*BuXPhos (21.2 mg, 10 mol%), and 2 mL of DMA. Next **1** (124 mg, 0.5 mmol, 1 equiv.) was weighted directly into the reaction chamber and both sides were closed with a pierce-through Teflon® seal and taken outside the glovebox. Once outside the glovebox, the reaction chamber was charged with Et_2Zn (1.0 M in hexanes, 1 mL – added *via* syringe) and the CO_2 release side was charged with layered ethylene glycol/H_2O (1.25 mL - 4:1). The reaction was displaced in a pre-heated heat block and let it stir for 20 hours at 40 ^{o}C. Once the time was completed the contents of the reaction was transferred to an extraction funnel with 1 M HCl (10 mL) and extracted 4 times with diethyl ether, 80.4 mg of 1,3,5-trimethoxybenzene was added as

internal standard and the organic phase was dried over magnesium sulfate and concentrated. With ^{1}H NMR analysis it was possible to observe 19% product **2** formation.

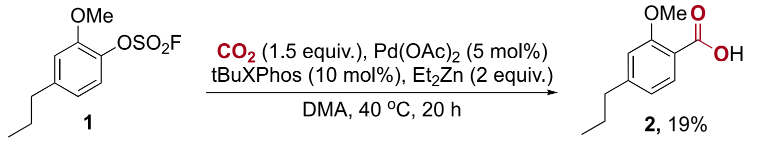

3.2 Procedure for the trial with Tsuji[5] conditions with near-stoichiometric CO_2:

A flame-dried two-chamber glassware (20 mL total volume) was taken inside the glovebox and the gas release chamber was charged with barium carbonate (148 mg, 1.5 equiv.) and camphorsulfonic acid (348.5 mg, 3 equiv.). The reaction chamber was charged with $NiCl_2(PPh_3)_2$ (16.4 mg, 5 mol%), PPh_3 (13.1 mg, 10 mol%), Mn powder (82.4 mg, 3 equiv.), and tetraethylammonium iodide (12.9 mg, 10 mol%). Then 0.75 mL of DMI was added followed by weighting **1** (124 mg, 0.5 mmol, 1 equiv.) directly in the reaction chamber, and both sides were closed with a pierce-through Teflon® seal and taken outside the glovebox. Once outside the glovebox, the CO_2 release side was charged with layered ethylene glycol/H_2O (1.25 mL - 4:1). The reaction was allowed to stir at 25 °C for 20 hours. Once the time was completed the contents of the reaction was transferred to an extraction funnel with 1 M HCl (10 mL) and extracted 4 times with diethyl ether, 76.6 mg of 1,3,5-trimethoxybenzene was added as internal standard and the organic phase was dried over magnesium sulfate and concentrated. With ^{1}H NMR analysis no product **2** could be observed.

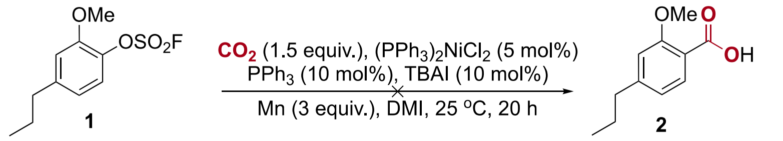

3.3 Procedure for the trial with Mei[6] conditions with near-stoichiometric CO_2:

A flame-dried two-chamber glassware (20 mL total volume) was taken inside the glovebox and one chamber was charged with barium carbonate (148 mg, 1.5 equiv.) and camphorsulfonic acid (348.5 mg, 3 equiv.). The other chamber was charged with $NiCl_2(PPh_3)_2$ (16.4 mg, 5 mol%), neocuproine (10.4 mg, 10 mol%), Mn powder (82.4 mg, 3 equiv.) and 0.5 mL of DMF, then the reaction was allowed to stir for 10 minutes at which time the solution had a dark blue color. Then **1** (124 mg, 0.5 mmol, 1 equiv.) was weighted directly in the reaction chamber followed by addition of another 0.5 mL of DMF, then both sides were closed with a pierce-through Teflon® seal and taken outside the glovebox. Once outside the glovebox, the CO_2 release side was charged with layered ethylene glycol/H_2O (1.25 mL - 4:1). The reaction was allowed to stir at 25 °C for 20 hours. Once the time was completed the contents of the reaction was transferred to an extraction funnel with 1 M HCl (10 mL) and extracted 4 times with diethyl ether, 80.0 mg of 1,3,5-trimethoxybenzene was added as internal standard and the organic phase was

dried over magnesium sulfate and concentrated. With ^{1}H NMR analysis it was possible to observe 23% product **2** formation.

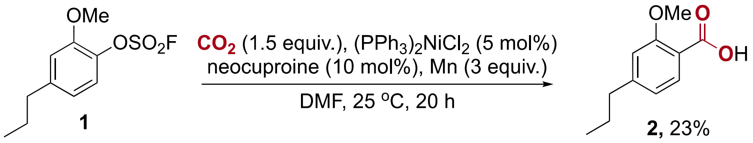

3.4 Procedure for the trial with Iwasawa[7,8] conditions with near-stoichiometric CO_2:

A flame-dried two-chamber glassware (20 mL total volume) was taken inside the glovebox and the gas release chamber was charged with barium carbonate (59.2 mg, 1.5 equiv.) and camphorsulfonic acid (139.4 mg, 3.0 equiv.). The reaction chamber was charged with palladium acetate (1.1 mg, 2.5 mol%), *t*BuXPhos (4.2 mg, 5 mol%), Ir(ppy)$_2$(dtbpy)(PF$_6$) (4.6mg, 2.5 mol%), cesium carbonate (195 mg, 3.0 equiv.), and 0.5 mL of DMA. Then **S1** (49.7 mg, 0.2 mmol, 1.0 equiv.) was weighted directly in the reaction chamber followed by the addition of DIPEA (100 μL, 3.0 equiv.), and another 0.5 mL of DMA, then both sides were closed with a pierce-through Teflon® seal and taken outside the glovebox. Once outside the glovebox, the CO_2 release side was charged with layered ethylene glycol:H_2O (1.25 mL - 4:1). The reaction was allowed to stir for 6 hours being irradiated with blue LEDs (Kessil LED A160WE Tuna Blue 40 W) with 5 cm between the center of the reaction chamber and the light source (a fan was displaced to keep the reaction from heating). Once the time was completed the contents of the reaction was transferred to an extraction funnel with 1 M HCl (10 mL) and extracted 4 times with diethyl ether, 33.9 mg of 1,3,5-trimethoxybenzene was added as internal standard and the organic phase was dried over magnesium sulfate and concentrated. With ^{1}H NMR analysis it was possible to observe 68% product **2** formation.

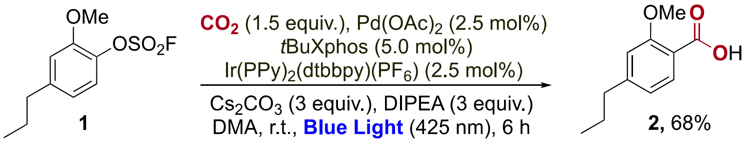

3.5 Procedure for the trial with Jana[9] conditions with near-stoichiometric CO_2:

A flame-dried two-chamber glassware (20 mL total volume) was taken inside the glovebox and the gas release chamber was charged with barium carbonate (59.2 mg, 1.5 equiv.) and camphorsulfonic acid (139.4 mg, 3.0 equiv.). The reaction chamber was charged with palladium acetate (2.2 mg, 5.0 mol%), DavePhos (7.8 mg, 10 mol%), Ir(ppy)$_2$(dtbpy)(PF$_6$) (3.6mg, 2.0 mol%), cesium carbonate (130 mg, 2.0 equiv.), and 1.0 mL of DMA. Then **1** (49.7 mg, 0.2 mmol, 1.0 equiv.) was weighted directly in the reaction chamber followed by the addition of DIPEA (70 μL, 2.0 equiv.), and another 1.0 mL of DMA, then both sides were closed with a pierce-through Teflon® seal and taken outside the glovebox. Once outside the glovebox, the CO_2 release side was charged with layered

ethylene glycol:H_2O (1.25 mL - 4:1). The reaction was allowed to stir for 6 hours being irradiated with blue LEDs (Kessil LED A160WE Tuna Blue 40 W) with 5 cm between the center of the reaction chamber and the light source (a fan was displaced to keep the reaction from heating). Once the time was completed the contents of the reaction was transferred to an extraction funnel with 1 M HCl (10 mL) and extracted 4 times with diethyl ether, 29.5 mg of 1,3,5-trimethoxybenzene was added as internal standard and the organic phase was dried over magnesium sulfate and concentrated. With ^1H NMR analysis it was possible to observe 26% product **2** formation.

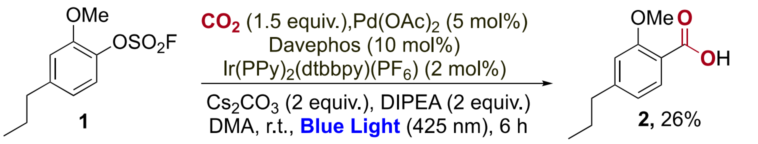

3.6 Procedure for the trial with König[10] conditions with near-stoichiometric CO_2:

A flame-dried two-chamber glassware (20 mL total volume) was taken inside the glovebox and the gas release chamber was charged with barium carbonate (59.2 mg, 1.5 equiv.) and camphorsulfonic acid (139.4 mg, 3.0 equiv.). The reaction chamber was charged with $NiBr_2$.glyme (6.2 mg, 10 mol%), neocuproine (8.3 mg, 20 mol%), 4CzIPN (1.6 mg, 1 mol%), potassium carbonate (27.6 mg, 1 equiv.), 4 Å molecular sieves (50 mg), Diethyl 1,4-dihydro-2,6-dimethyl-3,5-pyridinedicarboxylate (Hantzsch ester – HEH – 101.3 mg, 2 equiv.), and 2 mL of DMF. Then **1** (49.7 mg, 0.2 mmol, 1.0 equiv.) was weighted directly in the reaction chamber followed by the addition of another 2.0 mL of DMF, then both sides were closed with a pierce-through Teflon® seal and taken outside the glovebox. Once outside the glovebox, the CO_2 release side was charged with layered ethylene glycol/H_2O (1.25 mL - 4:1). The reaction was allowed to stir for 24 hours being irradiated with blue LEDs (Kessil LED A160WE Tuna Blue 40 W) with 5 cm between the center of the reaction chamber and the light source (a fan was displaced to keep the reaction from heating). Once the time was completed the contents of the reaction was transferred to an extraction funnel with 1 M HCl (10 mL) and extracted 4 times with diethyl ether, 40.6 mg of 1,3,5-trimethoxybenzene was added as internal standard and the organic phase was dried over magnesium sulfate and concentrated. With ^1H NMR analysis it was possible to observe 7% product **2** formation.

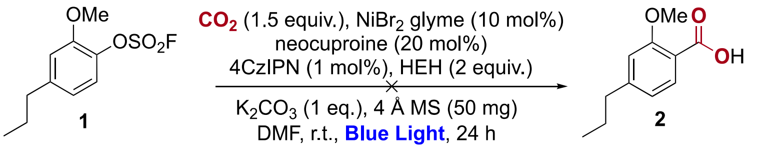

3.7 Procedure for the trial with Yu[11] conditions with near-stoichiometric CO_2:

Outside the glovebox the electrodes were prepared, a 2x1 cm piece of carbon paper (Toray paper 090 waterproofed) was attached to an aluminum holder (**Error! Reference source not found.**). In order to define the available area the electrode was covered using

Teflon® tape to leave an area of 2 cm^2 available (1x1 cm surface area with both sides uncovered), this was pierced through a Teflon® inlay and taken inside the glovebox together with the oven dried (120 °C) glassware. Once inside the glovebox, the release chamber was charged with barium carbonate (89 mg, 0.45 mmol, 1.5 equiv.) and camphorsulfonic acid (209 mg, 0.9 mmol, 3 equiv.). The cathodic chamber was charged with $Ni(acac)_2$ (7.7 mg, 0.03 mmol, 10 mol%), 4,4'-di-tert-butyl-2,2'-bipyridine (8.1 mg, 0.03 mmol, 10 mol%), KOtBu (16.8 mg, 0.15 mmol, 0.5 equiv.), 4 Å molecular sieve (100 mg), NaI (180.0 mg, 1.2 mmol, 4 equiv.). The anodic chamber was charged with LiCl (1.8 mmol) followed by 5 mL of NMP in each chamber. Then, **1** (0.3 mmol, 74.5 mg, 1 equiv.) was weighted inside the cathodic chamber followed by the addition of toluene (0.6 mmol) in the anodic chamber and the addition of the leftover 1 mL of NMP in each chamber. After that, ethylene glycol and water (1.25 mL 4:1) were layered in the release chamber and the reaction was closed and taken outside the glovebox. Once outside the glovebox the reaction was allowed to stir until a clear solution is obtained followed by applying -3 V (no reference electrode, cell potential) for 16 hours. With ^1H NMR analysis no product **2** could be observed.

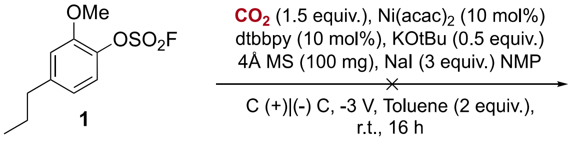

3.8 Procedure for the trial with Wang[12] conditions with near-stoichiometric CO_2:

Outside the glovebox the electrodes were prepared, a 2x1 cm piece of carbon paper (Toray paper 090 waterproofed) was attached to an aluminum holder (Figure 1). In order to define the available area the electrode was covered using Teflon® tape to leave an area of 2 cm^2 available (1x1 cm surface area with both sides uncovered), this was pierced through a Teflon® inlay and taken inside the glovebox together with the oven dried (120 °C) glassware. Once inside the glovebox, the release chamber was charged with barium carbonate (141 mg, 0.60 mmol, 1.5 equiv.) and camphorsulfonic acid (279 mg, 0.9 mmol, 3 equiv.). The cathodic chamber was charged with CoBr (8.8 mg, 0.04 mmol, 10 mol%), neocuproine (8.3 mg, 0.04 mmol, 10 mol%), $TBABF_4$ (198.0 mg, 0.60 mmol, 1.5 equiv.). The anodic chamber was charged with $TBABF_4$ (198.0 mg, 0.60 mmol, 1.5 equiv.) and ascorbic acid (141 mg, 2 equiv.), followed by the addition of 2 mL of DMF in the anodic chamber and 2 mL in the cathodic chamber (solvent addition was made using a 5mL single-use plastic syringe). After this, the starting material was weighted directly into the cathodic chamber (for liquid starting materials) and the last 1 mL of solvent is used to rinse the walls of the cathodic chamber, the solvent at the anodic chamber was leveled with the one over the cathodic side. The electrodes were rinsed with DMF before introducing them into the solution, then the setup is closed and taken outside of the glovebox. Once outside the glovebox 1.25mL of layered ethylene glycol/water (4:1) is

added through the wall of the gas release chamber, this is added with a syringe with the pierce trough septum (the solvents were layered inside the syringe). The reaction is allowed to stir for 10 minutes for the gas release to occur and for everything to become soluble. Then the Electroware is attached and applies a constant current for 2.2 mols of electrons. Once the reaction is completed it is quenched in acid and extracted with diethyl ether for ^{1}H NMR analysis. With ^{1}H NMR analysis no product **2** could be observed.

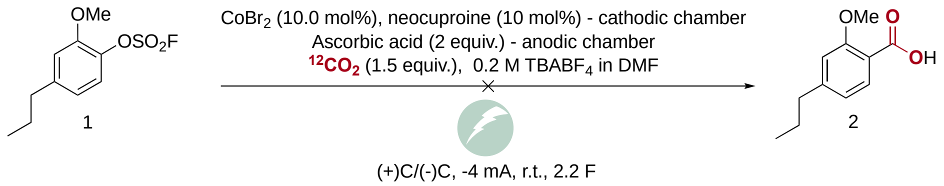

3.9 Procedure for the trial with Ackermann[13] conditions with near-stoichiometric CO_2:

Outside the glovebox the electrodes were prepared, a 2x1 cm piece of carbon paper (Toray paper 090 waterproofed) was attached to an aluminum holder (Figure 1). In order to define the available area the electrode was covered using Teflon® tape to leave an area of 2 cm^2 available (1x1 cm surface area with both sides uncovered), this was pierced through a Teflon® inlay and taken inside the glovebox together with the oven dried (120 ^{o}C) glassware. Once inside the glovebox, the release chamber was charged with barium carbonate (141 mg, 0.60 mmol, 1.5 equiv.) and camphorsulfonic acid (279 mg, 0.9 mmol, 3 equiv.). The cathodic chamber was charged with $Co(OAc)_2$ (7.1 mg, 0.04 mmol, 10 mol%), PPh_3 (21.0 mg, 0.08 mmol, 20 mol%), $TBABF_4$ (198.0 mg, 0.60 mmol, 1.5 equiv.). The anodic chamber was charged with $TBABF_4$ (198.0 mg, 0.60 mmol, 1.5 equiv.) and ascorbic acid (141 mg, 2 equiv.), followed by the addition of 2 mL of DMF in the anodic chamber and 2 mL in the cathodic chamber (solvent addition was made using a 5 mL single-use plastic syringe). After this, the starting material was weighted directly into the cathodic chamber (for liquid starting materials) and the last 1 mL of solvent is used to rinse the walls of the cathodic chamber, the solvent at the anodic chamber was leveled with the one over the cathodic side. The electrodes were rinsed with DMF before introducing them into the solution, then the setup is closed and taken outside of the glovebox. Once outside the glovebox 1.25mL of layered ethylene glycol/water is added through the wall of the gas release chamber, this is added with a syringe with the pierce trough septum (the solvents were layered inside the syringe). The reaction is allowed to stir for 10 minutes for the gas release to occur and for everything to become soluble. Then the Electroware is attached and applies a constant current for 2.2 mols of electrons. Once the reaction is completed it is quenched in acid and extracted with diethyl ether for ^{1}H NMR analysis. With ^{1}H NMR analysis no product **2** could be observed.

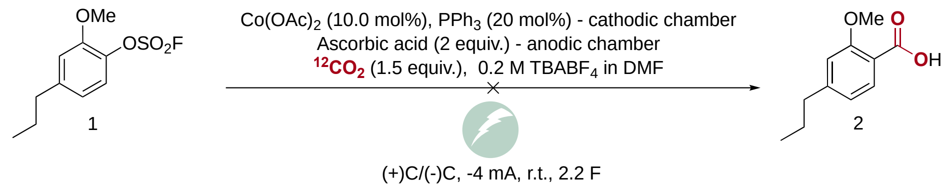

4 Mechanistic investigation (Experimental and DFT)

All plausible mechanisms go through a basic oxidative addition of an aryl halide, the major difference being which palladium complex is involved in the CO_2 insertion. Amatore, Jutand, and co-workers were the first to propose a mechanism for the Pd-catalysed carboxylation of aryl electrophiles.[14] For the catalytic system presented by Jutand and co-workers, $(PPh_3)_2PdCl_2$, an overall two-electron reduction of (L)Pd(Ar)(X) **III** is demonstrated which leads to $[(L)Pd(OCOAr)]^-$ **VII**. After in-depth experimental study they proposed that **VII** is in equilibrium with **I** and free aryl anion, which reacts promptly in the presence of CO_2, thus the organometallic catalyst does not play any chemical role in the carboxylation step.[14] Later, Martin and co-workers proposed that **III** (X = Br) is performing the CO_2 insertion, but with no experimental proof.[4] Iwasawa and co-workers proposed that (L)Pd(Ar) **V** is responsible for promoting CO_2 insertion, based on DFT and experimental investigation.[15]

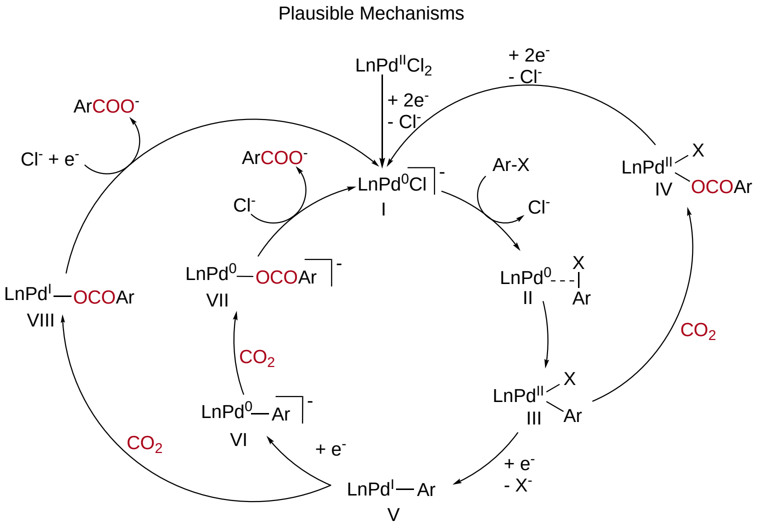

Supplementary Figure 12 Plausible mechanisms based on previous literature reports.

4.1 Computational details:

All density functional theory (DFT) results presented in this paper were performed in the Gaussian 16 package, at the temperature of 298.15 K and 1 atm pressure.[2] The geometry optimization of all molecules was executed at the B3LYP-D3/Def2SVP level of theory with the integral equation formalism polarizable continuum model (IEFPCM) for *N*,*N*-Dimethylformamide (DMF).[16–22] To check all minima and saddle points, vibrational analysis was performed at the same level as the geometry optimization, this also provided

the Gibbs free energy corrections. Besides the vibrational analysis, the transition states were further confirmed through IRC calculations. Single-point calculations of all structures were done at the B3LYP-D3/Def2TZVPP level of theory with IEFPCM for DMF. All structures were treated as full models with no symmetry constraints, using the ultrafine grid and tight convergence criteria.

4.1.1 DFT data:

We first investigated the ability of LPd(II)ArBr complexes to perform CO_2 insertion and observed that the barriers were too high for a room-temperature reaction with atmospheric pressure (Supplementary Figure 13).

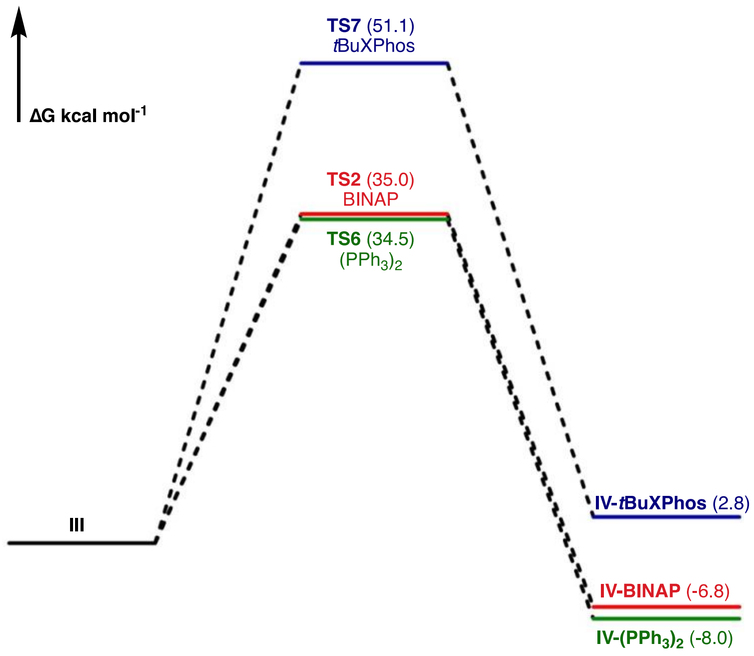

Supplementary Figure 13 Comparison of the barrier for CO_2 insertion of different LPd^{II} ligand systems that have been previously published in the literature and the developed one. All the presented barriers were calculated at the B3LYP-D3/def2-TZVPP//B3LYP-D3/def2-SVP with IEFPCM for DMF.

For the BINAP ligand, we also computed the carboxylation step for 3 different oxidative addition complexes that can be present in the reaction medium (X = Br or Cl or OSO_2F), but all have similar barriers. With the CV studies, it was possible to demonstrate that it is feasible to achieve a palladium(I) oxidation state with the BINAP ligand. Thus, we investigated the difference between **V** and **VI** in performing CO_2 insertion. Although **VI** has a smaller barrier both barriers can be overcome at room temperature (Supplementary Figure 15).

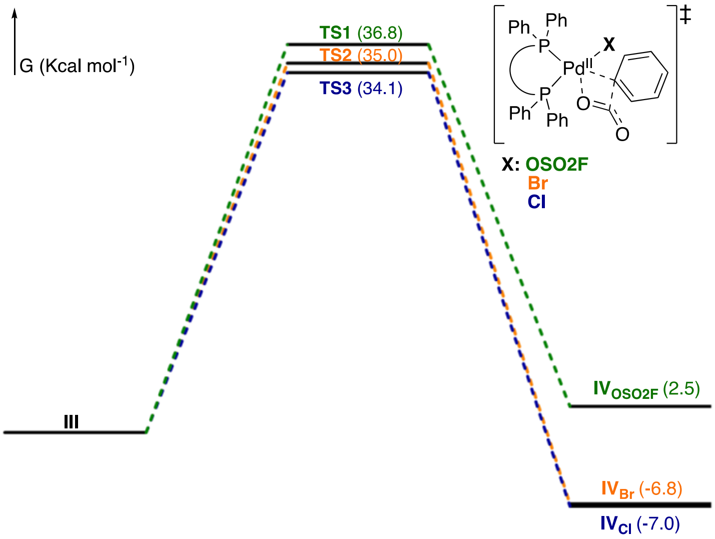

Supplementary Figure 14 Calculated barriers for CO_2 insertion with the different palladium species that can be present in the reaction medium for the (BINAP)Pd ligand system. All the presented barriers were calculated at the B3LYP-D3/def2-TZVPP//B3LYP-D3/def2-SVP with IEFPCM for DMF.

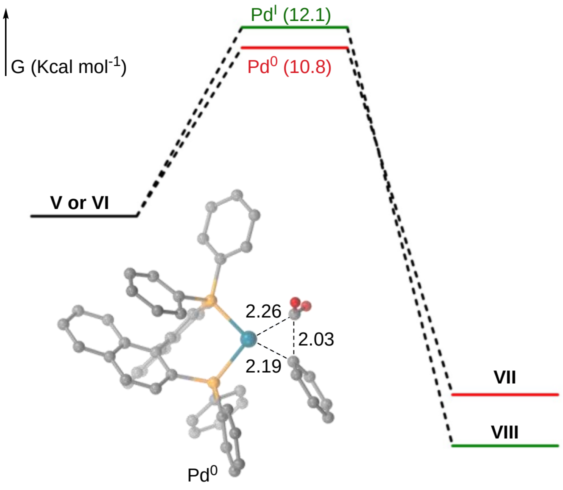

Supplementary Figure 15 Calculated barriers for CO_2 insertion with the different palladium species that can be present in the reaction medium for the (BINAP)Pd ligand system. All the presented barriers were calculated at the B3LYP-D3/def2-TZVPP//B3LYP-D3/def2-SVP with IEFPCM for DMF.

4.1.2 Imaginary frequencies for the transition states:

Transition State	Frequency
TS1	386.9*i*
TS2	370.7*i*
TS3	372.5*i*
TS4	240.1*i*
TS5	293.3*i*
TS6	388.7*i*
TS7	293.5*i*

Supplementary Table 1 Imaginary frequencies for the transition states

4.1.3 Computed energies:

Transition State	G kcal mol^{-1}	H kcal mol^{-1}	E kcal mol^{-1}
III-OSO2F	-2291134.5484645	-2291042.954172	-2291544.7125845
TS1	-2291097.7453385	-2291009.5652735	-2291510.491621
IV-OSO2F	-2291132.0818875	-2291044.40194	-2291546.9723375
III-Br	-3452207.651344	-3452120.8323265	-3452610.271659
TS2	-3452172.6093595	-3452088.841247	-3452577.3619195
IV-Br	-3452214.418304	-3452130.103639	-3452620.2721265
III-Cl	-2125696.4889395	-2125610.237182	-2126099.657062
TS3	-2125662.357332	-2125579.4037145	-2126067.949487

IV-Cl	-2125703.50169125	-2125619.82017375	-2126110.01501625
V	-1836855.409542	-1836769.2017095	-1837255.9245945
TS4	-1836843.33343	-1836760.213525	-1837245.768005
VIII	-1836870.10188975	-1836787.82283475	-1837275.29432725
VI	-1836936.76585825	-1836852.85969575	-1837338.50767325
TS5	-1836925.99268725	-1836843.61636975	-1837327.97859975
VII	-1836948.18209125	-1836866.78718375	-1837353.06830875
III-PPh3	-3260109.67118325	-3260027.03759075	-3260468.70974075
TS6	-3260075.19947075	-3259997.31354325	-3260438.12664575
IV-PPh3	-3260117.7101485	-3260037.1153035	-3260479.6797585
III-tBuXPHOS	-2884053.5201935	-2883972.1823885	-2884489.831611
TS7	-2884002.44464275	-2883925.75410275	-2884442.57063275
IV-tBuXPHOS	-2884050.71859425	-2883973.18720425	-2884491.73375175

Supplementary Table 2 Energy of molecules

Transition State	ΔG kcal mol^{-1}	ΔH kcal mol^{-1}
III-OSO2F	0	0
TS1	36.8	33.4
IV-OSO2F	2.5	-1.4

Supplementary Table 3 Variation of Gibbs free energy and enthalpy for TS1

Transition State	ΔG kcal mol^{-1}	ΔH kcal mol^{-1}
III-Br	0	0
TS2	35.0	32.0
IV-Br	-6.8	-9.3

Supplementary Table 4 Variation of Gibbs free energy and enthalpy for TS2

Transition State	ΔG kcal mol^{-1}	ΔH kcal mol^{-1}
III-Cl	0	0
TS3	34.1	30.1
IV-Cl	-7.0	-9.6

Supplementary Table 5 Variation of Gibbs free energy and enthalpy for TS3

Transition State	ΔG kcal mol^{-1}	ΔH kcal mol^{-1}
V	0	0
TS4	12.1	9.0
VIII	-14.7	-18.6

Supplementary Table 6 Variation of Gibbs free energy and enthalpy for TS4

Transition State	ΔG kcal mol^{-1}	ΔH kcal mol^{-1}
VI	0	0
TS5	10.8	9.2
VII	-11.4	-13.9

Supplementary Table 7 Variation of Gibbs free energy and enthalpy for TS5

Transition State	ΔG kcal mol^{-1}	ΔH kcal mol^{-1}
III-PPh_3	0	0
TS6	34.5	29.7
IV-PPh_3	-8.0	-10.1

Supplementary Table 8 Variation of Gibbs free energy and enthalpy for TS6

Transition State	ΔG kcal mol^{-1}	ΔH kcal mol^{-1}
III-tBuXPHOS	0	0
TS7	51.1	46.4
IV-tBuXPHOS	2.8	-1.0

Supplementary Table 9 Variation of Gibbs free energy and enthalpy for TS7

4.1.4 Intrinsic reaction coordinate (IRC):

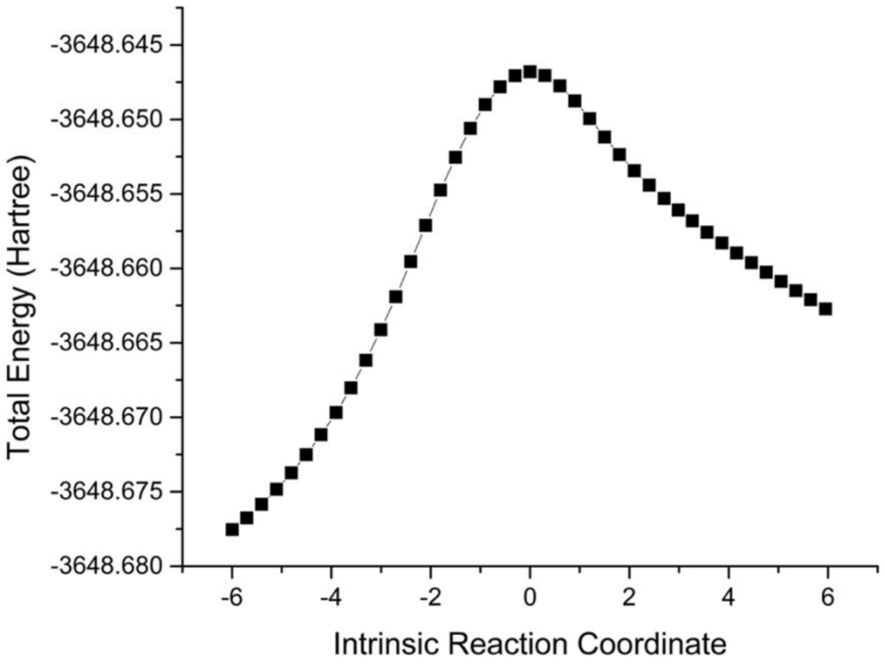

Supplementary Figure 16 IRC calculation for TS1

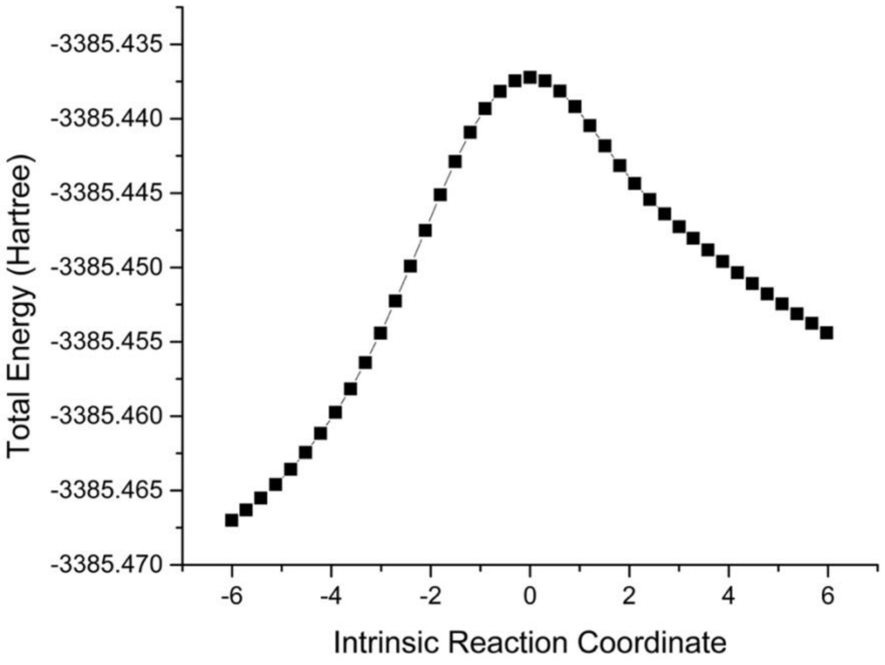

Supplementary Figure 17 IRC calculation for TS2

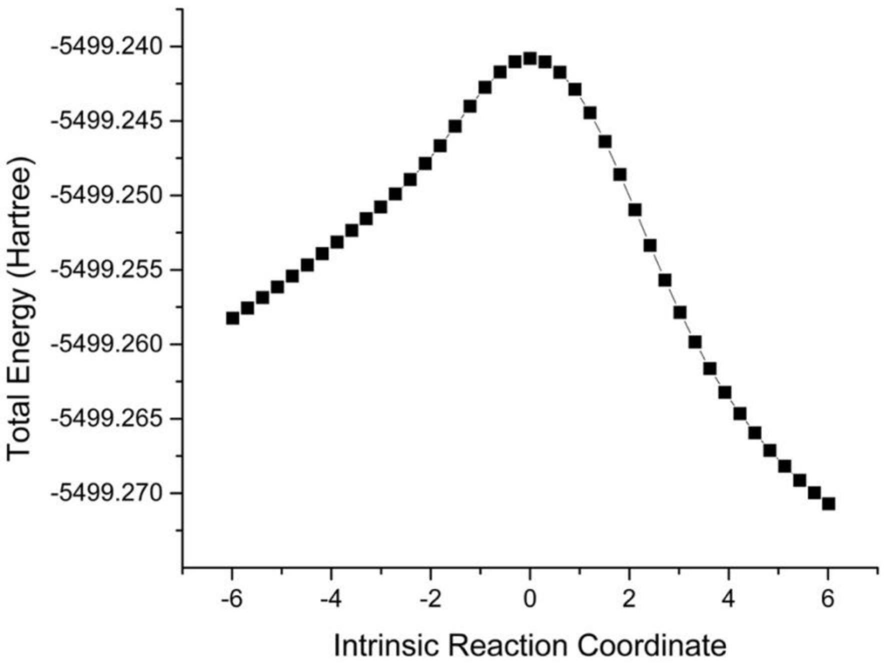

Supplementary Figure 18 IRC calculation for TS3

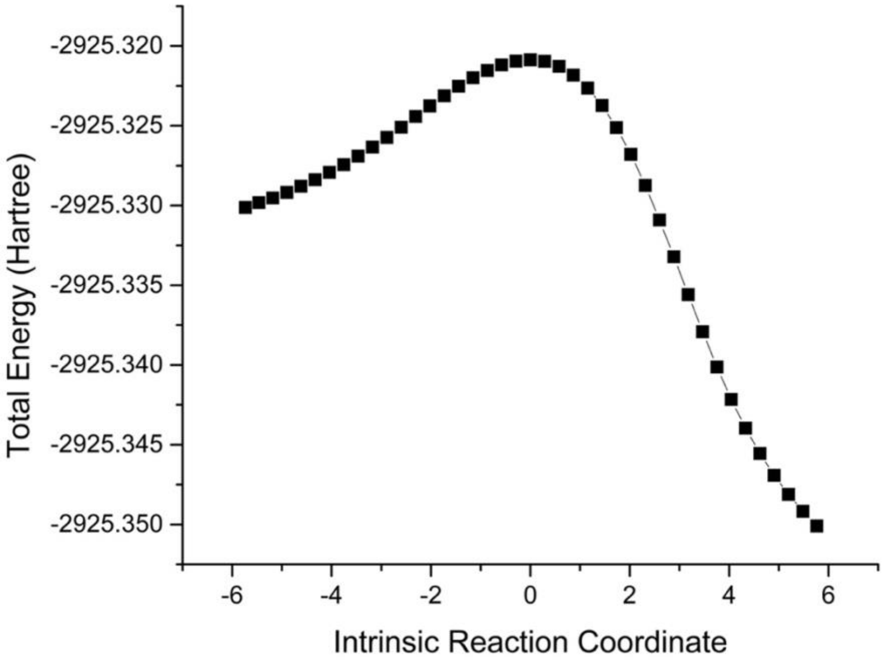

Supplementary Figure 19 IRC calculation for TS4

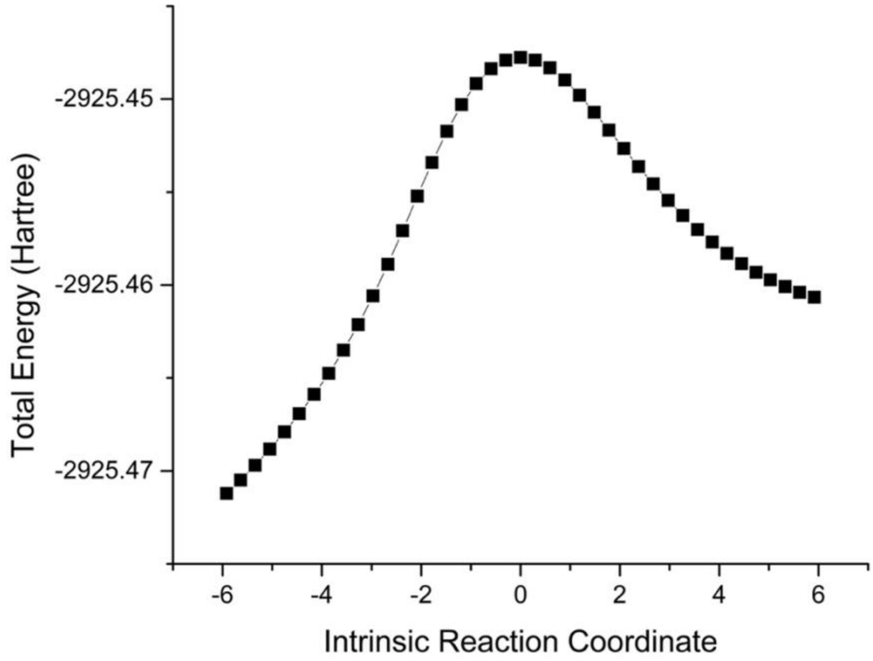

Supplementary Figure 20 IRC calculation for TS5

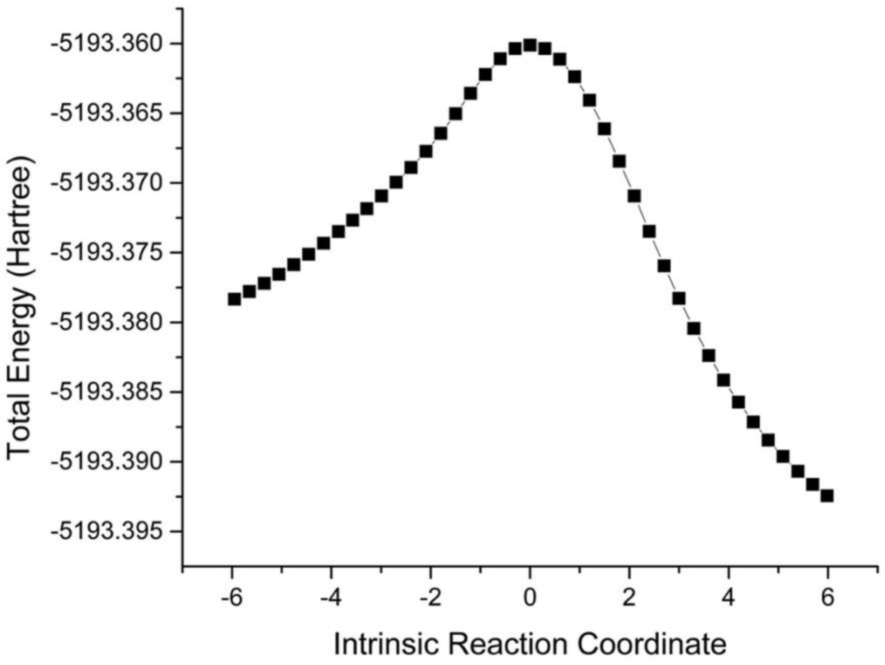

Supplementary Figure 21 IRC calculation for TS6

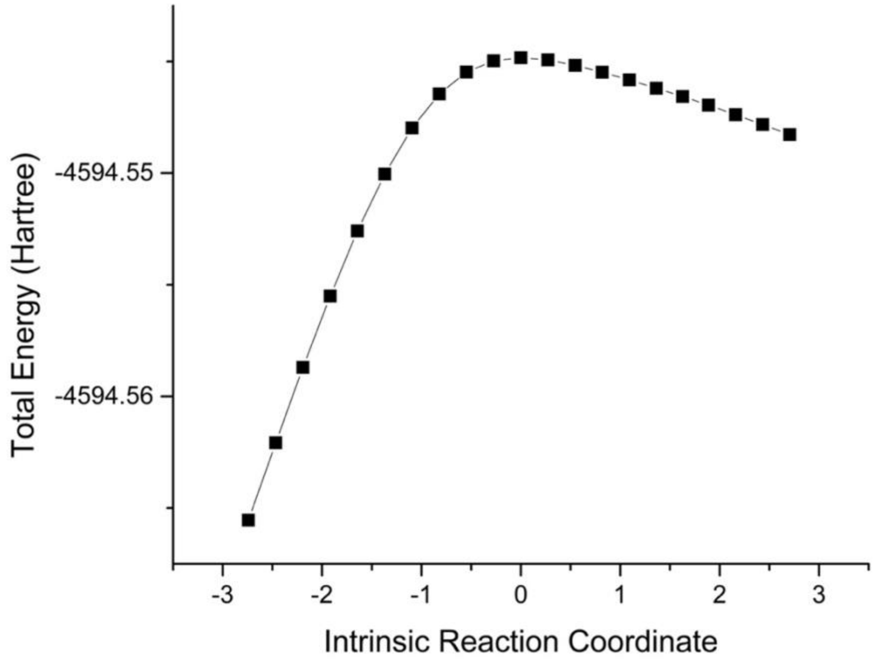

Supplementary Figure 22 IRC calculation for TS7

4.2 General Procedure for cyclic voltammetry data:

A stock solution of electrolyte in the desired solvent is prepared and degassed by bubbling argon for 10 minutes. This solution is transferred to a flame-dried three-neck glassware that was under an argon atmosphere (Supplementary Figure 23) and already contained the working (glassy carbon, 1 mm diameter), reference (leak-free saturated Ag/AgCl), and counter (platinum wire) electrodes. The CV's were measured and in between analyses, the working electrode was cleaned by doing 20 times infinity loops on a microfiber polishing cloth with alumina slurry. For the electrode exchange, the glassware is connected to the argon line and kept under constant argon pressure. For the CV's reported in CO_2 atmosphere, the exchange is done by bubbling the solution with CO_2 for 5 minutes to saturate the solvent.

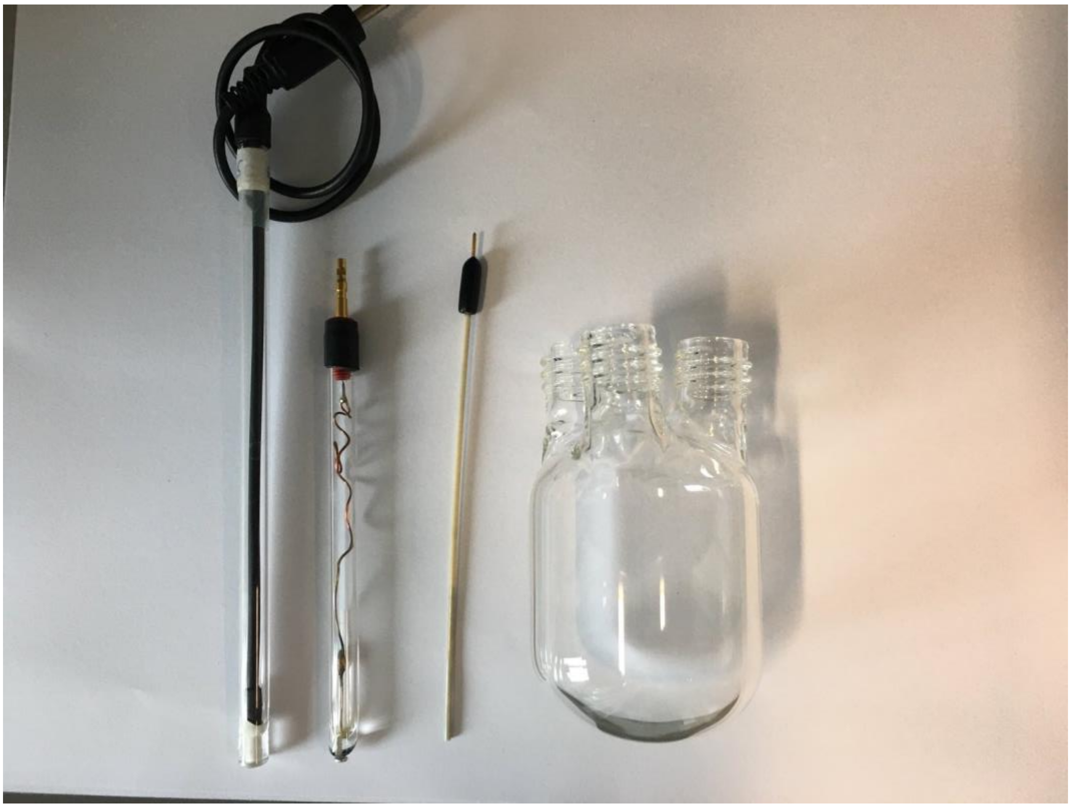

Supplementary Figure 23 Glassware and electrodes used for the obtention of CV data

4.2.1 Cyclic voltammetry data analysis:

The Randles–Ševčík equation (Supplementary Equation **1**) describes the effect of the scan rate in the peak current (i_p). With the same equation is possible to obtain the number of electrons transferred in a reduction or oxidation peak if the electrode area (A), diffusion coefficient (D_O), concentration (C_O), scan rate (v), and peak current are known.

$$(1)\ i_p = (2.69 \times 10^5) n^{3/2} A D_O^{1/2} C_O^{*} v^{1/2}$$

Supplementary Equation 1 Randles–Ševčík equation

To obtain the values of the diffusion coefficient for the pre-catalyst used for the catalytic reaction one can perform diffusion-ordered spectroscopy (DOSY) NMR. To decrease the errors related to the diffusion coefficient value, the D_O of ferrocene was calculated in the same manner in the same concentration which was used as an internal standard in the later cyclic voltammetry analysis. Data analysis was performed with the MestreNova software package using the peak fit DOSY transform (MestreNova) with 512 points in the diffusion dimension. Thus, we could obtain that the diffusion coefficient for (BINAP)$PdCl_2$ is 5.38E-6 (cm^2 s^{-1}) (Supplementary Figure 24, 25) and 1.26E-5 (cm^2 s^{-1}) for ferrocene (Supplementary Figure 26, 27).

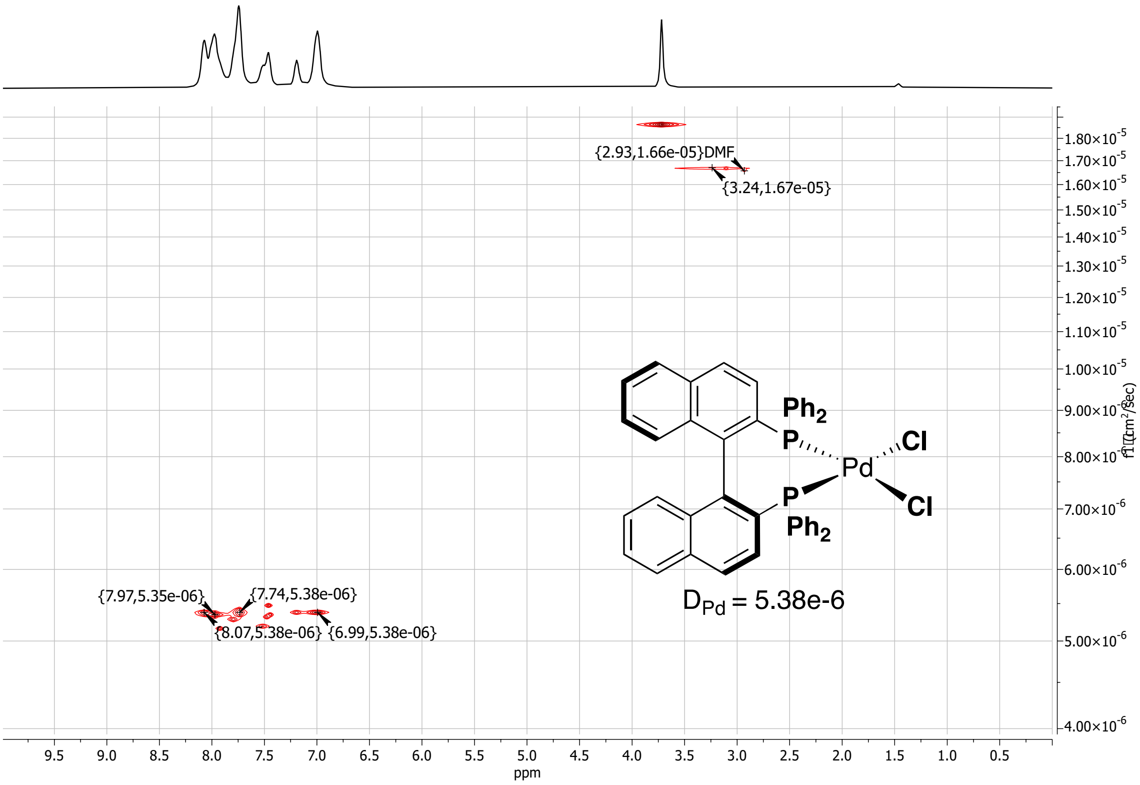

Supplementary Figure 24 Peak fit DOSY transform (MestreNova) with 512 points in the diffusion dimension of BINAPPdCl_2 in DMF

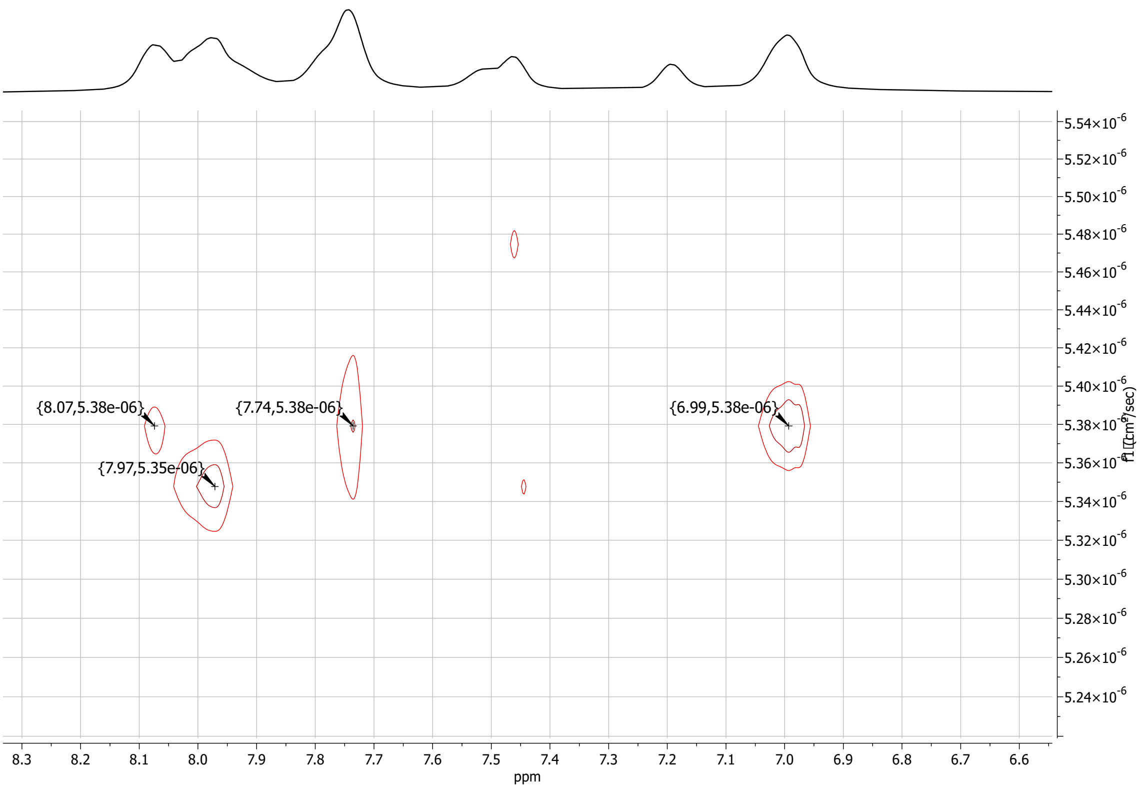

Supplementary Figure 25 Zoom of Peak fit DOSY transform (MestreNova) with 512 points in the diffusion dimension of $BINAPPdCl_2$ in DMF

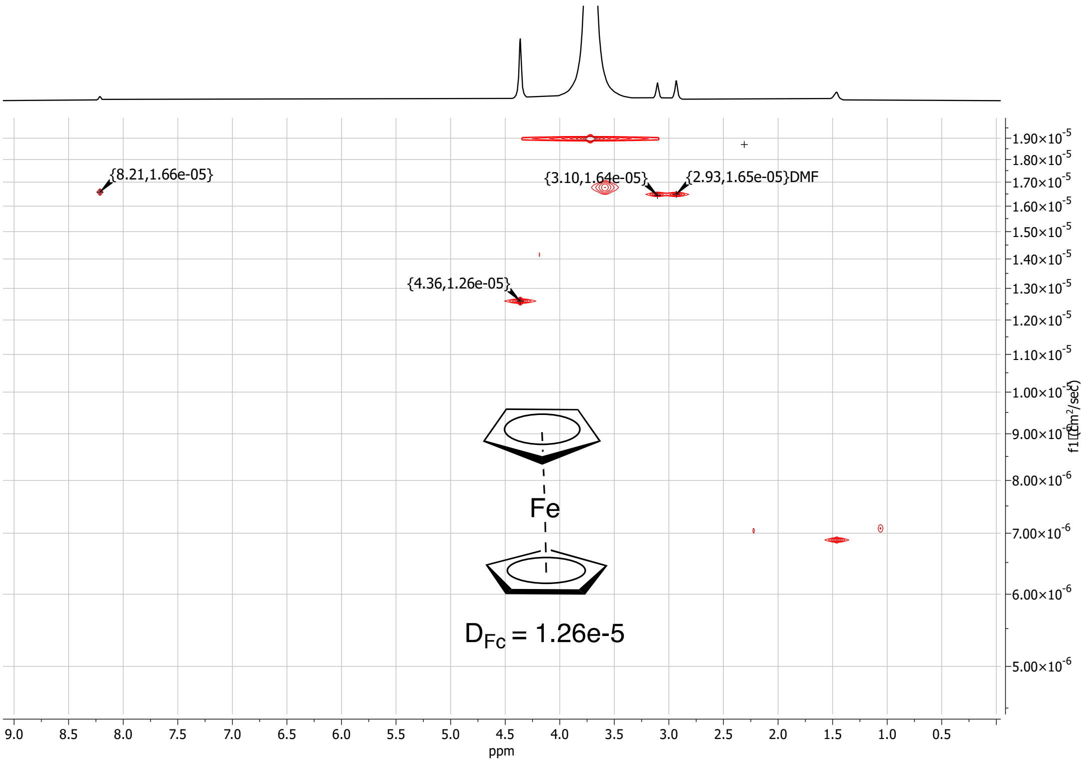

Supplementary Figure 26 Peak fit DOSY transform (MestreNova) with 512 points in the diffusion dimension of ferrocene in DMF

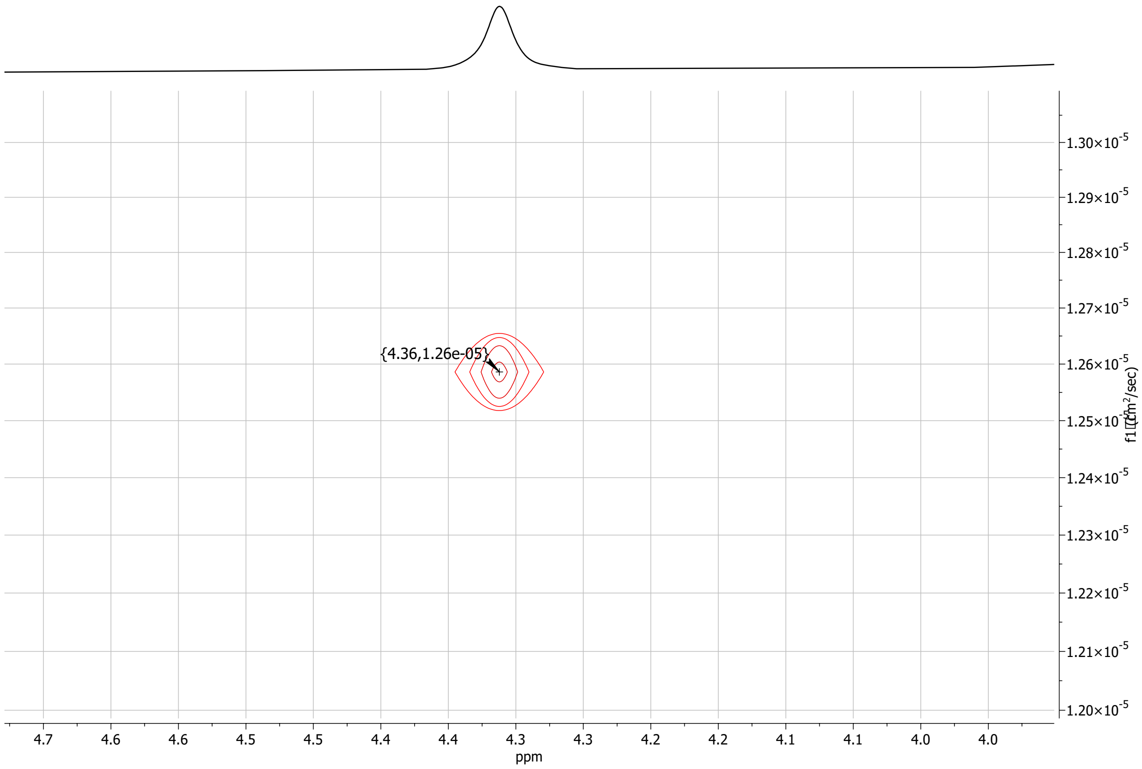

Supplementary Figure 27 Zoom of Peak fit DOSY transform (MestreNova) with 512 points in the diffusion dimension of ferrocene in DMF

Once the diffusion coefficients were determined one can obtain the correlation of the number of electrons transferred in the reduction peaks of (BINAP)$PdCl_2$ with a correlation from the Randles–Ševčík equation for ferrocene and the catalyst (**Supplementary Equation 2**). It is important to highlight that the cyclic voltammetry measurements were determined with both at the same concentration (canceling the concentration term), at the same solution (canceling the scan rate term), and with the same working electrode (canceling the electrode area term).

$$(2)\ i_{pFc} = (2.69 \times 10^5) 1^{\frac{3}{2}} A D_{Fc}^{\frac{1}{2}} C_O^* v^{\frac{1}{2}}$$

$$(3)\ i_{pPd} = (2.69 \times 10^5) n^{\frac{3}{2}} A D_{Pd}^{\frac{1}{2}} C_O^* v^{\frac{1}{2}}$$

$$(4)\ \frac{i_{pFc}}{i_{pPd}} = \frac{(2.69 \times 10^5) 1^{\frac{3}{2}} A D_{Fc}^{\frac{1}{2}} C_O^* v^{\frac{1}{2}}}{(2.69 \times 10^5) n^{\frac{3}{2}} A D_{Pd}^{\frac{1}{2}} C_O^* v^{\frac{1}{2}}}$$

$$(5)\ \frac{i_{pFc}}{i_{pPd}} = \frac{D_{Fc}^{\frac{1}{2}}}{n^{\frac{3}{2}} D_{Pd}^{\frac{1}{2}}}$$

$$(6)\ n^{\frac{3}{2}} = \left(\frac{D_{Fc}^{\frac{1}{2}} i_{pPd}}{D_{Pd}^{\frac{1}{2}} i_{pFc}}\right)$$

$$(7)\ n^{\frac{3}{2}} = \left(\frac{(1.26 \times 10^{-5})^{1/2}}{(5.38 \times 10^{-6})^{1/2}} \times \frac{i_{pPd}}{i_{pFc}}\right)$$

$$(8)\ n^{\frac{3}{2}} = \left(1.53 \times \frac{i_{pPd}}{i_{pFc}}\right)$$

$$(9)\ n = \left(1.53 \times \frac{i_{pPd}}{i_{pFc}}\right)^{\frac{2}{3}}$$

Supplementary Equation 2 Set of equations correlating the number of electrons transferred in the reduction peak of (BINAP)$PdCl_2$ with its peak current and the peak current of ferrocene at the same concentration in the same cyclic voltammogram.

After the determination of a formula to obtain the number of electrons for the reduction peak of the catalyst in correlation with that of ferrocene, a series of CV's with different scan rates was measured (Supplementary Figure 28 until 32). Two peak currents can be used for ferrocene, both the oxidation and reduction, although one needs to remind that the value needs to be normalized in the oxidation case, once the negative or positive value in the peak current is according to the used reference system to indicate oxidation or reduction. Applying the obtained formula for the first reduction wave (R^1) of palladium for the different scan rates leads to an average value of (1.11 ± 0.25). The same was applied for the second reduction wave R^2 and an average value of (1.13 ± 0.25) was found what confirms our previous assumption that is possible to access a palladium(I) intermediate with this ligand system.

Scan rate (mV s^{-1})	iP_{R1} (A)	iP_{Fc} oxid. (A)	iP_{Fc} red. (A)	n (from reduction)	n (from oxidation)
20	-3.37E-6	5.34E-06	-2.69E-06	1.54	9.77E-01
50	-4.74E-06	8.48E-06	-4.68E-06	1.34	9.01E-01
100	-6.21E-06	1.19E-05	-6.59E-06	1.28	8.63E-01
200	-8.44E-06	1.63E-05	-9.22E-06	1.25	8.57E-01
500	-1.28E-05	2.57E-05	-1.42E-05	1.24	8.36E-01

Supplementary Table 10 Summary of results for the number of electrons transferred at R^1

Scan rate (mV s^{-1})	iP_{R2} (A)	iP_{Fc} oxid. (A)	iP_{Fc} red. (A)	n (from reduction)	n (from oxidation)
20	-3.42E-06	5.34E-06	-2.69E-06	1.56	9.87E-01
50	-4.95E-06	8.48E-06	-4.68E-06	1.38	9.27E-01
100	-6.54E-06	1,19E-05	-6.59E-06	1.32	8.94E-01
200	-8.83E-06	1.63E-05	-9.22E-06	1.29	8.83E-01
500	-1.32E-05	2.57E-05	-1.42E-05	1.27	8.54E-01

Supplementary Table 11 Summary of results for the number of electrons transferred at R^2

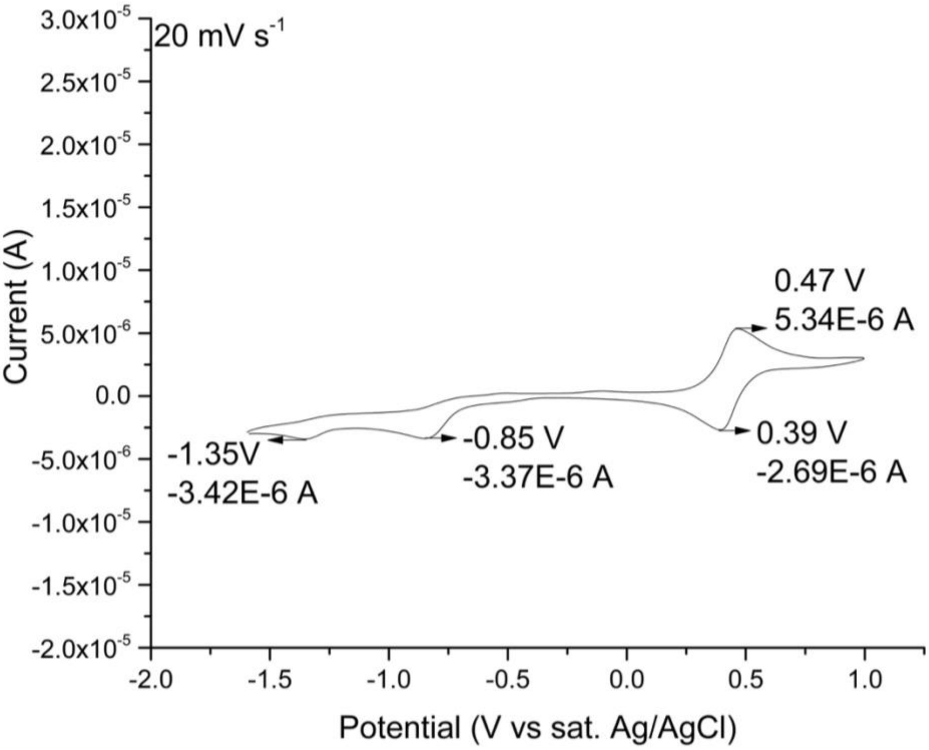

Supplementary Figure 28 Cyclic voltammogram of $(BINAP)PdCl_2$ (6 mM) and ferrocene (6 mM) in 0.2M $TBABF_4$ (DMF) measured at 20 mV s^{-1}

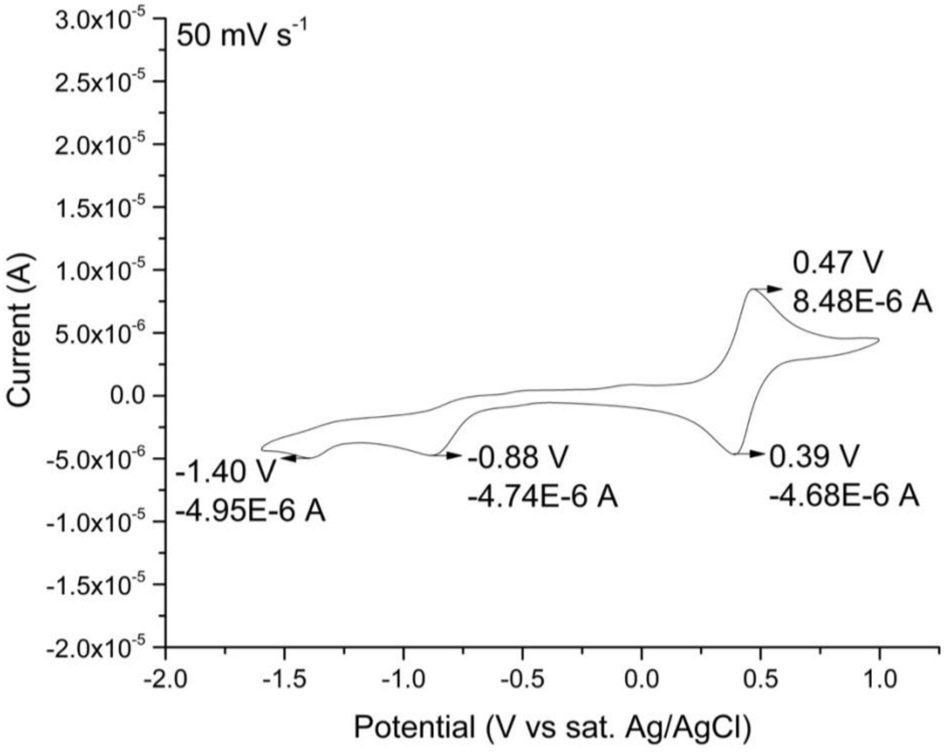

Supplementary Figure 29 Cyclic voltammogram of (BINAP)$PdCl_2$ (6 mM) and ferrocene (6 mM) in 0.2M $TBABF_4$ (DMF) measured at 50 mV s^{-1}

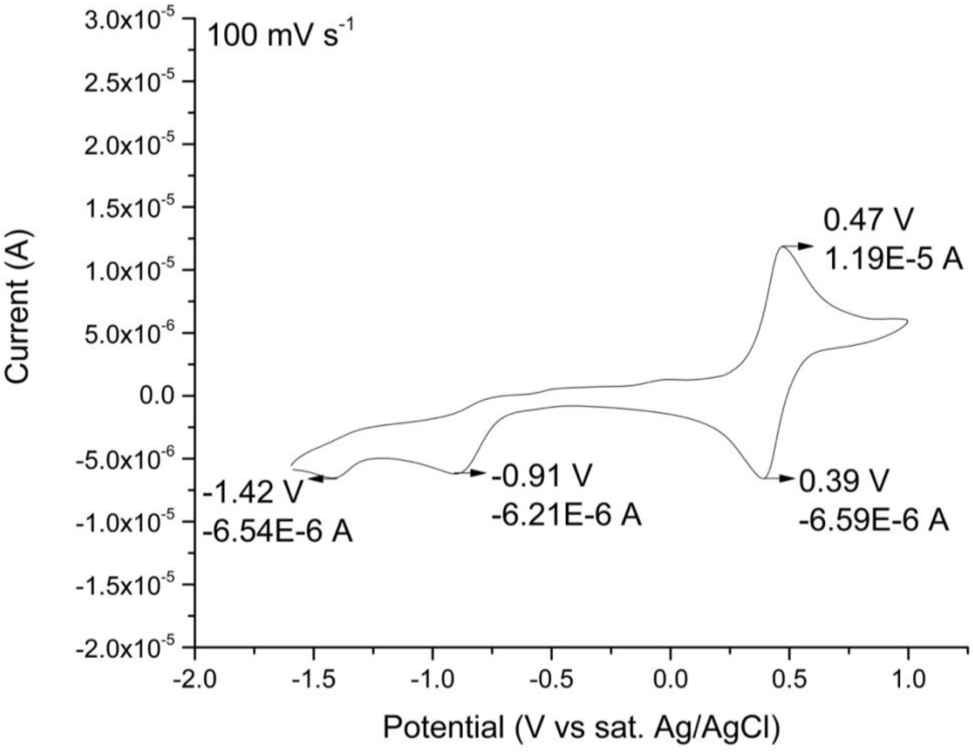

Supplementary Figure 30 Cyclic voltammogram of (BINAP)$PdCl_2$ (6 mM) and ferrocene (6 mM) in 0.2M $TBABF_4$ (DMF) measured at 100 mV s^{-1}

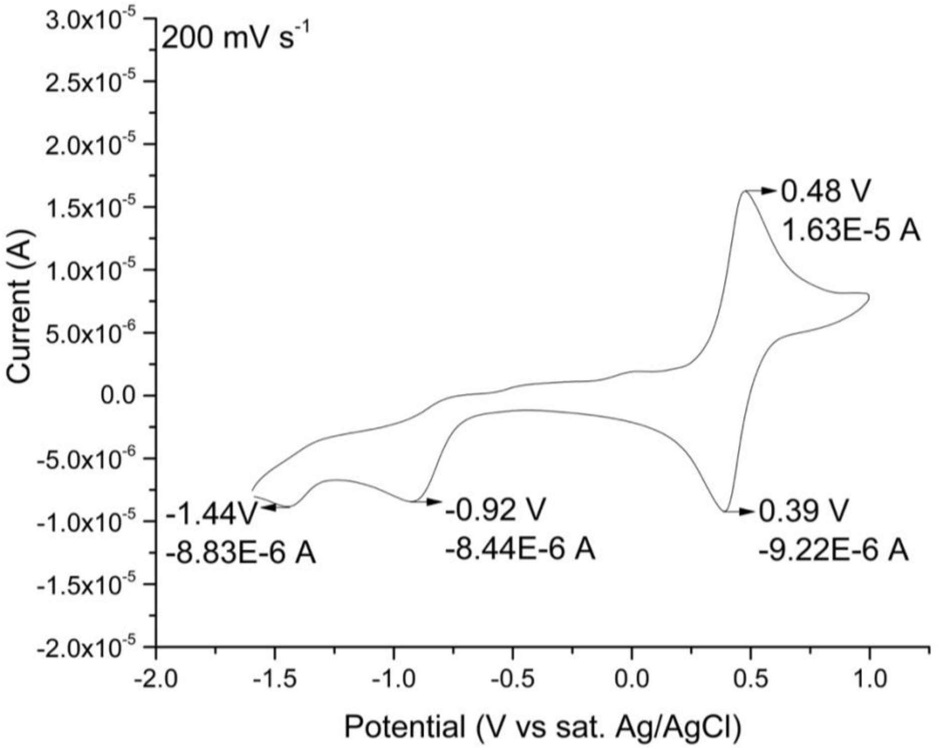

Supplementary Figure 31 Cyclic voltammogram of (BINAP)$PdCl_2$ (6 mM) and ferrocene (6 mM) in 0.2M $TBABF_4$ (DMF) measured at 200 mV s^{-1}

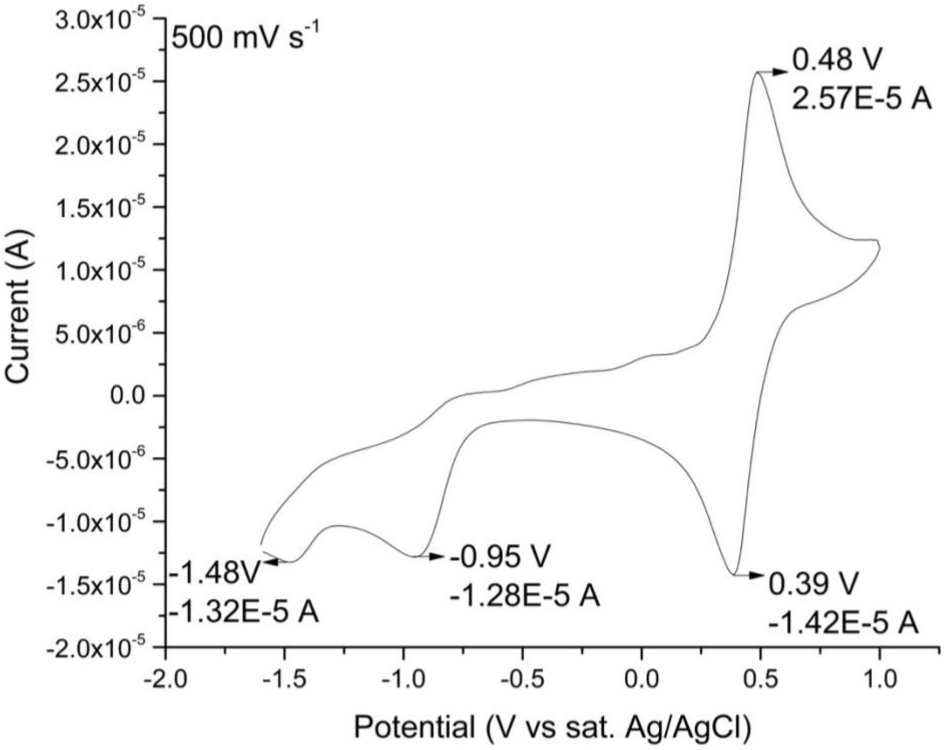

Supplementary Figure 32 Cyclic voltammogram of (BINAP)$PdCl_2$ (6 mM) and ferrocene (6 mM) in 0.2M $TBABF_4$ (DMF) measured at 500 mV s^{-1}

4.3 Electron donor study:

Ethanol oxidation often requires very high potentials or precious metals electrodes, which are not present in our system. Thus, in order to further observe that the ethanol is not being oxidized we performed CV's of separate addition of ethanol or DMF to 0.2 M $TBABF_4$ in acetonitrile (Supplementary Figure 33). In that, we observe that DMF oxidation occurs prior to ethanol oxidation, and that ethanol is probably reacting with the DMF oxidation product which leads to the formation of N-alkoxymethyl-N-methylformamide product.[23]

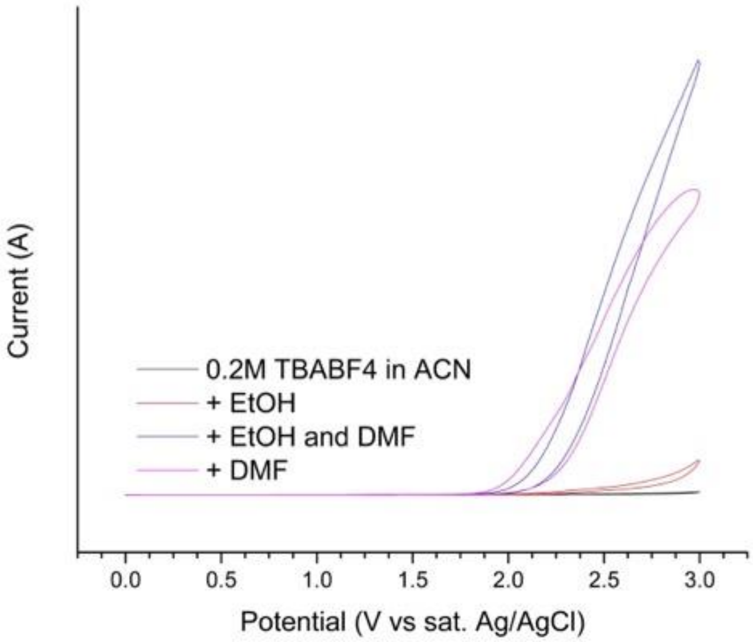

Supplementary Figure 33 Cyclic voltammetry studies for electron donor

4.4 Organometallic Synthesis

4.4.1 Synthesis of (BINAP)Pd(*o*-tolyl)(Br) complex 32

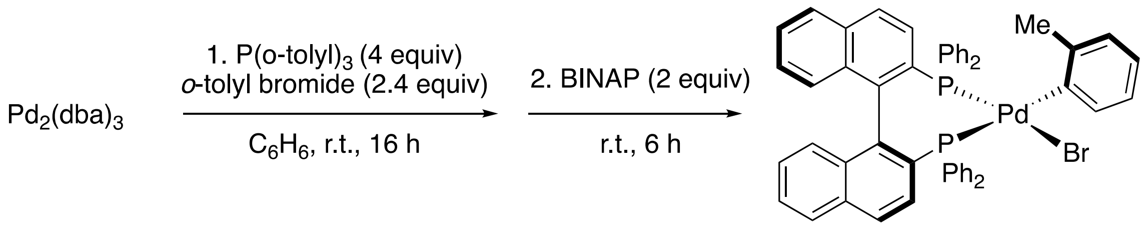

In the glovebox, $Pd(dba)_2$ (207 mg, 0.23 mmol) was added to a 50 mL round bottom flask with $P(o\text{-tolyl})_3$ (276 mg, 0.91 mmol, 3.95 equiv.), *o*-tolyl bromide (94.6 mg, 0.55 mmol, 2.4 equiv.). A stir bar was added, and the flask was charged with 15 mL of C_6H_6 and was let stir overnight. After 16 hours BINAP (281.8 mg, 0.45 mmol, 2.0 equiv.) was added and let stir for 6 hours. The suspension was then filtered through a celite plug and the filtrate concentrated to dryness. The solid was then washed with Et_2O (3 mL x 4) and

pentane (3 mL x 4) to afford a (BINAP)Pd(*o*-tolyl)(Br) (140.5 mg, 35 % yield) as a white solid.

^{1}H NMR (400 MHz, $CDCl_3$) δ 8.16 – 7.97 (m, 1H), 7.94 – 7.46 (m, 10H), 7.46 – 6.07 (m, 25H), 2.86 (s, 2H), 2.57 (s, 1H). **^{31}P NMR** (162 MHz, $CDCl_3$) δ 27.67 (d, J = 38.4 Hz), 24.72 (d, J = 39.0 Hz), 11.14 (d, J = 38.9 Hz), 9.92 (d, J = 38.5 Hz). Poor solubility prevented ^{13}C measurements.

NMR data matches previous reports as a mixture of isomers.
Monteiro, A. L., Davis, W. M., *J. Braz. Chem. Soc.*, **2004**, *15*, 83-95

4.4.2 Synthesis of (BINAP)Pd(phenyl)(Br)

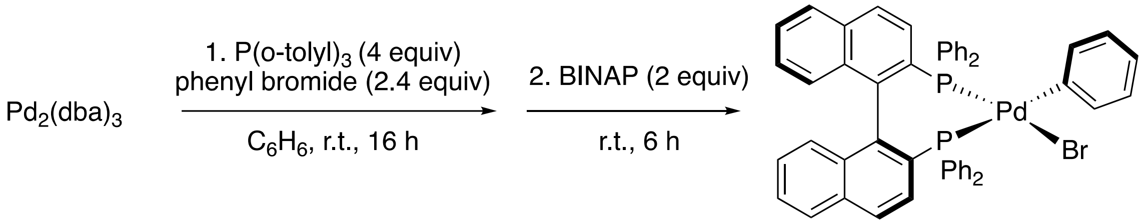

In the glovebox, $Pd(dba)_2$ (207 mg, 0.23 mmol) was added to a 50 mL round bottom flask with $P(o\text{-tolyl})_3$ (276 mg, 0.91 mmol, 3.95 equiv.), phenyl bromide (86.8 mg, 0.55 mmol, 2.4 equiv.). A stir bar was added, and the flask was charged with 15 mL of C_6H_6 and was let stir overnight. After 16 hours BINAP (281.8 mg, 0.45 mmol, 2.0 equiv.) was added and let stir for 6 hours. The suspension was then filtered through a celite plug and the filtrate concentrated to dryness. The solid was then washed with Et_2O (3 mL x 4) and pentane (3 mL x 4) to afford a (BINAP)Pd(*o*-tolyl)(Br) (79.1 mg, 20 % yield) as a white solid.

^{1}H NMR (400 MHz, CDC13) δ 7.86 (dd, J = 23.3, 8.3 Hz, 3H), 7.72 – 7.28 (m, 5H), 7.23 – 6.54 (m, 24H). **^{31}P NMR** (162 MHz, $CDCl_3$) δ 27.68 (d, J = 38.2 Hz), 9.91 (d, J = 38.1 Hz).

NMR data matches previous reports, with residual P(*o*-tolyl)$_3$ present.
Alcazar-Roman, L. M., Hartwig, John F., Rheingold, A. L., *et al. J. Am. Chem. Soc.* **2000**, *122*, 4618–4630

4.5 Mechanistic Experiments

4.5.1 Reaction of (BINAP)Pd(*o*-tolyl)(Br) with CO_2

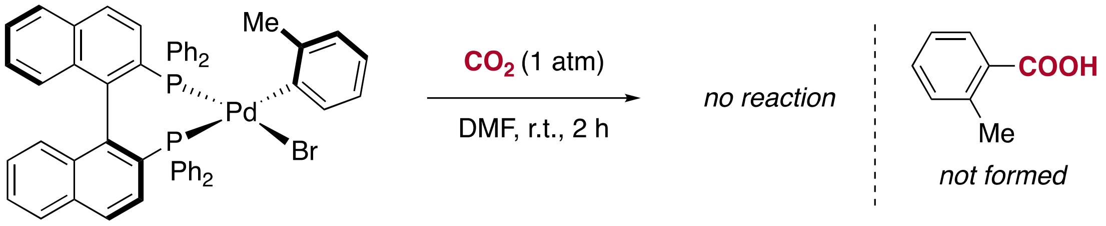

In the glovebox, (BINAP)Pd(*o*-tolyl)(Br) (10.5 mg, 0.012 mmol) was added to a 4 mL dram vial, and dissolved in 1 mL DMF. This solution was transferred to a J-young NMR tube, removed from the glovebox and an initial ^{31}P NMR spectrum was recorded. The J-Young NMR tube was then frozen at -196 °C, headspace removed under vacuum and allowed to thaw under an atmosphere of CO_2 to room temperature. ^{31}P NMR spectra were recorded periodically over 2 hours (Supplementary Figure 34) where no new species besides (BINAP)Pd(*o*-tolyl)(Br) were observed. After 2 hours, the reaction mixture was transferred to a 10 mL vial, and quenched with HCl. A stock solution of trimethoxybenzene (internal standard) was added to this solution, and the organic products were extracted with EtOAc 3 times. The solvent was then removed under vacuum where no formation of *o*-tolyl carboxylic acid was observed by ^{1}H NMR.

The lack of conversion of (BINAP)Pd(*o*-tolyl)(Br) by ^{31}P NMR, along with no formation of the carboxylated organic moiety, *o*-tolyl carboxylic acid upon quenching the reaction is consistent with no carboxylation from (BINAP)Pd(*o*-tolyl)(Br) at room temperature.

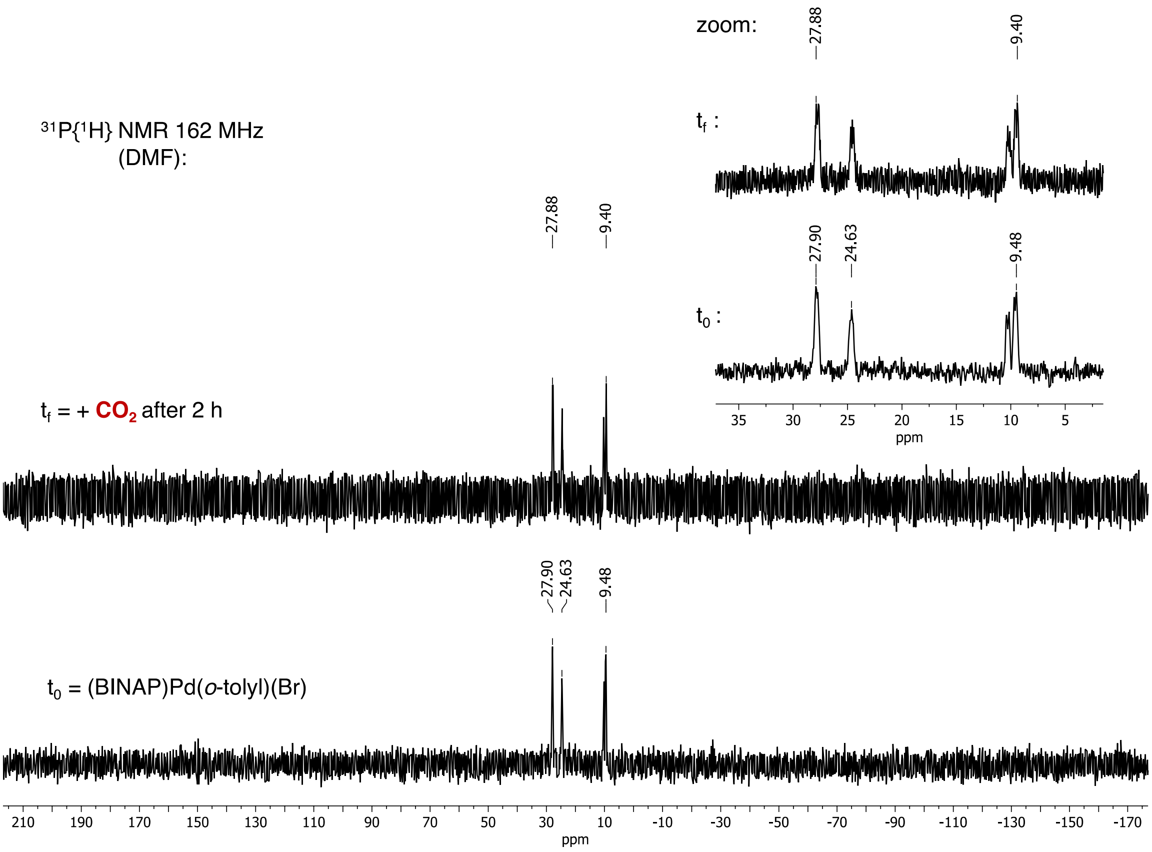

Supplementary Figure 34 Monitoring the reaction between (BINAP)Pd(*o*-tolyl)(Br) and CO_2 over 2 hours.

4.5.2 Cyclic voltammetry of complex 32:

The cyclic voltammetry of complex **32** was performed in an inert atmosphere and in the presence of CO_2 (Supplementary Figure 35). It was possible to observe a significant increase in the peak current with a shift for more reductive potentials in the presence of CO_2.

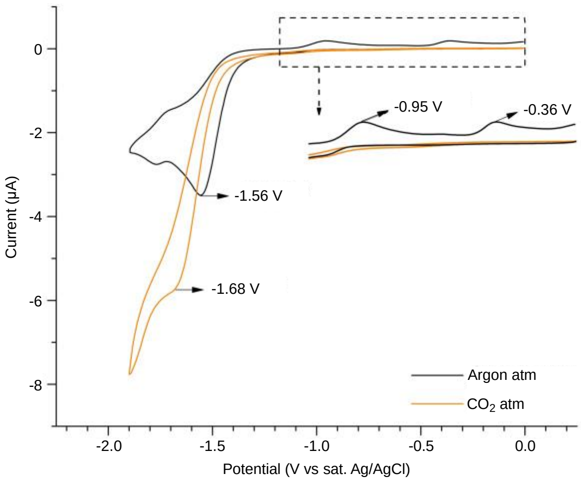

Supplementary Figure 35 Cyclic voltammetry of complex **32** under CO_2 and argon atmosphere.

4.5.3 Monitoring the catalytic reaction under constant potential:

In order to determine more information about the reaction components in catalysis, kinetic measurements were performed at a constant potential (-2 V), where the charge vs time was monitored.

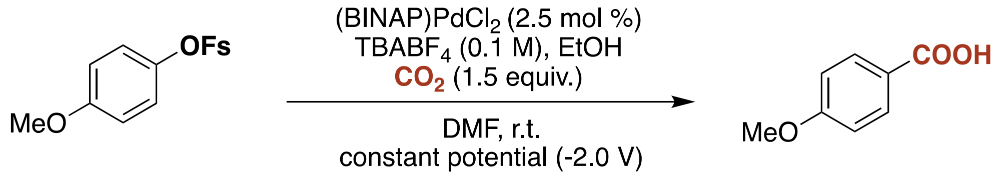

Performing the standard catalytic reaction conditions with 4-methoxyphenyl sulfurofluoridate, the charge over time was monitored in which a linear slope was observed (Supplementary Figure 36) which is consistent with a kinetic profile that occurs by 0 order reaction kinetics.

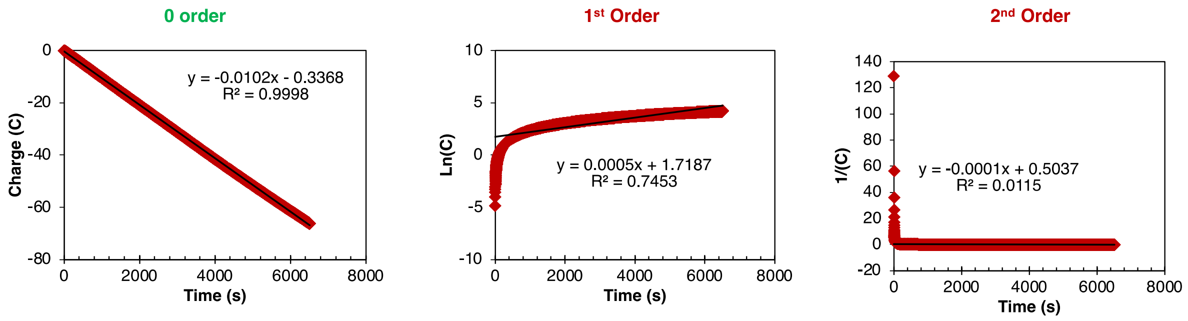

Supplementary Figure 36 Monitoring the standard catalytic reaction. Plots of 0 order, 1st order and 2nd order reactions, in which 0 order reaction kinetics fit the reaction profile.

4.5.4 Changing reaction parameters of standard catalytic reaction under constant potential:

In order to support these 0 order kinetics, the concentration of reaction components in the standard catalytic conditions were changed and the charge vs time at a constant potential (-2 V) was measured. Furthermore, the reactions were stopped at low conversions to compare the rate of conversion.

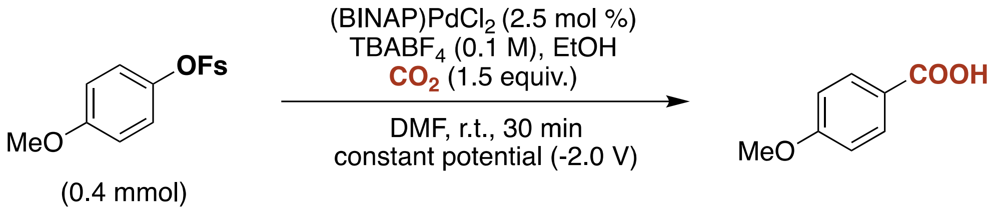

entry	deviation standard conditions	SM (%)	Prod (%)
1	none	70	14
2	(BINAP)PdCl_2 (5 mol %)	70	17
3	CO_2 (3 equiv)	71	15
4	carbon electron (1/2 size, 1 cm x 0.5 cm)	72	13

Supplementary Figure 37 Performing the standard catalytic conditions with modifications to the conditions.

Performing the standard catalytic reaction conditions with 4-methoxyphenyl sulfurofluoridate for 30 minutes with 4 conditions (Supplementary Figure 37 entries 1-4; standard conditions, 2 x Pd precatalyst concentration, 2 x CO_2 concentration, ½ electrode size), the charge over time was monitored in which a linear slope was observed in all cases which is consistent with the 0 order reaction kinetics previously observed. After 30 minutes, the reactions were stopped, TMB was added (67.2 mg, 0.4 mmol) and the reactions were quenched with HCl (1 M). The organic phase was extracted with EtOAc (x 3) and the solvent was then removed under vacuum where the integral ratio of TMB to starting material and carboxylic acid were determined by quantitative ^{1}H NMR. Thus, the same conversions of ca 70 % in each reaction, along with 0 order kinetic profiles of charge vs time support that the standard catalytic reaction proceeds by 0 order kinetics when a constant potential of -2.0 V is applied.

4.6 Further screening of reaction conditions after mechanistic study

With the mechanistic study, it was perceived that the amount of CO_2 does not influence the reaction rate and that ethanol is not an electron donor. Due to this, we turned back to the solvent screening attempted with acetonitrile and ascorbic acid and observed that ascorbic acid has low solubility in the medium. With ethanol as an additive the reaction also does not perform well, as demonstrated in the mechanistic section it is not oxidized in the reaction conditions. We then attempted triethylamine as a proof of concept to demonstrate the feasibility of solvent exchange. The exchange of $TBABF_4$ to TBABr made it feasible to run the reaction with only 1 mol% but reproducibility was an issue, to improve reproducibility the TBABr was used as an additive in $TBABF_4$ and 2 mol% catalyst loading.

4.6.1 General Procedure for the optimization of the amide-free solvent conditions:

On the benchtop, a 2x1 cm piece of carbon paper (Toray carbon paper 090 wet-proofed) was attached to an aluminum holder. To define the available area, the electrode was covered using Teflon® tape to leave an area of 2 cm^2 available (1x1 cm surface area with both sides uncovered), this was pierced through a Teflon® inlay. The cathodic chamber of a two-chamber electro glassware was charged with (BINAP)$PdCl_2$, and electrolyte. The anodic chamber was charged with the reductant (in case it is solid) and electrolyte. The solvent was added first to the anodic chamber followed by the cathodic chamber (2 mL of ACN to the anodic chamber followed by 2 mL in the cathodic chamber), then 2-methoxy-4-propylphenyl sulfurofluoridate (99.3 mg, 0.4 mmol) was weighted directly inside the cathodic chamber. After the extra 1mL of solvent left is used to rinse the walls of the cathodic chamber and the solvent is leveled at the anodic side (total of 3mL of ACN for each chamber), and the reaction flask was closed. The atmosphere of the reaction was exchanged with 3 cycles of vacuum followed by leaving under 1 atm of CO_2 (1 minute each time, in the last cycle the reaction was stirred while under CO_2 for 1 minute) using a needle attached to a Schlenk line. This was followed by attaching the electrodes to the Electroware, where -4 mA current was applied over 85 Coulombs. Once the reaction was complete, it is quenched with 1M HCl and extracted 4 times with EtOAc, the combined organic phases were dried with magnesium sulfate and concentrated under reduced pressure. To this 1,3,5-trimethoxybenzene was added and ^1H crude NMR was recorded.

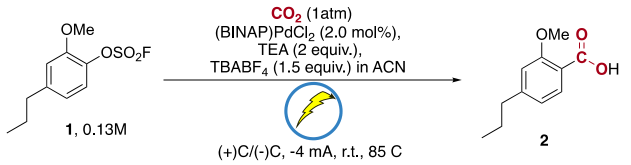

Entry	Change	Yield[a] **2** (%)
**1**	none	91[b]
**2**	$PdCl_2$ + Davephos i.o. (BINAP)$PdCl_2$	7[b]
**3**	EtOH (99%; 4 equiv.) i.o. TEA	57[b]
**4**	Cinnamaldehyde (2 equiv.) i.o. TEA	39[b]
**5**	Vanillin (2 equiv.) i.o. TEA	46[b]
**6**	Ethylene glycol (4 equiv.) i.o. TEA	90[b]
**7**	BINAP$PdCl_2$ (1 mol%)	80[b]
**8**	(BINAP)$PdCl_2$ (1 mol%) and TBABr (0.2M in ACN) at cathode	88[b]
**9**	as in **8** and Ethylene glycol (4 equiv.) i.o. TEA	74
**10**	(BINAP)$PdCl_2$ (0.1 mol%) and TBABr (0.2M in ACN) at cathode	17
**11**	(BINAP)$PdCl_2$ (2 mol%) + TBABr (0.2 equiv.) + $TBABF_4$ (1.3 equiv.) in cathode	84[c]

Supplementary Figure 38 Screening for amide-free conditions

[a] NMR yield calculated with 1,3,5-trimethoxybenzene as internal standard. [b] Reaction was set up inside the glovebox. [c] average of two isolated yields.

5 Synthesis of starting materials

1,1'-Sulfonyldiimidazole (SDI)

A suspension of imidazole (15.82 g, 4.6 equiv., 230 mmol) in 100 mL DCM was prepared in a 250 mL round bottom flask. The flask was cooled down using an ice bath, before a solution of sulfuryl chloride (4.13 mL, 1.0 equiv., 50 mmol) in 25 mL DCM was added to the stirring suspension using a dropping funnel. The ice bath was allowed to warm up to RT and the reaction was stirred overnight. The resulting yellow suspension was filtered through filter paper into a 500 mL round bottom flask and washed with 100 mL of DCM to remove the excess imidazole. The solvent of the filtrate was removed under reduced pressure to yield light-yellow crystals. The product was obtained pure after recrystallizing from boiling isopropanol (75 mL) as colorless crystals (8.52 g, 43 mmol, 86%).

^{1}H-NMR (400 MHz, $CDCl_3$) δ_H (ppm): 8.04 (s, 2H), 7.31 (s, 2H), 7.17 (s, 2H). **^{13}C-NMR (101 MHz, $CDCl_3$) δ_C** (ppm): 136.7, 132.6, 117.5.

The spectral data is consistent with those reported in literature:
Veryser, C., Demaerel, J., Bieliūnas, V., Gilles, P., de Borggraeve, W. M. *Organic Letters*, **2017**, *19*(19), 5244–5247.

5.1 Fluorosulfation reactions:

Fluorosulfation reactions were performed in a two-chamber flask with one chamber for the gas release of sulfuryl fluoride (from SDI and KF in TFA) and the second chamber for the reaction.

In-situ HF generation Safety Concern:

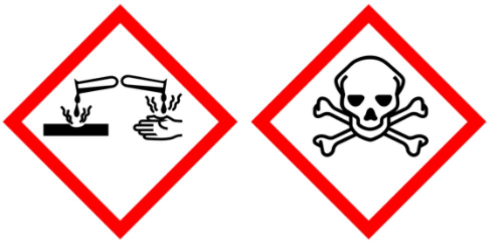

This procedure leads to the formation of HF in the gas release chamber, which represents a high danger if not carefully handled (we suggest careful reading of the SDS for HF before performing the reaction). Furthermore, proper quenching of the gas release reaction must be ensured to avoid further handling after the fluorosulfation reaction is completed.

Additionally, to the described method, Roth and Fuller (J. Org. Chem. 1991, 56, 3493-3496) have demonstrated how fluorosulfonic anhydride can be used for the

fluorosulfation of phenols. Several reports also demonstrate how this reaction can be carried with the direct use of sulfuryl fluoride gas, such as by Sanford (J. Am. Chem. Soc. 2017, 139, 4, 1452–1455), Shen (Org. Lett. 2023, 25, 13, 2318–2322), and Qin (Chem. Asian J. 2017, 12, 2323 –2331).

5.1.1 General Procedure I

The gas release side of a two-chamber flask (210 mL total volume) was charged with SDI (2.97 g, 1.5 equiv., 15 mmol), KF (2.32 g, 4.0 equiv., 40 mmol) and closed with a pierceable Teflon® sealed screw cap. The reaction side was charged with the phenol of interest (1.0 equiv., 10 mmol), K_2CO_3 (2.76 g, 2.0 equiv., 20 mmol) and 40 mL of MeCN before sealing it with a screw cap. After stirring the reaction side for 5 min to create a homogeneous suspension, TFA (20 mL) was added to the gas release side via syringe through the pierceable lid. Within a minute, the gas release could be observed. The reaction was stirred overnight at RT. Before the work-up the screwcaps were opened carefully and the reaction was stirred for 10 min to release excess sulfuryl fluoride. After evaporating the solvent, the pure product was obtained after passing through a silica dry column, rinsing with 250 mL of DCM and removing the solvent *in vacuo*.

5.1.2 General Procedure II

The gas release side of a two-chamber flask (100 mL total volume) was charged with SDI (1.49 g, 1.5 eq, 7.5 mmol) and KF (1.16 g, 4.0 equiv., 20 mmol) and closed with a pierceable Teflon® sealed screw cap. The reaction side was charged with the phenol of interest (1.0 equiv., 5 mmol), Et_3N (1.40 mL, 2.0 equiv., 10 mmol) and 20 mL of DCM before sealing it with a screw cap. TFA (5 mL) was added to the gas release side via syringe through the pierceable lid. Within a minute the gas release could be observed. The reaction was stirred overnight at RT. After completion, the screwcaps were opened carefully and the reaction was stirred for 10 min to release excess sulfuryl fluoride. After evaporating the solvent, the product was purified via flash column chromatography.

5.1.3 General Procedure III

General procedure C is identical to general procedure B with the exception that DIPEA (2.62 mL, 3.0 equiv., 15 mmol) was used instead of Et_3N, and the solvent in the reaction chamber was exchanged for MeCN.

Caution remarks

1) Glassware under pressure should always be examined for damages or scratches, the COware should under no circumstances be operated above (5 bar).

2) After the reaction, chamber A was quenched with sodium hydroxide solution to neutralize the leftover TFA and the *in situ* formed HF. The etching of the glassware was seen after multiple experiments.

2-methoxy-4-propylphenyl sulfurofluoridate

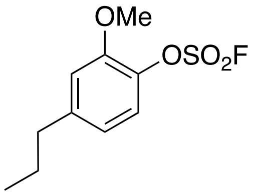

The title compound was prepared according to general procedure I from 2-methoxy-4-propyl phenol (1.66 g, 10.0 mmol) to yield the title compound as a colorless oil (2.40 g, 9.70 mmol, 97%).

^{1}H-NMR (400 MHz, CDCl$_3$) δ$_H$ (ppm): 7.21 (d, *J* = 8.3 Hz, 1H), 6.89 (s, 1H), 6.80 (d, *J* = 8.4 Hz, 1H), 3.89 (s, 3H), 2.61 (t, *J* = 7.7 Hz, 2H), 1.86 – 1.55 (m, 2H), 0.98 (t, *J* = 7.3 Hz, 3H). **^{13}C-NMR (101 MHz, CDCl$_3$) δ$_C$** (ppm)**:** 150.9, 145.1, 137.1, 121.9, 120.7, 113.6, 56.0, 38.0, 24.5, 13.7. **^{19}F-NMR (376 MHz, CDCl$_3$) δ$_F$** (ppm)**:** 39.1.

The spectral data is consistent with those reported in literature:
Pedersen, S. S., *et al. JACS Au,* **2023**, *4*, 1221–1229

[1,1'-biphenyl]-4-yl sulfurofluoridate

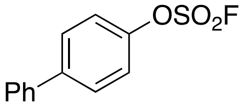

The title compound was prepared according to general procedure I from 4-phenylphenol (397 mg, 2.34 mmol) to yield the title compound as a colorless solid (492 mg, 1.95 mmol, 84%).
^{1}H-NMR (400 MHz, CDCl$_3$) δ$_H$ (ppm): 7.67 (d, *J* = 8.9 Hz, 2H), 7.56 (d, *J* = 7.0 Hz, 2H), 7.47 (t, *J* = 7.3 Hz, 2H), 7.44 – 7.38 (m, 3H). **^{13}C-NMR (101 MHz, CDCl$_3$) δ$_C$** (ppm)**:** 149.5, 142.2, 139.4, 129.2 (d, *J* = 1.8 Hz), 128.3, 127.4, 121.3. **^{19}F-NMR (376 MHz, CDCl$_3$) δ$_F$** (ppm): 37.6.

The spectral data is consistent with those reported in literature:
K. Domino, *et al. Angew. Chem. Int. Ed.* **2018**, *57*, 6858.

phenyl sulfurofluoridate

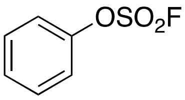

The title compound was prepared according to general procedure II from phenol (471 mg, 5.00 mmol). Flash column chromatography (2% EtOAc in pentane) yielded the title compound as a colorless oil (625 mg, 3.55 mmol, 71%).

^{1}H-NMR (400 MHz, $CDCl_3$) δ_H (ppm): 7.49 (t, *J* = 7.5 Hz, 2H), 7.42 (t, *J* = 7.3 Hz, 1H), 7.35 (d, *J* = 8.4 Hz, 2H). **^{13}C-NMR (101 MHz, $CDCl_3$) δ_C** (ppm): 150.2, 130.5, 128.8, 121.0 (d, *J* = 1.5 Hz). **^{19}F-NMR (376 MHz, $CDCl_3$) δ_F** (ppm): 37.5.

The spectral data is consistent with those reported in literature:
Wang, X., *et al. Chem. Comm.*, **2021**, *57*(66), 8170–8173.

2-isopropylphenyl sulfurofluoridate

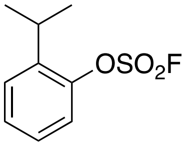

The title compound was prepared according to general procedure II from 2-isopropylphenol (681 mg, 5.00 mmol). Flash column chromatography (2 % EtOAc in pentane) yielded the title compound as a colorless oil (898 mg, 4.10 mmol, 82%).

^{1}H-NMR (400 MHz, $CDCl_3$) δ_H (ppm): 7.41 (dd, *J* = 7.8, 1.9 Hz, 1H), 7.38 – 7.33 (m, 1H), 7.33 – 7.29 (m, 1H), 7.29 – 7.23 (m, 1H), 3.31 (hept, *J* = 6.8 Hz, 1H), 1.26 (d, *J* = 6.8 Hz, 6H).**^{13}C-NMR (101 MHz, $CDCl_3$) δ_C** (ppm): 148.1, 140.8, 129.0, 128.1, 127.6, 120.8 (d, *J* = 1.4 Hz), 27.1, 23.2. **^{19}F-NMR (376 MHz, $CDCl_3$) δ_F** (ppm): 39.4.

The spectral data is consistent with those reported in literature:
Zheng, Q., *et al. J. Am. Chem. Soc.*, **2021**, *143*(10), 3753–3763.

propane-2,2-diylbis(4,1-phenylene) bis(sulfurofluoridate)

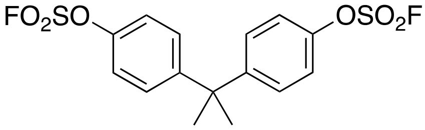

The title compound was prepared according to general procedure II from 4,4'-(propane-2,2-diyl)diphenol (1.14 g, 5.00 mmol). Flash column chromatography (5% EtOAc in pentane) yielded the title compound as a colorless solid (1.80 g, 4.60 mmol, 92%).

^{1}H-NMR (400 MHz, $CDCl_3$) δ_H (ppm): 7.33 – 7.23 (m, 8H), 1.70 (s, 6H). **^{13}C-NMR (101 MHz, $CDCl_3$) δ_C** (ppm): 150.6, 148.4, 128.9, 120.7, 43.1, 30.9. **^{19}F-NMR (376 MHz, $CDCl_3$) δ_F** (ppm): 37.5.

The spectral data is consistent with those reported in literature:
Gilles, P., *et al. J. Org. Chem.* **2019**, *84*(2), 1070–1078.

4-allyl-2-methoxyphenyl sulfurofluoridate

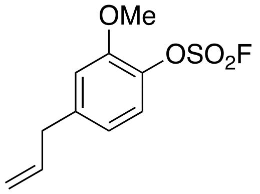

The title compound was prepared according to general procedure II from 4-allyl-2-methoxyphenol (821 mg, 5.00 mmol). Passing a silica dry column with DCM yielded the title compound as a colorless oil (960 mg, 3.90 mmol, 78%).

^{1}H-NMR (400 MHz, $CDCl_3$) δ_H (ppm): 7.23 (d, *J* = 8.3 Hz, 1H), 6.86 (s, 1H), 6.81 (d, *J* = 8.3 Hz, 1H), 6.01 – 5.87 (m, 1H), 5.17 – 5.08 (m, 2H), 3.90 (s, 3H), 3.40 (d, *J* = 6.7 Hz, 2H). **^{13}C-NMR (101 MHz, $CDCl_3$) δ_C** (ppm): 151.1, 142.3, 137.5, 136.4, 122.2, 120.9, 117.0, 113.7, 56.2, 40.2. **^{19}F-NMR (376 MHz, $CDCl_3$) δ_F** (ppm): 39.4.

The spectral data is consistent with those reported in literature:
Gilles, P., *et al. J. Org. Chem.* **2019**, *84*(2), 1070–1078.

4-methoxyphenyl sulfurofluoridate

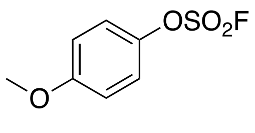

The title compound was prepared according to general procedure II from 4-methoxyphenol (1.24 g, 10.0 mmol). Passing a silica dry column with DCM yielded the title compound as a colorless oil (2.00 g, 9.70 mmol, 97%).

^{1}H-NMR (400 MHz, $CDCl_3$) δ_H (ppm): 7.26 (d, *J* = 9.2 Hz, 2H), 6.94 (d, *J* = 9.3 Hz, 2H), 3.83 (s, 3H). **^{13}C-NMR (101 MHz, $CDCl_3$) δ_C** (ppm): 159.4, 143.7, 122.2 (d, *J* = 1.1 Hz), 115.3, 55.9. **^{19}F-NMR (376 MHz, $CDCl_3$) δ_F** (ppm): 36.4.

The spectral data is consistent with those reported in literature:
Liang, Q., *et al. Org. Lett.* **2015**, *17*(8), 1942–1945.

4-cyanophenyl sulfurofluoridate

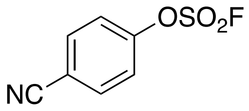

The title compound was prepared according to general procedure II from 4-cyanophenol (596 mg, 5.00 mmol). Flash column chromatography (10-30% EtOAc in heptane) yielded the title compound as a colorless solid (807 mg, 4.00 mmol, 80%).

^{1}H-NMR (400 MHz, $CDCl_3$) δ_H (ppm): 7.82 (d, *J* = 8.9 Hz, 2H), 7.50 (d, *J* = 8.4 Hz, 2H). **^{13}C-NMR (101 MHz, $CDCl_3$) δ_C** (ppm): 152.4, 134.8, 122.3 (d, *J* = 1.5 Hz), 117.2, 113.4. **^{19}F-NMR (376 MHz, $CDCl_3$) δ_F** (ppm): 39.4.

The spectral data is consistent with those reported in literature:
Liang, Q., *et al. Org. Lett.* **2015**, *17*(8), 1942–1945.

benzo[*d*][1,3]dioxol-5-yl sulfurofluoridate

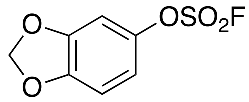

The title compound was prepared according to general procedure I from benzo[d][1,3]dioxol-5-ol (1381 mg, 10.0 mmol). Dry column chromatography (5% EtOAc in pentane) yielded the title compound as a colorless oil (2148 mg, 9.75 mmol, 98%).

^{1}H-NMR (400 MHz, $CDCl_3$) δ_H (ppm): 6.85 – 6.79 (m, 3H), 6.06 (s, 2H). **^{13}C-NMR (101 MHz, $CDCl_3$) δ_C** (ppm): 148.8, 147.8, 144.2, 114.2, 108.4, 103.1, 102.7. **^{19}F-NMR (376 MHz, $CDCl_3$) δ_F** (ppm): 36.6

The spectral data is consistent with those reported in literature:
Veryser, C., *et. al. Org. Lett.* **2017**, *19*(19), 5244–5247.

4-(methylthio)phenyl sulfurofluoridate

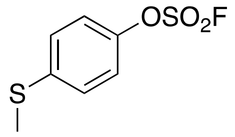

The title compound was prepared according to general procedure I from 4-(methylthio)phenol (1402 mg, 10.0 mmol). Flash column chromatography (1 to 10% EtOAc in heptane) yielded the title compound as a colorless oil (2027 mg, 9.1 mmol, 91%).

^{1}H-NMR (400 MHz, $CDCl_3$) δ_H (ppm): 7.30 (d, *J* = 9.2 Hz, 2H), 7.28 – 7.23 (m, 2H), 2.50 (s, 3H). **^{13}C-NMR (101 MHz, $CDCl_3$) δ_C** (ppm): 147.5, 140.2, 127.7, 121.4, 15.9. **^{19}F-NMR (376 MHz, $CDCl_3$) δ_F** (ppm): 37.3

The spectral data is consistent with those reported in literature:

Johansen, M. B., *et al. Org. Lett.* **2020**, *22*, 4068–4072

naphthalen-2-yl sulfurofluoridate

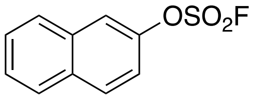

The title compound was prepared according to general procedure I from naphthalen-2-ol (721 mg, 5.0 mmol). Flash column chromatography (1 to 10% EtOAc in heptane) yielded the title compound as a colorless oil (922.7 mg, 4.1 mmol, 82%).

^{1}H-NMR (400 MHz, $CDCl_3$) δ_H (ppm): 7.96 (d, *J* = 9.0 Hz, 1H), 7.94 – 7.87 (m, 2H), 7.82 (d, *J* = 2.7 Hz, 1H), 7.63 – 7.56 (m, 2H), 7.48 – 7.41 (m, 1H). **^{13}C-NMR (101 MHz, $CDCl_3$) δ_C** (ppm): 147.7, 133.5, 132.6, 131.0, 128.2, 128.1, 127.8, 127.5, 119.2, 119.0. **^{19}F-NMR (376 MHz, $CDCl_3$) δ_F** (ppm): 37.7.

The spectral data is consistent with those reported in literature:
Veryser, C., *et. al. Org. Lett.* **2017**, *19*(19), 5244–5247.

2-oxo-1,2,3,4-tetrahydroquinolin-7-yl sulfurofluoridate

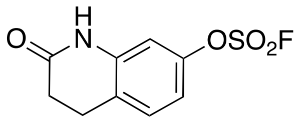

The title compound was prepared according to general procedure I from 7-hydroxy-3,4-dihydroquinolin-2(1H)-one (816 mg, 5.0 mmol). Flash column chromatography (diethyl ether) yielded the title compound (761.2 mg, 3.1 mmol, 62%).

^{1}H-NMR (400 MHz, $CDCl_3$) δ_H (ppm): 8.98 (s, 1H), 7.24 (s, 1H), 7.00 – 6.94 (m, 1H), 6.82 (d, J = 2.6 Hz, 1H), 3.01 (t, J = 7.6 Hz, 2H), 2.68 (t, J = 7.9 Hz, 2H). **^{13}C-NMR (101 MHz, $CDCl_3$) δ_C** (ppm): 171.7, 149.2, 139.2, 129.7, 124.4, 115.0, 108.3, 30.3, 25.0. **^{19}F-NMR (376 MHz, $CDCl_3$) δ_F** (ppm): -37.8. **HRMS** (ESI+) $C_9H_9FNO_4S$ $[M+H]^+$; calculated 246.0231, found 246.0238.

4-((3-chloro-4-fluorophenyl)amino)-7-methoxyquinazolin-6-yl sulfurofluoridate

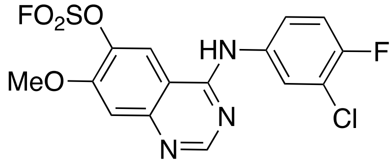

The title compound was prepared according to general procedure III from 4-((3-Chloro-4-fluorophenyl)amino)-7-methoxyquinazolin-6-ol (1.00 g, 3.13 mmol). The reaction was stirred for 72 h at RT. Flash column chromatography (10-40% EtOAc in heptane) yielded the title compound as an off-white solid (1.17 g, 2.91 mmol, 93%).

^{1}H-NMR (400 MHz, $CDCl_3$) δ_H (ppm): 8.75 (s, 1H), 7.91 (dd, *J* = 6.5, 2.7 Hz, 1H), 7.81 (s, 1H), 7.50 (ddd, *J* = 8.9, 4.0, 2.7 Hz, 1H), 7.43 (s, 1H), 7.20 (t, *J* = 8.8 Hz, 2H), 4.07 (s, 3H).**^{13}C-NMR (101 MHz, $CDCl_3$) δ_C** (ppm): 157.4, 156.7, 156.5, 155.3, 154.3, 151.6, 138.6, 134.3, 122.3 (d, J = 7.0 Hz), 121.5 (d, J = 18.7 Hz), 117.0 (d, J = 22.4 Hz), 114.8, 110.6, 108.3, 56.9. **^{19}F-NMR (376 MHz, $CDCl_3$) δ_F** (ppm): 40.5, -119.2. **HRMS** (ESI-) $C_{15}H_9ClF_2N_3O_4S$ $[M-H]^-$; calculated 399.9976, found 399.9971.

(13S)-17-hydroxy-13-methyl-7,8,9,11,12,13,14,15,16,17-decahydro-6H-cyclopenta[a]phenanthren-3-yl sulfurofluoridate

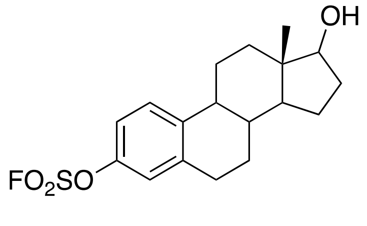

The title compound was prepared according to general procedure II from estradiol (500 mg, 1.75 mmol). Flash column chromatography (10-40% EtOAc in heptane) yielded the title compound as a colorless solid (546 mg, 1.54 mmol, 88%).

^{1}H-NMR (400 MHz, CDCl$_3$) δ_H (ppm): 7.36 (d, *J* = 8.8 Hz, 1H), 7.08 (d, *J* = 8.7 Hz, 1H), 7.04 (s, 1H), 3.74 (t, *J* = 8.5 Hz, 1H), 2.98 – 2.70 (m, 2H), 2.37 – 2.29 (m, 1H), 2.28 – 2.19 (m, 1H), 2.19 – 2.07 (m, 1H), 2.02 – 1.85 (m, 2H), 1.79 – 1.67 (m, 1H), 1.64 – 1.08 (m, 8H), 0.79 (s, 3H). **^{13}C-NMR (101 MHz, CDCl$_3$) δ_C** (ppm): 148.1, 141.3, 139.8, 127.4, 120.8, 117.8, 81.9, 50.1, 44.2, 43.3, 38.3, 36.7, 30.7, 29.7, 26.9, 26.2, 23.2, 11.1. **^{19}F-NMR (376 MHz, CDCl$_3$) δ_F** (ppm): 37.3.

The spectral data is consistent with those reported in literature:
Veryser, C., *et. al. Org. Lett.* **2017**, *19*(19), 5244–5247.

2,5,7,8-tetramethyl-2-(4,8,12-trimethyltridecyl)chroman-6-yl sulfurofluoridate

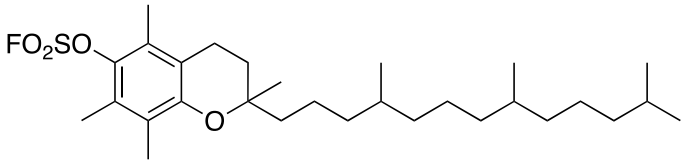

The title compound was prepared according to general procedure I from 2,5,7,8-tetramethyl-2-(4,8,12-trimethyltridecyl)chroman-6-ol (2154 mg, 5.0 mmol). Flash column chromatography (pure pentane) yielded the title compound as a colorless oil (1577 mg, 3.1 mmol, 62%).

^{1}H-NMR (400 MHz, CDCl$_3$) δ_H (ppm): 2.60 (t, J = 6.8 Hz, 2H), 2.23 (s, 3H), 2.20 (s, 3H), 2.10 (s, 3H), 1.89 – 1.73 (m, 2H), 1.62 – 1.02 (m, 24H), 0.89 – 0.82 (m, 12H). **^{13}C-NMR (101 MHz, CDCl$_3$)** (mixture of diastereomers) **δ_C** (ppm): 151.1, 142.0, 127.6, 126.2, 124.5, 118.5, 75.9, 40.1, 40.1, 39.5, 37.6, 37.6, 37.6, 37.5, 37.5, 37.5, 37.4, 32.9, 32.9, 32.8, 32.8, 31.0, 30.9, 28.1, 25.0, 25.0, 24.6, 24.0, 22.9, 22.8, 21.1, 20.8, 19.9, 19.8, 19.8, 19.7, 13.7, 13.7, 12.9, 12.8, 12.1. **^{19}F-NMR (376 MHz, CDCl$_3$) δ_F** (ppm): 41.4.

The spectral data is consistent with those reported in literature:
Veryser, C., *et. al. Org. Lett.* **2017**, *19*(19), 5244–5247.

Adapalane starting material (6-(3-((3r,5r,7r)-adamantan-1-yl)-4-methoxyphenyl)naphthalen-2-yl sulfurofluoridate):

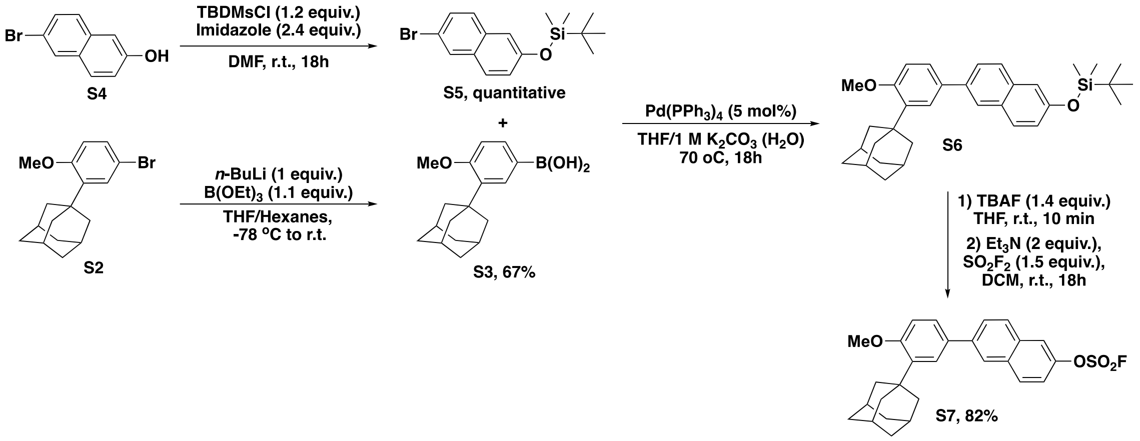

To a solution of **S2** (5.3 mmol) in THF (18 mL) at -78 °C under argon, *n*-BuLi (1.9 M, 2.6 mL, 5.3 mmol) was added dropwise. The mixture was allowed to stir for 5 minutes at -78 °C. Then triethyl borate (5.83 mmol) was added and stirred for 30 minutes, let the reaction warm to room temperature for 1 hour, and quenched with 1 M HCl. The reaction content was diluted with diethyl ether, washed with water and brine, dried over sodium sulfate, and concentrated under a vacuum. The desired product **S3** (1.02g, 67%) was obtained after flash chromatography (10 % EtOAc in heptane to pure heptane).

In a round bottom flask **S4** (2.7g, 12.3 mmol, 1 equiv.), TBDMsCl (2.2g, 14.7 mmol, 1.2 equiv.) and imidazole (2.0g, 30.0 mmol, 2.4 equiv.) were added, and diluted in 10 mL of DMF and allowed to stir overnight at room temperature. After, the reaction was quenched with sodium bicarbonate solution (1M, 150 mL) and washed with EtOAc/Heptane (1:1) 3 times (50 mL). The combined organic phases were washed with sodium bicarbonate solution (1M, 50 mL) 4 times, dried over sodium sulfate, and concentrated to provide **S5** (4.1g, 12.2 mmol, quant.).

Inside the glovebox **S3** (1020.0 mg, 3.55 mmol, 1.2 equiv.), **S5** (998.5 mg, 2.96 mmol, 1 equiv.), and $Pd(PPh_3)_4$ (133 mg, 0.12 mmol, 5 mol%) were added to a pressure tube, dissolved in 9 mL of THF and 7.5 mL of potassium carbonate solution (1M). The tube was closed and taken outside the glovebox, where it was allowed to stir at 70 °C overnight. The reaction was cooled to room temperature and the layers separated, the aqueous layer was further extracted 3 times with diethyl ether, the organic phases combined dried over sodium sulfate and concentrated under reduced pressure. The crude of this reaction was dissolved in 15 mL of THF and TBAF solution (1M in THF, 4 mL, 4 mmol) was added and allowed to stir at room temperature for 10 minutes. The reaction was then diluted in EtOAc and washed with 1M HCl (2 times), water (2 times), and brine. The organic phase was then dried over sodium sulfate and concentrated under vacuum. In a two-chamber

set-up the crude of this reaction was added and dissolved in 12 mL of DCM followed by the addition of 840 µL of triethylamine and closure of the chamber (it was assumed 3 mmol of starting material). In the release side, SDI (892 mg, 4.6 mmol, 1.5 equiv.), and KF (697 mg, 12 mmol, 4 equiv.) were added and the whole system was closed before 5 mL of TFA was added via syringe in the release side. The reaction was allowed to stir at room temperature overnight, and then the contents of the reaction were transferred to a round bottom flask to be concentrated. The desired product **S7** was obtained as a white solid (1.13g, 2.4 mmol, 82%) after flash column chromatography (heptane to 20% EtOAc in heptane).

^{1}H-NMR (500 MHz, $CDCl_3$) δ_H (ppm): 1.81 (t, J = 3.1 Hz, 6H), 2.11 (t, J = 3.1 Hz, 3H), 2.19 (d, J = 2.9 Hz, 6H), 3.91 (s, 3H), 7.01 (d, J = 8.4 Hz, 1H), 7.44 (dd, J = 9.0, 2.5 Hz, 1H), 7.53 (dd, J = 8.4, 2.3 Hz, 1H), 7.59 (d, J = 2.4 Hz, 1H), 7.82 (d, J = 2.5 Hz, 1H), 7.85 (dd, J = 8.5, 1.8 Hz, 1H), 7.92 (d, J = 8.5 Hz, 1H), 7.99 (d, J = 9.0 Hz, 1H), 8.03 (d, J = 1.7 Hz, 1H). **^{13}C-NMR (126 MHz, $CDCl_3$) δ_C** (ppm): 159.1, 147.4, 140.7, 139.2, 133.1, 132.4, 132.1, 131.0, 128.5, 127.7, 126.1, 125.8, 125.0, 119.4, 118.8, 112.3, 55.3, 40.8, 37.4, 37.3, 29.3. **^{19}F-NMR (473 MHz, $CDCl_3$) δ_F** (ppm): 37.7. **HRMS** (ESI+) $C_{27}H_{28}FO_4S$ $[M+H]^+$; calculated 467.1687, found 467.1678.

Bexarotene starting material (4-(1-(3,5,5,8,8-pentamethyl-5,6,7,8-tetrahydronaphthalen-2-yl)vinyl)phenyl sulfurofluoridate):

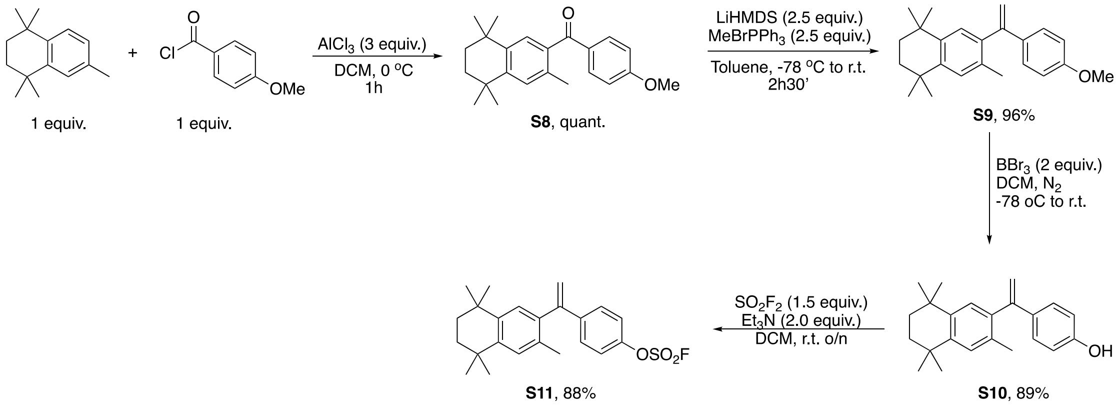

In a flame-dried round bottom flask 1,1,4,4,6-pentamethyl-1,2,3,4-tetrahydronaphthalene (2.0 g, 9.9 mmol, 1 equiv.), 4-methoxybenzoyl chloride (1.7g, 9.9 mmol, 1 equiv.) were added and dissolved in 20 mL of dry DCM. The solution was cooled to 0 °C and $AlCl_3$ (3.9g, 29.7 mmol, 3 equiv.) was portion-wise added over 10 minutes. The reaction was allowed to stir at 0 °C for 1h, quenched with ice and washed with water. The aqueous phase was further extracted 3 times with DCM, the organic phases were combined and dried over sodium sulfate. The desired product **S8** was obtained as a colorless oil (3.3g, 9.9 mmol, quantitative) after flash column chromatography (heptane to 10% EtOAc in heptane).

A solution of LiHMDS (2.0 g, 11.9 mmol, 2.5 equiv. in 12 mL of toluene) was prepared in a flame-dried round bottom flask. In a second round bottom flask, a slurry of $MeBrPPh_3$ (4.3g, 11.9 mmol, 2.5 equiv. in 47 mL of toluene) was prepared and cooled to 0 ^oC. A third solution was prepared dissolving **S8** (1.6g, 4.7 mmol, 1 equiv.) in 47 mL of toluene. Under an inert atmosphere (argon) the LiHMDS solution was slowly added (over 5 minutes) to the $MeBrPPh_3$ solution (with constant cooling) and stirred for 15 minutes after addition. At this time the slurry was cooled to -78 ^oC and the **S8** solution was added over 10 minutes, then the reaction was allowed to stir at -78 ^oC for 1h, and allowed to warm up to room temperature over a period of 90 minutes (while constant stirring). The reaction was quenched with ice and washed with water, the organic phase was dried over sodium sulfate and concentrated under vacuum. The desired product **S9** was obtained as a white solid (1.5g, 4.5 mmol, 96%) after flash column chromatography (heptane to 5% EtOAc in heptane).

To a flame-dried round bottom flask **S9** (1.46g, 4.4 mmol, 1 equiv.) was added and dissolved in 40 mL of dry DCM, the atmosphere was exchanged to argon and cooled to -78 ^oC. To this, BBr_3 (830 µL, 8.7 mmol, 2 equiv.) was slowly added over 10 minutes, the reaction was allowed to stir at -78 ^oC for 10 minutes and then allowed to warm up to room temperature over the period of 1h (while stirring). The reaction was quenched with ice and washed with water, the aqueous phase was further extracted with DCM (4 times), the organic phases combined and dried over sodium sulfate, and concentrated under vacuum. The desired product **S10** was obtained as a white solid (1.24g, 3.9 mmol, 89%) after flash column chromatography (heptane to 30% EtOAc in heptane).

The desired final product **S11** was obtained with general procedure II with the use of **S10** (1.23g, 3.8 mmol, 1 equiv.), triethylamine (1064 µL) in 15 mL of DCM. Gas release was obtained with SDI (1.13g), and KF (882.9 mg) with 5 mL of TFA. The final product **S11** was obtained as a white solid (1.35g, 3.4 mmol, 88%) after flash column chromatography (heptane to 1% EtOAc).

^{1}H-NMR (400 MHz, $CDCl_3$) δ_H (ppm): 7.40 – 7.35 (m, 2H), 7.27 – 7.23 (m, 2H), 7.10 (d, J = 11.0 Hz, 2H), 5.74 (d, J = 1.2 Hz, 1H), 5.30 (d, J = 1.2 Hz, 1H), 1.96 (d, J = 0.6 Hz, 3H), 1.70 (s, 4H), 1.30 (s, 6H), 1.28 (s, 6H). **^{13}C-NMR (101 MHz, $CDCl_3$) δ_C** (ppm): 149.4, 148.4, 144.7, 142.6, 141.9, 137.9, 132.8, 128.6, 128.3, 128.1, 120.8, 116.7, 35.3, 35.3, 34.2, 34.0, 32.1, 32.0, 20.1.**^{19}F-NMR (376 MHz, $CDCl_3$) δ_F** (ppm): -37.6. **HRMS** (ESI+) $C_{23}H_{28}FO_3S$ $[M+H]^+$; calculated 403.1738, found 403.1739.

5.2 Synthesis of aryl bromides

Tamibarotene starting material (4-bromo-N-(5,5,8,8-tetramethyl-5,6,7,8-tetrahydronaphthalen-2-yl)benzamide):

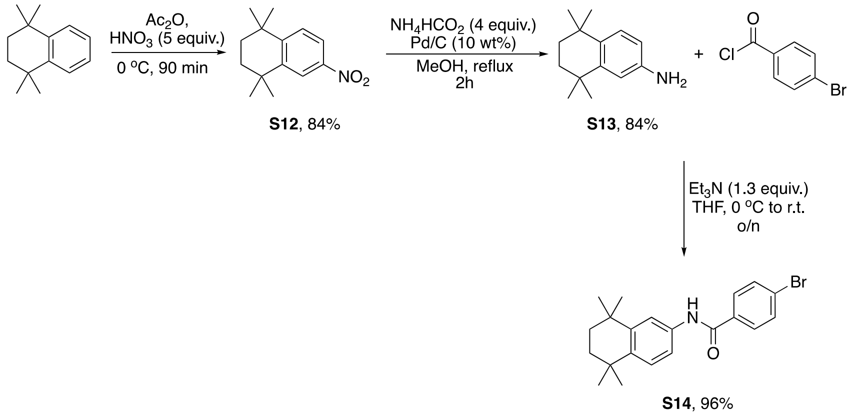

To a round bottom flask, 1,1,4,4-tetramethyl-1,2,3,4-tetrahydronaphthalene (2.0 g, 10.6 mmol, 1 equiv.) was added and dissolved in 10 mL of acetic anhydride and cooled to 0 °C. A solution of HNO_3 (3.6 mL, 53.1 mmol, 5 equiv.) in 15 mL of acetic anhydride was slowly added over 15 minutes and let stir for 90 minutes. The reaction was poured in ice and extracted with diethyl ether (3 times), the organic phases were combined, washed with concentrated sodium bicarbonate solution, dried over sodium sulfate, and concentrated. The desired product **S12** was obtained as a yellowish solid (2.1 g, 8.9 mmol, 84%) after flash column chromatography (heptane to 4% EtOAc in heptane).

In a round bottom flask, **S12** (1.06 g, 4.6 mmol, 1 equiv.) was added and dissolved in 20 mL of MeOH. Palladium on carbon (54 mg, 5 wt%), and ammonium formate (1.17 g, 18.6 mmol, 4 equiv.) were added to the solution and the mixture was refluxed for 2 hours. After 2 hours the reaction crude was filtered through celite, washed with MeOH, and concentrated under vacuum. The desired product **S13** was obtained as a white solid (787.8 mg, 3.9 mmol, 84%) after flash column chromatography (heptane to 15% EtOAc in heptane).

In a round bottom flask, **S13** (787.7 mg, 3.9 mmol, 1 equiv.) was added, dissolved in 8 mL of THF, and triethylamine was added (702 µL, 5.0 mmol, 1.3 equiv.). The solution was cooled to 0 °C and a solution of 4-bromobenzoyl chloride (935.2 mg, 4.3 mmol, 1.1 equiv) was added dropwise. The mixture was allowed to stir overnight while warming up to room temperature. The reaction mixture was concentrated and purified by flash column chromatography (3% EtOAc in heptane to 25% EtOAc in heptane) to yield the final compound **S14** (1.44 g, 3.7 mmol, 96%) as a white solid.

^{1}H-NMR (400 MHz, $CDCl_3$) δ_H (ppm): 7.74 (d, J = 8.6 Hz, 2H), 7.67 (br, 1H), 7.62 (d, J = 8.7 Hz, 2H), 7.50 (d, J = 2.3 Hz, 1H), 7.41 (dd, J = 8.5, 2.4 Hz, 1H), 7.31 (d, J = 8.6 Hz, 1H), 1.69 (s, 4H), 1.30 (s, 6H), 1.28 (s, 6H). **^{13}C-NMR (101 MHz, $CDCl_3$) δ_C** (ppm): 164.8, 146.0, 141.9, 135.2, 134.1, 132.1, 128.7, 127.5, 126.6, 118.4, 118.3, 35.2, 35.1, 34.6, 34.2, 32.0, 31.9.

The spectral data is consistent with those reported in literature:
Takashima-Hirano, M., *et. al. ACS Med. Chem. Lett.* **2012**, 3, 804–807

4-bromo-N,N-dipropylbenzenesulfonamide

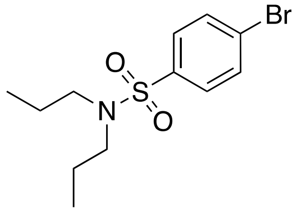

The procedure was modified from Chen, T. Q., *et. al. J. Am. Chem. Soc.* **2022**, *144*, 8296–8305.

Inside the glovebox, a flame-dried 8 mL quartz flask equipped with a stir bar was charged with $[Cu(MeCN)_4]BF_4$ (31.5 mg, 0.2 equiv., 0.1 mmol), *N*-fluoro-2,4,6-trimethylpyridinium tetrafluoroborate (NFTPT) (113.5 mg, 1.0 mmol, 0.5 mmol), 1,3-dibromo-5,5-dimethylhydantoin (DBDMH) (107.2 mg, 0.75 equiv., 0.38 mmol), probenecid (142.7 mg, 1.0 equiv., 0.5 mmol) and 5 mL of anhydrous MeCN. The flask was closed air-tight with a screw cap and the reaction was irradiated with a Kessil® PR160L-370 lamp with the flask touching the lamp. After stirring the reaction at 1500 rpm for 6h it was diluted with 1M NH_4Cl and extracted with EtOAc (4 x 10 mL). The combined organic layers were dried over $MgSO_4$ and the solvent was removed *in vacuo*. Flash column chromatography (heptane to 10% EtOAc in heptane) yielded the title compound as a colorless solid (84.9 mg, 0.27 mmol, 53%).

^{1}H-NMR (400 MHz, $CDCl_3$) δ_H (ppm): 7.72 – 7.59 (m, 4H), 3.11 – 3.01 (m, 4H), 1.54 (sext, J = 7.5 Hz, 4H), 0.86 (t, J = 7.4 Hz, 6H). **^{13}C-NMR (101 MHz, $CDCl_3$) δ_C** (ppm): 139.4, 132.3, 128.7, 127.2, 50.1, 22.1, 11.3.

The spectral data is consistent with those reported in literature:
Kayumov, M., *et al. Adv. Synth. Catal.*, **2020**, *362*(4), 776–781.

6 Synthesis of aryl carboxylic acids

Cleaning procedure used for all electrochemical carboxylation reactions:

Once the reaction is completed both cathodic and anodic crude mixtures are combined and quenched to obtain the desired product as described below. After that, it is important to realize a methodic cleaning of the reaction glassware to ensure no catalyst or organic compounds are left in the frit. In this cleaning, it is important to only add the solvent or aqua regia in the cathodic chamber and allow it to diffuse to the anodic chamber, what will ensure that the liquid goes through the frit and thus allow its cleaning. It starts by adding acetone (2 times) followed by water (2 times) and aqua regia (1 time). With this you can ensure proper cleaning but to avoid leftover aqua regia in the frit further rinsing is necessary. First water (2 times) followed by 96% ethanol (2 times) is used to ensure no aqua regia is left and proper drying of the glassware.

6.1 General Procedure for the electrocarboxylation of aryl bromides and aryl fluorosulfates with $^{12}CO_2$ (1 atm) and ascorbic acid as electron donor (General procedure A)

The cathodic chamber was charged with $BINAPPdCl_2$ (3.2 mg, 1 mol%), starting material (for solid precursors) (0.4 mmol, 1 equiv.), and $TBABF_4$ (198 mg). The anodic chamber was charged with ascorbic acid (141mg, 2 equivalents) and $TBABF_4$ (198 mg), followed by the addition of 2 mL of DMF in the anodic chamber and 2 mL in the cathodic chamber (Solvent addition was made using a 5mL single-use plastic syringe). After this, the starting material was weighted directly into the cathodic chamber (for liquid starting materials) and the last 1 mL of solvent is used to rinse the walls of the cathodic chamber, the solvent at the anodic chamber was leveled with the one over the cathodic side. The electrodes were rinsed with DMF before introducing them into the solution, then the setup is closed on both sides. The atmosphere of the reaction is exchanged with 3 cycles of vacuum, CO_2 using a needle attached to a vac-line. The reaction is allowed to stir until everything is soluble, then it is attached to the Electroware (galvanostat equipment) that applied a constant current for 2.2 mols of electrons.

6.2 General Procedure for the electrocarboxylation of aryl bromides and aryl fluorosulfates with $^{12}CO_2$ (1 atm) and ethanol as additive (General procedure B).

The cathodic chamber was charged with $BINAPPdCl_2$ (3.2 mg, 1 mol%), starting material (for solid precursors) (0.4 mmol, 1 equiv.), and $TBABF_4$ (198 mg). The anodic chamber was charged with $TBABF_4$ (198 mg), followed by the addition of 2 mL of DMF in the anodic chamber and 2 mL in the cathodic chamber (Solvent addition was made using a 5mL single-use plastic syringe). After this, the starting material was weighted directly inside the cathodic chamber (for liquid starting materials) followed by the addition of 99 µL of ethanol 96% in the anodic chamber (for the case in which ethanol was used as an additive). The last 1 mL of solvent is used to rinse the walls of the cathodic chamber, the

solvent at the anodic chamber was leveled with the one over the cathodic side. The electrodes were rinsed with DMF before introducing them into the solution, then the setup is closed on both sides. The atmosphere of the reaction is exchanged with 3 cycles of vacuum, CO_2 using a needle attached to a vac-line. The reaction is allowed to stir until everything is soluble, then it is attached to the Electroware (Galvanstat equipment or potentiostat for the constant potential reactions) that applied 2.2 mols of electrons.

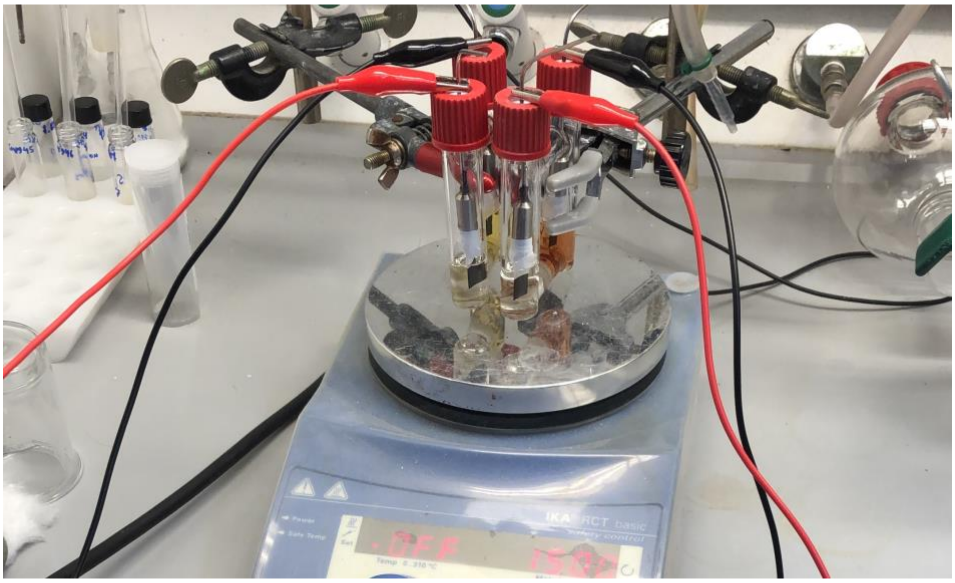

Supplementary Figure 39 Picture of the setup for the electrocarboxylation of aryl electrophiles with excess CO_2

6.3 General Procedure for the electrocarboxylation of aryl bromides and aryl fluorosulfates with CO_2 (1.5 equiv.) (General procedure C).

The cathodic chamber was charged with BINAPPdCl$_2$ (3.2 mg, 1 mol%), starting material (0.4 mmol, 1 equiv.) and TBABF$_4$ (198 mg). The anodic chamber was charged with TBABF$_4$ (198 mg). The gas release chamber was charged with $BaCO_3$ (118 mg, 1.5 equiv.) and CSA (280 mg, 3 equiv.). Followed by the addition of 2 mL of DMF in the anodic chamber and 2 mL in the cathodic chamber (solvent addition was made using a 5mL single-use plastic syringe). After this, the starting material was weighted directly inside the cathodic chamber (for liquid starting materials) followed by the addition of 99 µL of ethanol 96% in the anodic chamber (for the case in which ethanol was used as an additive). The last 1 mL of solvent is used to rinse the walls of the cathodic chamber, the solvent at the anodic chamber was leveled with the one over the cathodic side. The electrodes were rinsed with DMF before introducing them into the solution, then the setup is closed on all 3 sides. The atmosphere of the reaction is exchanged with 3 cycles of vacuum, argon using a needle attached to a vac-line. After, 1.25 mL of layered ethylene glycol/H_2O (4:1) is added through the wall of the gas release chamber, this is added with a syringe with the pierce trough septum (the solvents were layered inside the syringe). The reaction is allowed to stir for 10 minutes for the gas release to occur and for

everything to become soluble. Then the Electroware is attached and applies a constant current for 2.2 mols of electrons.
The solvent layering inside the syringe is done by first taking around 0.3 mL of water followed by excluding the residual air from the syringe while making sure only 0.25 mL of water is left in the syringe. This is followed by slowly taking ethylene glycol in the syringe (to avoid mixing) until the 1.25 mL mark is reached.

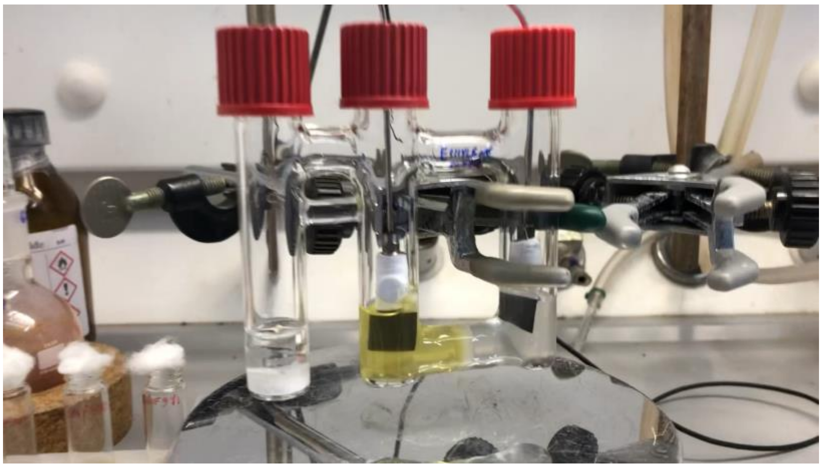

Supplementary Figure 40 Picture of the setup for the electrocarboxylation of aryl electrophiles with near-stoichiometric release of CO_2

6.4 General Procedure for the electrocarboxylation of aryl bromides and aryl fluorosulfates with CO_2 (1 atm) and acetonitrile as solvent (General procedure D).

In the benchtop the cathodic compartment of a two-chamber electrochemistry glassware was charged with $BINAPPdCl_2$ (6.4 mg, 2 mol%), starting material (for solid precursors) (0.4 mmol, 1 equiv.), $TBABF_4$ (171 mg), and TBABr (26 mg). The anodic chamber was charged with $TBABF_4$ (198 mg), followed by the addition of 2 mL of ACN in the anodic chamber and 2 mL in the cathodic chamber (solvent addition was made using a 5mL single-use plastic syringe). After this, the starting material was weighted directly into the cathodic chamber (for liquid starting materials) and triethylamine (112 µL, 0.8 mmol, 2 equiv.) was added in the anodic chamber, the last 1 mL of solvent is used to rinse the walls of the reactor, the solvent at the anodic chamber was leveled with the one over the cathodic side. The electrodes were introduced into the solution, then the setup is closed on both sides. The atmosphere of the reaction is exchanged with 3 cycles of vacuum, CO_2 using a needle attached to a Schlenk-line, in the last cycle, the reaction is allowed to stir for 1 minute under CO_2 before the current is applied. Then, the electrodes are attached to the Electroware that applied a constant current of -4 mA for 2.2 mols of electrons.

Purification method I

Once the reaction is done it is quenched with 1M HCl (2mL), diluted in 20mL of water, and extracted 3 times with EtOAc (10 mL) (a fourth extraction is done and by TLC analysis of the 4th organic fraction decision is taken if further extractions are required), the combined organic phases were dried with sodium sulfate and concentrated under reduced pressure, the product was obtained after column chromatography.

Purification method II

Once the reaction is done it is quenched with 1M HCl (2mL) and extracted 4 times with Et_2O, the combined organic phases were washed 2 times with 1M NH_4Cl, once with water (this was checked by TLC for the product if none is present it is discarded). The same organic phase is extracted 3 times with 0.1M NaOH solution that has the pH adjusted to 1 and extracted 4 times with Et_2O. The combination of these last organic fractions is dried with sodium sulfate and concentrated under reduced pressure to provide the desired carboxylic acid.

Purification method III

Once the reaction is done 2 equivalents of MeI are added and the reaction is let to stir for another hour, at which point it is diluted in 20mL of water, and extracted 3 times with EtOAc (10 mL) (a fourth extraction is done and by TLC analysis of the 4th organic fraction decision is taken if further extractions are required), the combined organic phases were dried with sodium sulfate and concentrated under reduced pressure. This concentrate was added straight to a column packed in pentane, and a flash column was made with the described eluent to afford the desired methyl ester.

Purification method IV

To the reaction mixture, 15 mL of 1 M NaOH (15 mL) + H_2O (5 mL) were added and washed with DCM (3 x 15 mL). The basic solution was acidified to < pH 2 using 4 M HCl and extracted with EtOAc (4 x 20 mL). Drying the combined organic layers over $MgSO_4$ and removing the solvent in vacuo yielded the desired product.

Purification method V

Once the reaction is done its content is transferred to a round bottom flask in which 2M HCl (1 mL) is added to quench the reaction, the flask was rinsed with EtOAc and transferred to the same round bottom flask which had the solvents concentrated by blowing nitrogen. Once the solvent is concentrated the crude mixture is diluted in DMSO and filtered with a syringe filter into a 5 mL vial, the round bottom flask is rinsed three times and filtered into the same vial. The crude is then purified by preparative HPLC (C_{18} 10 µm 19x250 mm column) with the described eluent to afford the desired product.

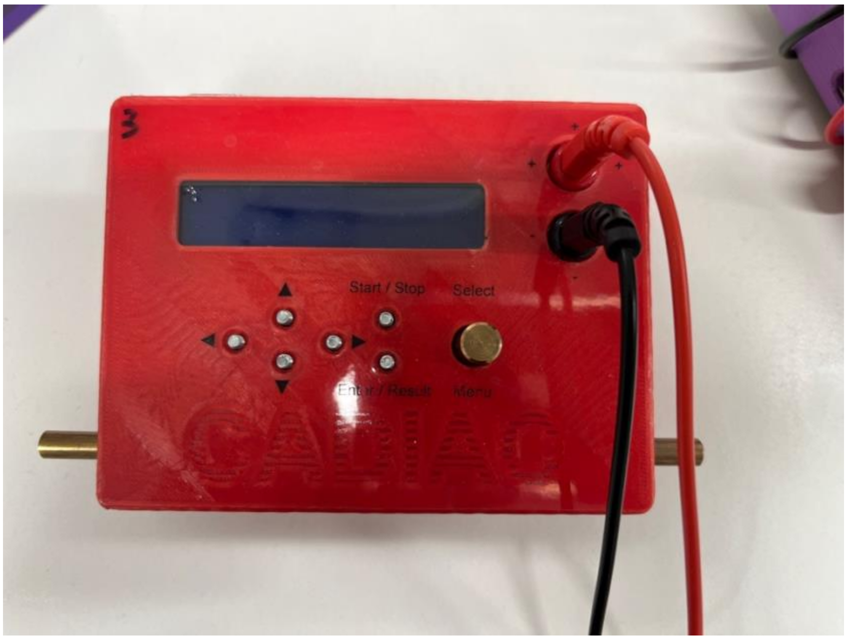

Supplementary Figure 41 Electroware equipment used for the constant current experiments.

6.5 Faradaic efficiency:

All reactions were run with 2.2 equivalents of electrons relative to the mmol of the starting material. Thus, the Faradaic efficiency is calculated as demonstrated below (Supplementary Supplementary Equation **3**) and the results are summarized in Supplementary Figure 42.

$$(10)\ Q_{total} = mmol_{starting\ material} \times 2.2 \times F$$

$$(11)\ Q_{consumed} = mmol_{product} \times 2 \times F$$

$$(12)\ F.E. = \frac{Q_{consumed}}{Q_{total}} \times 100$$

$$(13)\ F.E. = \frac{mmol_{product} \times 2 \times F}{mmol_{starting\ material} \times 2.2 \times F} \times 100$$

$$(14)\ F.E. = \frac{mmol_{product}}{mmol_{starting\ material}} \times 100 \times \frac{2}{2.2}$$

$$(15)\ F.E. = Yield(\%) \times \frac{2}{2.2}$$

Supplementary Equation 3 Formula used for the Faradic Efficiency calculation.

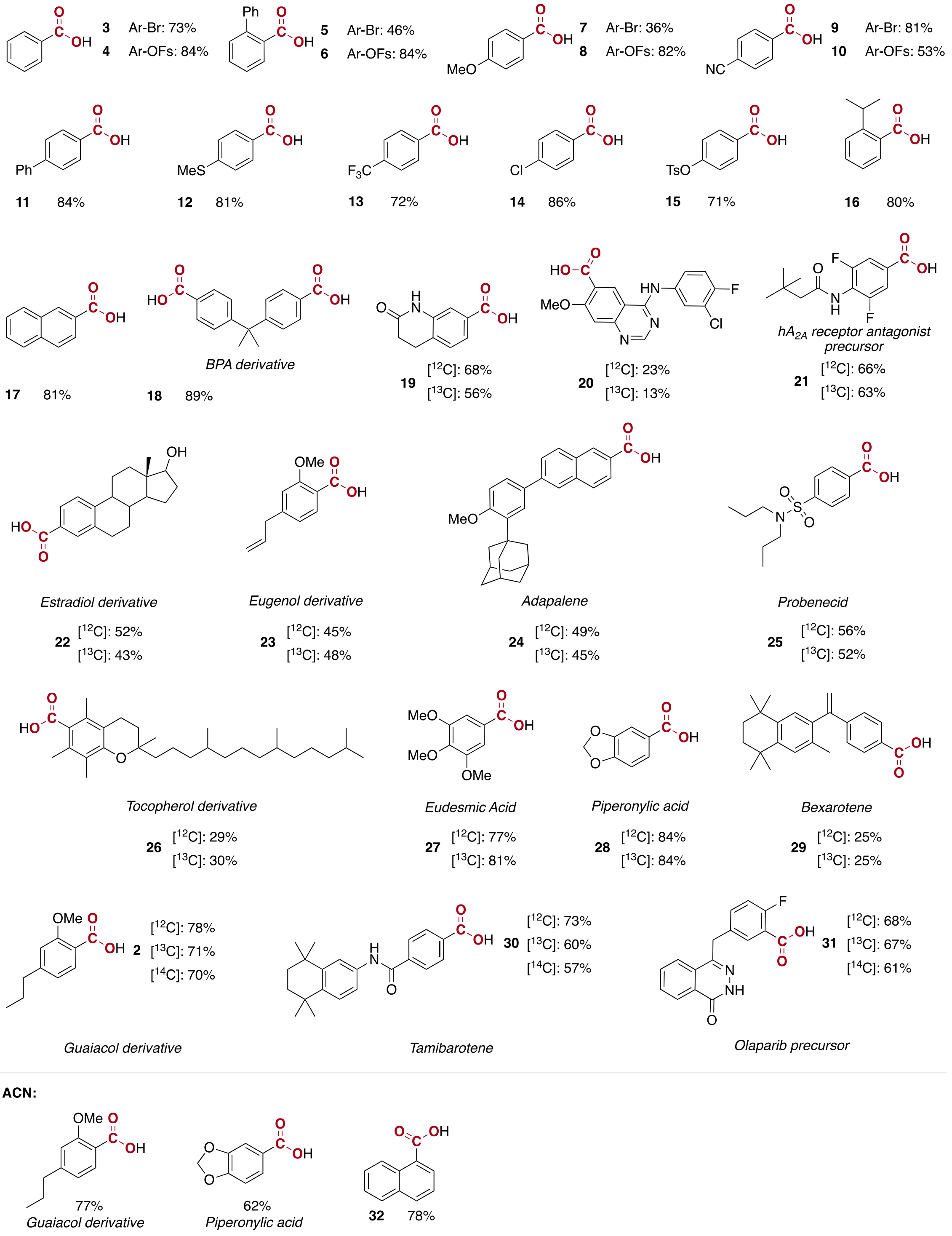

Supplementary Figure 42 Faradaic Efficiencies

6.6 Synthesized carboxylic acids:

Benzoic acid (3 and 4)

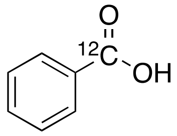

The title compound was prepared according to general procedure A (with a constant current of -2 mA for 96.6 C) employing bromobenzene (71.5 mg, 0.45 mmol) as starting material. Purification method IV yielded the title compound as an off-white solid (44.2 mg, 0.36 mmol, 80%). F.E. = 73%.

The title compound was prepared according to general procedure A (with a constant current of -2 mA for 90 C) employing bromobenzene (66.5 mg, 0.42 mmol) as starting material. Purification method IV yielded the title compound as an off-white solid (41.3 mg, 0.34 mmol, 80%). F.E. = 73%.

The title compound was prepared according to general procedure A (with a constant current of -4 mA for 82.6 C) employing phenyl fluorosulfate (68.6 mg, 0.39 mmol) as starting material. Purification method IV yielded the title compound as an off-white solid (45.2 mg, 0.37 mmol, 95%). F.E. = 86%.

The title compound was prepared according to general procedure B (with a constant current of -4 mA for 90.4 C) employing phenyl fluorosulfate (75.0 mg, 0.43 mmol) as starting material. Purification method IV yielded the title compound as an off-white solid (46.5 mg, 0.38 mmol, 90%). F.E. = 82%.

^{1}H-NMR (400 MHz, $CDCl_3$) δ_H (ppm): 8.13 (d, J = 7.2 Hz, 2H), 7.63 (t, J = 7.5 Hz, 1H), 7.49 (t, J = 7.8 Hz, 2H).**^{13}C-NMR (101 MHz, $CDCl_3$) δ_C** (ppm): 172.0, 134.0, 130.4, 129.4, 128.6.

The spectral data is consistent with those reported in literature:
Yuan, Y.-C., *et al. Org. Lett.* **2017**, *19*, 6404–6407.

[1,1'-biphenyl]-2-carboxylic acid (5 and 6)

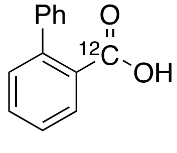

The title compound was prepared according to general procedure B (with a constant current of -4 mA for 85 C) employing [1,1'-biphenyl]-2-yl sulfurofluoridate (101.3 mg,

0.40 mmol) as starting material. Purification method II yielded the title compound as a white crystalline solid (74.3 mg, 0.38 mmol, 94%). F.E. = 85%.

The title compound was prepared according to general procedure B (with a constant current of -4 mA for 85 C) employing [1,1'-biphenyl]-2-yl sulfurofluoridate (101 mg, 0.40 mmol) as starting material. Purification method II yielded the title compound as a white crystalline solid (73.2 mg, 0.37 mmol, 92%). F.E. = 84%.

The title compound was prepared according to general procedure B (with a constant current of -2mA for 86.8 C) employing [1,1'-biphenyl]-2-Bromo (95.3 mg, 0.41 mmol) as starting material. Purification method II yielded the title compound as a white crystalline solid (38.5 mg, 0.19 mmol, 49%). F.E. = 45%.

The title compound was prepared according to general procedure B (with a constant current of -2mA for 85 C) employing [1,1'-biphenyl]-2-Bromo (93.2 mg, 0.40 mmol) as starting material. Purification method II yielded the title compound as a white crystalline solid (41.2 mg, 0.21 mmol, 52%). F.E. = 47%.

^{1}H NMR (400 MHz, $CDCl_3$) δ_H (ppm): 7.95 (dd, *J* = 7.8, 1.5 Hz, 1H), 7.57 (td, *J* = 7.5, 1.4 Hz, 1H), 7.44 – 7.32 (m, 7H). **^{13}C NMR (101 MHz, $CDCl_3$) δ_C** (ppm): 173.45, 143.5, 141.2, 132.2, 131.4, 130.8, 129.4, 128.6, 128.2, 127.5, 127.3.

The spectral data is consistent with those reported in the literature:
Bhunia, SM., *et al. Org. Lett.* **2019**, *21*, 4632–4637

4-Methoxybenzoic acid (7 and 8)

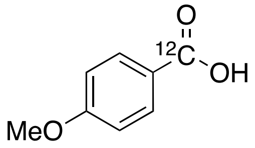

The title compound was prepared according to general procedure A (with a constant current of -4 mA for 86.4 C) employing 4-methoxyphenyl sulfurofluoridate (84 mg, 0.41 mmol) as starting material. Purification method I (Pentane to 30% EtOAc in Pentane) yielded the title compound as an off-white crystalline solid (59.4 mg, 0.39 mmol, 96%). F.E. = 87%.

The title compound was prepared according to general procedure B (with a constant current of -4 mA for 85 C) employing 4-methoxyphenyl sulfurofluoridate (82.3 mg, 0.40 mmol) as starting material. Purification method I (Pentane to 30% EtOAc in Pentane) yielded the title compound as an off-white crystalline solid (51.8 mg, 0.34 mmol, 85%). F.E. = 77%.

The title compound was prepared according to general procedure A (with a constant current of -4 mA for 87 C) employing 4-Bromo anisole (77 mg, 0.41 mmol) as starting material. Purification method I (Pentane to 30% EtOAc in Pentane) yielded the title compound as a yellowish crystalline solid (26.2 mg, 0.17 mmol, 42%). F.E. = 38%.

The title compound was prepared according to general procedure B (with a constant current of -4 mA for 85 C) employing 4-Bromo anisole (75 mg, 0.40 mmol) as starting material. Purification method I (Pentane to 30% EtOAc in Pentane) yielded the title compound as a yellowish crystalline solid (22.3 mg, 0.15 mmol, 37%). F.E. = 34%.

^{1}H NMR (400 MHz, $CDCl_3$) δ_H (ppm): 8.07 (d, *J* = 9.0 Hz, 2H), 6.95 (d, *J* = 9.0 Hz, 2H), 3.88 (s, 3H). **^{13}C NMR (101 MHz, $CDCl_3$) δ_C** (ppm): 171.7, 164.2, 132.5, 121.8, 113.9, 55.6.

The spectral data is consistent with those reported in the literature:
Sun, GQ., *et al. Nat Commun* **2021,** *12*, 7086.

4-cyanobenzoic acid (9 and 10)

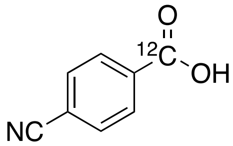

The title compound was prepared according to general procedure A (with a constant current of -4 mA for 85 C) employing 4-cyanophenyl sulfurofluoridate (80.5 mg, 0.40 mmol) as starting material. Purification method I (Heptane to 40% EtOAc in Heptane, the eluent was acidified with formic acid 0.5%) yielded the title compound as a yellowish crystalline solid (31.7 mg, 0.22 mmol, 54%). F.E. = 49%.

The title compound was prepared according to general procedure B (with a constant potential of -1.8 V (vs. sat. Ag/AgCl) for 85 C) employing 4-cyanophenyl sulfurofluoridate (80.5 mg, 0.40 mmol) as starting material. Purification method I (Heptane to 40% EtOAc in Heptane, the eluent was acidified with formic acid 0.5%) yielded the title compound as a white crystalline solid (36.3 mg, 0.25 mmol, 62%). F.E. = 56%

The title compound was prepared according to general procedure A (with a constant current of -2 mA for 85 C) employing 4-cyanophenyl bromide (72.2 mg, 0.40 mmol) as starting material. Purification method I (Flash column chromatography pentane to 30% acetone in pentane, the eluent was acidified with 1% formic acid) yielded the title compound as a colorless solid (52.4 mg, 0.36 mmol, 90%). F.E. = 82%.

The title compound was prepared according to general procedure A (with a constant current of -2 mA for 85 C) employing 4-cyanophenyl bromide (72.3 mg, 0.40 mmol) as starting material. Purification method I (Flash column chromatography pentane to 30% acetone in pentane, the eluent was acidified with 1% formic acid) yielded the title compound as a colorless solid (52.3 mg, 0.36 mmol, 89%). F.E. = 81%.

^{1}H NMR (400 MHz, $CDCl_3$) δ_H (ppm): 8.22 (d, J = 8.3 Hz, 2H), 7.80 (d, J = 8.4 Hz, 2H). **^{13}C NMR (101 MHz, $CDCl_3$) δ_C** (ppm): 169.4, 133.0, 132.5, 130.8, 117.9, 117.5.

The spectral data is consistent with those reported in the literature:
Bhunia, SM., *et al. Org. Lett.* **2019**, *21*, 4632–4637

[1,1'-biphenyl]-4-carboxylic acid (11)

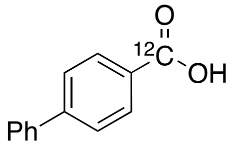

The title compound was prepared according to general procedure A (with a constant current of -4 mA for 85 C) employing [1,1'-biphenyl]-4-yl sulfurofluoridate (101.0 mg, 0.40 mmol) as starting material. Purification method I (Pentane to 30% EtOAc in Pentane) yielded the title compound as an off-white crystalline solid (71.2 mg, 0.36 mmol, 90%). F.E. = 82%.

The title compound was prepared according to general procedure C (with the use of $Ba^{12}CO_3$, AA as the additive and a constant current of -4 mA for 85 C) employing [1,1'-biphenyl]-4-yl sulfurofluoridate (101 mg, 0.40 mmol) as starting material. Purification method I (Pentane to 30% EtOAc in Pentane) yielded the title compound as an off-white crystalline solid (74.9 mg, 0.38 mmol, 94%). F.E. = 85%.

^{1}H NMR (400 MHz, DMSO-d_6) δ_H (ppm): 12.98 (s, 1H), 8.02 (d, J = 8.6 Hz, 2H), 7.80 (d, J = 8.4 Hz, 2H), 7.73 (d, J = 7.3 Hz, 2H), 7.50 (t, J = 7.5 Hz, 2H), 7.42 (t, J = 7.3 Hz, 1H). **^{13}C NMR (101 MHz, DMSO-d_6) δ_C** (ppm): 167.2, 144.3, 139.0, 130.0, 129.6, 129.1, 128.3, 127.0, 126.8.

The spectral data is consistent with those reported in the literature:
Sun, GQ., *et al. Nat Commun* **2021,** *12*, 7086.

4-(methylthio)benzoic acid (12)

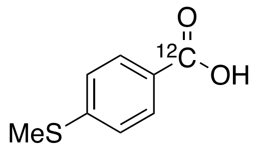

The title compound was prepared according to general procedure A (with a constant current of -4 mA for 76.4 C) employing 4-(methylthio)phenyl sulfurofluoridate (87 mg, 0.39 mmol) as starting material. Purification method I (Pentane to 30% EtOAc in Pentane) yielded the title compound as a pale-yellow powder (60 mg, 0.36 mmol, 91%). F.E. = 83%.

The title compound was prepared according to general procedure B (with a constant current of -4 mA for 85 C) employing 4-(methylthio)phenyl sulfurofluoridate (88.9 mg, 0.40 mmol) as starting material. Purification method I (Pentane to 30% EtOAc in Pentane) yielded the title compound as a pale-yellow powder (58.2 mg, 0.35 mmol, 87%). F.E. = 79%.

^{1}H NMR (400 MHz, $CDCl_3$) δ_H (ppm): 8.01 (d, *J* = 8.6 Hz, 2H), 7.28 (d, *J* = 8.6 Hz, 2H), 2.53 (s, 3H). **^{13}C NMR (101 MHz, $CDCl_3$) δ_C** (ppm): 171.6, 146.9, 130.6, 125.3, 125.0, 14.9.

The spectral data is consistent with those reported in the literature:
Sun, GQ., *et al. Nat Commun* **2021,** *12*, 7086.

4-(trifluoromethyl)benzoic acid (13)

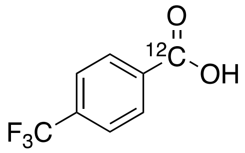

The title compound was prepared according to general procedure B (with a constant current of -4 mA for 88.7 C) employing p-trifluoromethylphenyl bromide (94.0 mg, 0.42 mmol) as starting material. Purification method I (Flash column chromatography 30-100% EtOAc in heptane, eluent was acidified with 1% formic acid) yielded the title compound as a colorless solid (64.4 mg, 0.34 mmol, 81%). F.E. = 74%.

The title compound was prepared according to general procedure B (with a constant current of -4 mA for 87.7 C) employing p-trifluoromethylphenyl bromide (93.0 mg, 0.41 mmol) as starting material. Purification method I (Flash column chromatography 30-100% EtOAc in heptane, eluent was acidified with 1% formic acid) yielded the title compound as a colorless solid (60.5 mg, 0.32 mmol, 77%). F.E. = 70%.

^{1}H NMR (400 MHz, DMSO-d$_6$) δ_H (ppm): 8.13 (d, J = 8.2 Hz, 2H), 7.87 (d, J = 8.4 Hz, 2H). **^{13}C NMR (101 MHz, DMSO-d$_6$) δ_C** (ppm): 166.2, 134.6, 132.5 (q, J = 32.1 Hz), 130.2, 125.7 (q, J = 3.9 Hz), 123.9 (d, J = 272.9 Hz). **^{19}F NMR (376 MHz, DMSO-d$_6$) δ_F** (ppm): -61.6.

The spectral data is consistent with those reported in the literature:
Bhunia, SM., *et al. Org. Lett.* **2019**, *21*, 4632–4637

4-chlorobenzoic acid (14)

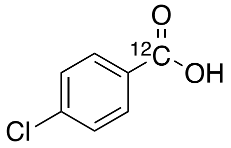

The title compound was prepared according to general procedure B (with a constant current of -2 mA for 85 C) employing 1-bromo-4-chlorobenzene (75.6 mg, 0.40 mmol) as starting material. Purification method II yielded the title compound as a white solid (56.0 mg, 0.36 mmol, 91%). F.E. = 83%.

The title compound was prepared according to general procedure B (with a constant current of -2 mA for 86.4 C) employing 1-bromo-4-chlorobenzene (77.9 mg, 0.41 mmol) as starting material. Purification method II yielded the title compound as a white solid (62.4 mg, 0.40 mmol, 98%). F.E. = 89%.

^{1}H NMR (400 MHz, DMSO-d$_6$) δ_H (ppm): 13.18 (s, 1H), 7.94 (d, J = 8.6 Hz, 2H), 7.57 (d, J = 8.7 Hz, 2H). **^{13}C NMR (101 MHz, DMSO-d$_6$) δ_C** (ppm): 166.5, 137.8, 131.2, 129.6, 128.8.

The spectral data is consistent with those reported in the literature:
Ma, C., *et al. Org. Lett.* **2019**, *21*, 2464–2467

4-(tosyloxy)benzoic acid (15)

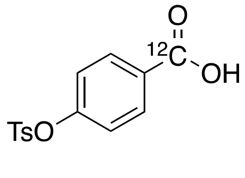

The title compound was prepared according to general procedure B (with a constant current of -2 mA for 85 C) employing 4-bromophenyl 4-methylbenzenesulfonate (124 mg, 0.40 mmol) as starting material. Purification method II yielded the title compound as a white solid (86.3 mg, 0.30 mmol, 74%). F.E. = 67%.

The title compound was prepared according to general procedure B (with a constant current of -2 mA for 85 C) employing 4-bromophenyl 4-methylbenzenesulfonate (124 mg, 0.40 mmol) as starting material. Purification method II yielded the title compound as a white solid (95.3 mg, 0.33 mmol, 82%). F.E. = 75%.

^{1}H NMR (400 MHz, CDCl$_3$) δ$_H$ (ppm): 8.05 (d, J = 8.8 Hz, 2H), 7.72 (d, J = 8.4 Hz, 2H), 7.33 (d, J = 8.2 Hz, 2H), 7.11 (d, J = 8.8 Hz, 2H), 2.46 (s, 3H). **^{13}C NMR (101 MHz, CDCl$_3$) δ$_C$** (ppm): 170.9, 153.8, 146.0, 132.2, 130.1, 128.6, 128.1, 122.6, 21.9.

The spectral data is consistent with those reported in the literature:
Friis, D. S., *et al. Org. Lett.* **2013**, *15*, 1378–1381

2-isopropylbenzoic acid (16)

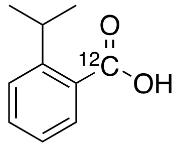

The title compound was prepared according to general procedure B (with a constant current of -4 mA for 87.2 C) employing 2-isopropylphenyl sulfurofluoridate (89.7 mg, 0.41 mmol) as starting material. Purification method II yielded the title compound as a pale-yellow powder (59.4 mg, 0.36 mmol, 88%). F.E. = 80%.

The title compound was prepared according to general procedure B (with a constant current of -4 mA for 85 C) employing 2-isopropylphenyl sulfurofluoridate (88.0 mg, 0.40 mmol) as starting material. Purification method II yielded the title compound as a pale-yellow powder (57.6 mg, 0.35 mmol, 87%). F.E. = 79%.

^{1}H NMR (400 MHz, CDCl$_3$) δ$_H$ (ppm): 7.94 (dd, *J* = 7.9, 1.5 Hz, 1H), 7.57 – 7.48 (m, 1H), 7.47 (dd, *J* = 7.9, 1.6 Hz, 1H), 7.30 – 7.23 (m, 1H), 3.95 (hept, *J* = 6.8 Hz, 1H), 1.29 (d, *J* = 6.8 Hz, 6H). **^{13}C NMR (101 MHz, CDCl$_3$) δ$_C$** (ppm): 174.0, 151.2, 132.9, 131.0, 128.4, 126.6, 125.7, 29.5, 24.2.

The spectral data is consistent with those reported in the literature:
Wang, H., *et al. Chem. Eur. J.,* **2009,** *15,* 1499-1507

2-naphthoic acid (17)

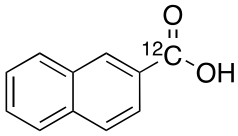

The title compound was prepared according to general procedure B (with a constant current of -4 mA for 85 C) employing naphthalen-2-yl sulfurofluoridate (90.5 mg, 0.40 mmol) as starting material. Purification method II yielded the title compound as a pale-yellow powder (61.8 mg, 0.36 mmol, 89%). F.E. = 81%.

The title compound was prepared according to general procedure B (with a constant current of -4 mA for 85 C) employing naphthalen-2-yl sulfurofluoridate (90.5 mg, 0.40 mmol) as starting material. Purification method II yielded the title compound as a pale-yellow powder (62.1 mg, 0.36 mmol, 90%). F.E. = 82%.

^{1}H NMR (400 MHz, $CDCl_3$) δ_H (ppm): 8.74 (s, 1H), 8.14 (dd, *J* = 8.6, 1.8 Hz, 1H), 8.00 (d, *J* = 7.1 Hz, 1H), 7.92 (t, *J* = 7.5 Hz, 2H), 7.66 – 7.55 (m, 2H). **^{13}C NMR (101 MHz, $CDCl_3$) δ_C** (ppm): 172.3, 136.1, 132.6, 132.3, 129.7, 128.8, 128.5, 128.0, 126.9, 126.6, 125.5

The spectral data is consistent with those reported in the literature:
Sedelmeier, J., *et al. Org. Lett.*, **2010**, *12*, 3618–3621

4,4'-(propane-2,2-diyl)dibenzoic acid (18)

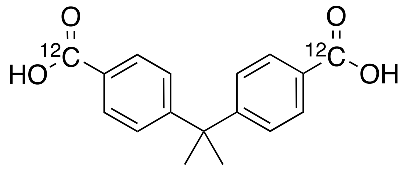

The title compound was prepared according to general procedure B (with a constant current of -2 mA for 169.8 C) employing propane-2,2-diylbis(4,1-phenylene) bis(sulfurofluoridate) (157 mg, 0.40 mmol) as starting material. Purification method II (employingEtOAc for the organic phase) yielded the title compound as a white solid (110.8 mg, 0.39 mmol, 97%). F.E. = 88%.

The title compound was prepared according to general procedure B (with a constant current of -2 mA for 169.8 C) employing propane-2,2-diylbis(4,1-phenylene) bis(sulfurofluoridate) (157 mg, 0.40 mmol) as starting material. Purification method II (employingEtOAc for the organic phase) yielded the title compound as a white solid (111.0 mg, 0.39 mmol, 98%). F.E. = 89%.

^{1}H NMR (400 MHz, Acetone-d_6) δ_H (ppm): 7.97 (d, *J* = 8.7 Hz, 4H), 7.40 (d, *J* = 8.7 Hz, 4H), 1.75 (s, 6H). **^{13}C NMR (101 MHz, Acetone-d_6) δ_C** (ppm): 167.4, 156.1, 130.4, 129.1, 127.8, 44.3, 30.6. **HRMS** (ESI-) $C_{17}H_{15}O_4$ $[M-H]^-$; calculated 283.0976, found 283.0973.

methyl 2-oxo-1,2,3,4-tetrahydroquinoline-7-carboxylate (19)

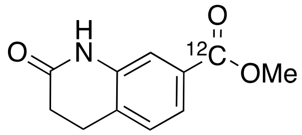

The title compound was prepared according to general procedure B (with a constant current of -4 mA for 85 C) employing 2-oxo-1,2,3,4-tetrahydroquinolin-7-yl sulfurofluoridate (98.1 mg, 0.40 mmol) as starting material. Purification method III (Heptane to 50% acetone in heptane) yielded the title compound as a white powder (62.1 mg, 0.30 mmol, 75%). F.E. = 68%.

^{1}H NMR (400 MHz, Acetone-d$_6$) δ_H (ppm): 9.25 (br, 1H), 7.62 – 7.55 (m, 2H), 7.32 (d, J = 8.2 Hz, 1H), 3.86 (s, 3H), 3.07 – 3.00 (m, 2H), 2.59 – 2.50 (m, 2H). **^{13}C NMR (101 MHz, Acetone-d$_6$) δ_C** (ppm): 170.6, 166.9, 139.8, 130.3, 130.2, 128.9, 124.0, 116.5, 52.3, 30.9, 26.2.

The spectral data is consistent with those reported in the literature:
Zhang, L., *et al. Org. Lett.* **2013**, *15*, 2128–2131

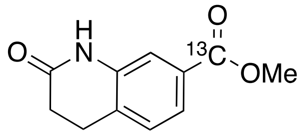

The title compound was prepared according to general procedure C (employing Ba^{13}CO$_3$, EtOH as the additive, and a constant current of -4 mA for 85 C) employing 2-oxo-1,2,3,4-tetrahydroquinolin-7-yl sulfurofluoridate (98.6 mg, 0.40 mmol) as starting material. Purification method III (Flash column chromatography, 10-40% EtOAc in heptane) yielded the title compound as a colourless solid (51.1 mg, 0.25 mmol, 62%). F.E. = 56%.

^{1}H-NMR (400 MHz, CDCl$_3$) δ_H (ppm): 8.18 (s, 1H), 7.67 (ddd, *J* = 7.8, 4.0, 1.6 Hz, 1H), 7.45 (d, *J* = 2.8 Hz, 1H), 7.24 (d, *J* = 7.8 Hz, 1H), 3.91 (d, *J* = 3.8 Hz, 3H), 3.03 (t, *J* = 7.5 Hz, 2H), 2.67 (t, 2H). **^{13}C-NMR (101 MHz, CDCl$_3$) δ_C** (ppm): 171.5, 166.7 (^{13}C-enriched), 137.7 (d, *J* = 5.5 Hz), 129.8 (d, *J* = 75.6 Hz), 129.0, 128.2 (d, *J* = 5.1 Hz), 124.5 (d, *J* = 2.6 Hz), 116.4 (d, *J* = 3.0 Hz), 52.4 (d, *J* = 2.5 Hz), 30.4, 25.7. **HRMS** (ESI+) $C_{10}{}^{13}CH_{12}NO3$ [M+H]$^+$; calculated 207.0845, found 207.0845.

Methyl 4-((3-chloro-4-fluorophenyl)amino)-7-methoxyquinazoline-6-carboxylate (20)

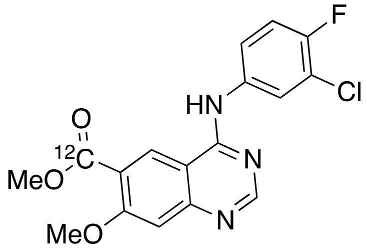

The title compound was prepared according to general procedure B (with a constant current of -4 mA for 85 C) employing 4-((3-chloro-4-fluorophenyl)amino)-7-methoxyquinazolin-6-yl sulfurofluoridate (161 mg, 0.40 mmol) as starting material. Purification method III (10% EtOAc in heptane to pure EtOAc) yielded the title compound as a pale-yellow powder (36 mg, 0.10 mmol, 25%). F.E. = 23%.

^{1}H NMR (400 MHz, Acetone-d$_6$) δ$_H$ (ppm): 9.45 (s, 1H), 8.67 (s, 1H), 8.64 (s, 1H), 8.26 (dd, J = 6.8, 2.7 Hz, 1H), 7.84 (ddd, J = 8.9, 4.2, 2.7 Hz, 1H), 7.36 – 7.28 (m, 2H), 4.03 (s, 3H), 3.89 (s, 3H). **^{13}C NMR (101 MHz, Acetone-d$_6$) δ$_C$** (ppm): 166.6, 162.0, 158.7, 157.3, 155.0 (d, *J* = 243.5 Hz), 154.8, 137.3 (d, *J* = 3.3 Hz), 127.3, 124.7, 123.1 (d, *J* = 6.8 Hz), 122.9, 120.4 (d, *J* = 18.4 Hz), 117.2 (d, *J* = 21.9 Hz), 109.6, 108.7, 56.6, 52.6. **^{19}F NMR (376 MHz, Acetone-d$_6$) δ$_F$** (ppm): -118.5.

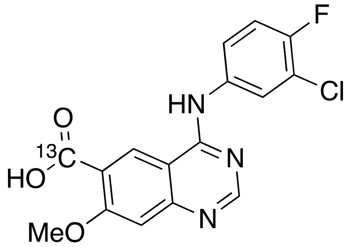

The title compound was prepared according to general procedure C (employing Ba^{13}CO$_3$, EtOH as the additive, and a constant current of -4 mA for 85 C) employing 4-((3-chloro-4-fluorophenyl)amino)-7-methoxyquinazolin-6-yl sulfurofluoridate (161 mg, 0.40 mmol) as starting material. After the reaction was completed the crude was taken into sodium bicarbonate solution and extracted with DCM 3 times, after the water phase was neutralized, solid extracted, concentrated and purified by prep-HPLC (gradient from 5% ACN in pH 10 H_2O solution (NH_4OH) to 80% ACN in pH 10 H_2O solution) to yield the title compound as a pale-yellow powder (21 mg, 0.06 mmol, 14%). F.E. = 13%.

^{1}H NMR (500 MHz, DMSO -d$_6$) δ$_H$ (ppm): 13.19 (s, 1H), 10.05 (s, 1H), 8.88 (d, J = 5.3 Hz, 1H), 8.61 (s, 1H), 8.18 (dd, J = 6.9, 2.6 Hz, 1H), 7.83 (ddd, J = 9.0, 4.3, 2.6 Hz, 1H), 7.44 (t, J = 9.1 Hz, 1H), 7.28 (d, J = 1.7 Hz, 1H), 3.96 (s, 3H). **^{13}C NMR (126 MHz, DMSO-d$_6$) δ$_C$** (ppm): 166.8 (13C enriched), 160.6, 157.6, 156.2, 153.4 (d, J = 243.3),

153.2, 136.4 (d, J = 3.1), 126.4, 123.7, 123.0 (d, J = 73.4), 122.5 (d, J = 6.9), 118.8 (d, J = 18.5), 116.6 (d, J = 21.7), 108.4 (d, J = 4.7), 107.5, 56.2. **^{19}F NMR (471 MHz, DMSO-d$_6$) δ$_F$** (ppm): -122.7. **HRMS** (ESI-) $C_{15}{}^{13}CH_{10}ClFN_3O_3$ $[M-H]^-$; calculated 347.0434, found 347.0433.

4-(3,3-dimethylbutanamido)-3,5-difluorobenzoic acid (21)

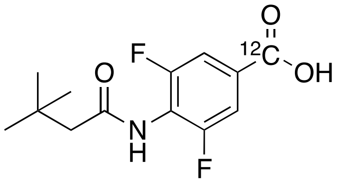

The title compound was prepared according to general procedure A (with a constant current of -4 mA for 85 C) employing N-(4-bromo-2,6-difluorophenyl)-3,3-dimethylbutanamide (122.5 mg, 0.40 mmol) as starting material. Purification method II yielded the title compound as an off-white crystalline solid (79.2 mg, 0.29 mmol, 73%).

^{1}H NMR (500 MHz, DMSO-d$_6$) δ$_H$ (ppm): 9.81 (1H, s), 7.59–7.64 (2H, m), 2.24 (2H, s), 1.03 (9H, s). **^{13}C NMR (126 MHz, DMSO-d$_6$) δ$_C$** (ppm): 169.7, 110.9–114.3 (m), 119.0 (d, *J* = 17.1), 130.3 (d, *J* = 8.4), 157.1 (dd, *J* = 249.8, 5.7), 165.0–165.3 (m), 48.4, 30.7, 29.5.**^{19}F NMR (376 MHz, DMSO-d$_6$) δ$_F$** (ppm): -115.6. F.E. = 66%.

The spectral data is consistent with those reported in the literature:
Korsager, S., *et al. J. Am. Chem. Soc.* **2013**, *135*, 2891–2894

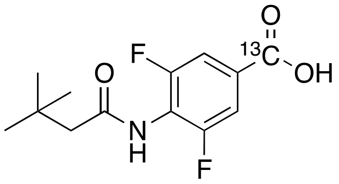

The title compound was prepared according to general procedure C (employing $Ba^{13}CO_3$, EtOH as the additive, and a constant current of -2 mA for 85.7 C) employing *N*-(4-bromo-2,6-difluorophenyl)-3,3-dimethylbutanamide (123.7 mg, 0.40 mmol) as starting material. Purification method I (Flash column chromatography 10-40% EtOAc in heptane, eluent was acidified with 1% formic acid) yielded the title compound as a colourless solid (75.6 mg, 0.28 mmol, 69%). F.E. = 63%.

^{1}H-NMR (400 MHz, DMSO-d$_6$) δ$_H$ (ppm): 13.43 (s, 1H), 9.81 (s, 1H), 7.61 (dd, J = 8.0, 4.4 Hz, 2H), 2.24 (s, 2H), 1.03 (s, 9H).**^{13}C-NMR (101 MHz, DMSO-d$_6$) δ$_C$** (ppm): 169.7, 165.1 (t, *J* = 2.9 Hz, ^{13}C-enriched), 158.4 – 155.9 (m), 130.2 (d, *J* = 73.1 Hz), 119.0 (t, *J* = 17.0 Hz), 112.6 (d, *J* = 23.6 Hz), 48.4, 30.7, 29.5. **^{19}F NMR (376 MHz, DMSO-d$_6$) δ$_F$**

(ppm): -115.6 (d, J = 8.8 Hz). **HRMS** (ESI-) $C_{12}{}^{13}CH_{14}F_2NO_3$ [M-H]$^-$; calculated 271.0981, found 271.0979.

(13S)-17-hydroxy-13-methyl-7,8,9,11,12,13,14,15,16,17-decahydro-6H-cyclopenta[a]phenanthrene-3-carboxylic acid (22)

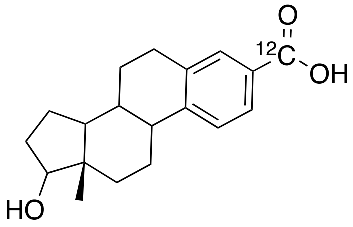

The title compound was prepared according to general procedure C (employing $Ba^{12}CO_3$, ascorbic acid as the electron donor, and a constant current of -4 mA for 85 C) employing (8R,9S,13S,14S,17S)-17-hydroxy-13-methyl-7,8,9,11,12,13,14,15,16,17-decahydro-6*H*-cyclopenta[*a*]phenanthren-3-yl fluorosulfate (141.4 mg, 0.40 mmol) as starting material. Purification method I (Flash column chromatography 20-50% EtOAc in heptane, eluent was acidified with 1% formic acid) yielded the title compound as an off-white solid (67.8 mg, 0.23 mmol, 57%). F.E. = 52%.

^{1}H-NMR (400 MHz, DMSO-d$_6$) δ_H (ppm): 12.69 (s, 1H), 7.66 (d, J = 8.1 Hz, 1H), 7.62 (s, 1H), 7.38 (d, J = 8.1 Hz, 1H), 4.51 (s, 1H), 3.53 (t, J = 8.5 Hz, 1H), 2.98 – 2.69 (m, 2H), 2.40 – 2.24 (m, 1H), 2.28 – 2.15 (m, 1H), 1.93 – 1.75 (m, 3H), 1.66 – 1.53 (m, 1H), 1.48 – 1.00 (m, 7H), 0.66 (s, 3H). **^{13}C-NMR (101 MHz, DMSO-d$_6$) δ_C** (ppm): 167.4, 145.4, 136.6, 129.7, 127.9, 126.5, 125.5, 80.0, 49.6, 44.2, 42.7, 38.0, 36.5, 29.9, 28.8, 26.5, 25.6, 22.8, 11.2.

The spectral data is consistent with those reported in literature:
Shi, Y., *et al. Chem. Bio.*, **2001**, *8*(5), 501–510.

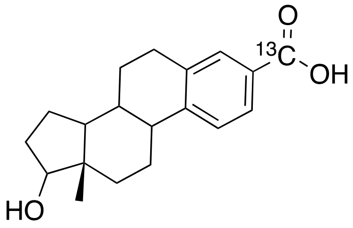

The title compound was prepared according to general procedure C (employing $Ba^{13}CO_3$, ascorbic acid as the electron donor, and a constant current of -4 mA for 87 C) employing (8R,9S,13S,14S,17S)-17-hydroxy-13-methyl-7,8,9,11,12,13,14,15,16,17-decahydro-6*H*-cyclopenta[*a*]phenanthren-3-yl fluorosulfate (145.2 mg, 0.41 mmol) as starting material. Purification method I (Flash column chromatography 20-50% EtOAc in

heptane, eluent was acidified with 1% formic acid) yielded the title compound as an off-white solid (58.1 mg, 0.19 mmol, 47%). F.E. = 43%.

^{1}H-NMR (400 MHz, DMSO-d$_6$) δ$_H$ (ppm): 12.69 (s, 1H), 7.70 – 7.63 (m, 1H), 7.62 (d, *J* = 2.3 Hz, 1H), 7.37 (d, *J* = 8.3 Hz, 1H), 4.50 (s, 1H), 3.52 (t, *J* = 8.4 Hz, 1H), 2.87 – 2.78 (m, 2H), 2.35 – 2.26 (m, 1H), 2.24 – 2.15 (m, 1H), 1.93 – 1.75 (m, 3H), 1.67 – 1.51 (m, 1H), 1.44 – 1.04 (m, 7H), 0.66 (s, 3H). **^{13}C-NMR (101 MHz, DMSO-d$_6$) δ$_C$** (ppm): 167.4 (^{13}C enriched), 145.3, 136.6, 129.7, 127.9 (d, *J* = 71.9 Hz), 126.5, 125.4, 80.0, 49.6, 44.1, 42.7, 38.0, 36.5, 29.9, 28.8, 26.5, 25.6, 22.8, 11.2. **HRMS** (ESI-) calc. for $C_{18}{}^{13}CH_{23}O_3$ [M-H]$^-$: 300.1686, found 300.1684.

4-Allyl-2-methoxybenzoic acid (23)

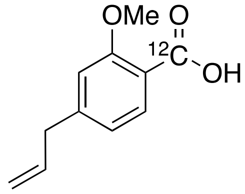

The title compound was prepared according to general procedure B (with a constant current of -1 mA for 88.5 C) employing 4-Allyl-2-methoxyphenyl fluorosulfate (102.8 mg, 0.42 mmol) as starting material. Purification method I (Flash column chromatography 10-40% EtOAc in heptane, eluent was acidified with 1% formic acid) yielded the title compound as an off-white waxy solid (40.5 mg, 0.21 mmol, 50%). F.E. = 45%.

^{1}H-NMR (400 MHz, CDCl$_3$) δ$_H$ (ppm): 8.08 (d, J = 8.2 Hz, 1H), 6.96 (d, J = 8.0 Hz, 1H), 6.86 (s, 1H), 6.09 – 5.77 (m, 1H), 5.27 – 4.99 (m, 2H), 4.06 (s, 3H), 3.44 (d, J = 6.7 Hz, 2H). **^{13}C-NMR (101 MHz, CDCl$_3$) δ$_C$** (ppm): 165.6, 158.2, 148.5, 135.8, 134.0, 122.7, 117.4, 115.6, 111.8, 56.7, 40.4.

The spectral data is consistent with those reported in literature:
Bhunia, S. K., *et al. Org. Lett.*, **2019**, *21*(12), 4632–4637.

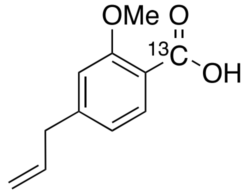

The title compound was prepared according to general procedure C (employing Ba^{13}CO$_3$, EtOH as the additive, and a constant current of -1 mA for 85 C) employing 4-Allyl-2-methoxyphenyl fluorosulfate (98.6 mg, 0.40 mmol) as starting material. Purification

method I (Flash column chromatography 10-40% EtOAc in heptane, the eluent was acidified with 1% formic acid) yielded the title compound as an off-white waxy solid (41.0 mg, 0.21 mmol, 53%). F.E. = 48%.

^{1}H-NMR (400 MHz, $CDCl_3$) δ_H (ppm): 8.09 (dd, *J* = 8.0, 4.6 Hz, 1H), 6.97 (d, *J* = 8.1 Hz, 1H), 6.87 (s, 1H), 6.19 – 5.74 (m, 1H), 5.37 – 4.94 (m, 2H), 4.06 (s, 3H), 3.44 (d, *J* = 6.7 Hz, 2H). **^{13}C-NMR (101 MHz, $CDCl_3$) δ_C** (ppm): 165.5 (^{13}C-enriched), 158.3, 148.5, 135.8, 134.0 (d, *J* = 1.6 Hz), 122.7 (d, *J* = 4.3 Hz), 117.4, 115.6 (d, *J* = 69.3 Hz), 111.9 (d, *J* = 3.2 Hz), 56.7, 40.4. **HRMS** (ESI-) $C_{10}{}^{13}CH_{11}O_3$ $[M\text{-}H]^-$; calculated 192.0747, found 192.0743.

methyl 6-(3-((3r,5r,7r)-adamantan-1-yl)-4-methoxyphenyl)-2-naphthoate (24)

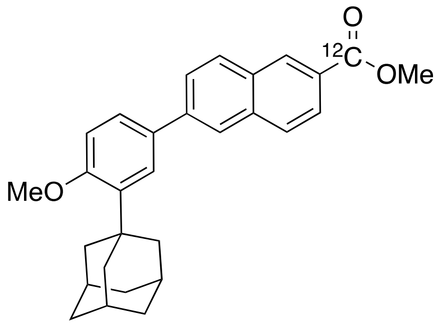

The title compound was prepared according to general procedure C (employing $Ba^{12}CO_3$, EtOH as the additive, 5 mol% of (BINAP)$PdCl_2$ catalyst, and a constant potential of -1.8 V (vs. sat. Ag/AgCl) for 85 C) employing 6-(3-((3r,5r,7r)-adamantan-1-yl)-4-methoxyphenyl)naphthalen-2-yl sulfurofluoridate (186.6 mg, 0.40 mmol) as starting material. Purification method III (10% EtOAc in Hept to 50% EtOAc in Hept) yielded the title compound as a white solid (91.4 mg, 0.21 mmol, 54%). F.E. = 49%.

^{1}H NMR (400 MHz, $CDCl_3$) δ_H (ppm): 8.61 (s, 1H), 8.07 (dd, *J* = 8.7, 1.7 Hz, 1H), 8.01 (s, 1H), 7.99 (d, *J* = 8.6 Hz, 1H), 7.92 (d, *J* = 8.7 Hz, 1H), 7.80 (dd, *J* = 8.6, 1.8 Hz, 1H), 7.60 (d, *J* = 2.4 Hz, 1H), 7.55 (dd, *J* = 8.4, 2.4 Hz, 1H), 7.00 (d, *J* = 8.4 Hz, 1H), 3.99 (s, 3H), 3.91 (s, 3H), 2.18 (s, 6H), 2.11 (s, 3H), 1.81 (s, 6H). **^{13}C NMR (101 MHz, $CDCl_3$) δ_C** (ppm): 167.5, 159.1, 141.6, 139.2, 136.1, 132.7, 131.4, 131.0, 129.9, 128.4, 127.1, 126.6, 126.1, 125.9, 125.7, 124.9, 112.3, 55.3, 52.4, 40.8, 37.4, 37.3, 29.3.

The spectral data is consistent with those reported in the literature:
Liu, Z., *et al. Org. Process Res. Dev.* **2006**, *10*, 285–288

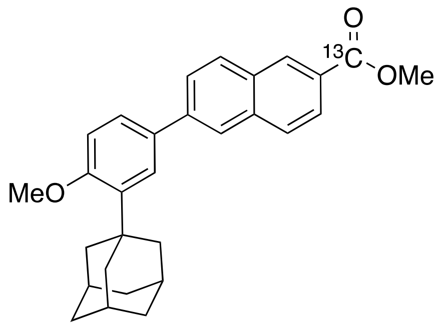

The title compound was prepared according to general procedure C (employing $Ba^{13}CO_3$, EtOH as the additive, 5 mol% of $(BINAP)PdCl_2$ catalyst, and a constant current of -2 mA for 85 C) employing 6-(3-((3r,5r,7r)-adamantan-1-yl)-4-methoxyphenyl)naphthalen-2-yl sulfurofluoridate (187.0 mg, 0.40 mmol) as starting material. Purification method III (10% EtOAc in Hept to 50% EtOAc in Hept) yielded the title compound as an off-white solid (86.0 mg, 0.20 mmol, 50%). F.E. = 45%.

^{1}H NMR (500 MHz, $CDCl_3$) δ_H (ppm): 8.61 (d, *J* = 3.0 Hz, 1H), 8.07 (ddd, *J* = 8.6, 3.4, 1.7 Hz, 1H), 8.01 (s, 1H), 7.99 (d, *J* = 8.5 Hz, 1H), 7.92 (d, *J* = 8.6 Hz, 1H), 7.80 (dd, *J* = 8.5, 1.8 Hz, 1H), 7.60 (d, *J* = 2.4 Hz, 1H), 7.55 (dd, *J* = 8.4, 2.4 Hz, 1H), 7.00 (d, *J* = 8.4 Hz, 1H), 3.99 (d, *J* = 3.8 Hz, 3H), 3.91 (s, 3H), 2.19 (d, *J* = 2.9 Hz, 6H), 2.11 (s, 3H), 1.81 (s, 6H). **^{13}C NMR (126 MHz, $CDCl_3$) δ_C** (ppm): 167.5 (^{13}C enriched), 159.1, 141.5, 139.2, 136.1, 132.7, 131.4 (d, J = 4.9), 131.0 (d, J = 2.4), 129.8, 128.4 (d, J = 4.4), 127.4, 126.6, 126.1, 125.9, 125.7 (d, J = 2.7), 124.9, 112.3, 55.3, 52.3 (d, J = 2.5), 40.8, 37.4, 37.3, 29.3.

4-(N,N-dipropylsulfamoyl)benzoic acid (25)

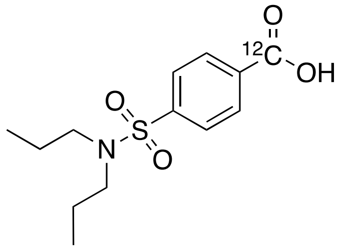

The title compound was prepared according to general procedure C (employing $Ba^{12}CO_3$, EtOH as the additive, and a constant potential of -1.8 V (vs. sat. Ag/AgCl) for 85 C) employing 4-bromo-*N*,*N*-dipropylbenzenesulfonamide (128.7 mg, 0.40 mmol) as starting material. Purification method I (Flash column chromatography 10-40% EtOAc in heptane, the eluent was acidified with 1% formic acid) yielded the title compound as a colorless solid (71.1 mg, 0.25 mmol, 62%). F.E. = 56%.

^{1}H-NMR (400 MHz, $CDCl_3$) δ_H (ppm): 8.23 (d, J = 8.7 Hz, 2H), 7.92 (d, J = 8.6 Hz, 2H), 3.16 – 3.08 (m, 4H), 1.56 (sext, J = 7.3 Hz, 4H), 0.88 (t, J = 7.4 Hz, 6H). **^{13}C-NMR (101 MHz, $CDCl_3$) δ_C** (ppm): 169.8, 145.4, 132.5, 131.0, 127.3, 50.1, 22.1, 11.3.

The spectral data is consistent with those reported in literature:
Chung, S., *et al. Tetrahedron Lett.*, **2019**, *60*, 792–795.

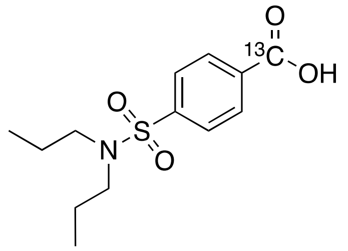

The title compound was prepared according to general procedure C (employing $Ba^{13}CO_3$, EtOH as the additive, and a constant potential of -1.8 V (vs. Ag/AgCl) for 85 C) employing 4-bromo-*N*,*N*-dipropylbenzenesulfonamide (127.6 mg, 0.40 mmol) as starting material. Purification method I (Flash column chromatography 10-40% EtOAc in heptane, eluent was acidified with 1% formic acid) yielded the title compound as a white solid (64.7 mg, 0.23 mmol, 57%). F.E. = 52%.

^{1}H-NMR (400 MHz, $CDCl_3$) δ_H (ppm): 8.24 (dd, J = 8.7, 4.2 Hz, 2H), 7.92 (d, J = 7.9 Hz, 2H), 3.21 – 3.03 (m, 4H), 1.56 (sext, J = 7.4 Hz, 4H), 0.88 (t, J = 7.4 Hz, 6H). **^{13}C-NMR (101 MHz, $CDCl_3$) δ_C** (ppm): 170.6 (^{13}C-enriched), 145.3, 132.6 (d, J = 72.6 Hz), 131.0 (d, J = 2.7 Hz), 127.3 (d, J = 4.5 Hz), 50.1, 22.1, 11.3. HRMS (ESI-) calc. for $C_{12}{}^{13}CH_{18}NO_4S$ $[M-H]^-$: 285.0996, found 285.0989.

2,5,7,8-tetramethyl-2-(4,8,12-trimethyltridecyl)chromane-6-carboxylic acid (26)

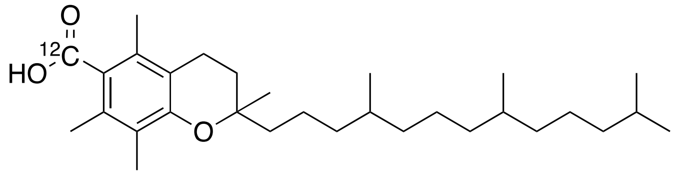

The title compound was prepared according to general procedure B (with a constant potential of -1.7 V vs. Ag/AgCl for 85 C) employing (R)-2,5,7,8-tetramethyl-2-((4R,8R)-4,8,12-trimethyltridecyl)chroman-6-yl fluorosulfate (205.2 mg, 0.40 mmol) as starting material. Purification method II yielded the title compound as a white solid (57.8 mg, 0.13 mmol, 32%). F.E. = 29%.

^{1}H NMR (400 MHz, $CDCl_3$) δ_H (ppm): 2.61 (t, J = 6.8 Hz, 2H), 2.28 (s, 3H), 2.25 (s, 3H), 2.10 (s, 3H), 1.89 – 1.73 (m, 2H), 1.63 – 1.47 (m, 3H), 1.45 – 1.34 (m, 4H), 1.32 - 1.20 (m, 11H), 1.17 – 1.04 (m, 6H), 0.90 – 0.81 (m, 12H).

^{13}C NMR (101 MHz, $CDCl_3$) (mixture of diastereoisomers) **δ_C** (ppm): 176.3, 152.8, 131.7, 130.8, 125.3, 123.0, 117.4, 75.7, 40.1, 40.0, 39.5, 37.7, 37.7, 37.6, 37.6, 37.5, 37.5, 37.4, 32.9, 32.9, 32.9, 32.9, 31.2, 31.2, 28.1, 25.0, 25.0, 24.6, 24.1, 22.9, 22.8, 21.2, 20.5, 19.9, 19.8, 19.8, 19.7, 17.3, 16.4, 11.7.

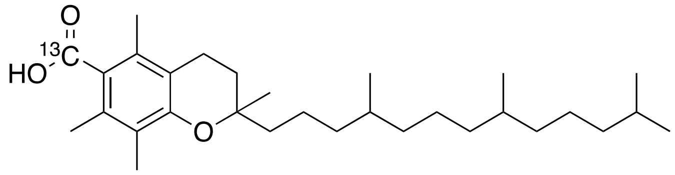

The title compound was prepared according to general procedure C (employing $Ba^{13}CO_3$, EtOH as the additive, and a constant potential of -1.7 V vs. Ag/AgCl for 89 C) employing (R)-2,5,7,8-tetramethyl-2-((4R,8R)-4,8,12-trimethyltridecyl)chroman-6-yl fluorosulfate (214.8 mg, 0.42 mmol) as starting material. Purification method I (Flash column chromatography 10-40% EtOAc in heptane, eluent was acidified with 1% formic acid) yielded the title compound as an off-white waxy solid (63.4 mg, 0.14 mmol, 33%).

^{1}H NMR (400 MHz, $CDCl_3$) δ_H (ppm): 2.61 (t, J = 6.8 Hz, 2H), 2.29 (s, 3H), 2.26 (s, 3H), 2.11 (s, 3H), 1.93 – 1.69 (m, 2H), 1.63 – 1.47 (m, 3H), 1.45 – 1.34 (m, 4H), 1.32 - 1.20 (m, 11H), 1.18 – 1.04 (m, 6H), 0.96 – 0.79 (m, 12H).
^{13}C NMR (101 MHz, $CDCl_3$) (mixture of diastereoisomers) **δ_C** (ppm): 176.9 (^{13}C enriched), 152.8, 131.7 (d, J = 2.6 Hz), 130.7 (d, J = 2.6 Hz), 125.4 (d, J = 73.0 Hz), 123.0 (d, J = 4.8 Hz), 117.4 (d, J = 4.8 Hz), 75.7, 40.1, 40.0, 39.5, 37.7, 37.6, 37.6, 37.6, 37.5, 37.5, 37.4, 32.9, 32.9, 32.9, 32.8, 31.2, 31.2, 28.1, 25.0, 25.0, 24.6, 24.1, 22.9, 22.8, 21.2, 20.5, 19.9, 19.8, 19.8, 19.7, 17.3, 17.3, 16.4, 16.4, 11.7. **HRMS** (ESI-) calc. for $C_{29}{}^{13}CH_{49}O_3$ $[M-H]^-$: 458.3721, found 458.3722

3,4,5-trimethoxybenzoic acid (27)

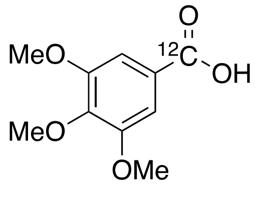

The title compound was prepared according to general procedure A (with a constant current of -4 mA for 85 C) employing 5-bromo-1,2,3-trimethoxybenzene (98.8 mg, 0.40 mmol) as starting material. Purification method II yielded the title compound as an off-white crystalline solid (72.0 mg, 0.34 mmol, 85%). F.E. = 77%

^{1}H NMR (400 MHz, $CDCl_3$) δ_H (ppm): 7.38 (s, 2H), 3.93 (s, 3H), 3.93 (s, 6H). **^{13}C NMR (101 MHz, $CDCl_3$) δ_C** (ppm): 171.9, 153.1, 143.1, 124.2, 107.5, 61.1, 56.4.

The spectral data is consistent with those reported in the literature:
Sun, C., *et al. Green Chem.*, **2020**, *22*, 3489-3494

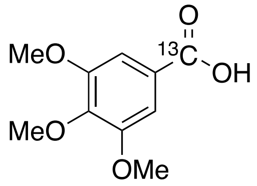

The title compound was prepared according to general procedure C (employing $Ba^{13}CO_3$, ascorbic acid as the electron donor, and a constant current of -4 mA for 85 C) employing 5-bromo-1,2,3-trimethoxybenzene (98.8 mg, 0.40 mmol) as starting material. Purification method I (Flash chromatography column pentane to 40% acetone in pentane eluent was acidified with 2% formic acid) yielded the title compound as an off-white crystalline solid (75.9 mg, 0.36 mmol, 89%). F.E. = 81%

^{1}H NMR (400 MHz, $CDCl_3$) δ_H (ppm): 7.38 (d, J = 4.6 Hz, 2H), 3.94 (s, 3H), 3.93 (s, 6H). **^{13}C NMR (101 MHz, $CDCl_3$) δ_C** (ppm): 171.7 (^{13}C-enriched), 153.1 (d, J = 6.6 Hz), 143.1, 124.2 (d, J = 73.7 Hz), 107.5 (d, J = 2.9 Hz), 61.1, 56.4. **HRMS** (ESI-) $C_9{}^{13}CH_{11}O_5$ $[M-H]^-$; calculated 212.0646, found 212.0643.

benzo[d][1,3]dioxole-5-carboxylic acid (28)

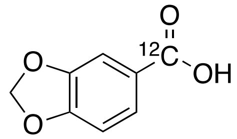

The title compound was prepared according to general procedure B (with a constant current of -4 mA for 88.5 C) employing benzo[d][1,3]dioxol-5-yl sulfurofluoridate (91.8 mg, 0.42 mmol) as starting material. Purification method II yielded the title compound as an off-white crystalline solid (64.0 mg, 0.39 mmol, 94%). F.E. = 85%.

The title compound was prepared according to general procedure B (with a constant current of -4 mA for 86.2 C) employing benzo[d][1,3]dioxol-5-yl sulfurofluoridate (89.4 mg, 0.41 mmol) as starting material. Purification method II yielded the title compound as an off-white crystalline solid (61.8 mg, 0.37 mmol, 91%). F.E. = 83%.

The title compound was prepared according to general procedure D employing benzo[d][1,3]dioxol-5-yl sulfurofluoridate (88.0 mg, 0.40 mmol) as starting material.

Purification method II yielded the title compound as an off-white crystalline solid (46.9 mg, 0.28 mmol, 71%). F.E. = 64%.

The title compound was prepared according to general procedure D employing benzo[d][1,3]dioxol-5-yl sulfurofluoridate (88.0 mg, 0.40 mmol) as starting material. Purification method II yielded the title compound as an off-white crystalline solid (43.5 mg, 0.26 mmol, 66%). F.E. = 60%.

^{1}H NMR (400 MHz, Acetone-d$_6$) δ_H (ppm): 7.65 (dd, J = 8.1, 1.8 Hz, 1H), 7.41 (d, J = 1.8 Hz, 1H), 6.94 (d, J = 8.1 Hz, 1H), 6.11 (s, 2H). **^{13}C NMR (101 MHz, Acetone-d$_6$) δ_C** (ppm): 166.9, 152.6, 148.8, 126.1, 125.4, 109.9, 108.7, 103.0.

The spectral data is consistent with those reported in the literature:
Zhang, Z., *et al. Angew. Chem. Int. Ed.* **2019**, *58*, 14110–14114

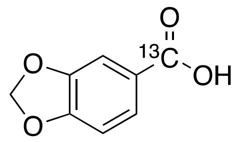

The title compound was prepared according to general procedure C (employing Ba^{13}CO$_3$, EtOH as additive, and a constant current of -4 mA for 86 C) employing benzo[d][1,3]dioxol-5-yl sulfurofluoridate (89 mg, 0.41 mmol) as starting material. Purification method II yielded the title compound as an off-white crystalline solid (62.1 mg, 0.37 mmol, 93%). F.E. = 84%.

^{1}H NMR (500 MHz, DMSO, 25°C) δ_H (ppm): 6.12 (s, 2H), 6.99 (dd, J = 8.2, 0.8 Hz, 1H), 7.36 (dd, J = 3.9, 1.7 Hz, 1H), 7.54 (ddd, J = 8.1, 4.2, 1.7 Hz, 1H). **^{13}C NMR (126 MHz, DMSO, 25°C) δ_C** (ppm): 166.6 (13C enriched), 151.14, 147.5 (J = 6.4), 125.0 (J = 3.1), 124.4, 108.8 (J = 3.1), 108.1 (J = 5.4), 101.9. **HRMS** (ESI-) calc. for $C_7{}^{13}H_5O_4$ [M-H]$^-$: 166.0227, found 166.0212.

4-(1-(3,5,5,8,8-pentamethyl-5,6,7,8-tetrahydronaphthalen-2-yl)vinyl)benzoic acid (29)

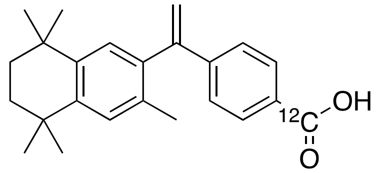

The title compound was prepared according to general procedure C (employing Ba^{12}CO$_3$, a constant current of -4 mA for 85 C and 5 mol% of (BINAP)PdCl$_2$) employing 4-(1-(3,5,5,8,8-pentamethyl-5,6,7,8-tetrahydronaphthalen-2-yl)vinyl)phenyl sulfurofluoridate

(161 mg, 0.40 mmol) as starting material. Purification method V (eluent 35% ACN in pH 10 H_2O solution (NH_4OH) until 55% ACN in pH 10 H_2O solution) yielded the title compound as a white solid (37 mg, 0.11 mmol, 27%). F.E. = 25%.

^{1}H NMR (500 MHz, $CDCl_3$) δ_H (ppm): 1.29 (s, 6H), 1.32 (s, 6H), 1.71 (s, 4H), 1.96 (s, 3H), 5.36 (s, 1H), 5.84 (s, 1H), 7.09 (s, 1H), 7.14 (s, 1H), 7.39 (d, J = 8.1 Hz, 2H), 8.04 (d, J = 8.1 Hz, 2H). **^{13}C NMR (126 MHz, $CDCl_3$) δ** (ppm): 172.2, 149.3, 146.6, 144.6, 142.5, 138.1, 132.9, 130.5, 128.2, 126.8, 117.3, 35.4, 35.3, 34.2, 34.0, 32.1, 32.0, 20.1.

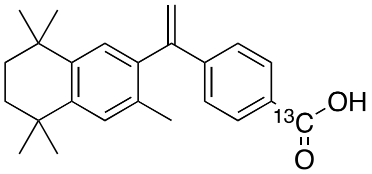

The title compound was prepared according to general procedure C (employing $Ba^{13}CO_3$, EtOH as additive, a constant current of -4 mA for 85 C and 5 mol% of (BINAP)$PdCl_2$) employing 4-(1-(3,5,5,8,8-pentamethyl-5,6,7,8-tetrahydronaphthalen-2-yl)vinyl)phenyl sulfurofluoridate (161 mg, 0.40 mmol) as starting material. Purification method V (gradient from 35% ACN in pH 10 H_2O solution (NH_4OH) until 55% ACN in pH 10 H_2O solution) yielded the title compound as a white solid (39.2 mg, 0.11 mmol, 28%). F.E. = 25%.

^{1}H NMR (500 MHz, $CDCl_3$) δ_H (ppm): 1.28 (s, 6H), 1.31 (s, 6H), 1.71 (s, 4H), 1.95 (s, 3H), 5.35 (d, J = 1.3 Hz, 1H), 5.84 (d, J = 1.3 Hz, 1H), 7.09 (s, 1H), 7.13 (s, 1H), 7.38 (d, J = 7.8 Hz, 2H), 8.03 (dd, J = 8.5, 3.9 Hz, 2H). **^{13}C NMR (126 MHz, $CDCl_3$) δ_C** (ppm): 171.8 (^{13}C enriched), 149.3, 146.6, 144.6, 142.5, 138.1, 132.9, 130.4 (d, *J* = 2.8), 128.2, 126.8 (d, *J* = 4.5), 117.3, 35.4, 35.3, 34.2, 34.1, 32.1, 32.0, 20.1. **HRMS** (ESI-) calc. for $C_{23}{}^{13}CH_{27}O_2$ $[M-H]^-$: 348.2050, found 348.2036.

2-methoxy-4-propylbenzoic acid (2)

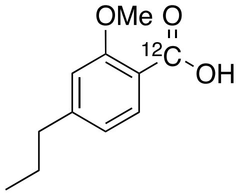

The title compound was prepared according to general procedure B (with a constant current of -4 mA for 21549 seconds) employing 2-methoxy-4-propylphenyl sulfurofluoridate (100.8 mg, 0.41 mmol) as starting material. Purification method II yielded the title compound as an off-white crystalline solid (66.6 mg, 0.34 mmol, 84%). F.E. = 78%.

The title compound was prepared according to general procedure D employing 2-methoxy-4-propylphenyl sulfurofluoridate (99.0 mg, 0.40 mmol) as starting material. Purification method II yielded the title compound as an off-white crystalline solid (66.4 mg, 0.34 mmol, 85%). F.E. = 77%.

The title compound was prepared according to general procedure D employing 2-methoxy-4-propylphenyl sulfurofluoridate (99.0 mg, 0.40 mmol) as starting material. Purification method II yielded the title compound as an off-white crystalline solid (65.1 mg, 0.34 mmol, 84%). F.E. = 77%.

^{1}H NMR (500 MHz, $CDCl_3$) δ_H (ppm): 10.83 (br, 1H), 8.03 (d, *J* = 8.0 Hz, 1H), 6.92 (d, *J* = 8.1 Hz, 1H), 6.84 (s, 1H), 4.04 (s, 3H), 2.62 (t, *J* = 8.0 Hz, 2H), 1.65 (h, *J* = 7.4 Hz, 2H), 0.93 (t, *J* = 7.4 Hz, 3H). **^{13}C NMR (126 MHz, $CDCl_3$) δ_C** (ppm): 165.8, 158.2, 151.3, 133.6, 122.4, 115.1, 111.8, 56.6, 38.3, 24.2, 13.8.

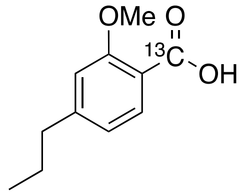

The title compound was prepared according to general procedure C (employing $Ba^{13}CO_3$, EtOH as the additive, and a constant current of -4 mA for 85 C) employing 2-methoxy-4-propylphenyl sulfurofluoridate (99 mg, 0.40 mmol) as starting material. Purification method V (gradient from 10% ACN in pH 10 H_2O solution (NH_4OH) to 35% ACN in pH 10 H_2O solution) yielded the title compound as a white solid (57.6 mg, 0.30 mmol, 75%). F.E. = 68%.

^{1}H NMR (500 MHz, $CDCl_3$) δ_H (ppm): 8.07 (dd, *J* = 8.0, 4.5 Hz, 1H), 6.94 (d, *J* = 8.1 Hz, 1H), 6.84 (s, 1H), 4.06 (s, 3H), 2.63 (t, *J* = 8.0 Hz, 2H), 1.67 (h, *J* = 7.4 Hz, 2H), 0.95 (t, *J* = 7.3 Hz, 3H). **^{13}C NMR (126 MHz, $CDCl_3$) δ_C** (ppm): 165.6 (13c enriched), 158.2, 151.3, 133.8, 122.6 (d, *J* = 4.4), 115.2 (d, *J* = 69.3), 111.8 (*J* = 3.1), 77.2, 56.7, 38.4, 24.2, 13.9.

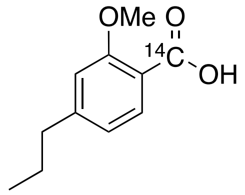

The title compound was prepared according to general procedure C (employing a mixture of $Ba^{12}CO_3$ and $Ba^{14}CO_3$ (5:1), EtOH as additive, and a constant current of -4 mA for 85 C) employing 2-methoxy-4-propylphenyl sulfurofluoridate (99 mg, 0.40 mmol) as

starting material. Purification method III yielded the title compound as a white solid (60.8 mg, 0.31 mmol, 77%) (100 MBq, SA: 355 GBq/mol, 47% RCY, >99% RCP). F.E. = 70%.

^{1}H NMR (500 MHz, CDCl$_3$) δ$_H$ (ppm): 8.06 (dd, *J* = 7.9, 2.1 Hz, 1H), 6.94 (dd, *J* = 8.0, 1.5 Hz, 1H), 6.84 (s, 1H), 4.06 (d, *J* = 1.1 Hz, 3H), 2.63 (t, *J* = 7.6 Hz, 2H), 1.66 (h, *J* = 7.4 Hz, 2H), 0.95 (t, *J* = 7.3 Hz, 3H). **^{13}C NMR (126 MHz, CDCl$_3$) δ$_C$** (ppm): 165.7, 158.2, 151.3, 133.8, 122.5, 115.2, 111.8, 56.7, 38.4, 24.2, 13.9. **HRMS** (ESI+) calc. for $C_{10}{}^{14}CH_{15}O_3$ $[M+H]^+$: 197.1048 , found 197.1055.

All reported radiochemical yields were computed using the specific activity of the purchased $Ba^{14}CO_3$ (2123.8 MBq mmol^{-1}) and the amount of $Ba^{14}CO_3$ used for the gas release. In this case, it would lead to 0.1 mmol of $Ba^{14}CO_3$ with a total activity of 212.4 MBq, leading to 47% RCY.

4-((5,5,8,8-tetramethyl-5,6,7,8-tetrahydronaphthalen-2-yl)carbamoyl)benzoic acid (30)

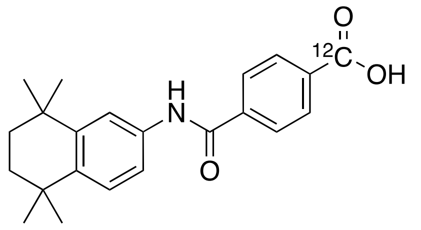

The title compound was prepared according to general procedure C (employing $Ba^{12}CO_3$, EtOH as additive, and a constant current of -4 mA for 85 C) employing 4-bromo-N-(5,5,8,8-tetramethyl-5,6,7,8-tetrahydronaphthalen-2-yl)benzamide (155.5 mg, 0.40 mmol) as starting material. Purification method III (10% EtOAc in Hept to 30% EtOAc in Hept, the eluent was acidified with formic acid 0.1%) yielded the title compound as a white solid (112.4 mg, 0.32 mmol, 80%). F.E. = 73%.

^{1}H NMR (400 MHz, DMSO-d$_6$) δ$_H$ (ppm): 13.27 (br, 1H), 10.25 (s, 1H), 8.10 – 8.01 (m, 4H), 7.68 (d, *J* = 2.3 Hz, 1H), 7.58 (dd, *J* = 8.6, 2.3 Hz, 1H), 7.29 (d, *J* = 8.6 Hz, 1H), 1.64 (s, 4H), 1.25 (s, 6H), 1.23 (s, 6H). **^{13}C NMR (101 MHz, DMSO-d$_6$) δ$_C$** (ppm): 166.8, 164.5, 144.5, 140.1, 138.8, 136.4, 133.2, 129.2, 127.8, 126.5, 118.3, 118.1, 34.6, 34.5, 34.0, 33.6, 31.7, 31.6.

The spectral data is consistent with those reported in the literature:
Zuo, D., *et. al., Angew. Chem. Int. Ed.* **2022**, *61*, e202202794

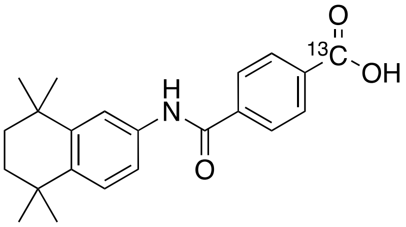

The title compound was prepared according to general procedure C (employing $Ba^{13}CO_3$, EtOH as additive, and a constant current of -4 mA for 85 C) employing 4-bromo-N-(5,5,8,8-tetramethyl-5,6,7,8-tetrahydronaphthalen-2-yl)benzamide (155.5 mg, 0.40 mmol) as starting material. Purification method V (gradient from 10% ACN in pH 3 H_2O solution (TFA) to 80% ACN in pH 3 H_2O solution) yielded the title compound as a white solid (92.3 mg, 0.26 mmol, 66%). F.E. = 60%.

^{1}H NMR (500 MHz, CD_3CN) δ_H (ppm): 8.75 (s, 1H), 8.08–8.14 (m, 2H), 7.99 (d, *J* = 8.0 Hz, 2H), 7.61 (d, *J* = 2.3 Hz, 1H), 7.50 (dd, *J* = 8.5, 2.3 Hz, 1H), 7.33 (d, *J* = 8.5 Hz, 1H), 1.70 (s, 4H), 1.28 (s, 6H), 1.27 (s, 6H). **^{13}C NMR (126 MHz, CD_3CN) δ_C** (ppm): 167.3 (13C enriched), 165.8, 146.4, 142.2, 140.3, 137.1, 134.0, 133.4, 130.7 (d, *J* = 2.6), 128.5 (d, *J* = 4.5), 127.9, 119.7 (d, *J* = 13.1), 35.7, 35.7, 35.1, 34.7, 32.1, 32.0. HRMS (ESI+) calc. for $C_{21}{}^{13}CH_{26}NO_3$ $[M+H]^+$: 353.1941, found 353.1946.

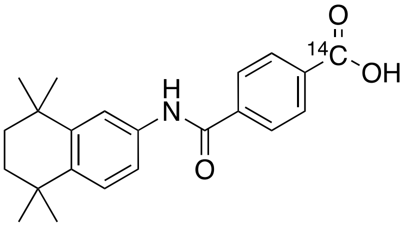

The title compound was prepared according to general procedure C (employing a 5:1 dilution of $Ba^{12}CO_3$: $Ba^{14}CO_3$, EtOH as additive, and a constant current of -4 mA for 85 C) employing 4-bromo-N-(5,5,8,8-tetramethyl-5,6,7,8-tetrahydronaphthalen-2-yl)benzamide (155.5 mg, 0.40 mmol) as starting material. Purification method V (gradient from 10% ACN in pH 3 H_2O solution (TFA) to 80% ACN in pH 3 H_2O solution) yielded the title compound as a white solid (89 mg, 0.25 mmol, 63%) (80 MBq, SA: 326 GBq/mol, 38% RCY, >99% RCP). F.E. = 57%.

^{1}H NMR (500 MHz, CD_3CN) δ_H (ppm): 8.73 (s,1H), 8.09–8.13 (m, 2H), 7.97–8.02 (m, 2H), 7.61 (d, *J* = 2.3 Hz, 1H), 7.50 (dd, *J* = 8.6, 2.3 Hz, 1H), 7.34 (d, *J* = 8.5 Hz, 1H), 1.71 (d, *J* = 1.1 Hz, 4H), 1.28 (s, 6H), 1.27 (s, 7H). **^{13}C NMR (126 MHz, CD_3CN) δ_C** (ppm): 167.1, 165.7, 146.4, 142.2, 140.3, 137.1, 133.6, 130.7, 128.5, 127.9, 119.7, 119.6, 35.7, 35.7, 35.1, 34.7, 32.1, 32.0. HRMS (ESI+) calc. for $C_{21}{}^{14}CH_{26}NO_3$ $[M+H]^+$: 355.1940, found 354.1951.

2-fluoro-5-((4-oxo-3,4-dihydrophthalazin-1-yl)methyl)benzoic acid (31)

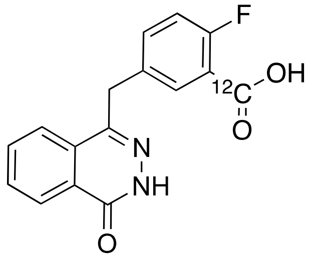

The title compound was prepared according to general procedure C (employing Ba^{12}CO$_3$, EtOH as additive, 5 mol% of (BINAP)PdCl$_2$ catalyst and a constant current of -4 mA for 85 C) employing 4-(3-bromo-4-fluorobenzyl)phthalazin-1(2H)-one (133.3 mg, 0.40 mmol) as starting material. Purification method I (10% EtOAc in Hept to 99.9% EtOAc, the eluent was acidified with formic acid 0.1%) yielded the title compound as a white solid (89.8 mg, 0.30 mmol, 75%). F.E. = 68%.

^{1}H NMR (400 MHz, DMSO-d$_6$) δ$_H$ (ppm): 13.24 (s, 1H), 12.59 (s, 1H), 8.26 (dd, *J* = 7.9, 1.5 Hz, 1H), 7.98 (d, *J* = 8.1 Hz, 1H), 7.90 (td, *J* = 7.6, 1.5 Hz, 1H), 7.87 – 7.79 (m, 2H), 7.62 – 7.54 (m, 1H), 7.23 (dd, *J* = 10.8, 8.5 Hz, 1H), 4.35 (s, 2H). **^{13}C NMR (101 MHz, DMSO-d$_6$) δ$_C$** (ppm): 165.0 (d, *J* = 2.9 Hz), 159.9 (d, *J* = 255.7 Hz), 159.3, 144.9, 134.9 (d, *J* = 9.2 Hz), 134.3 (d, *J* = 3.7 Hz), 133.6, 131.8, 131.6, 129.1, 127.9, 126.1, 125.5, 119.1 (d, *J* = 10.6 Hz), 117.0 (d, *J* = 22.4 Hz), 36.3. **^{19}F NMR (376 MHz, DMSO-d$_6$) δ$_F$** (ppm): -114.0

The spectral data is consistent with those reported in the literature:
Zmuda, F., *et.al. J. Med. Chem.* **2015**, *58*, 8683–8693

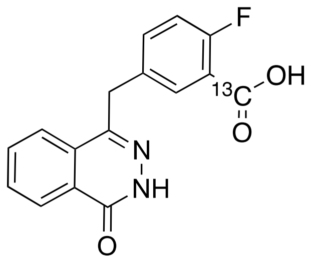

The title compound was prepared according to general procedure C (employing Ba^{13}CO$_3$, EtOH as additive, 5 mol% of (BINAP)PdCl$_2$ catalyst and a constant current of -4 mA for

85 C) employing 4-(3-bromo-4-fluorobenzyl)phthalazin-1(2H)-one (133.3 mg, 0.40 mmol) as starting material. Purification method V (gradient from 38% ACN in pH 3 H_2O solution (TFA) to 58% ACN in pH 3 H_2O solution) yielded the title compound as a white solid (89.1 mg, 0.30 mmol, 74%). F.E. = 67%.

^{1}H NMR (500 MHz, DMSO-d$_6$) δ$_H$ (ppm): 13.24 (s, 1H), 12.59 (s, 1H), 8.26 (dd, *J* = 7.9, 1.4 Hz, 1H), 7.98 (d, *J* = 8.0 Hz, 1H), 7.90 (td, *J* = 7.5, 1.4 Hz, 1H), 7.79–7.86 (m, 2H), 7.57 (ddd, *J* = 8.5, 4.6, 2.5 Hz, 1H), 7.23 (ddd, *J* = 10.5, 8.5, 1.7 Hz, 1H), 4.35 (s, 2H). **^{13}C NMR (126 MHz, DMSO-d$_6$) δ$_C$** (ppm): 165.2 (d, *J* = 2.8) (13C enriched), 161.1, 159.7, 159.1, 145.3, 135.1 (d, *J* = 8.8), 134.5 (t, *J* = 4.0), 133.8, 131.9 (d, *J* = 16.1), 129.2, 128.0, 126.3, 125.6, 119.3 (dd, *J* = 73.0, 10.6), 117.3 (d, *J* = 2.7), 36.5. **^{19}F NMR (473 MHz, DMSO-d$_6$) δ$_H$** (ppm): -114.0 (d, *J* = 4.3). **HRMS** (ESI+) calc. for $C_{15}{}^{13}CH_{12}FN_2O_3$ $[M+H]^+$: 300.0860, found 300.0864.

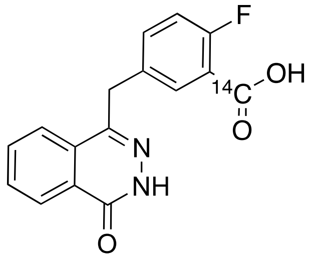

The title compound was prepared according to general procedure C (employing $Ba^{14}CO_3$, EtOH as additive, 5 mol% of (BINAP)$PdCl_2$ catalyst and a constant current of -4 mA for 85 C) employing 4-(3-bromo-4-fluorobenzyl)phthalazin-1(2H)-one (133.3 mg, 0.40 mmol) as starting material. Purification method V (gradient from 38% ACN in pH 3 H_2O solution (TFA) to 58% ACN in pH 3 H_2O solution) (88% of the crude material was purified) yielded the title compound as a white solid (70.7 mg, 0.24 mmol, 67%) (465 MBq, SA: 1937 GBq/mol, 37% RCY, >99% RCP). F.E. = 61%.

^{1}H NMR (500 MHz, DMSO) δ$_H$ (ppm): 13.23 (s, 1H), 12.59 (s, 1H), 8.26 (dd, J = 7.9, 1.4 Hz, 1H), 7.97 (d, J = 7.7 Hz, 1H), 7.89 (ddd, J = 8.1, 7.2, 1.4 Hz, 1H), 7.8–7.84 (m, 2H), 7.57 (ddd, J = 8.5, 4.6, 2.5 Hz, 1H), 7.23 (dd, J = 10.8, 8.5 Hz, 1H), 4.35 (s, 2H). **^{13}C NMR (126 MHz, DMSO-d$_6$) δ$_C$** (ppm): 165.00 (d, *J* = 3.0), 159.90 (d, *J* = 255.8), 159.4, 144.9, 134.9 (d, *J* = 8.8), 134.3 (d, *J* = 3.7), 133.6, 131.9, 131.6, 129.1, 127.9, 126.1, 125.5, 119.1 (d, *J* = 10.6), 117.0 (d, *J* = 22.5), 36.3. **^{19}F NMR (473 MHz, DMSO-d$_6$) δ$_H$** (ppm): -114.0. **HRMS** (ESI+) calc. for $C_{15}{}^{14}CH_{12}FN_2O_3$ $[M+H]^+$: 301.0859, found 301.0865.

1-naphthoic acid (33)

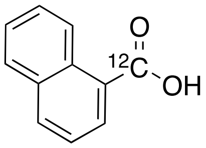

The title compound was prepared according to general procedure D employing 1-bromonaphthalene (83 mg, 0.40 mmol) as starting material. Purification method II yielded the title compound as a white solid (59.7mg, 0.35 mmol, 87%). F.E. = 79%.

The title compound was prepared according to general procedure D employing 1-bromonaphthalene (83 mg, 0.40 mmol) as starting material. Purification method II yielded the title compound as a white solid (57.6 mg, 0.33 mmol, 84%). F.E. = 76%.

^{1}H-NMR (500 MHz, $CDCl_3$) δ_H (ppm): 9.12 (dd, J = 8.7, 1.0 Hz, 1H), 8.44 (dd, J = 7.3, 1.3 Hz, 1H), 8.11 (d, J = 8.2 Hz, 1H), 7.91–7.95 (m, 1H), 7.68 (ddd, J = 8.6, 6.8, 1.4 Hz, 1H), 7.56–7.6 (m, 2H). **^{13}C-NMR (126 MHz, $CDCl_3$) δ_C** (ppm): 173.6, 134.8, 134.1, 132.1, 131.8, 128.9, 128.3, 126.5, 126.1, 125.7, 124.7.

The spectral data is consistent with those reported in literature:
A. Correa, *et al. J. Am. Chem. Soc.* **2009**, *131*, 15974–15975.

7 NMR Spectra

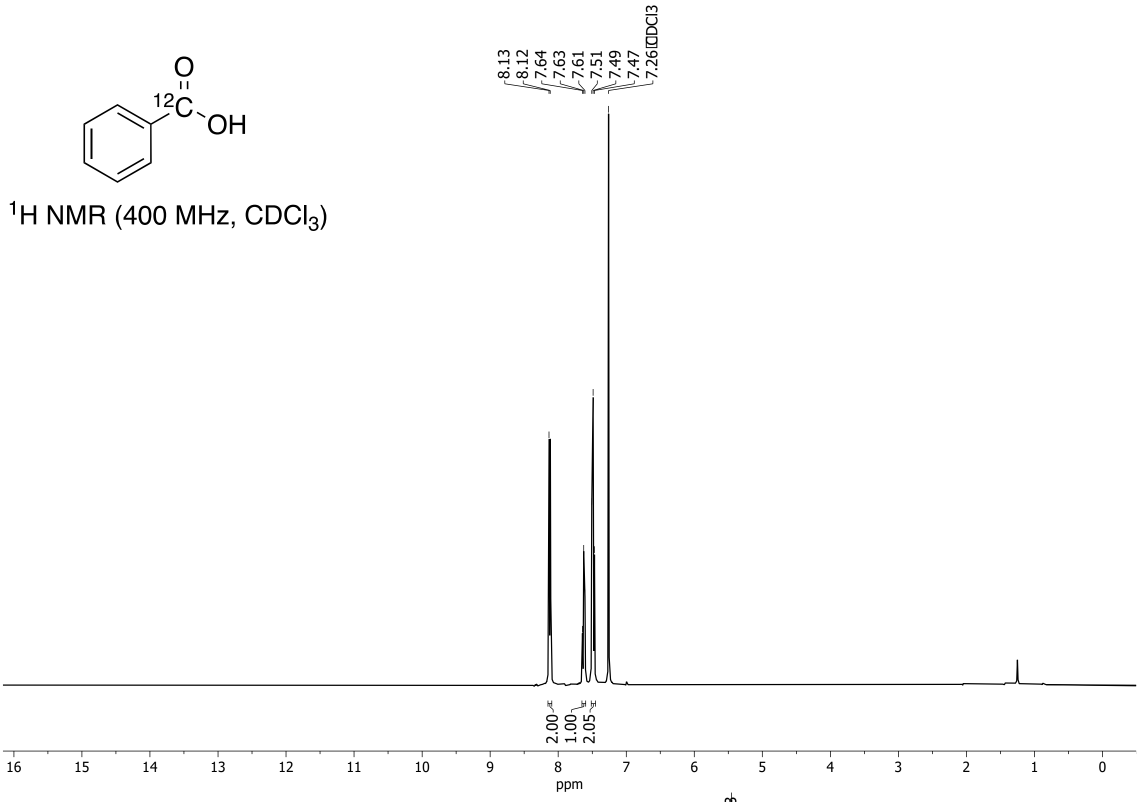

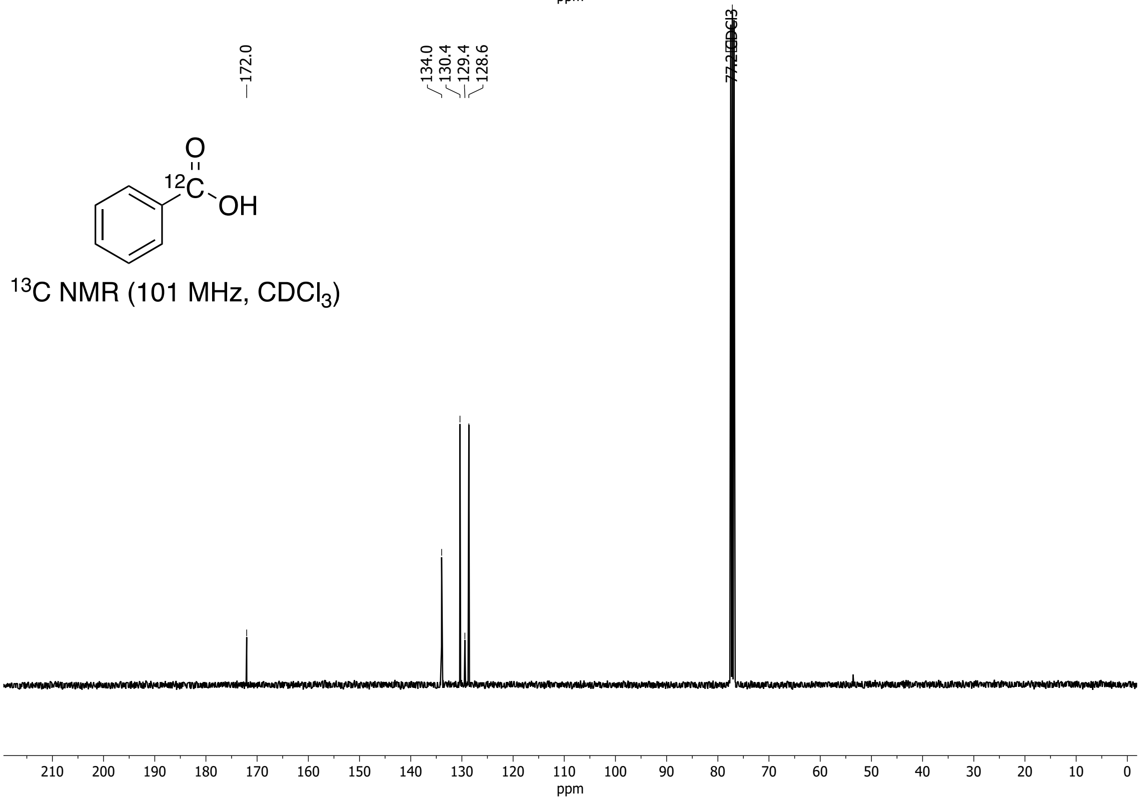

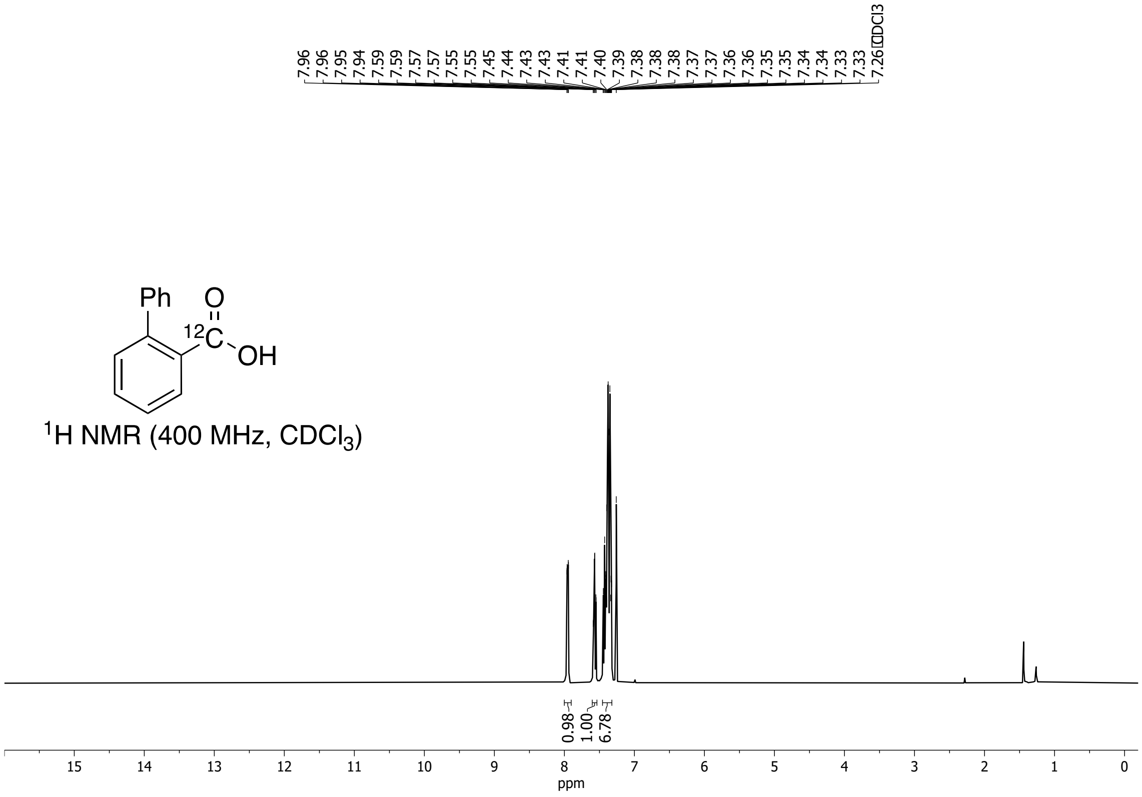
7.96
7.96
7.95
7.94
7.59
7.59
7.57
7.57
7.55
7.55
7.45
7.44
7.43
7.43
7.41
7.41
7.40
7.39
7.38
7.38
7.37
7.37
7.36
7.35
7.35
7.34
7.34
7.33
7.33
7.26 CDCl3
Ph
O
12C
OH
1H NMR (400 MHz, CDCl3)
0.98
1.00
6.78
ppm

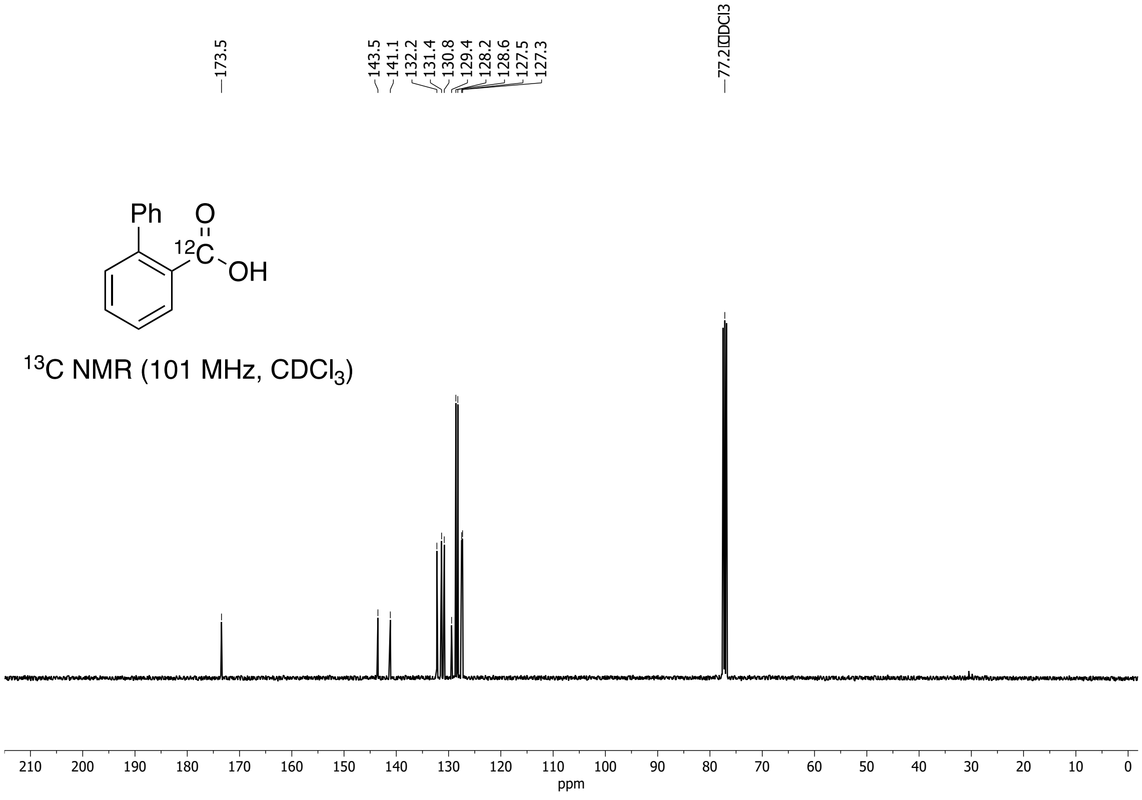
173.5
143.5
141.1
132.2
131.4
130.8
129.4
128.2
128.6
127.5
127.3
77.2 CDCl3
Ph
O
12C
OH
13C NMR (101 MHz, CDCl3)
ppm

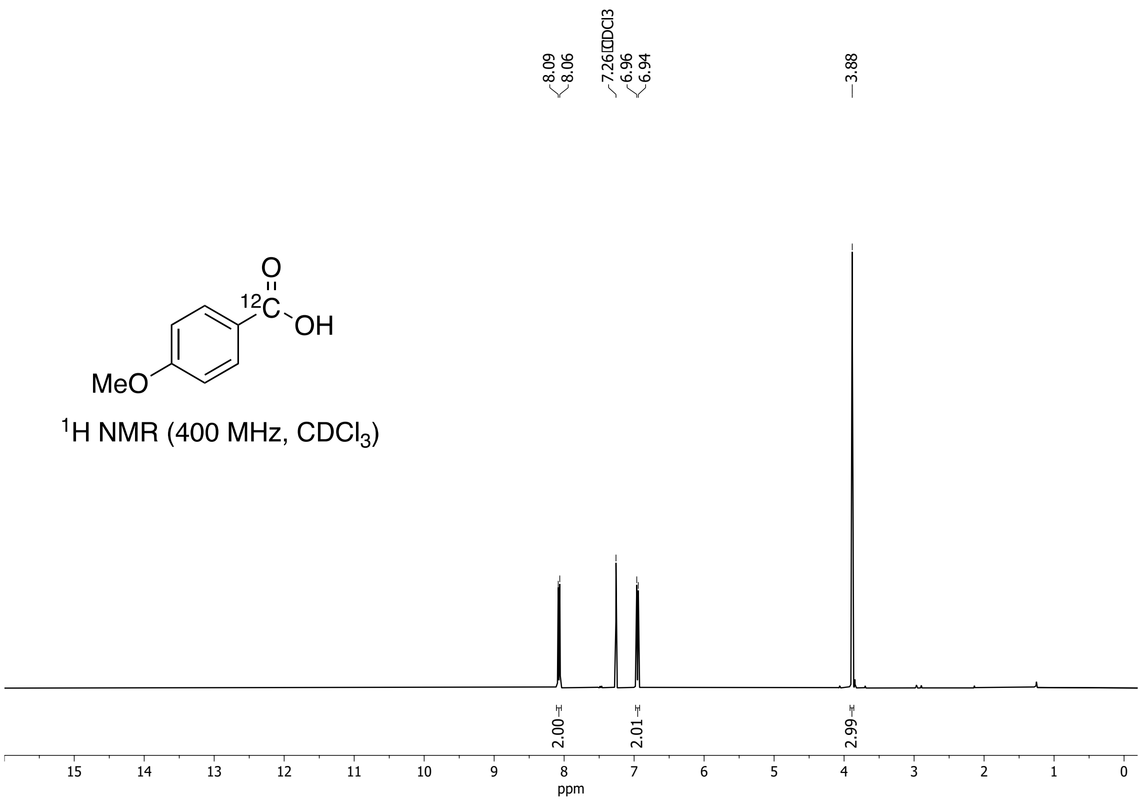
8.09
8.06
7.26 CDCl3
6.96
6.94
3.88
O
12C
OH
MeO
1H NMR (400 MHz, CDCl3)
2.00
2.01
2.99
15 14 13 12 11 10 9 8 7 6 5 4 3 2 1 0
ppm

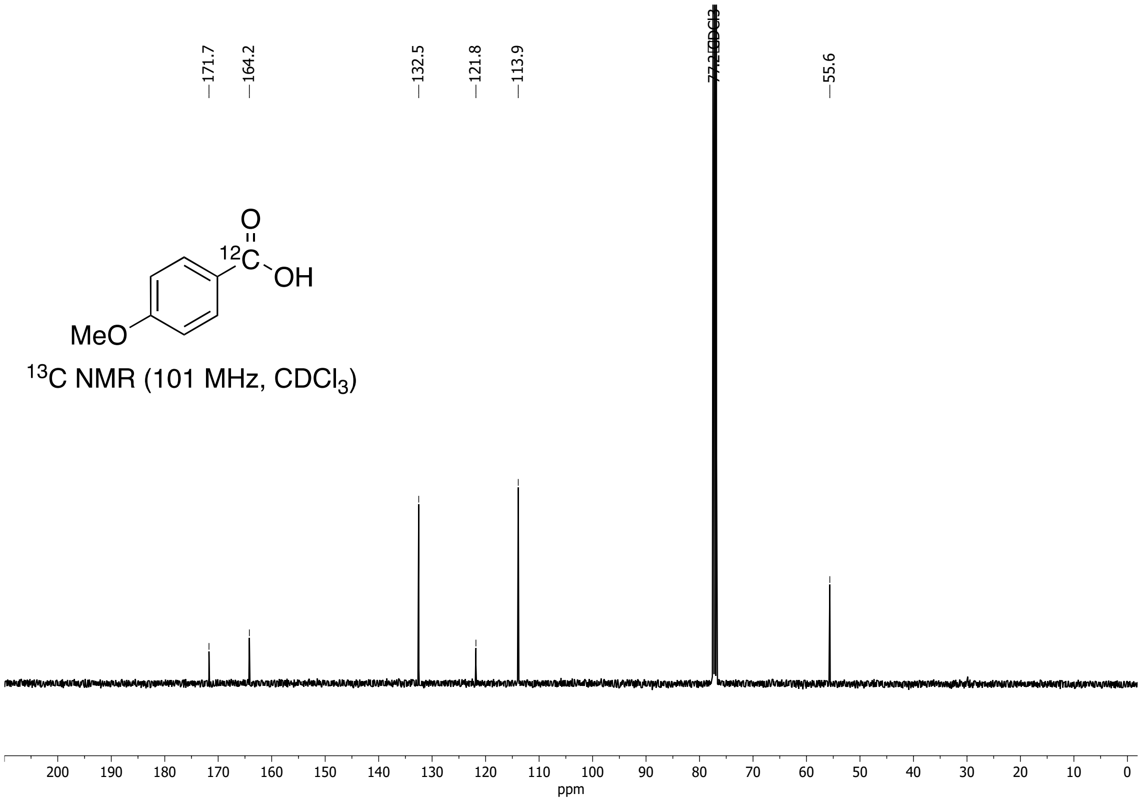
171.7
164.2
132.5
121.8
113.9
55.6
O
12C
OH
MeO
13C NMR (101 MHz, CDCl3)
200 190 180 170 160 150 140 130 120 110 100 90 80 70 60 50 40 30 20 10 0
ppm

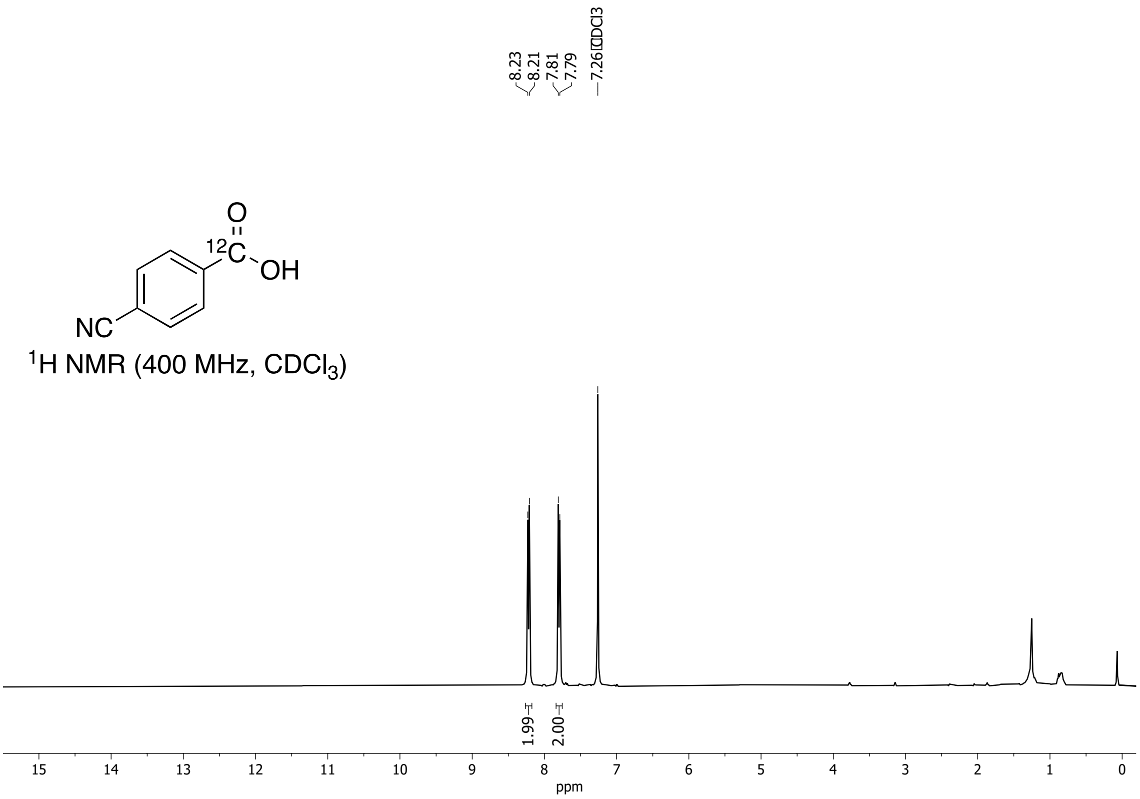
8.23
8.21
7.81
7.79
7.26 CDCl3
O
12C
OH
NC
1H NMR (400 MHz, CDCl3)
1.99
2.00
15 14 13 12 11 10 9 8 7 6 5 4 3 2 1 0
ppm

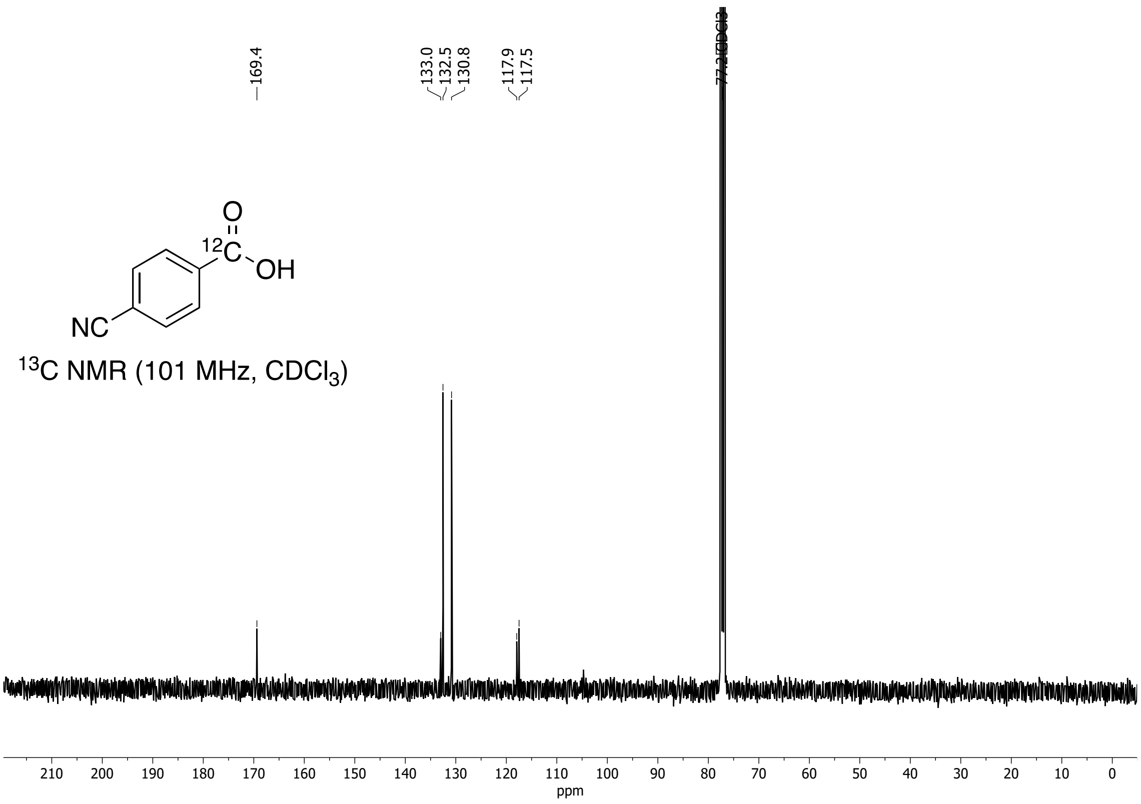
169.4
133.0
132.5
130.8
117.9
117.5
O
12C
OH
NC
13C NMR (101 MHz, CDCl3)
210 200 190 180 170 160 150 140 130 120 110 100 90 80 70 60 50 40 30 20 10 0
ppm

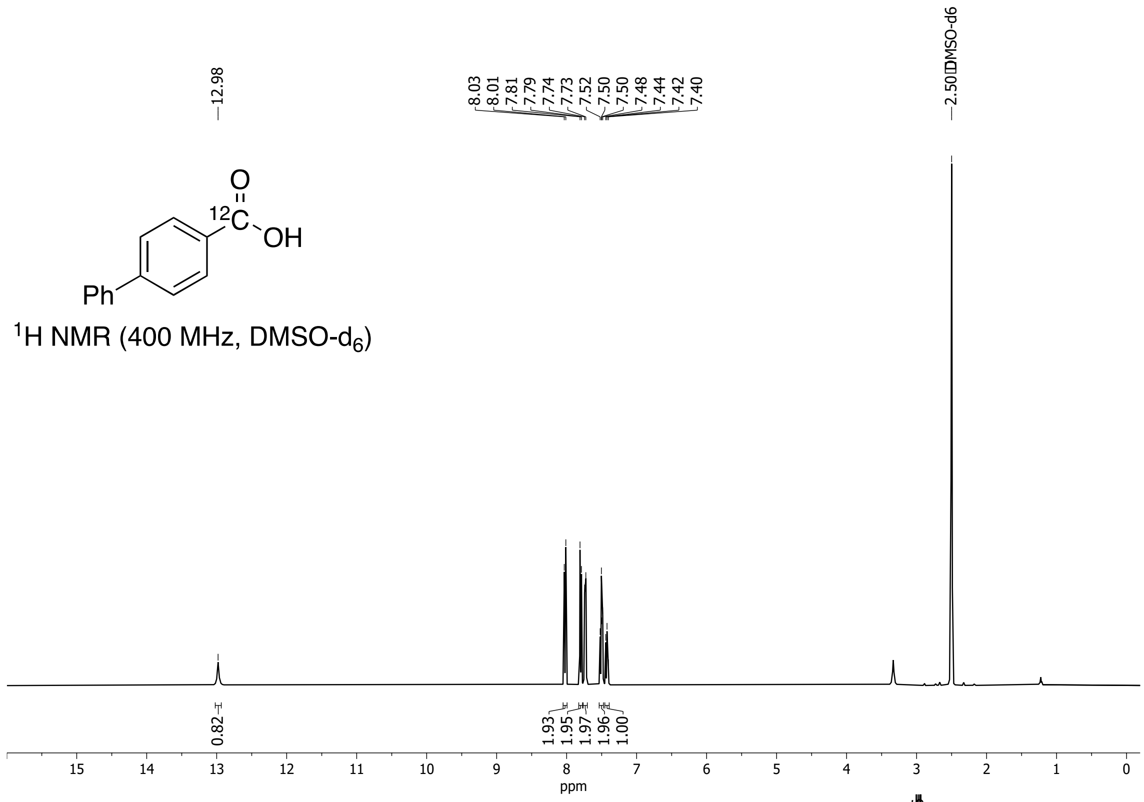
12.98
8.03
8.01
7.81
7.79
7.74
7.73
7.52
7.50
7.50
7.48
7.44
7.42
7.40
2.50 DMSO-d6
O
12C
OH
Ph
1H NMR (400 MHz, DMSO-d6)
0.82
1.93
1.95
1.97
1.96
1.00
15
14
13
12
11
10
9
8
7
6
5
4
3
2
1
0
ppm

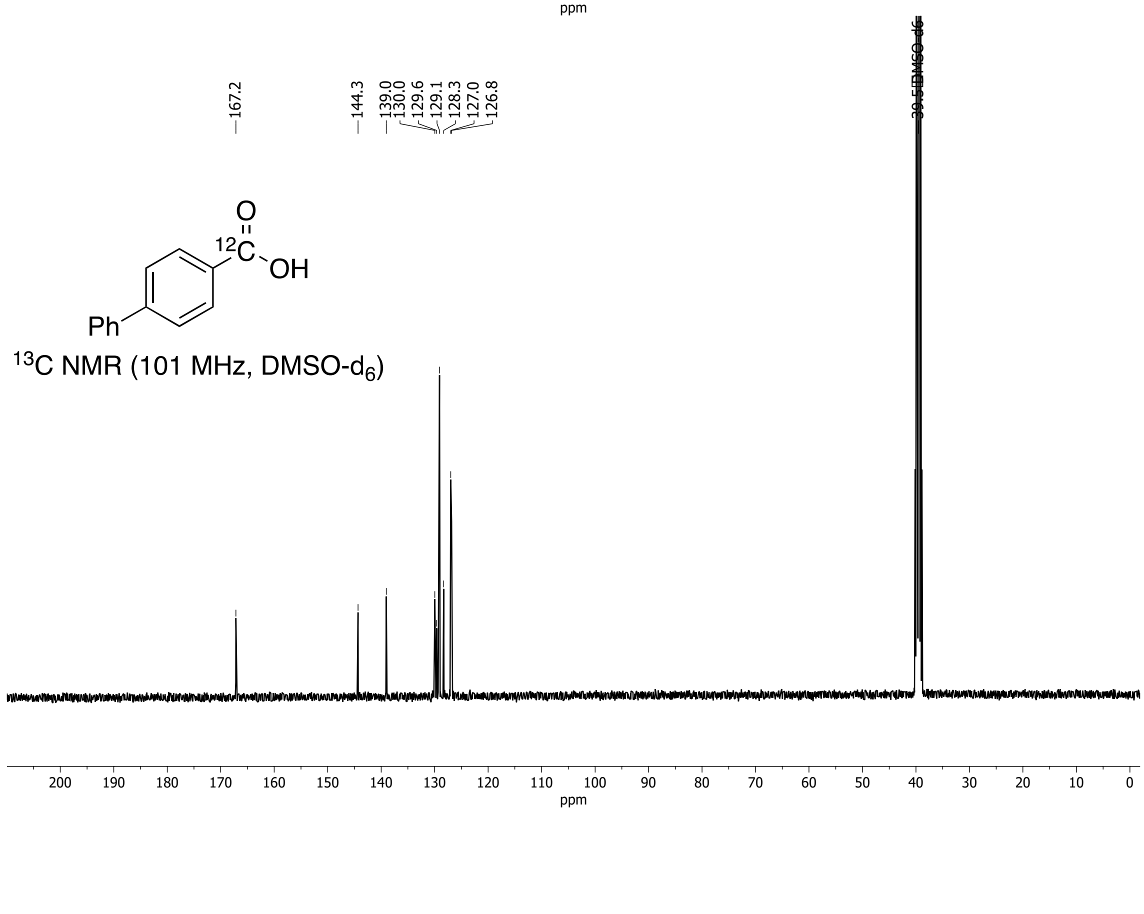
167.2
144.3
139.0
130.0
129.6
129.1
128.3
127.0
126.8
39.5 DMSO-d6
O
12C
OH
Ph
13C NMR (101 MHz, DMSO-d6)
200
190
180
170
160
150
140
130
120
110
100
90
80
70
60
50
40
30
20
10
0
ppm

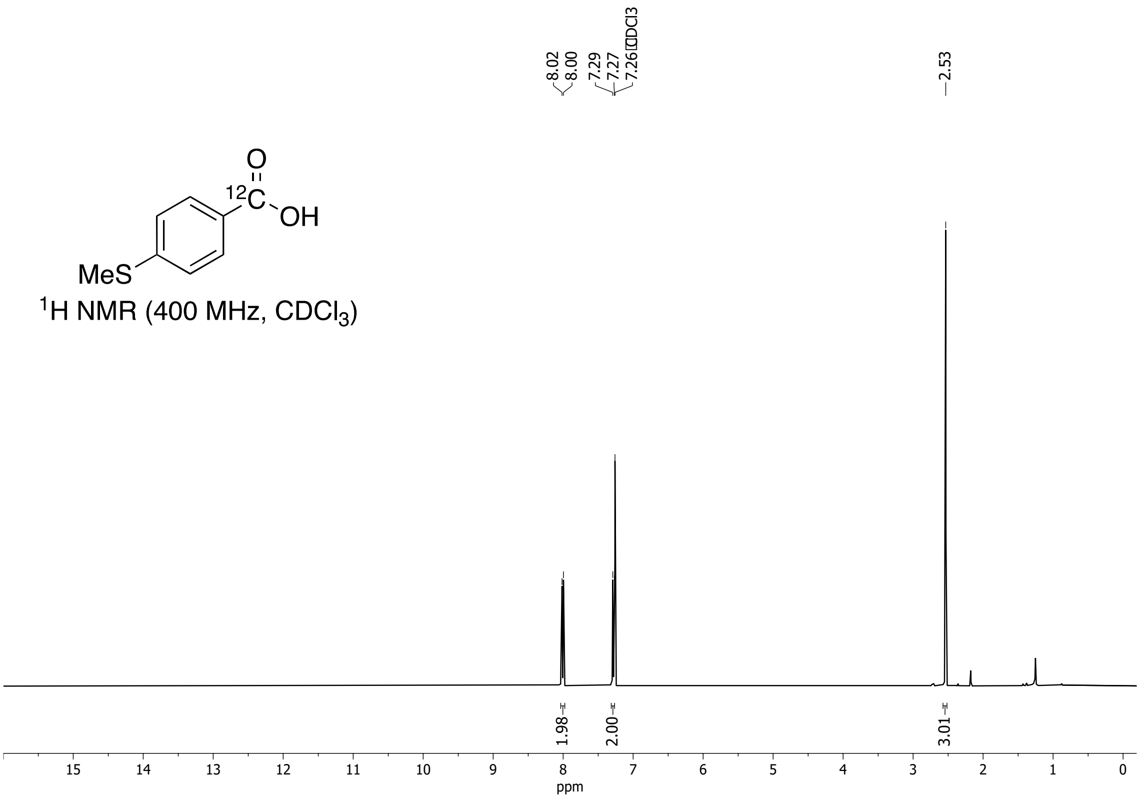

^{1}H NMR (400 MHz, CDCl$_3$)
MeS
^{12}C
OH
O
8.02
8.00
7.29
7.27
7.26 CDCl3
2.53
1.98
2.00
3.01
15 14 13 12 11 10 9 8 7 6 5 4 3 2 1 0
ppm

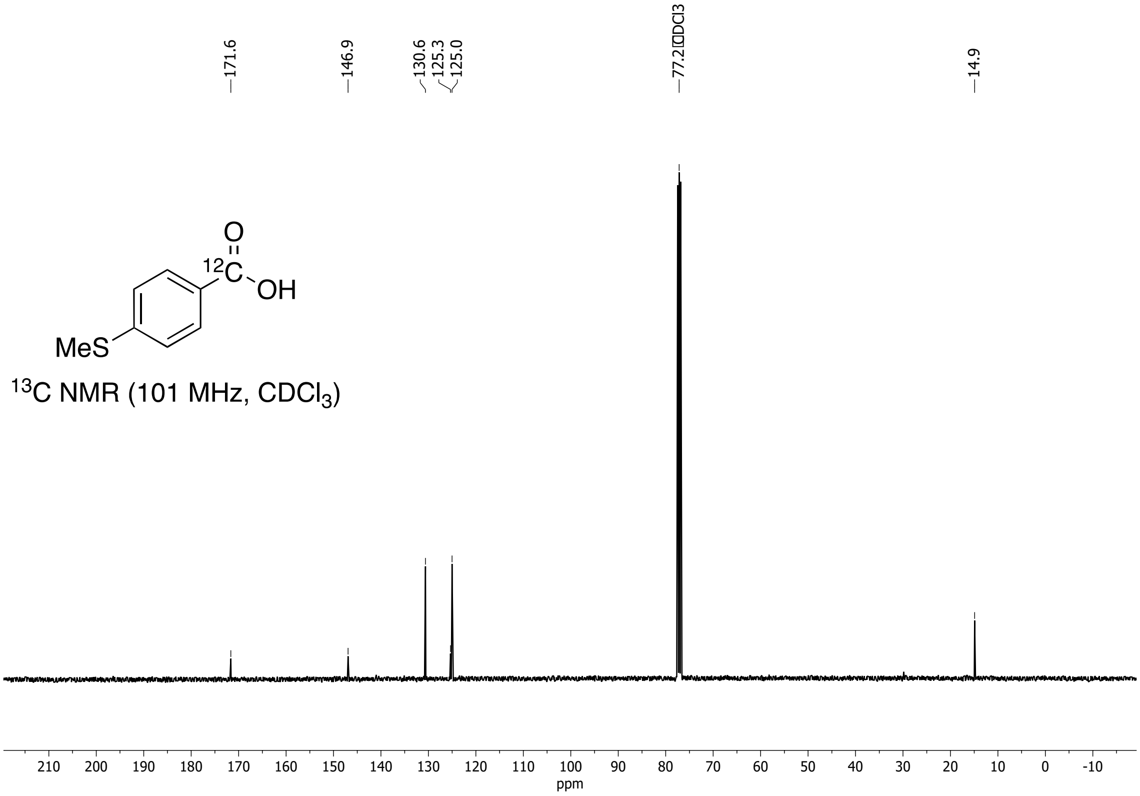

^{13}C NMR (101 MHz, CDCl$_3$)
MeS
^{12}C
OH
O
171.6
146.9
130.6
125.3
125.0
77.2 CDCl3
14.9
210 200 190 180 170 160 150 140 130 120 110 100 90 80 70 60 50 40 30 20 10 0 -10
ppm

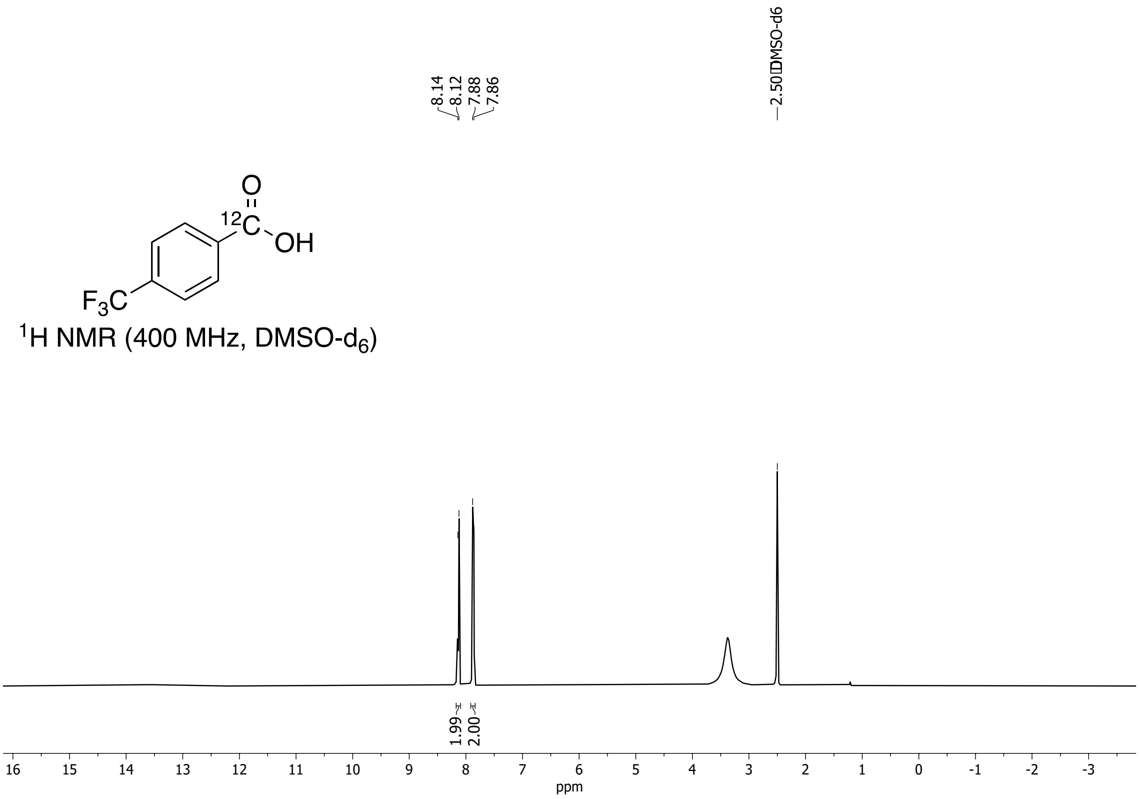
8.14
8.12
7.88
7.86
2.50 DMSO-d6
O
12C
OH
F3C
1H NMR (400 MHz, DMSO-d6)
1.99
2.00
16 15 14 13 12 11 10 9 8 7 6 5 4 3 2 1 0 -1 -2 -3
ppm

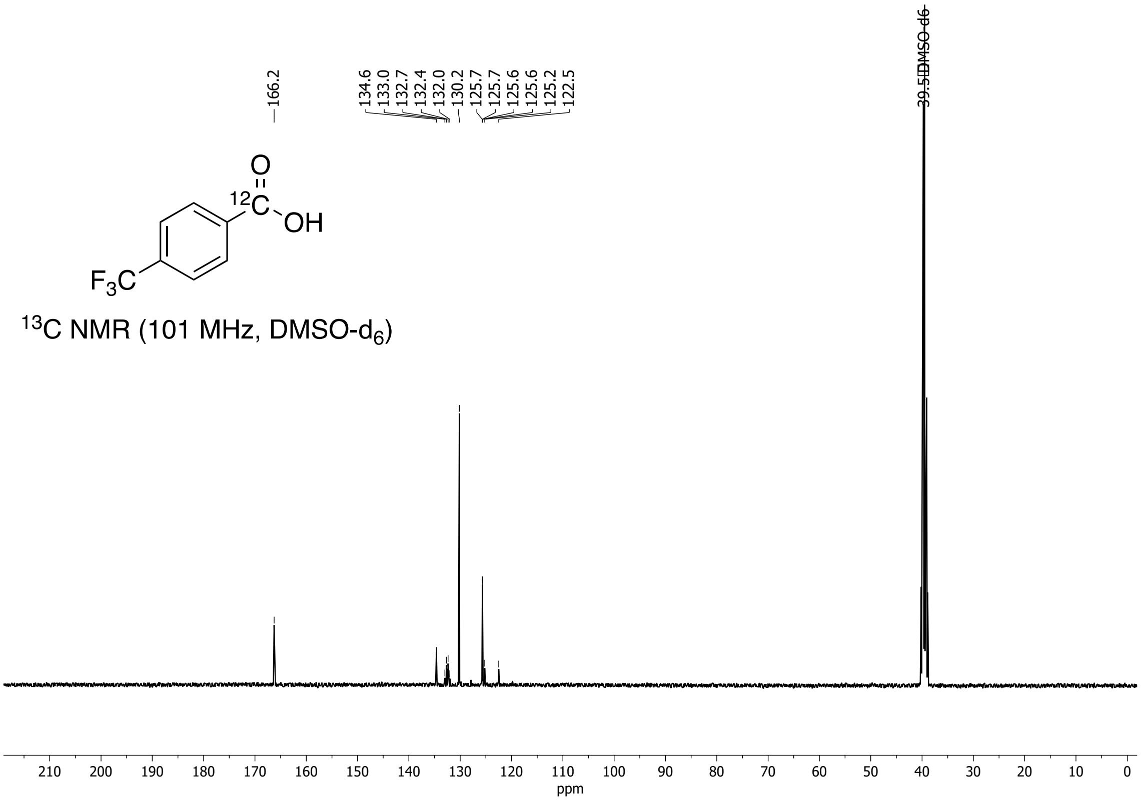
166.2
134.6
133.0
132.7
132.4
132.0
130.2
125.7
125.7
125.6
125.6
125.2
122.5
39.52 DMSO-d6
O
12C
OH
F3C
13C NMR (101 MHz, DMSO-d6)
210 200 190 180 170 160 150 140 130 120 110 100 90 80 70 60 50 40 30 20 10 0
ppm

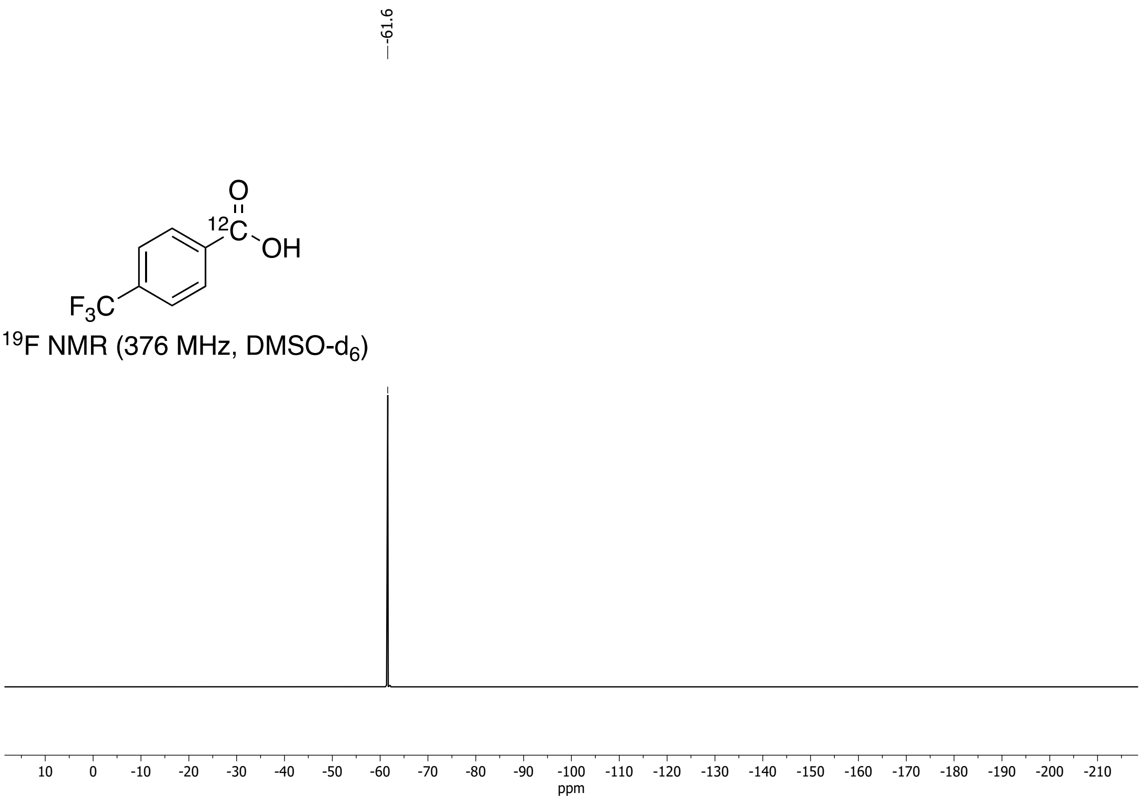

-61.6
O
12C
OH
F3C
19F NMR (376 MHz, DMSO-d6)
ppm

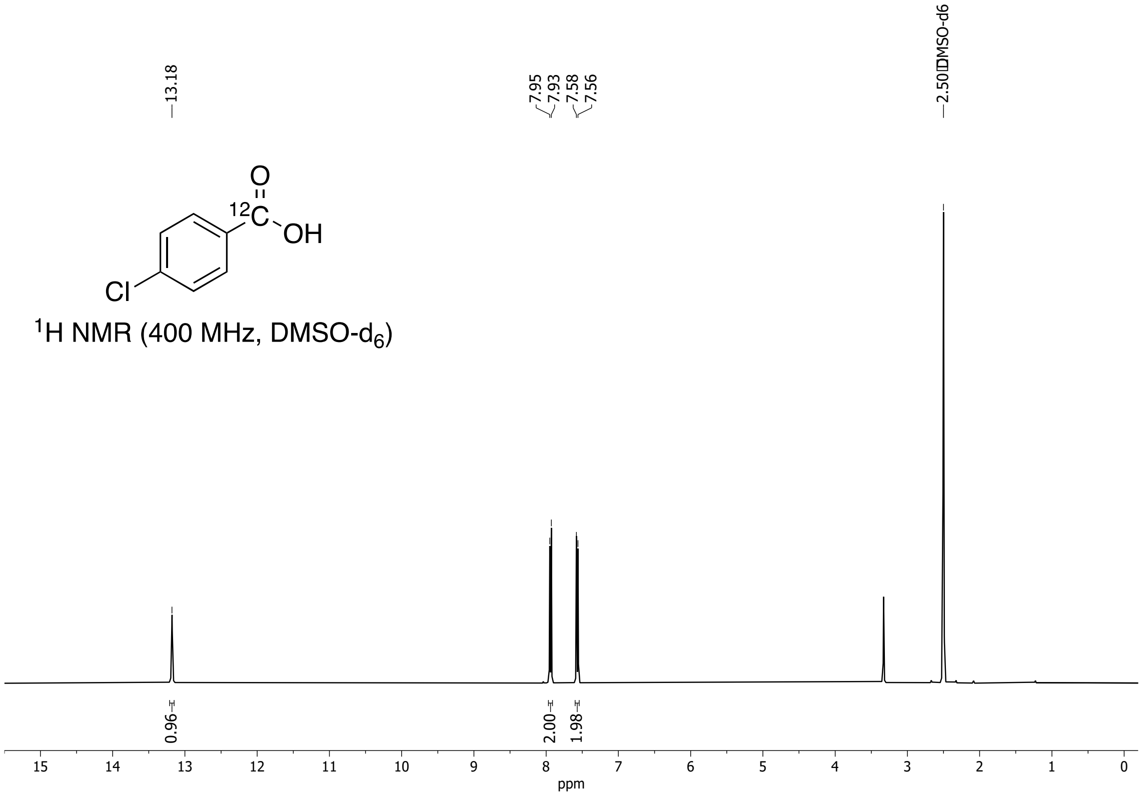

13.18
7.95
7.93
7.58
7.56
2.50 DMSO-d6
O
12C
OH
Cl
1H NMR (400 MHz, DMSO-d6)
0.96
2.00
1.98
ppm

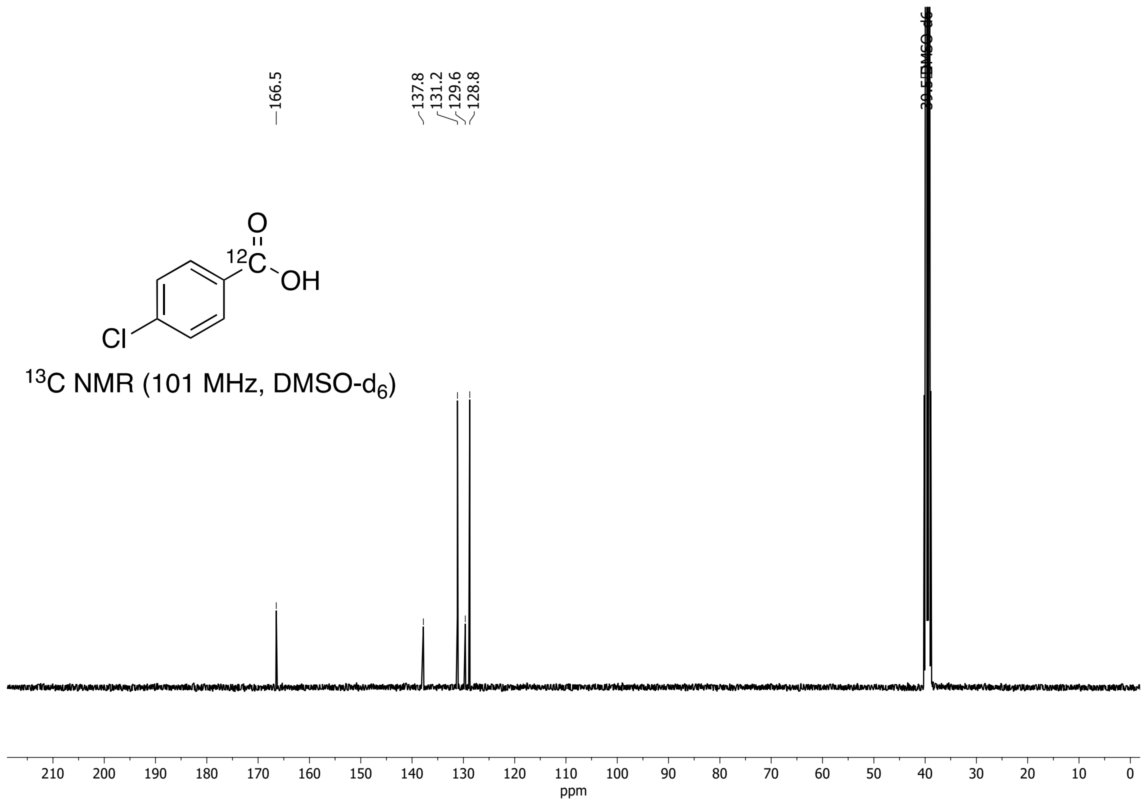
166.5
137.8
131.2
129.6
128.8
O
12C
OH
Cl
13C NMR (101 MHz, DMSO-d6)
210 200 190 180 170 160 150 140 130 120 110 100 90 80 70 60 50 40 30 20 10 0
ppm

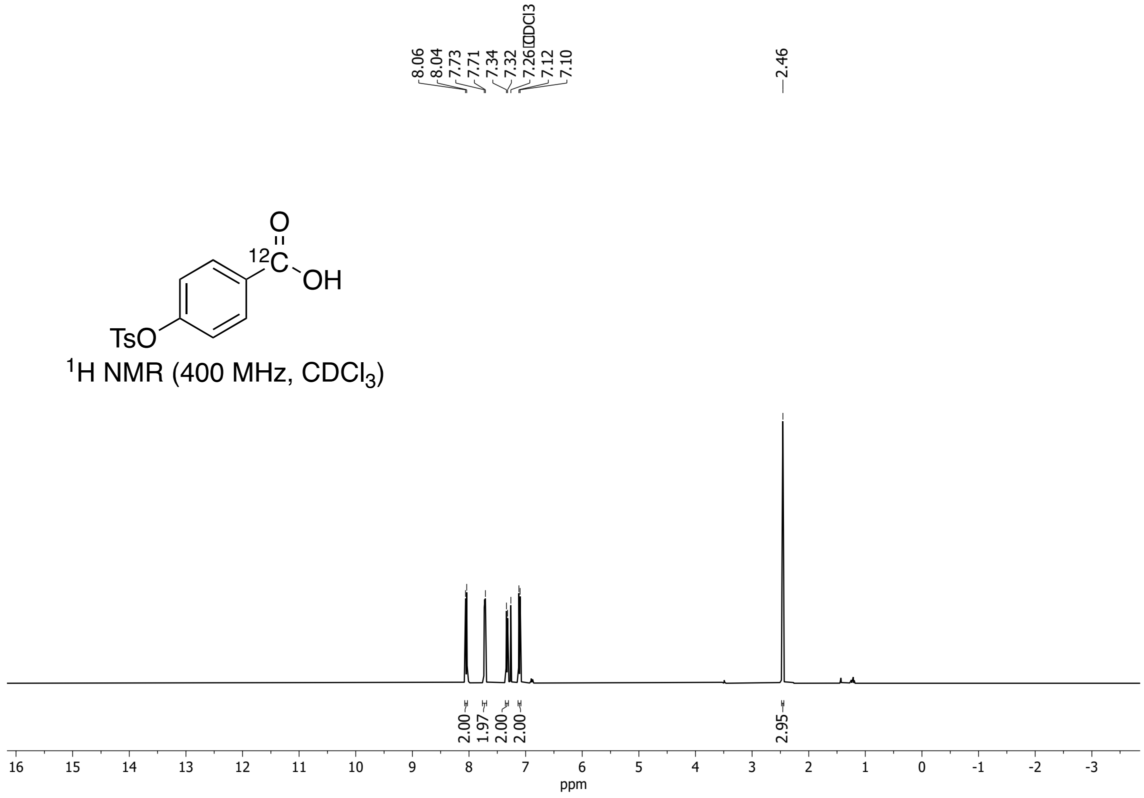
8.06
8.04
7.73
7.71
7.34
7.32
7.26 CDCl3
7.12
7.10
2.46
O
12C
OH
TsO
1H NMR (400 MHz, CDCl3)
2.00
1.97
2.00
2.00
2.95
16 15 14 13 12 11 10 9 8 7 6 5 4 3 2 1 0 -1 -2 -3
ppm

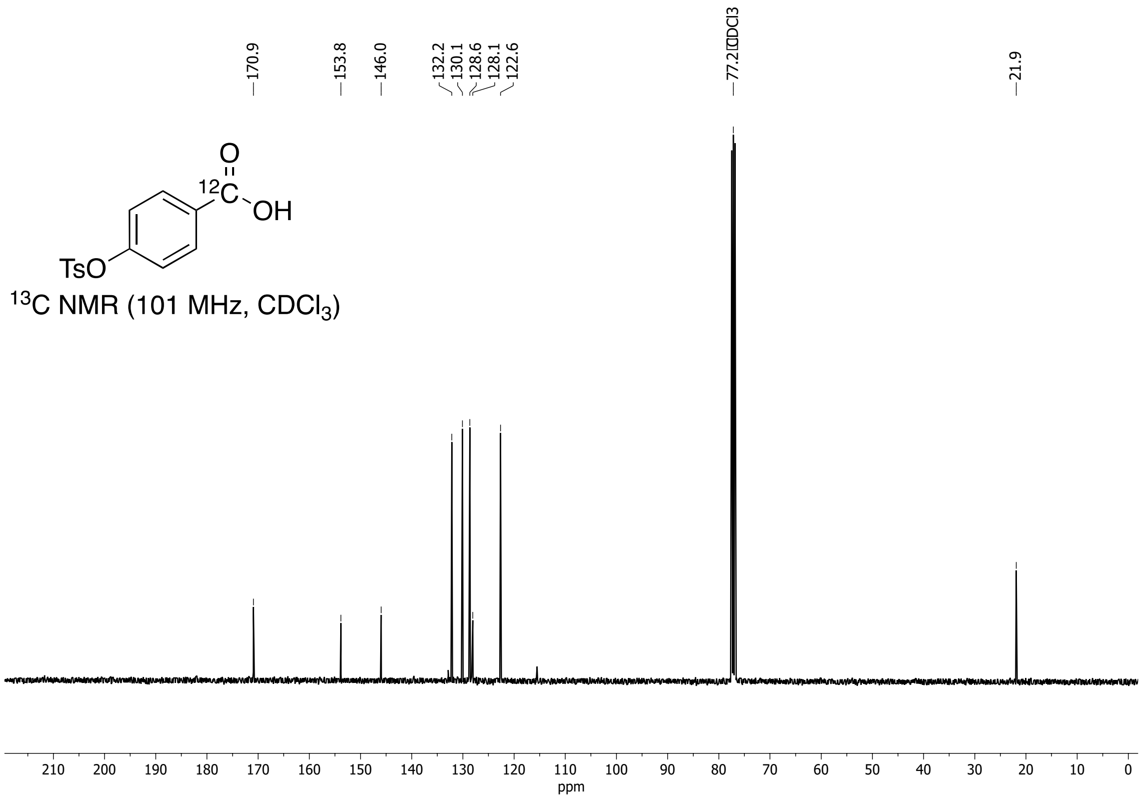
TsO
12C
O
OH
13C NMR (101 MHz, CDCl3)
170.9
153.8
146.0
132.2
130.1
128.6
128.1
122.6
77.2 CDCl3
21.9
ppm

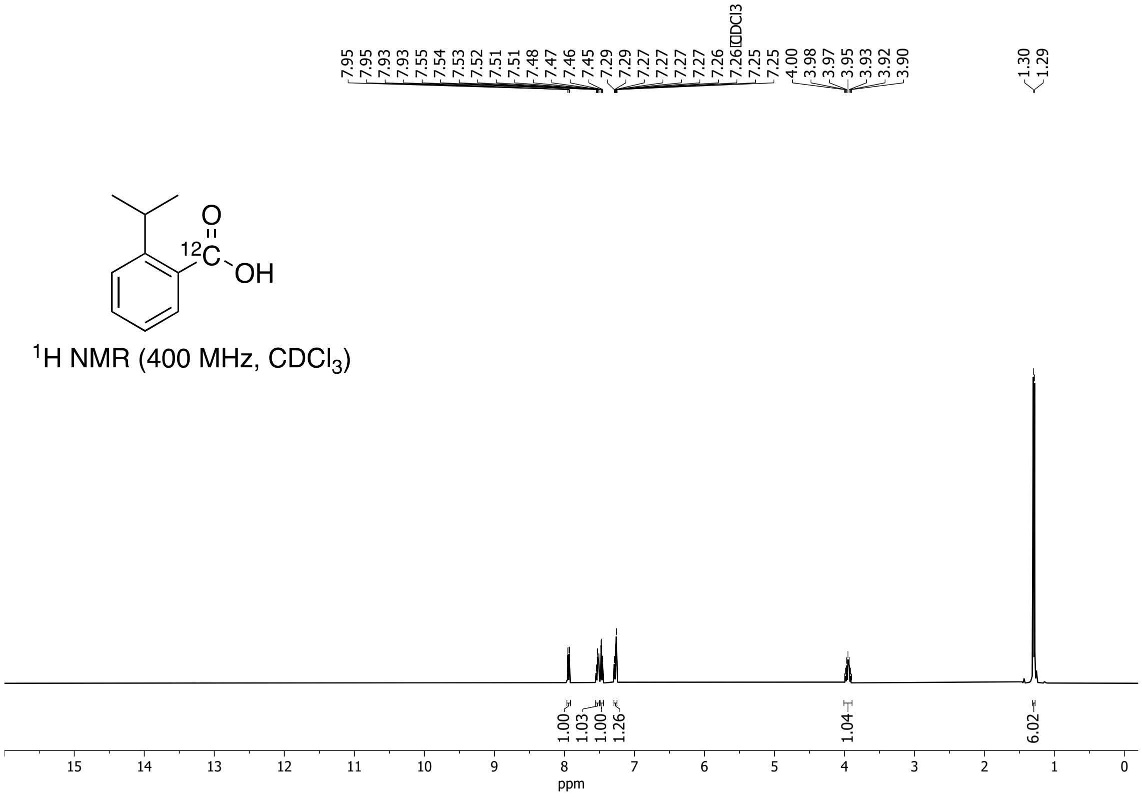
12C
O
OH
1H NMR (400 MHz, CDCl3)
7.95
7.95
7.93
7.93
7.55
7.54
7.53
7.52
7.51
7.51
7.48
7.47
7.46
7.45
7.29
7.29
7.27
7.27
7.27
7.27
7.26
7.26 CDCl3
7.25
7.25
4.00
3.98
3.97
3.95
3.93
3.92
3.90
1.30
1.29
1.00
1.03
1.00
1.26
1.04
6.02
ppm

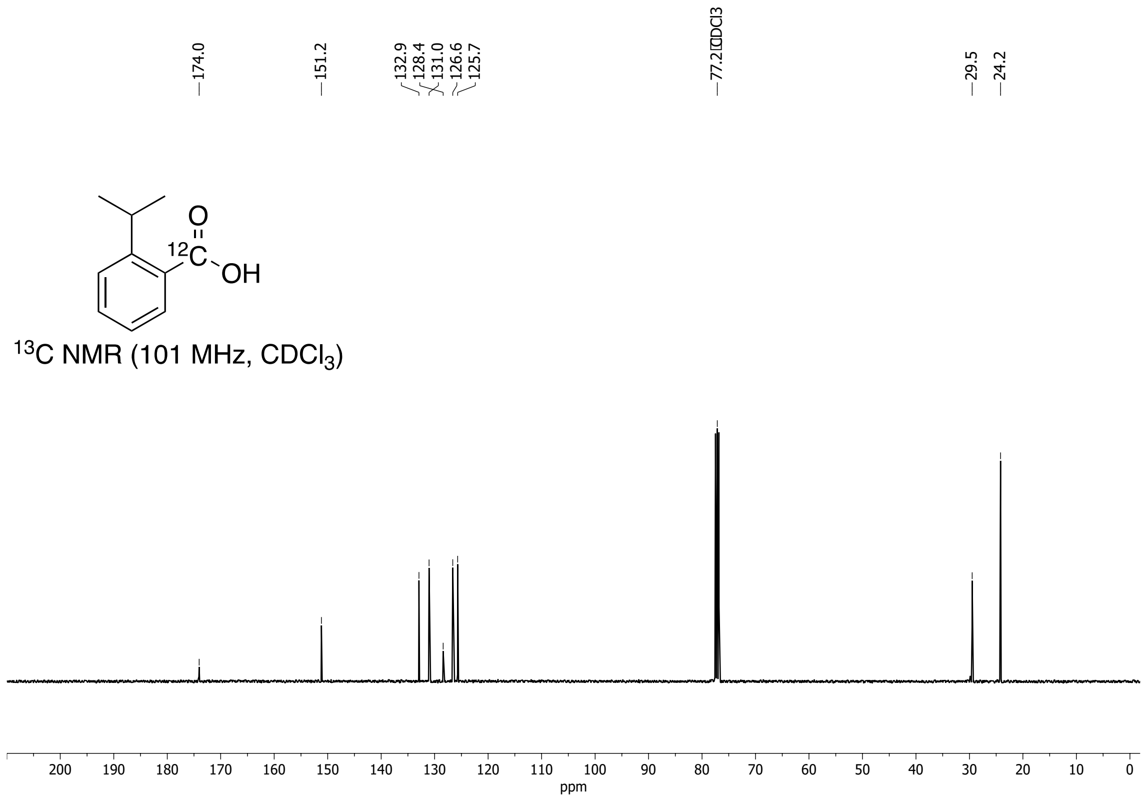
174.0
151.2
132.9
128.4
131.0
126.6
125.7
77.2 CDCl3
29.5
24.2
O
12C
OH
13C NMR (101 MHz, CDCl3)
200 190 180 170 160 150 140 130 120 110 100 90 80 70 60 50 40 30 20 10 0
ppm

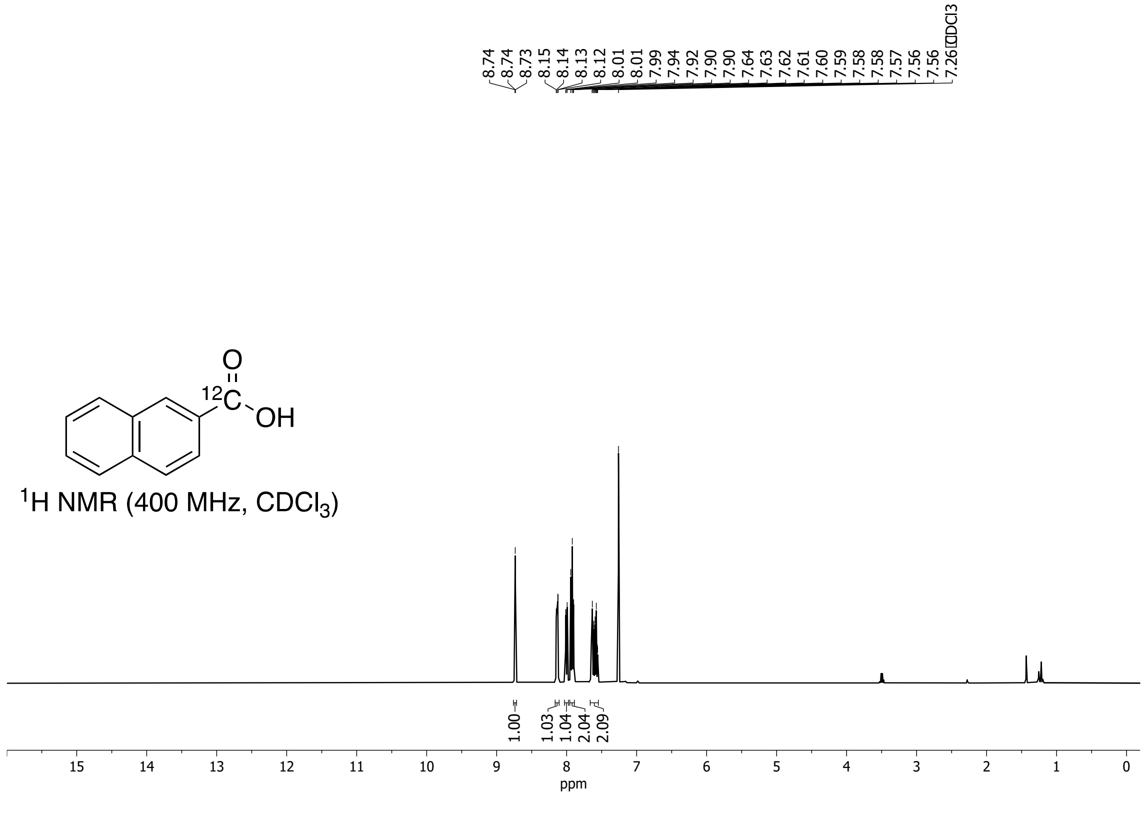
8.74
8.74
8.73
8.15
8.14
8.13
8.12
8.01
8.01
7.99
7.94
7.92
7.90
7.90
7.64
7.63
7.62
7.61
7.60
7.59
7.58
7.58
7.57
7.56
7.56
7.26 CDCl3
O
12C
OH
1H NMR (400 MHz, CDCl3)
1.00
1.03
1.04
2.04
2.09
15 14 13 12 11 10 9 8 7 6 5 4 3 2 1 0
ppm

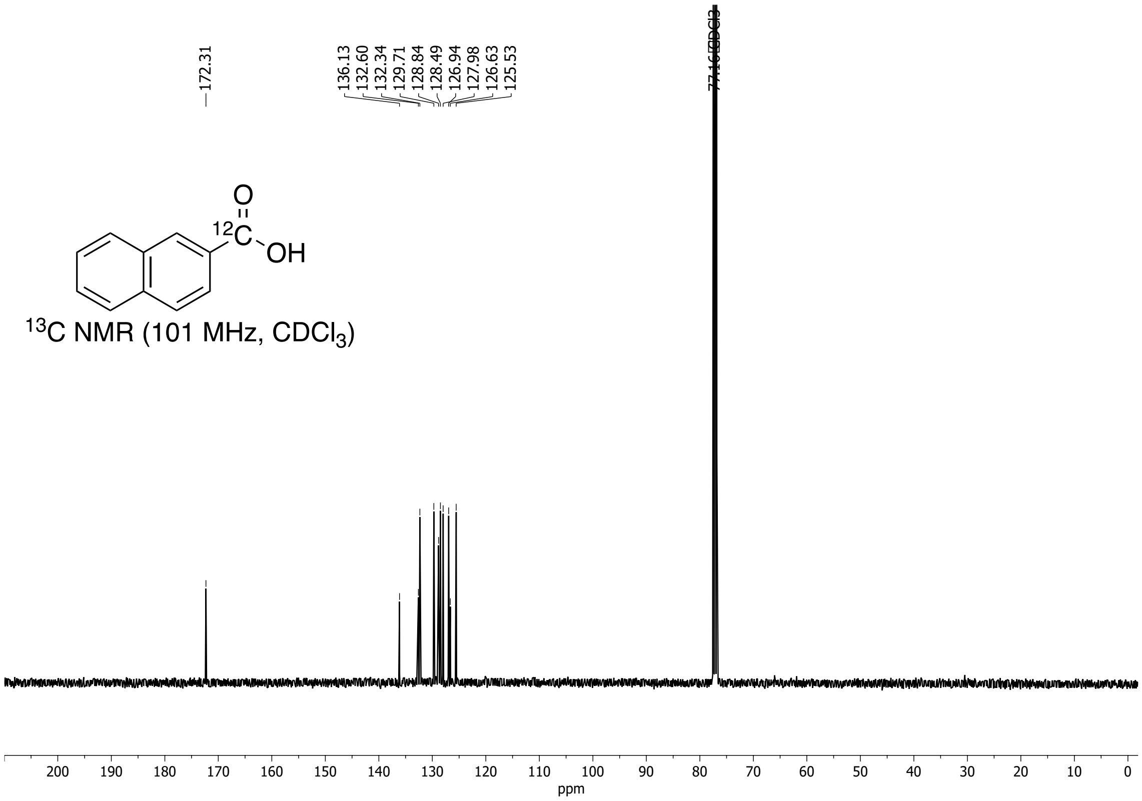
172.31
136.13
132.60
132.34
129.71
128.84
128.49
126.94
127.98
126.63
125.53
O
12C
OH
13C NMR (101 MHz, CDCl3)
ppm

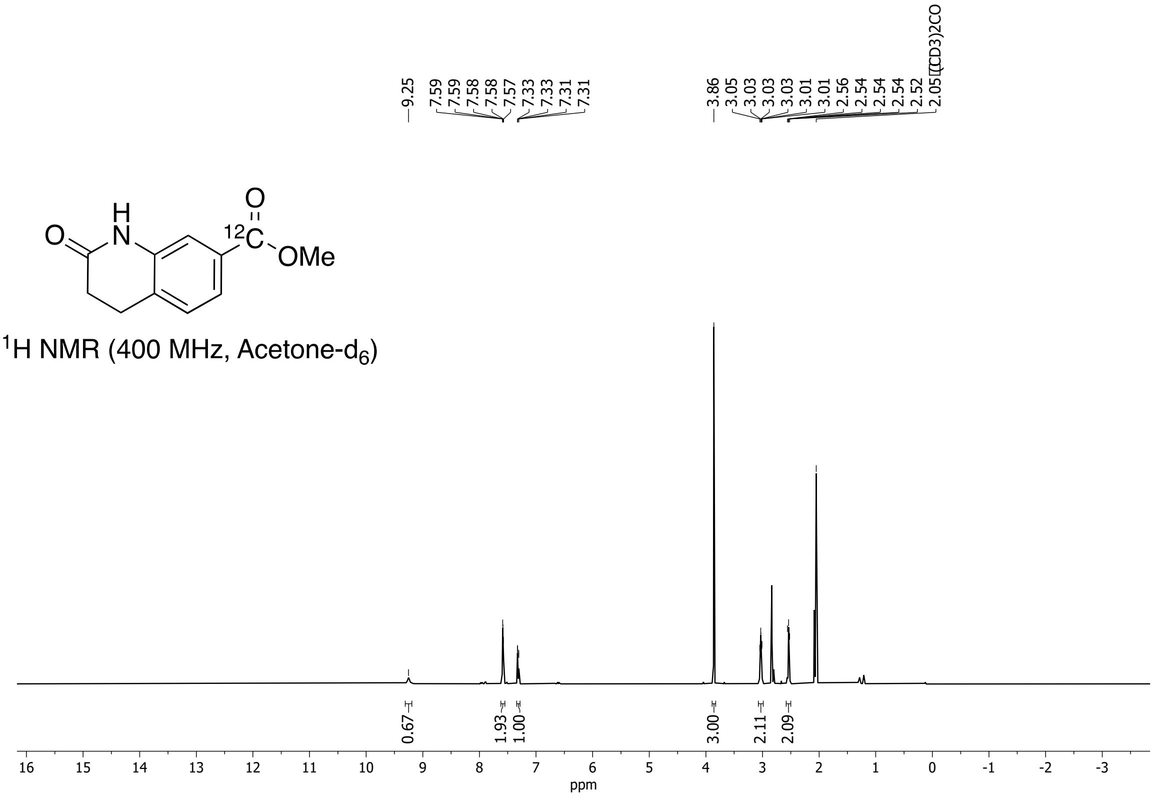
9.25
7.59
7.59
7.58
7.57
7.33
7.33
7.31
7.31
3.86
3.05
3.03
3.03
3.01
2.56
2.54
2.54
2.52
(CD3)2CO
O
H
N
12C
OMe
1H NMR (400 MHz, Acetone-d6)
0.67
1.93
1.00
3.00
2.11
2.09
ppm

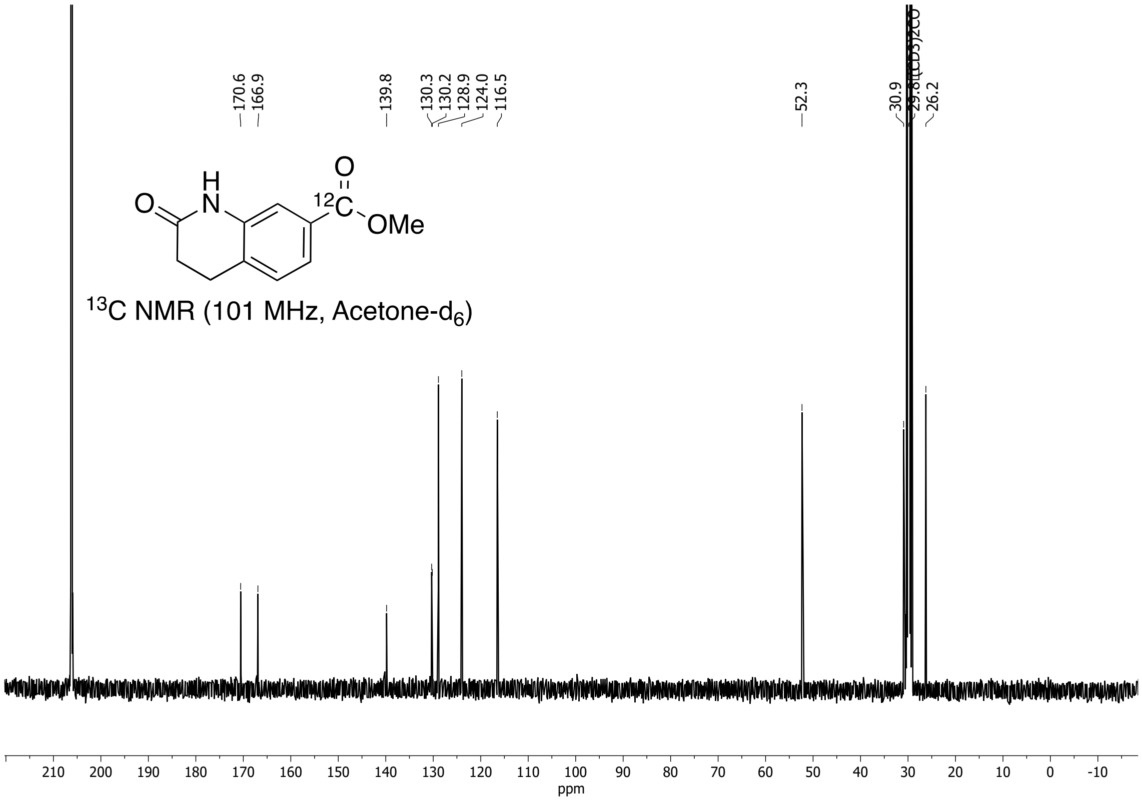

170.6
166.9
139.8
130.3
130.2
128.9
124.0
116.5
52.3
30.9
29.8 (CD3)2CO
26.2
O
H
N
12C
OMe
13C NMR (101 MHz, Acetone-d6)
ppm

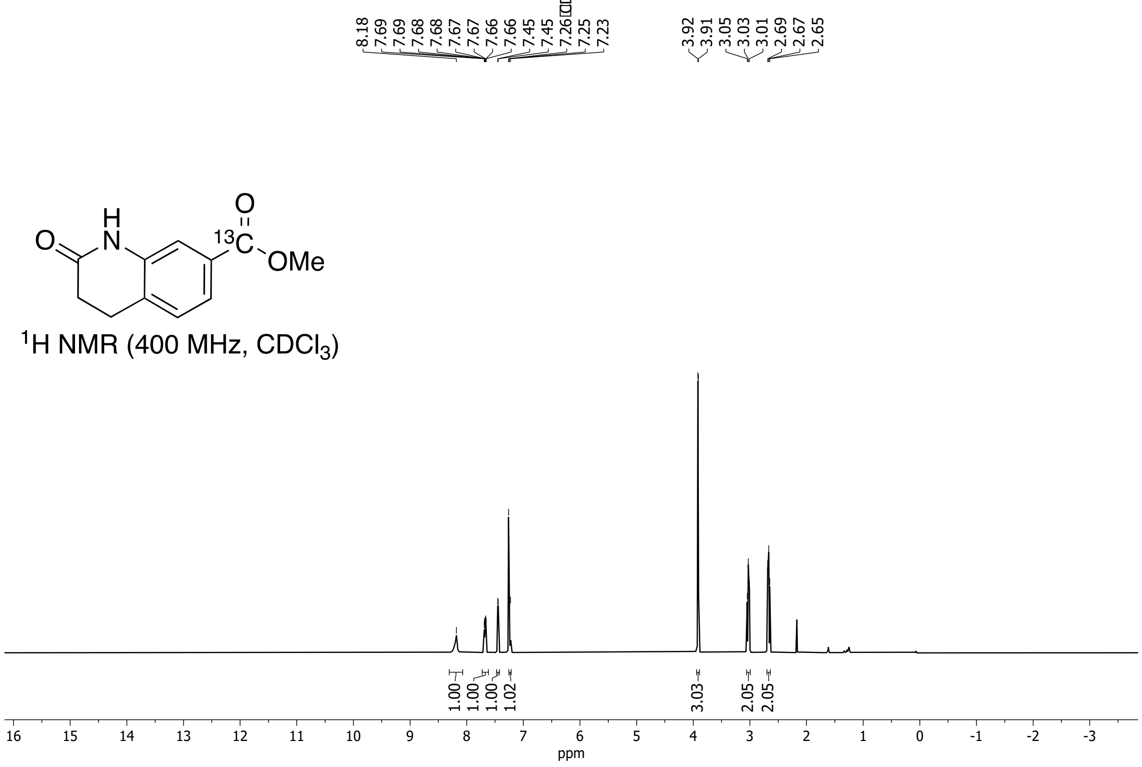

8.18
7.69
7.69
7.68
7.68
7.67
7.67
7.66
7.66
7.45
7.45
7.26 CDCl3
7.25
7.23
3.92
3.91
3.05
3.03
3.01
2.69
2.67
2.65
O
H
N
13C
OMe
1H NMR (400 MHz, CDCl3)
1.00
1.00
1.00
1.02
3.03
2.05
2.05
ppm

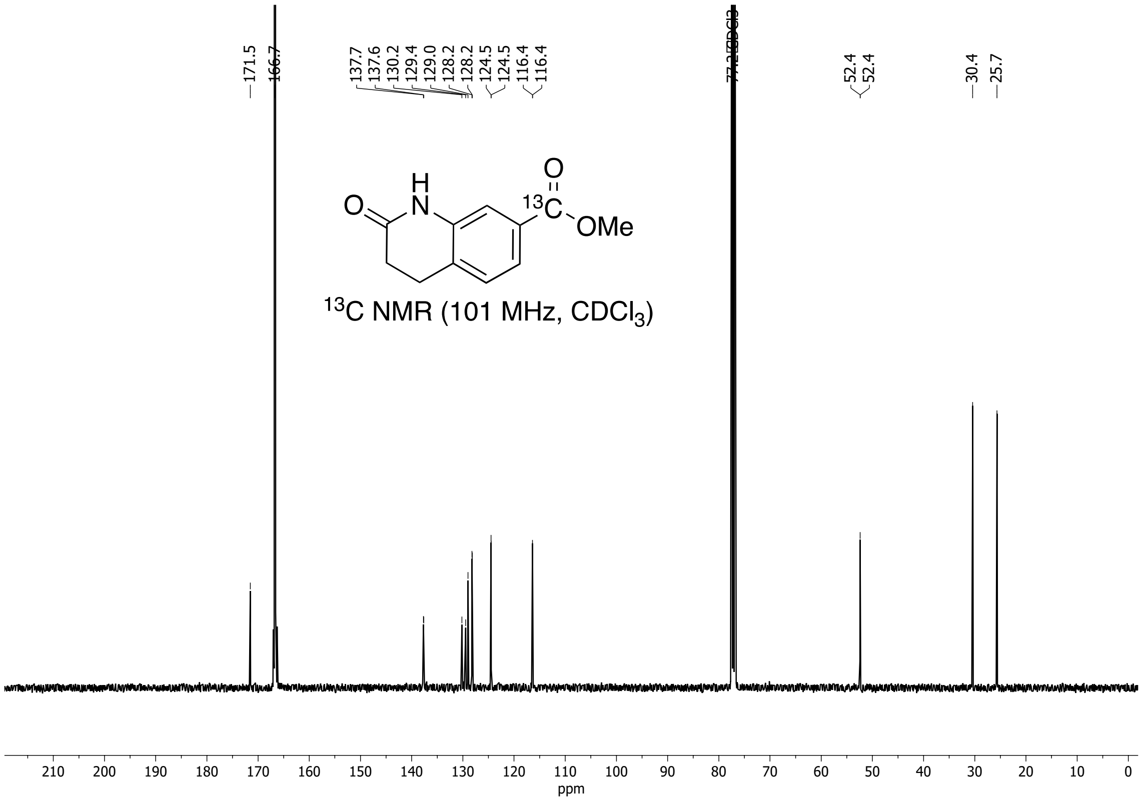

171.5
166.7
137.7
137.6
130.2
129.4
129.0
128.2
128.2
124.5
124.5
116.4
116.4
52.4
52.4
30.4
25.7
H
O
N
13C
O
OMe
13C NMR (101 MHz, CDCl3)
210 200 190 180 170 160 150 140 130 120 110 100 90 80 70 60 50 40 30 20 10 0
ppm

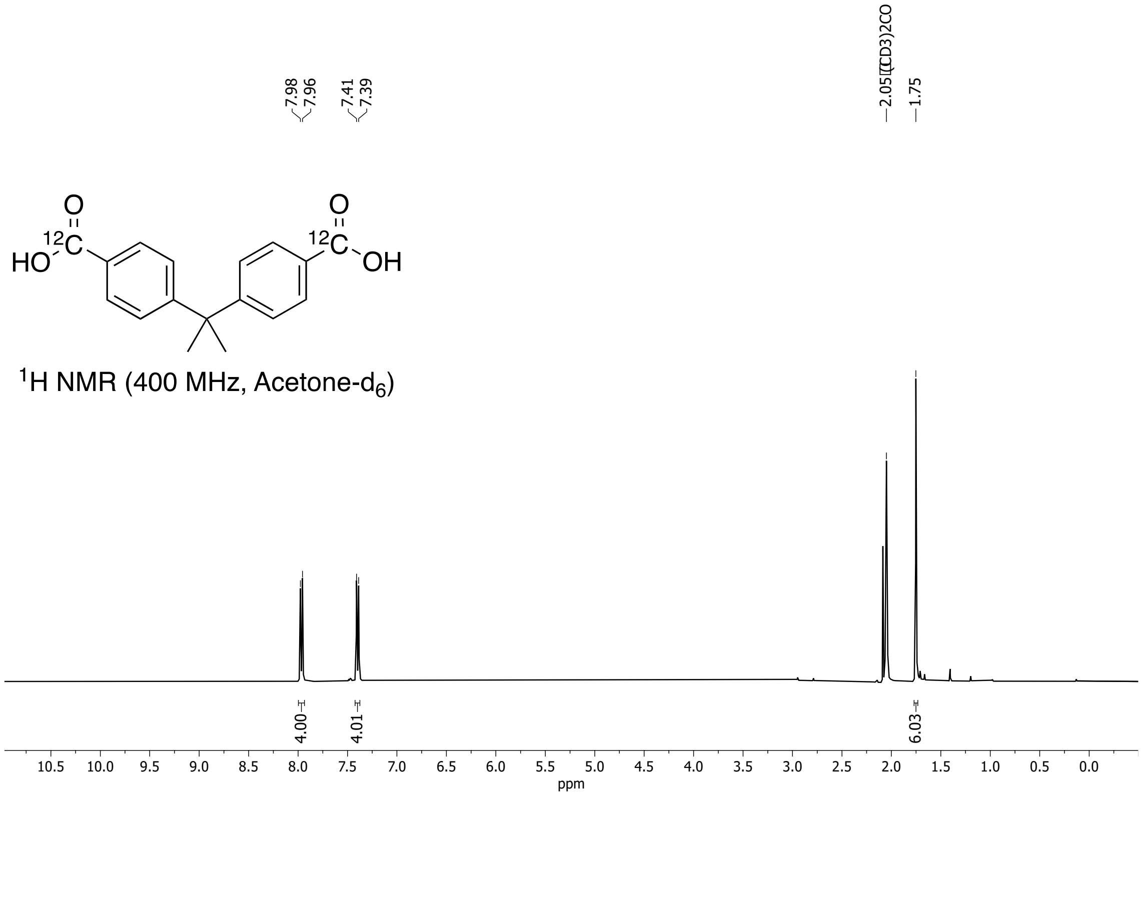

7.98
7.96
7.41
7.39
2.05 ((CD3)2CO)
1.75
O
12C
HO
O
12C
OH
1H NMR (400 MHz, Acetone-d6)
4.00
4.01
6.03
10.5 10.0 9.5 9.0 8.5 8.0 7.5 7.0 6.5 6.0 5.5 5.0 4.5 4.0 3.5 3.0 2.5 2.0 1.5 1.0 0.5 0.0
ppm

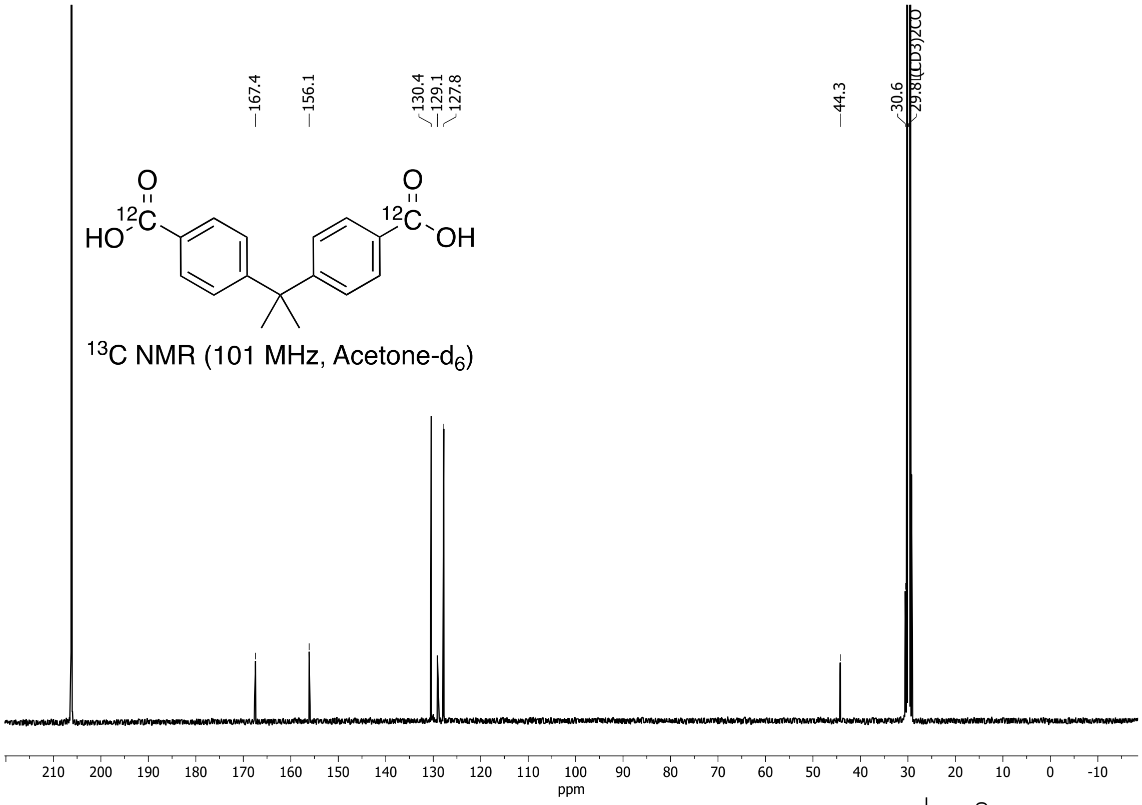

167.4
156.1
130.4
129.1
127.8
44.3
30.6
29.8((CD3)2CO
^{13}C NMR (101 MHz, Acetone-d$_6$)
210 200 190 180 170 160 150 140 130 120 110 100 90 80 70 60 50 40 30 20 10 0 -10
ppm

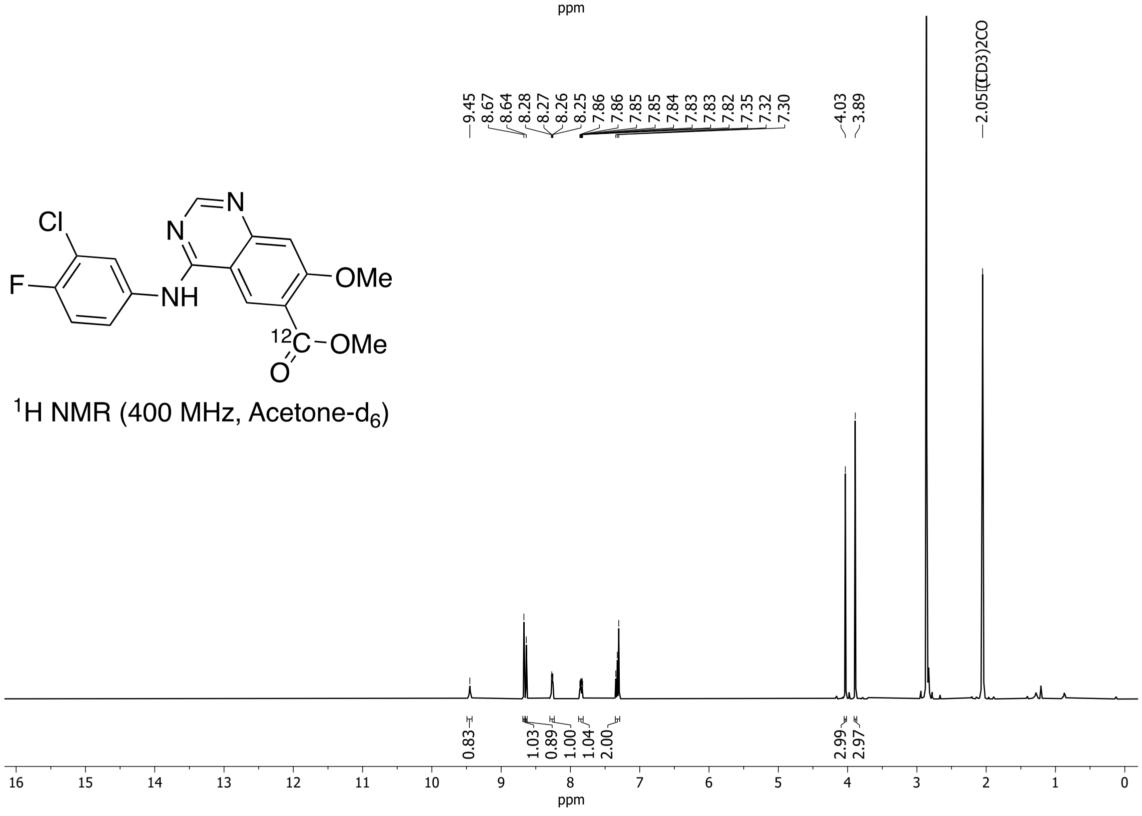

9.45
8.67
8.64
8.28
8.27
8.26
8.25
7.86
7.86
7.85
7.85
7.84
7.83
7.83
7.82
7.35
7.32
7.30
4.03
3.89
2.05((CD3)2CO
^{1}H NMR (400 MHz, Acetone-d$_6$)
0.83
1.03
0.89
1.00
1.04
2.00
2.99
2.97
16 15 14 13 12 11 10 9 8 7 6 5 4 3 2 1 0
ppm

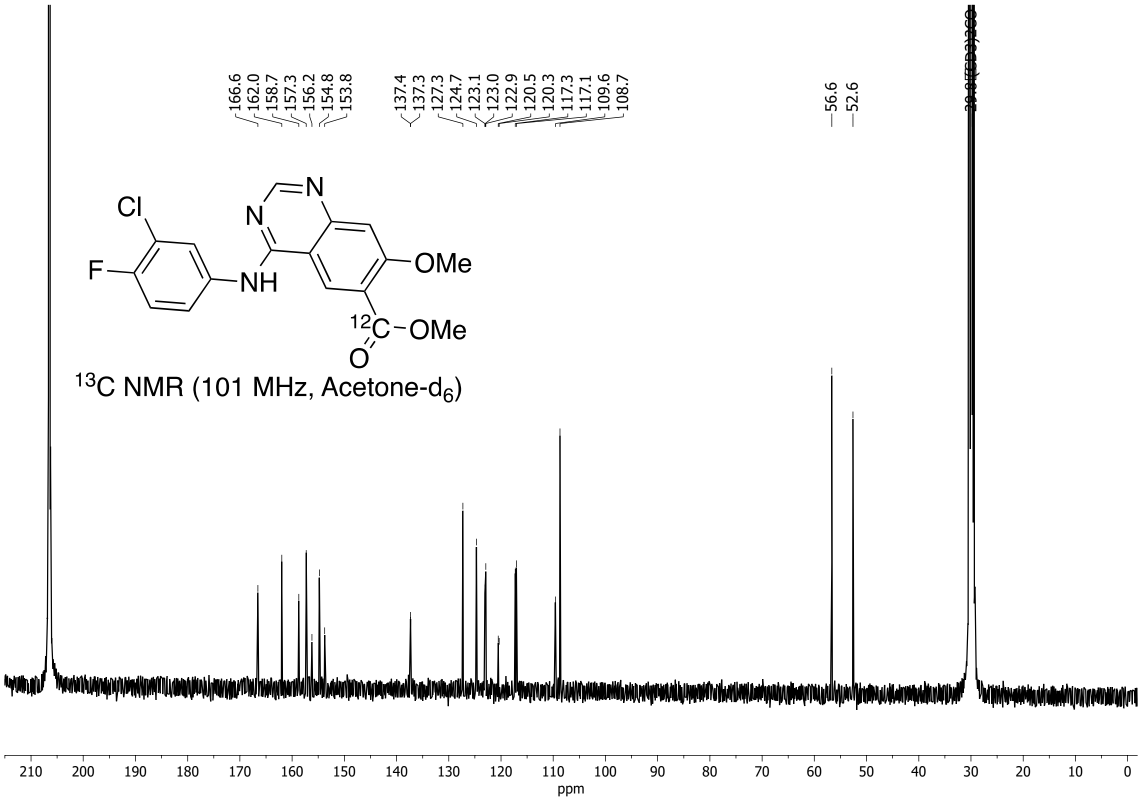

166.6
162.0
158.7
157.3
156.2
154.8
153.8
137.4
137.3
127.3
124.7
123.1
123.0
122.9
120.5
120.3
117.3
117.1
109.6
108.7
56.6
52.6
Cl
F
NH
N
N
OMe
12C
OMe
O
13C NMR (101 MHz, Acetone-d6)
210 200 190 180 170 160 150 140 130 120 110 100 90 80 70 60 50 40 30 20 10 0
ppm

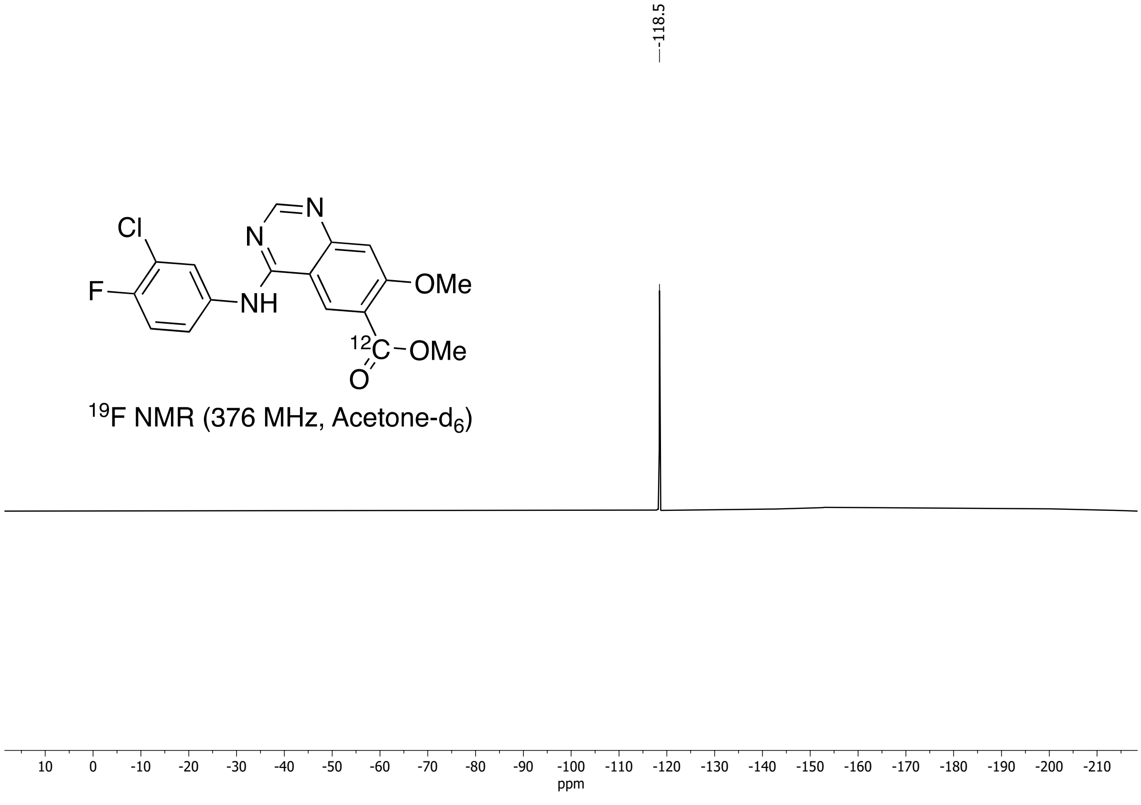

-118.5
Cl
F
NH
N
N
OMe
12C
OMe
O
19F NMR (376 MHz, Acetone-d6)
10 0 -10 -20 -30 -40 -50 -60 -70 -80 -90 -100 -110 -120 -130 -140 -150 -160 -170 -180 -190 -200 -210
ppm

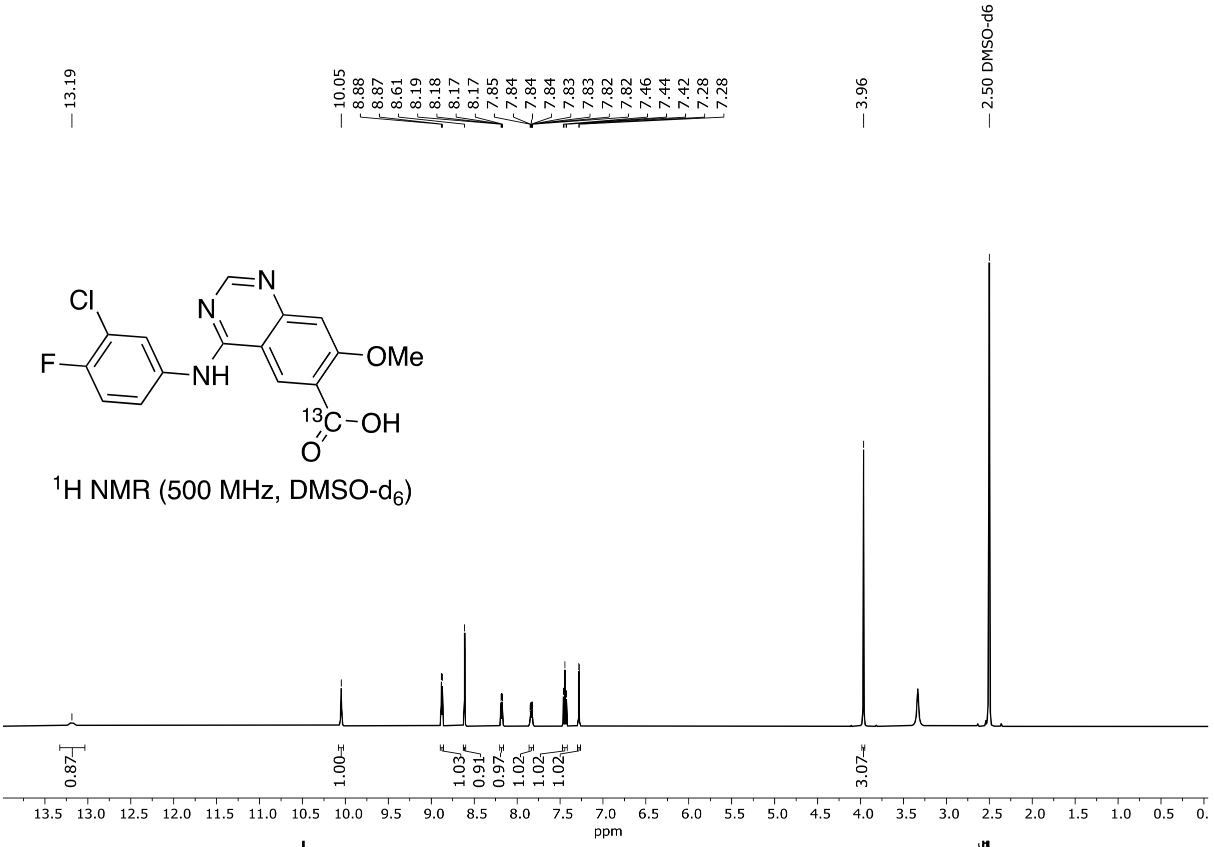

^{1}H NMR (500 MHz, DMSO-d$_6$)

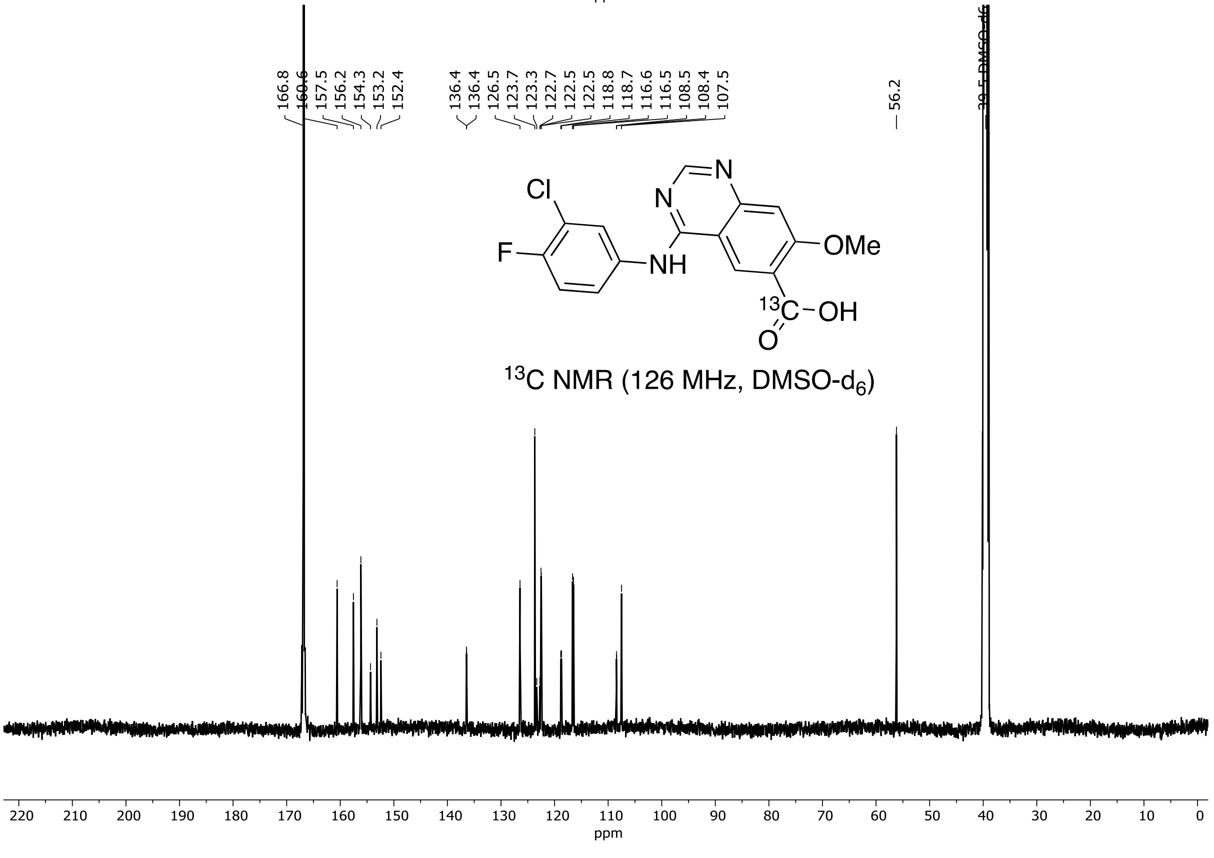

^{13}C NMR (126 MHz, DMSO-d$_6$)

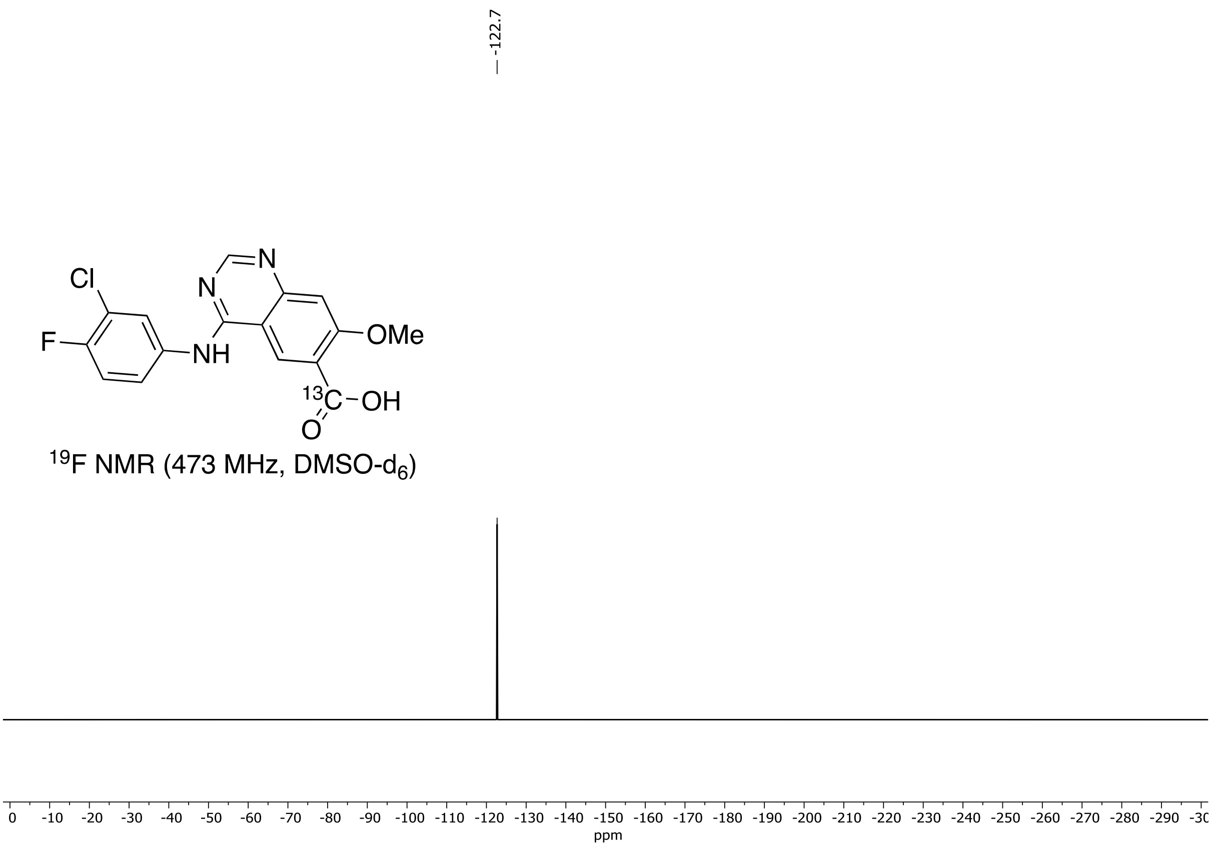
-122.7
Cl
F
N
N
NH
OMe
13C-OH
O
19F NMR (473 MHz, DMSO-d6)
0 -10 -20 -30 -40 -50 -60 -70 -80 -90 -100 -110 -120 -130 -140 -150 -160 -170 -180 -190 -200 -210 -220 -230 -240 -250 -260 -270 -280 -290 -30
ppm

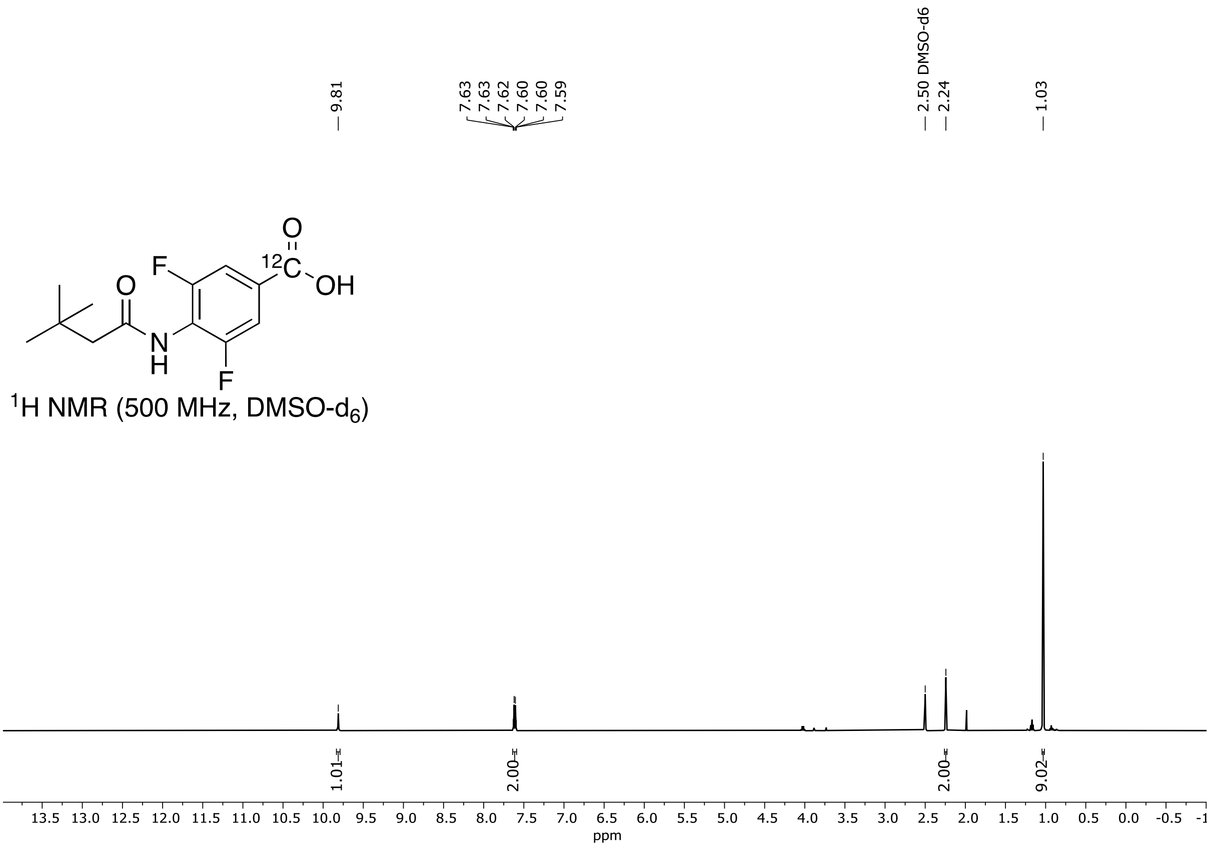
9.81
7.63
7.63
7.62
7.60
7.60
7.59
2.50 DMSO-d6
2.24
1.03
O
F
12C
OH
O
N
H
F
1H NMR (500 MHz, DMSO-d6)
1.01
2.00
2.00
9.02
13.5 13.0 12.5 12.0 11.5 11.0 10.5 10.0 9.5 9.0 8.5 8.0 7.5 7.0 6.5 6.0 5.5 5.0 4.5 4.0 3.5 3.0 2.5 2.0 1.5 1.0 0.5 0.0 -0.5 -1
ppm

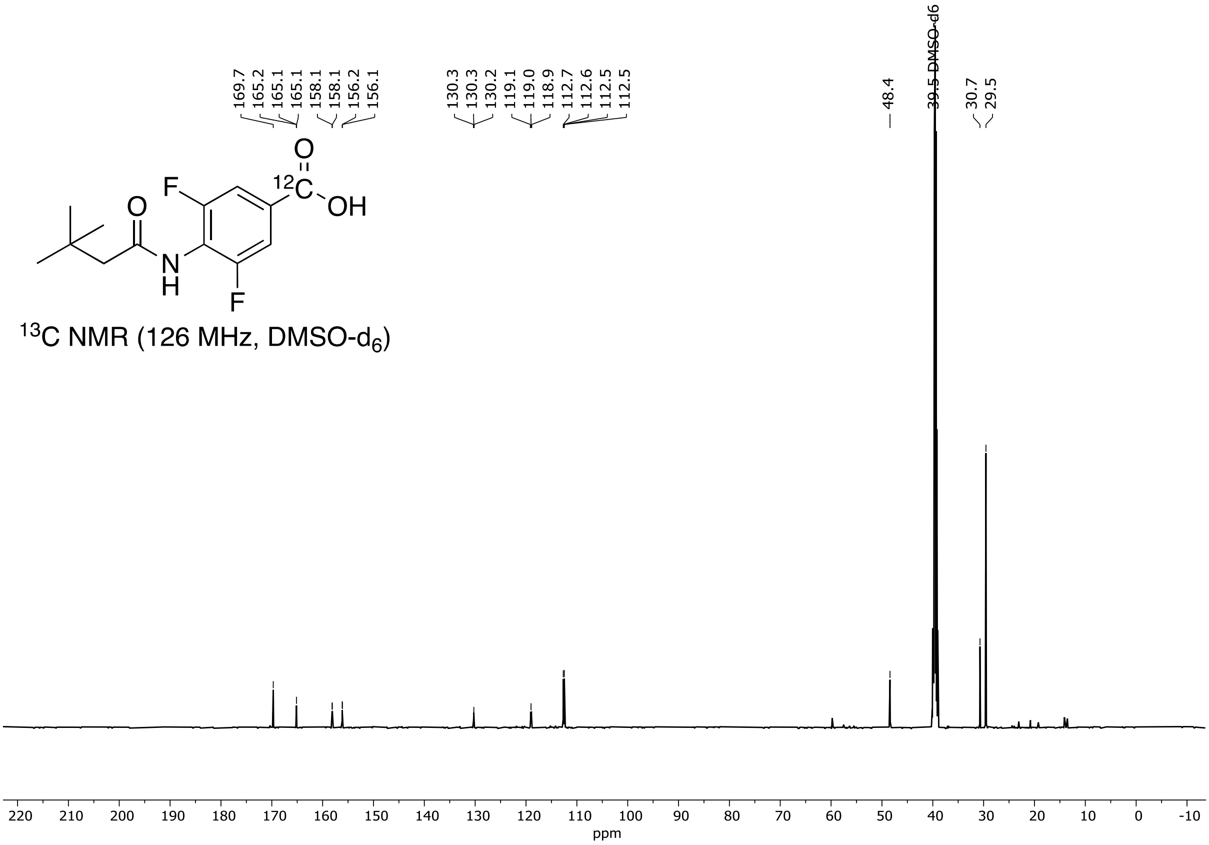
169.7
165.2
165.1
165.1
158.1
158.1
156.2
156.1
130.3
130.3
130.2
119.1
119.0
118.9
112.7
112.6
112.5
112.5
48.4
39.5 DMSO-d6
30.7
29.5
O
F
12C
OH
O
N
H
F
13C NMR (126 MHz, DMSO-d6)
220 210 200 190 180 170 160 150 140 130 120 110 100 90 80 70 60 50 40 30 20 10 0 -10
ppm

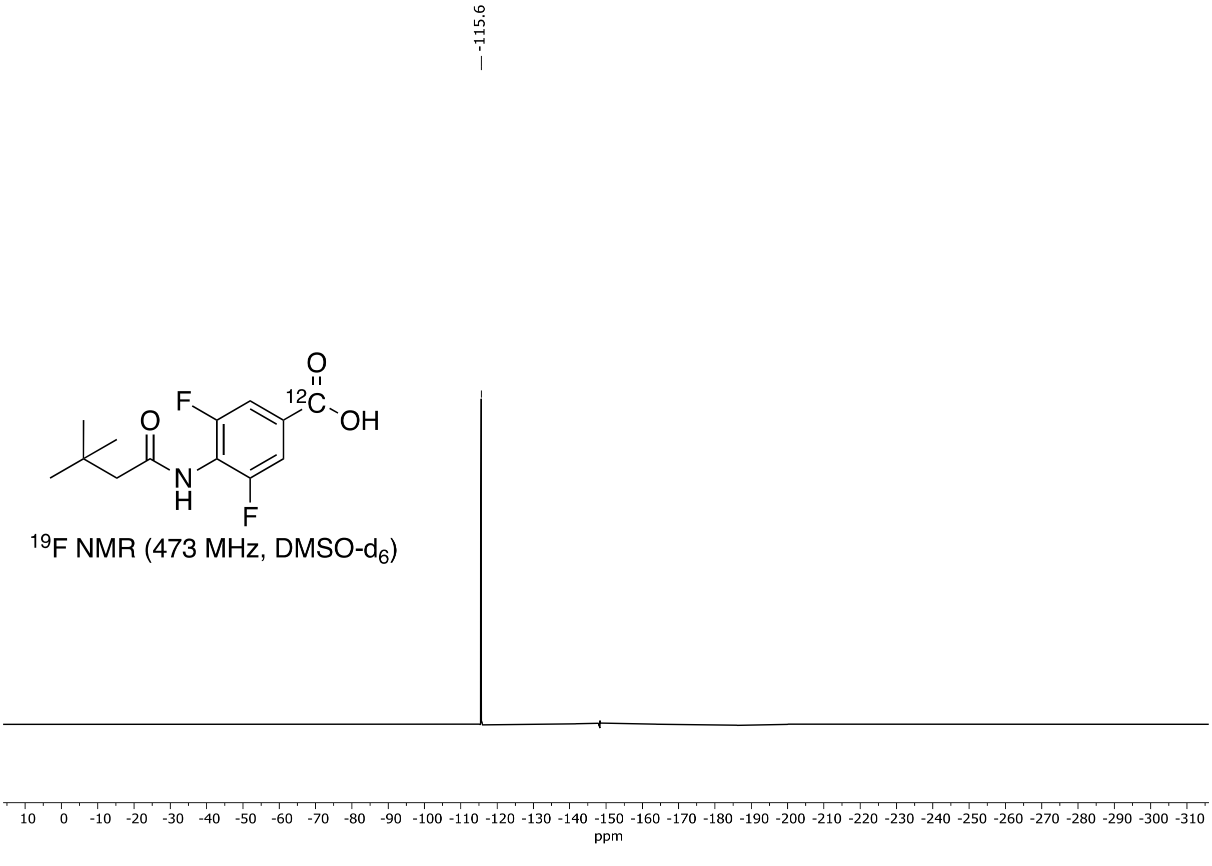
-115.6
O
F
12C
OH
O
N
H
F
19F NMR (473 MHz, DMSO-d6)
10 0 -10 -20 -30 -40 -50 -60 -70 -80 -90 -100 -110 -120 -130 -140 -150 -160 -170 -180 -190 -200 -210 -220 -230 -240 -250 -260 -270 -280 -290 -300 -310
ppm

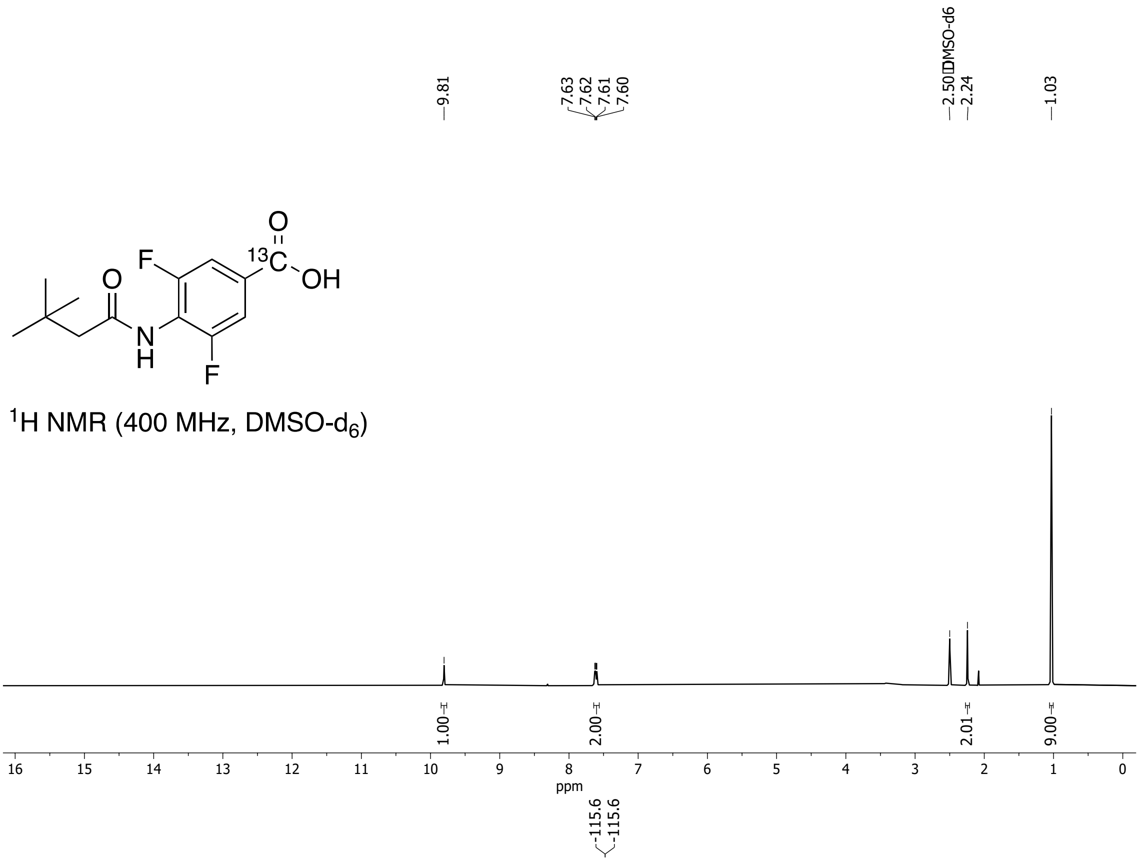
9.81
7.63
7.62
7.61
7.60
2.50 DMSO-d6
2.24
1.03
1H NMR (400 MHz, DMSO-d6)
1.00
2.00
2.01
9.00
16
15
14
13
12
11
10
9
8
7
6
5
4
3
2
1
0
ppm

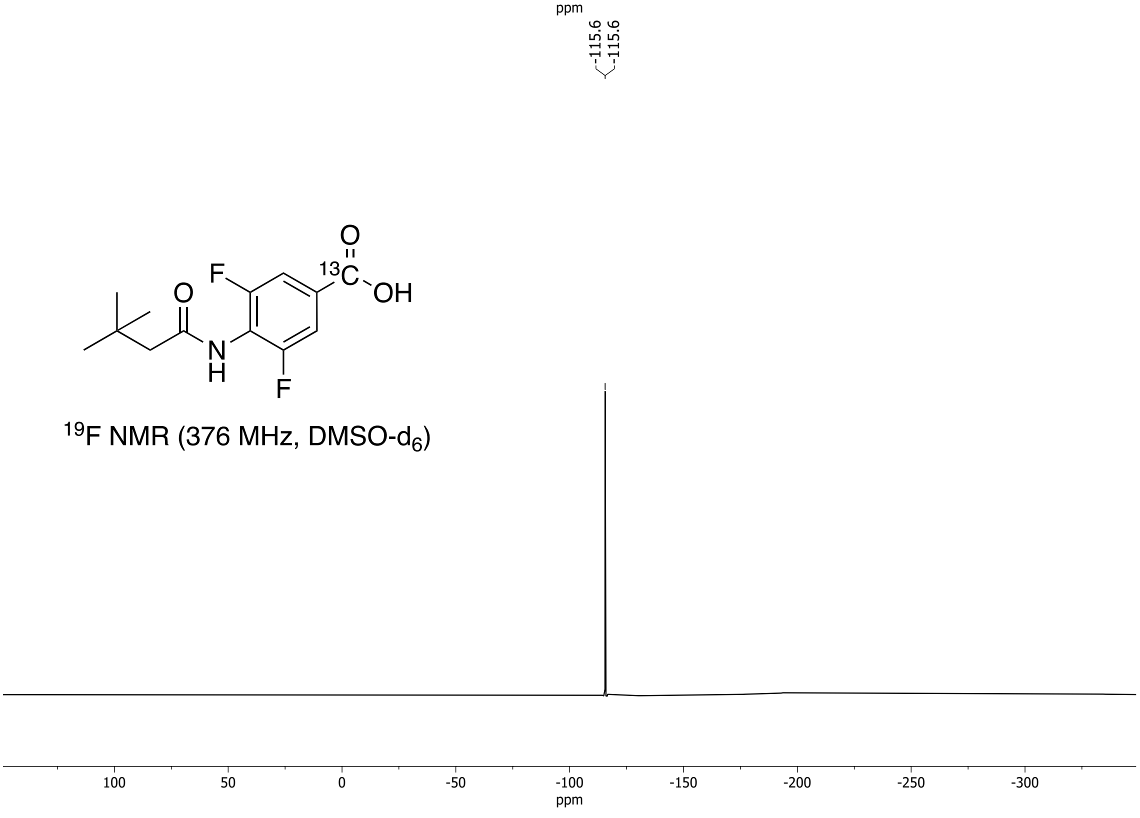
-115.6
-115.6
19F NMR (376 MHz, DMSO-d6)
100
50
0
-50
-100
-150
-200
-250
-300
ppm

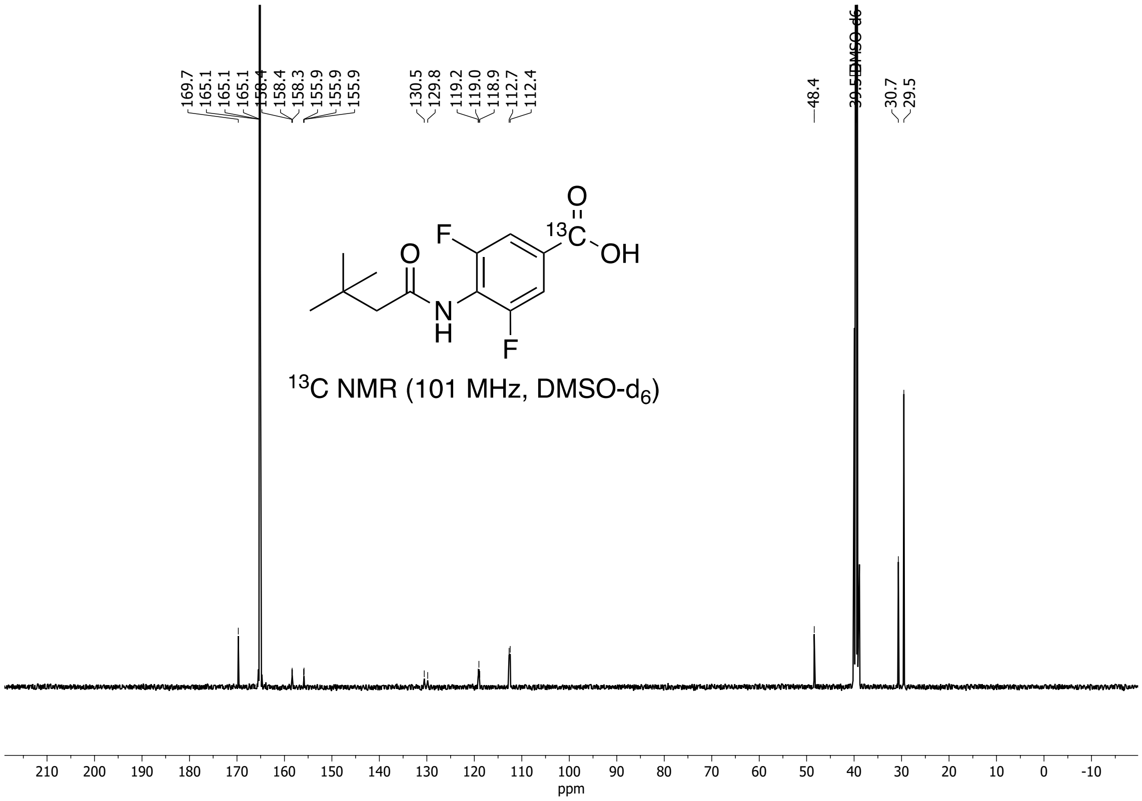
169.7
165.1
165.1
165.1
158.4
158.4
158.3
155.9
155.9
155.9
130.5
129.8
119.2
119.0
118.9
112.7
112.4
48.4
DMSO-d6
30.7
29.5
O
F
13C
OH
O
N
H
F
13C NMR (101 MHz, DMSO-d6)
210 200 190 180 170 160 150 140 130 120 110 100 90 80 70 60 50 40 30 20 10 0 -10
ppm

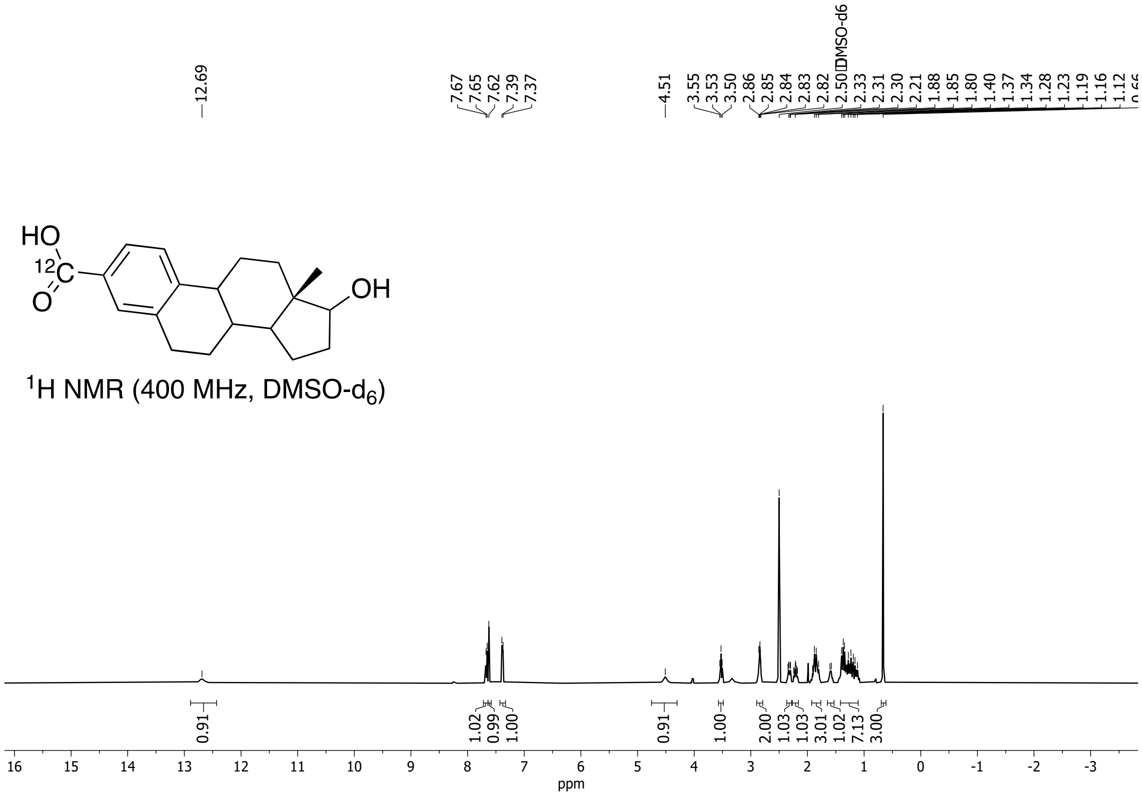
12.69
7.67
7.65
7.62
7.39
7.37
4.51
3.55
3.53
3.50
2.86
2.85
2.84
2.83
2.82
2.50 DMSO-d6
2.33
2.31
2.30
2.21
1.88
1.85
1.80
1.40
1.37
1.34
1.28
1.23
1.19
1.16
1.12
HO
12C
O
OH
1H NMR (400 MHz, DMSO-d6)
0.91
1.02
0.99
1.00
0.91
1.00
2.00
1.03
1.03
3.01
1.02
7.13
3.00
16 15 14 13 12 11 10 9 8 7 6 5 4 3 2 1 0 -1 -2 -3
ppm

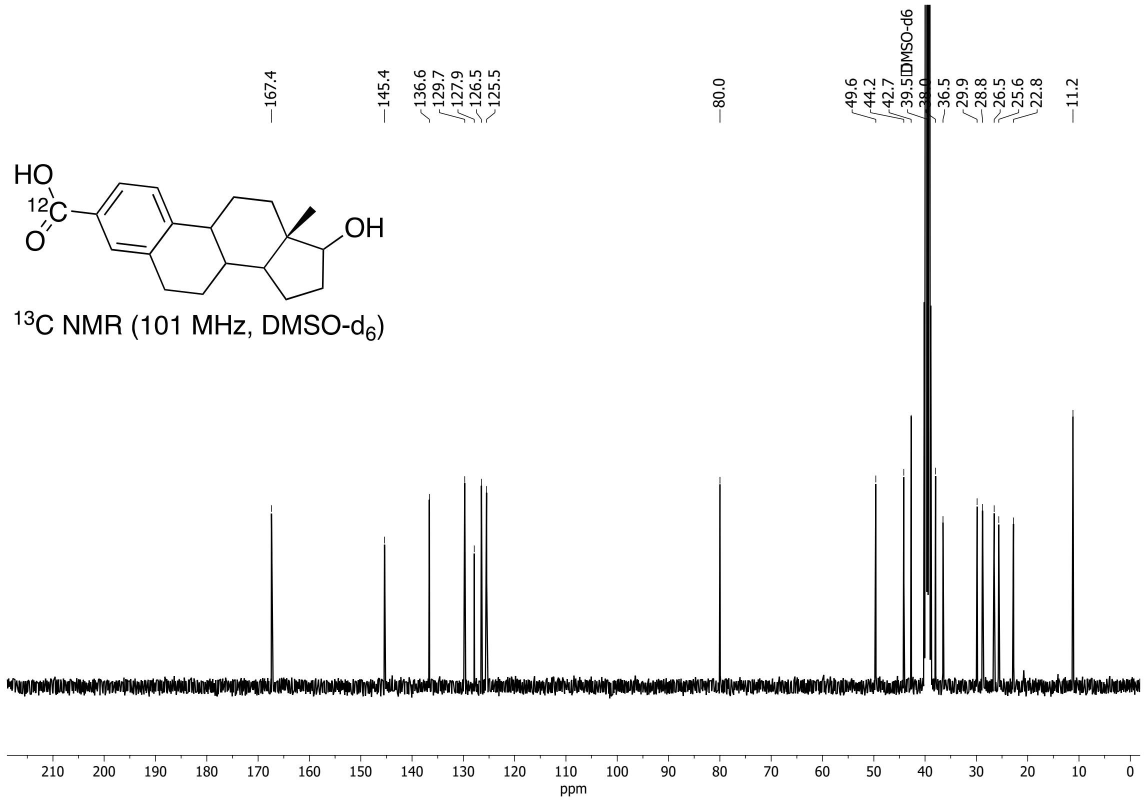
HO
12C
O
OH
13C NMR (101 MHz, DMSO-d6)
167.4
145.4
136.6
129.7
127.9
126.5
125.5
80.0
49.6
44.2
42.7
39.5 DMSO-d6
36.5
29.9
28.8
26.5
25.6
22.8
11.2
ppm

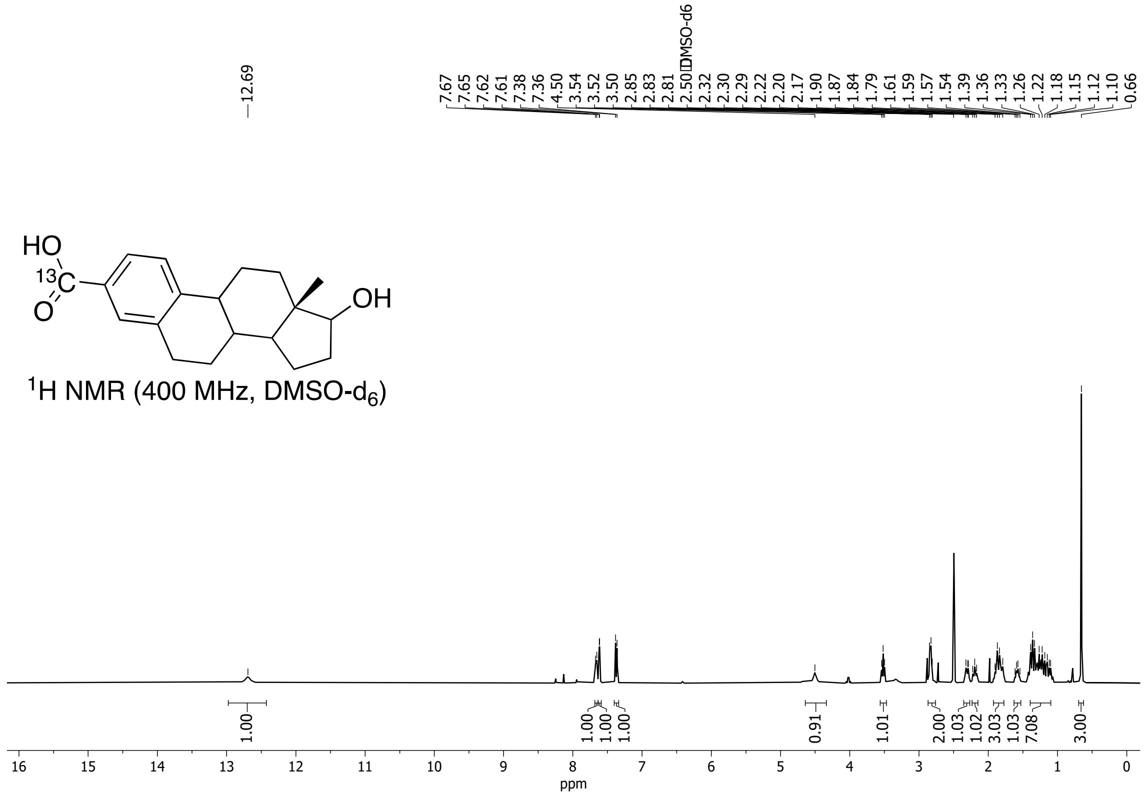
HO
13C
O
OH
1H NMR (400 MHz, DMSO-d6)
12.69
7.67
7.65
7.62
7.61
7.38
7.36
4.50
3.54
3.52
3.50
2.85
2.83
2.81
2.50 DMSO-d6
2.32
2.30
2.29
2.22
2.20
2.17
1.90
1.87
1.84
1.79
1.61
1.59
1.57
1.54
1.39
1.36
1.33
1.26
1.22
1.18
1.15
1.12
1.10
0.66
1.00
1.00
1.00
1.00
0.91
1.01
2.00
1.03
1.02
3.03
1.03
7.08
3.00
ppm

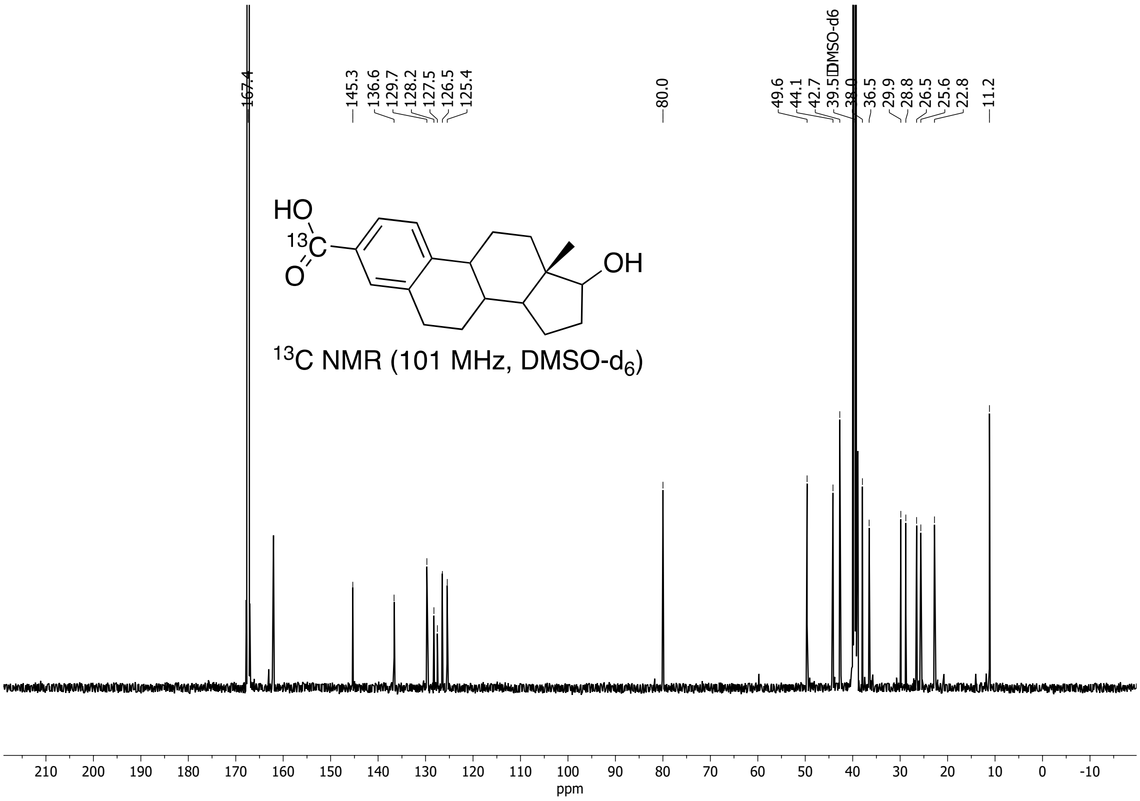
167.4
145.3
136.6
129.7
128.2
127.5
126.5
125.4
80.0
49.6
44.1
42.7
39.5 DMSO-d6
38.0
36.5
29.9
28.8
26.5
25.6
22.8
11.2
HO
13C
O
OH
13C NMR (101 MHz, DMSO-d6)
210 200 190 180 170 160 150 140 130 120 110 100 90 80 70 60 50 40 30 20 10 0 -10
ppm

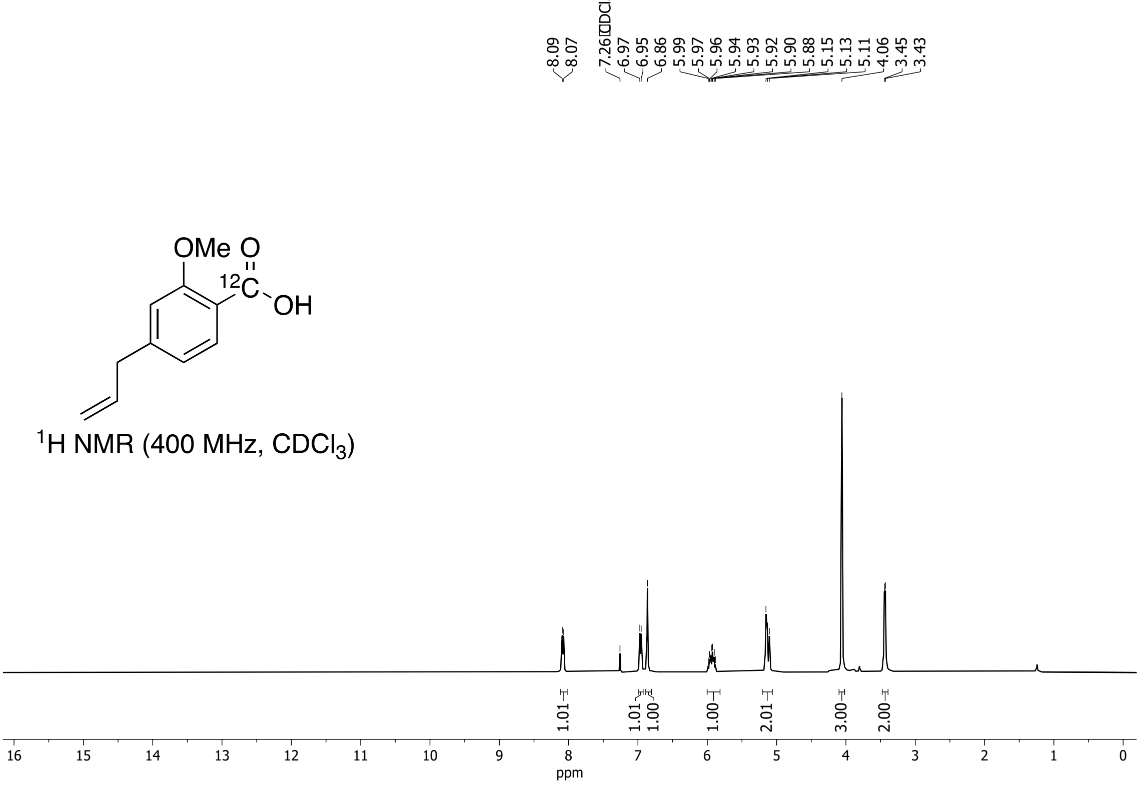
8.09
8.07
7.26 CDCl3
6.97
6.95
6.86
5.99
5.97
5.96
5.94
5.93
5.92
5.90
5.88
5.15
5.13
5.11
4.06
3.45
3.43
OMe
O
12C
OH
1H NMR (400 MHz, CDCl3)
1.01
1.01
1.00
1.00
2.01
3.00
2.00
16 15 14 13 12 11 10 9 8 7 6 5 4 3 2 1 0
ppm

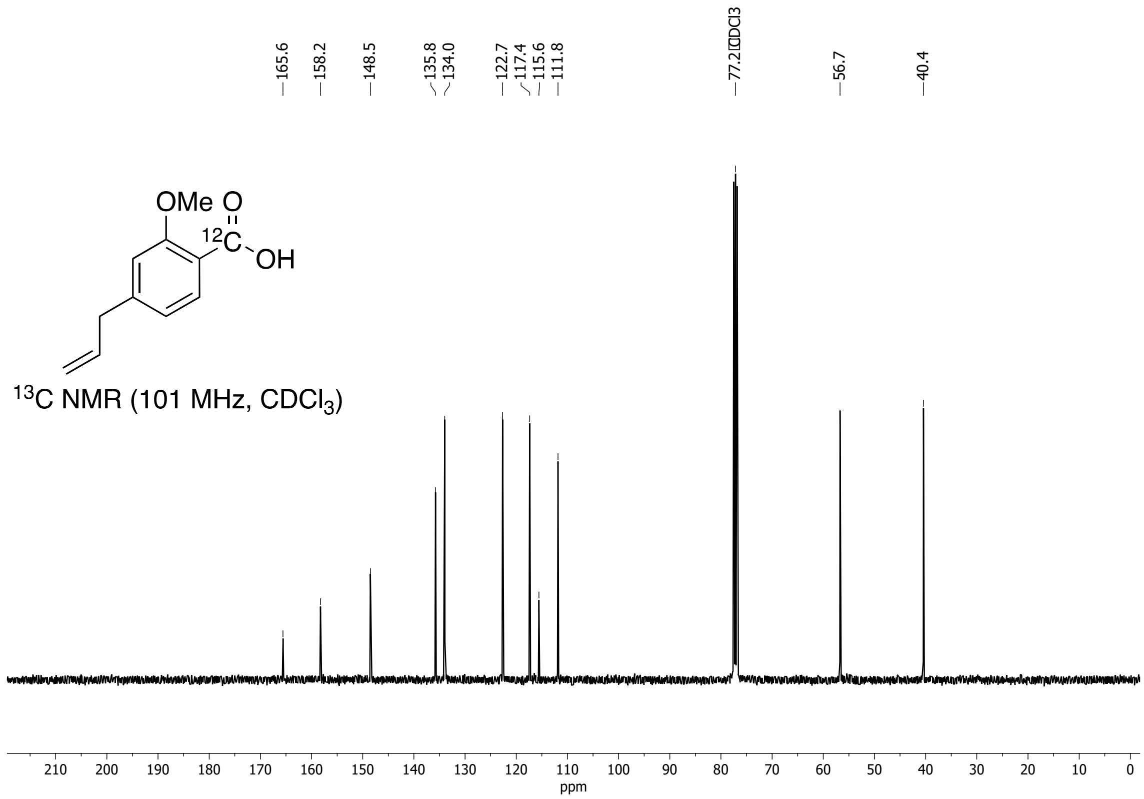
165.6
158.2
148.5
135.8
134.0
122.7
117.4
115.6
111.8
77.2 CDCl3
56.7
40.4
OMe O
12C
OH
13C NMR (101 MHz, CDCl3)
ppm

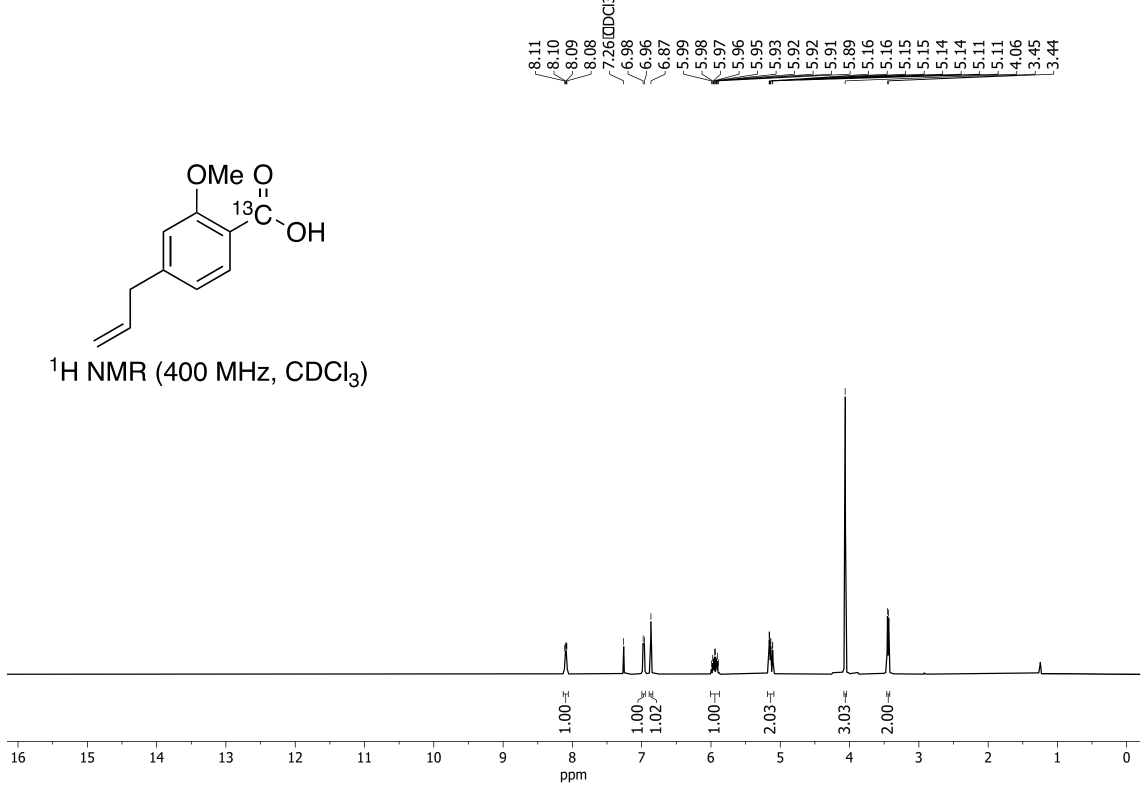
8.11
8.10
8.09
8.08
7.26 CDCl3
6.98
6.96
6.87
5.99
5.98
5.97
5.96
5.95
5.93
5.92
5.92
5.91
5.89
5.16
5.16
5.15
5.15
5.14
5.14
5.11
5.11
4.06
3.45
3.44
OMe O
13C
OH
1H NMR (400 MHz, CDCl3)
1.00
1.00
1.02
1.00
2.03
3.03
2.00
ppm

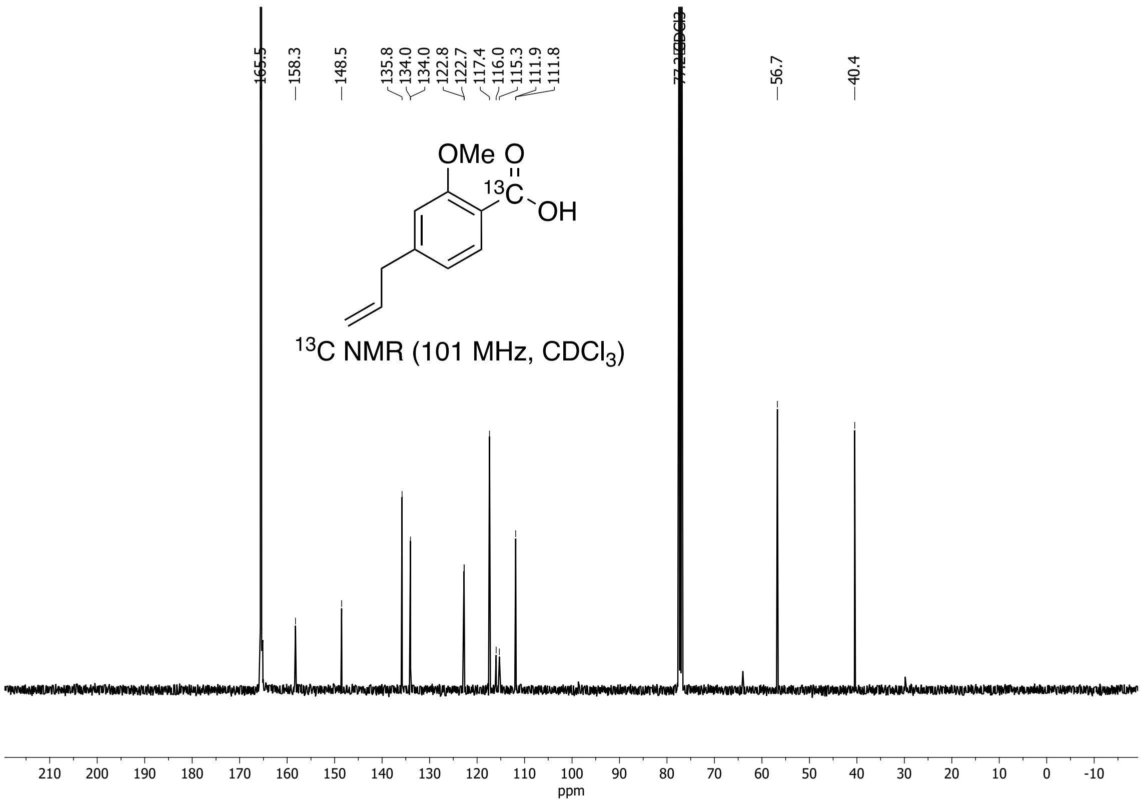
165.5
158.3
148.5
135.8
134.0
134.0
122.8
122.7
117.4
116.0
115.3
111.9
111.8
77.2 CDCl3
56.7
40.4
OMe O
13C
OH
13C NMR (101 MHz, CDCl3)
ppm

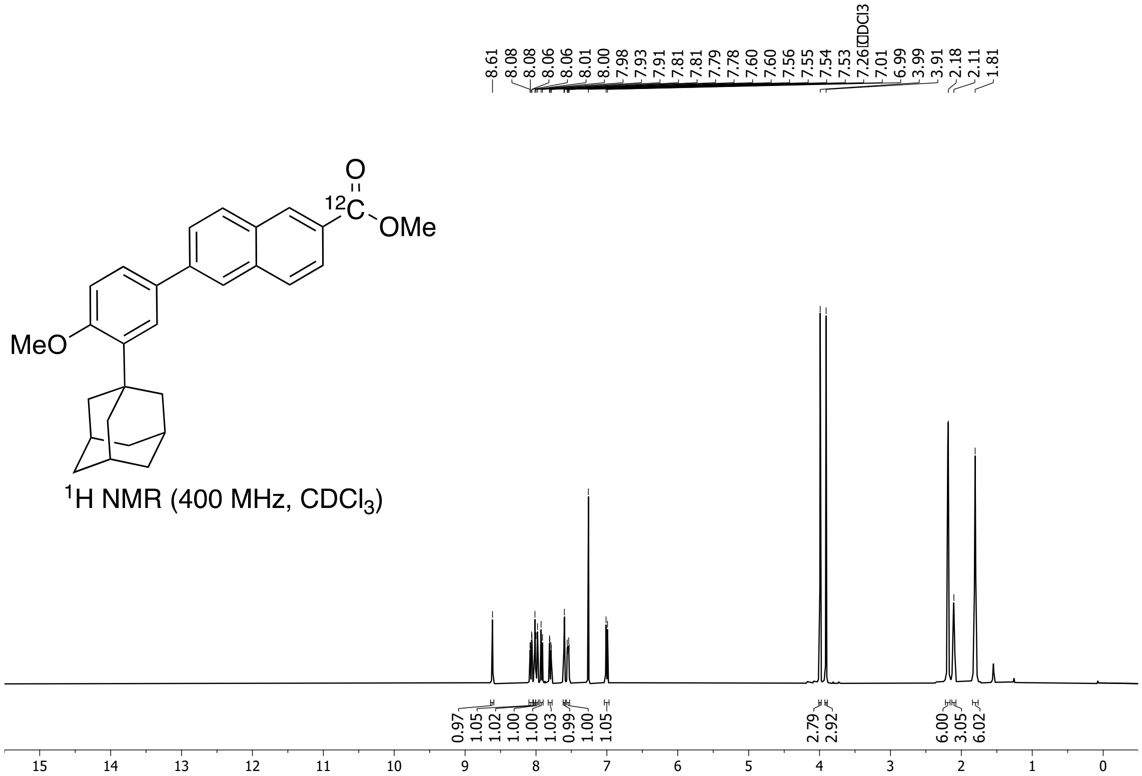
8.61
8.08
8.08
8.06
8.06
8.01
8.00
7.98
7.93
7.91
7.81
7.81
7.79
7.78
7.60
7.60
7.56
7.55
7.54
7.53
7.26 CDCl3
7.01
6.99
3.99
3.91
2.18
2.11
1.81
O
12C
OMe
MeO
1H NMR (400 MHz, CDCl3)
0.97
1.05
1.02
1.00
1.00
1.03
0.99
1.00
1.05
2.79
2.92
6.00
3.05
6.02
ppm

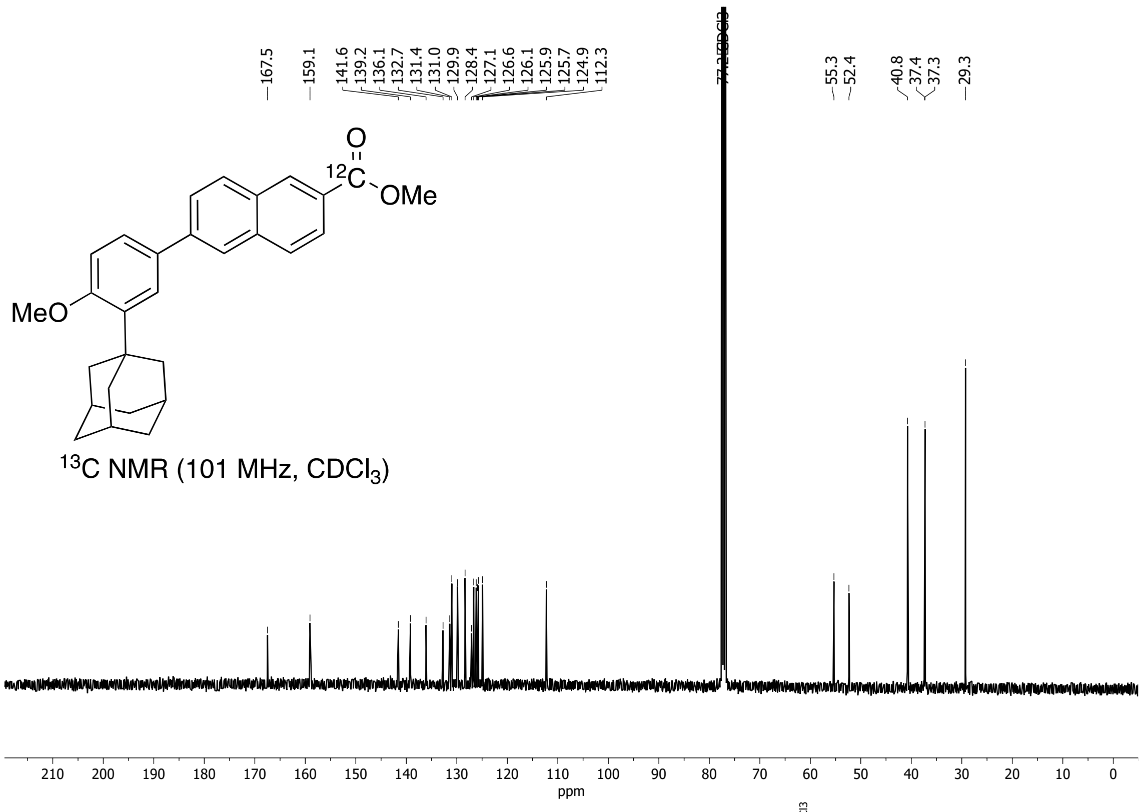

O
12C
OMe
MeO
13C NMR (101 MHz, CDCl3)
ppm

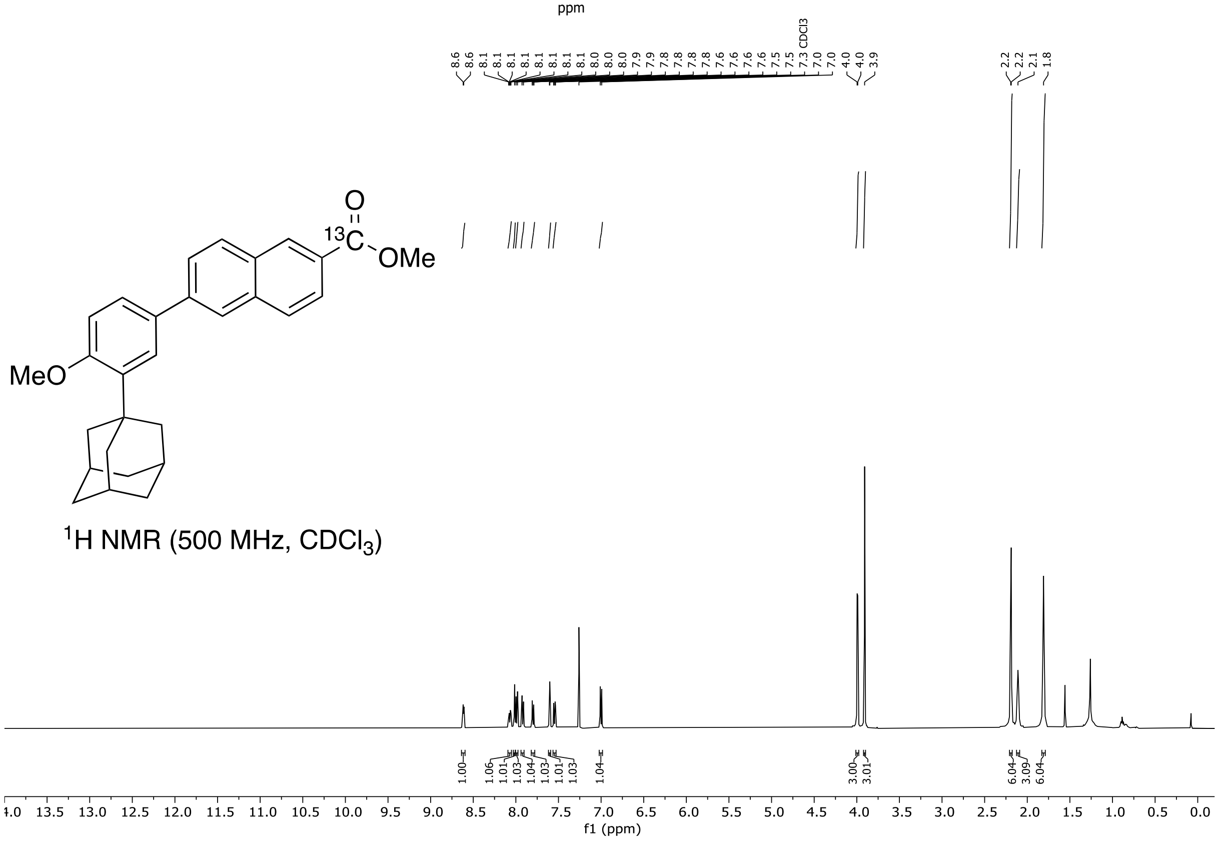

O
13C
OMe
MeO
1H NMR (500 MHz, CDCl3)
f1 (ppm)

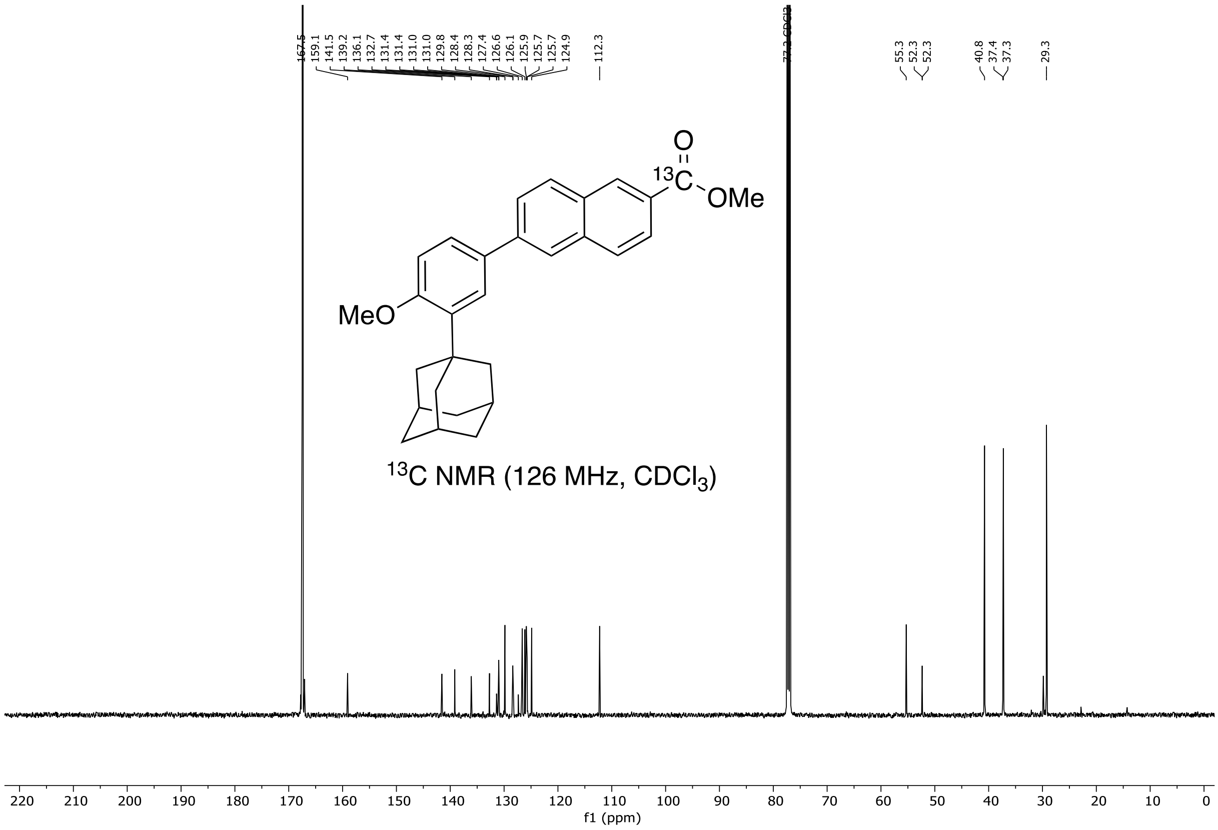

O
13C
OMe
MeO
13C NMR (126 MHz, CDCl3)
f1 (ppm)

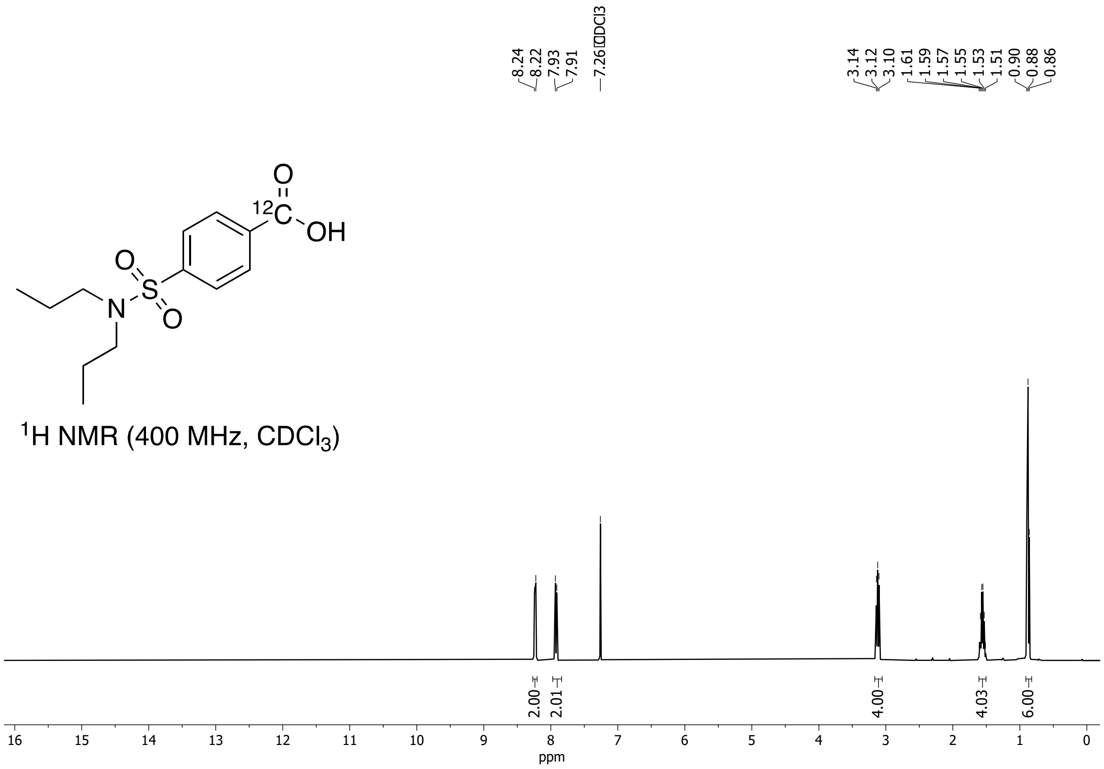

O
12C
OH
O
S
N
O
1H NMR (400 MHz, CDCl3)
ppm

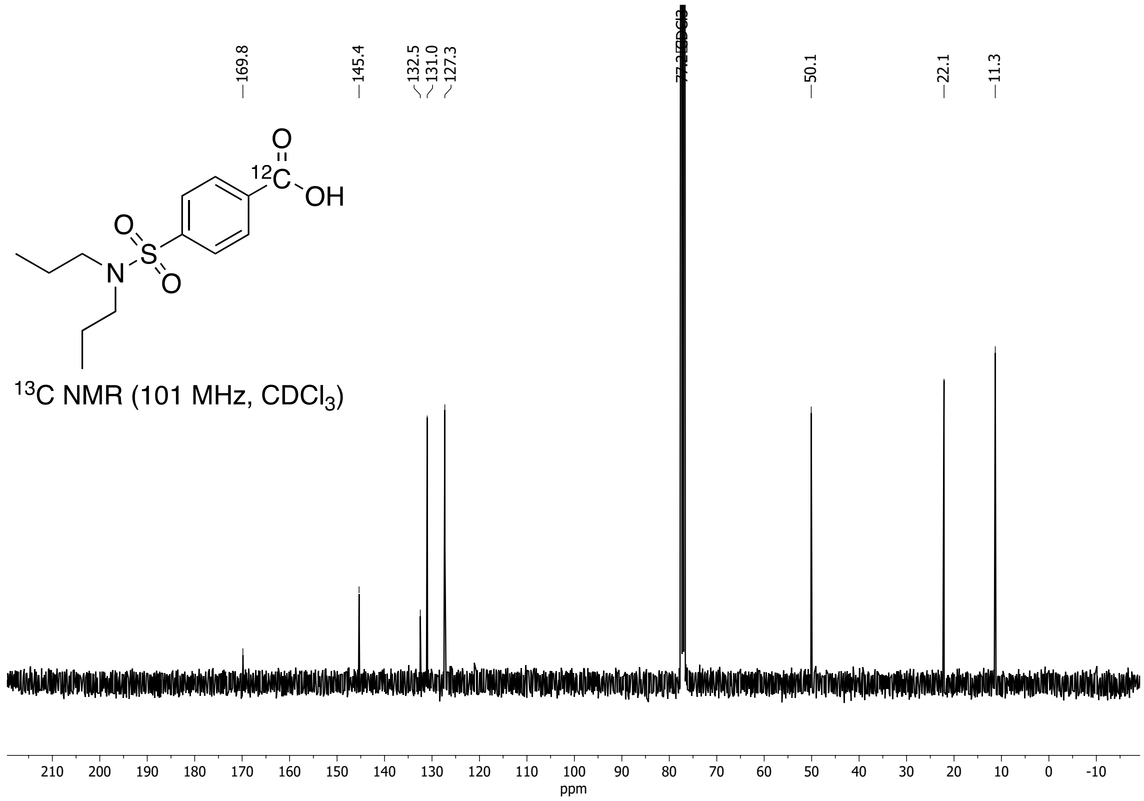

^{13}C NMR (101 MHz, $CDCl_3$)

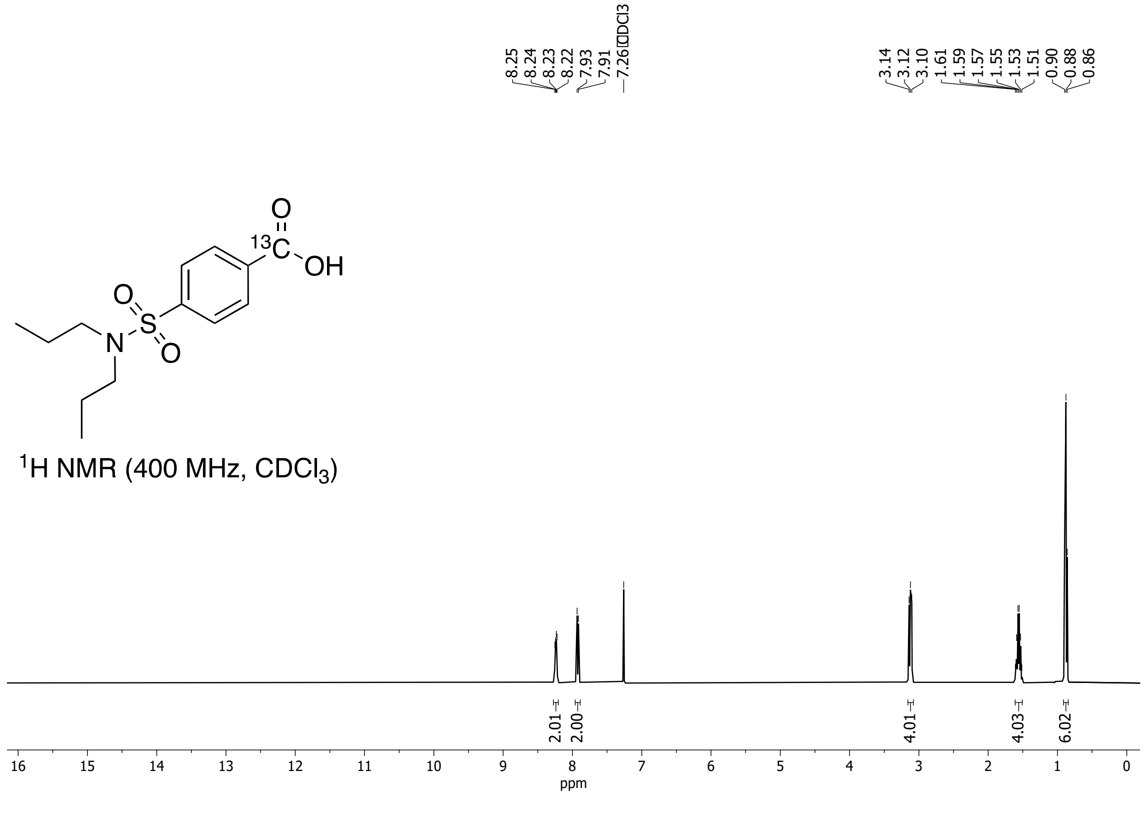

^{1}H NMR (400 MHz, $CDCl_3$)

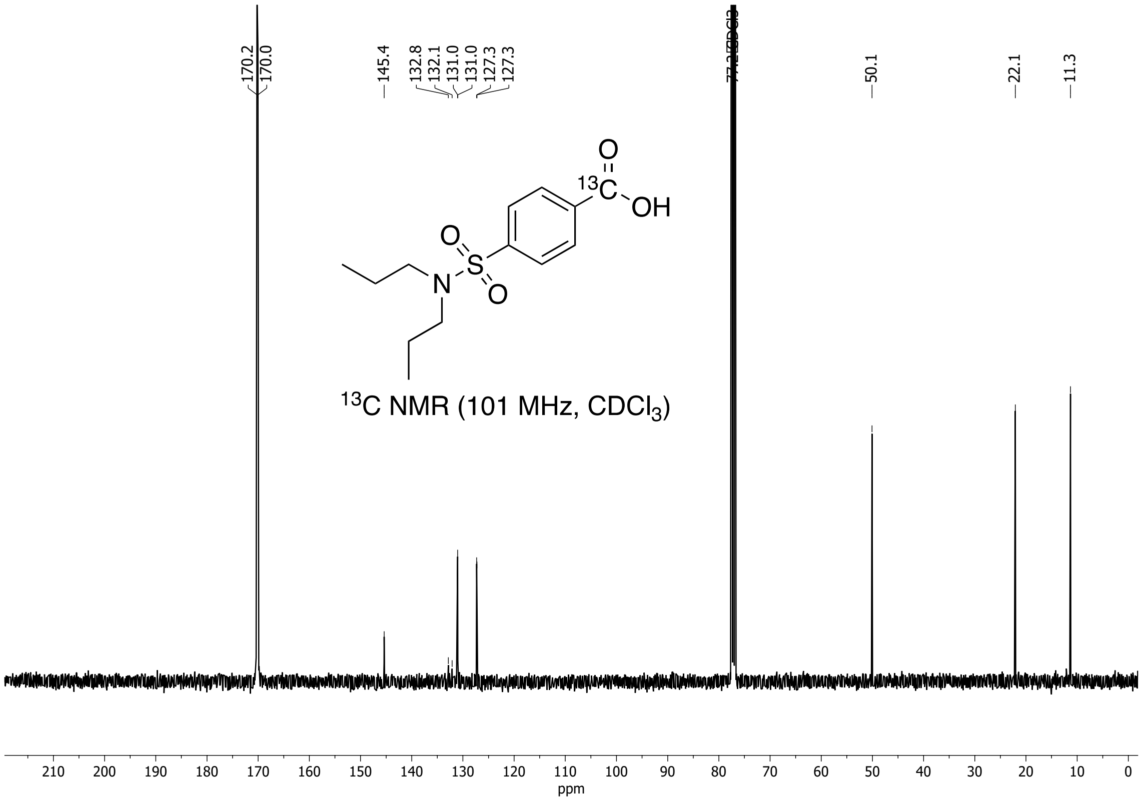

170.2
170.0
145.4
132.8
132.1
131.0
131.0
127.3
127.3
50.1
22.1
11.3
O
13C
OH
O
S
N
O
13C NMR (101 MHz, CDCl3)
210 200 190 180 170 160 150 140 130 120 110 100 90 80 70 60 50 40 30 20 10 0
ppm

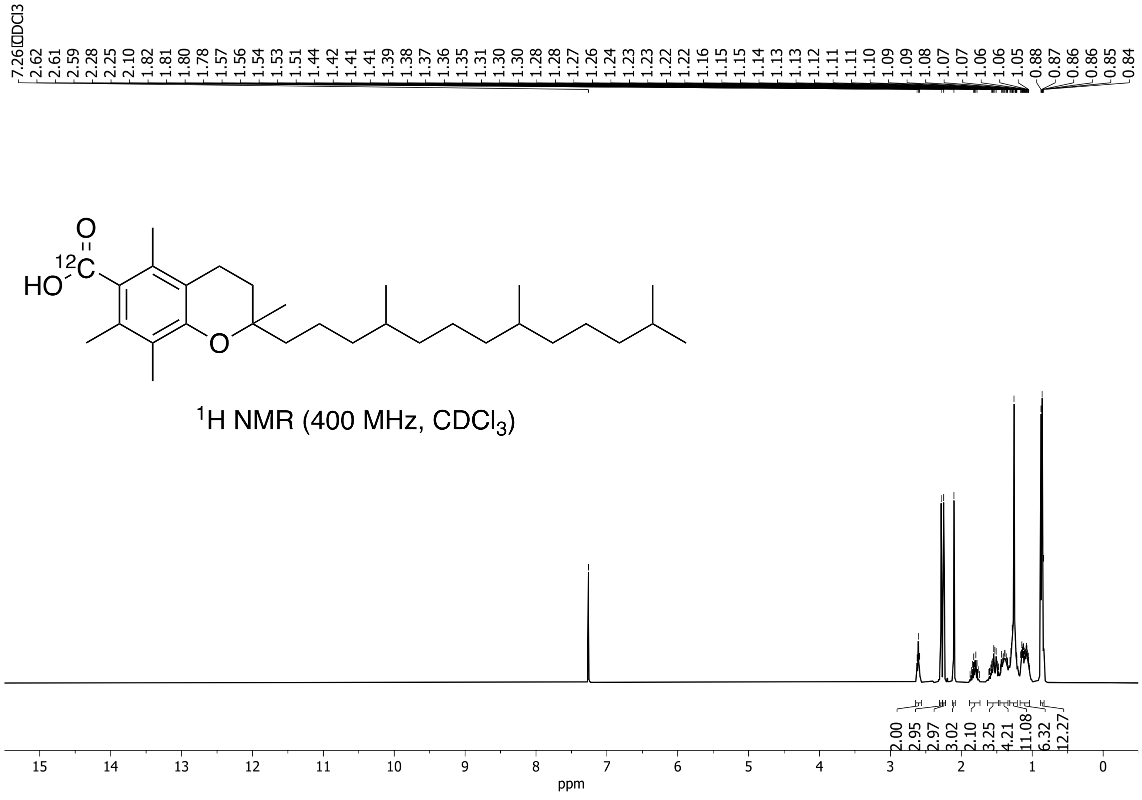

7.26 CDCl3
2.62
2.61
2.59
2.28
2.25
2.10
1.82
1.81
1.80
1.78
1.57
1.56
1.54
1.53
1.51
1.44
1.42
1.41
1.39
1.38
1.37
1.36
1.35
1.31
1.30
1.30
1.28
1.28
1.27
1.26
1.24
1.23
1.23
1.22
1.22
1.16
1.15
1.15
1.14
1.13
1.13
1.12
1.11
1.11
1.10
1.09
1.09
1.08
1.07
1.07
1.06
1.06
1.05
0.88
0.87
0.86
0.86
0.85
0.84
O
12C
HO
O
1H NMR (400 MHz, CDCl3)
2.00
2.95
2.97
3.02
2.10
3.25
4.21
11.08
6.32
12.27
15 14 13 12 11 10 9 8 7 6 5 4 3 2 1 0
ppm

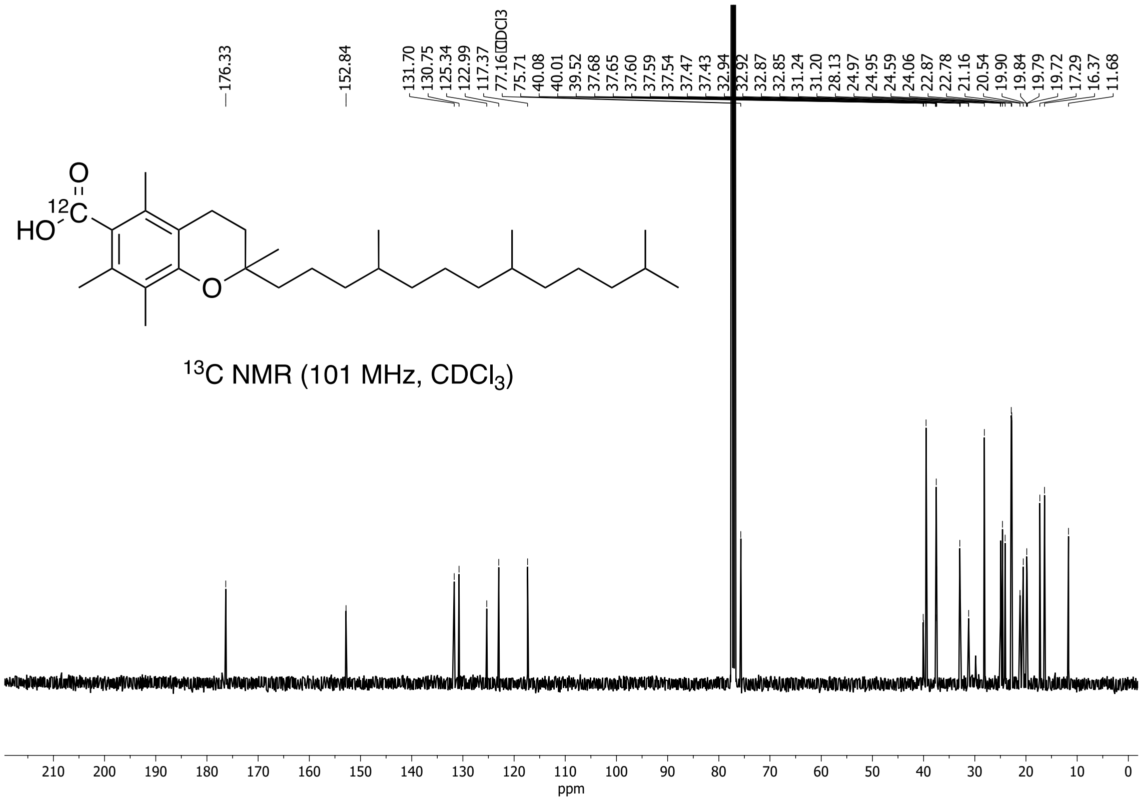
176.33
152.84
131.70
130.75
125.34
122.99
117.37
77.16 CDCl3
75.71
40.08
40.01
39.52
37.68
37.65
37.60
37.59
37.54
37.47
37.43
32.94
32.92
32.87
32.85
31.24
31.20
28.13
24.97
24.95
24.59
24.06
22.87
22.78
21.16
20.54
19.90
19.84
19.79
19.72
17.29
16.37
11.68
O
12C
HO
O
13C NMR (101 MHz, CDCl3)
ppm

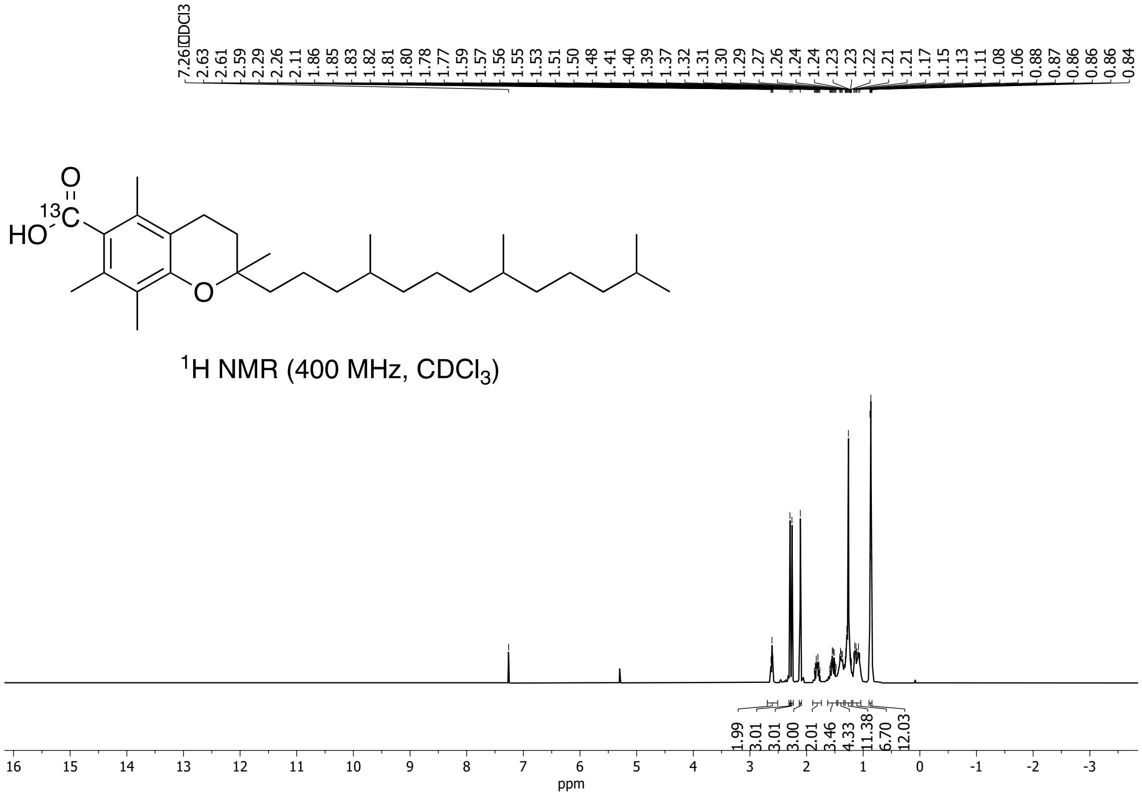
7.26 CDCl3
2.63
2.61
2.59
2.29
2.26
2.11
1.86
1.85
1.83
1.82
1.81
1.80
1.78
1.77
1.59
1.57
1.56
1.55
1.53
1.51
1.50
1.48
1.41
1.40
1.39
1.37
1.32
1.31
1.30
1.29
1.27
1.26
1.24
1.24
1.23
1.23
1.22
1.21
1.21
1.17
1.15
1.13
1.11
1.08
1.06
0.88
0.87
0.86
0.86
0.86
0.84
O
13C
HO
O
1H NMR (400 MHz, CDCl3)
1.99
3.01
3.01
3.00
2.01
3.46
4.33
11.38
6.70
12.03
ppm

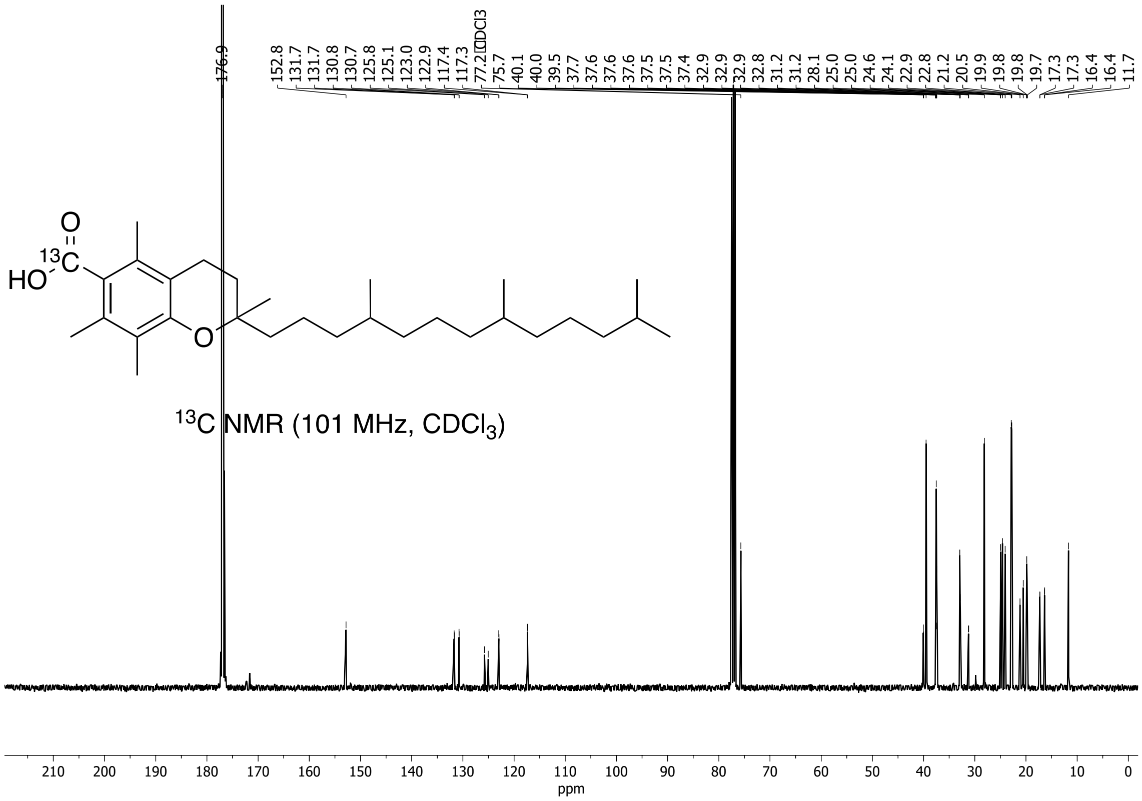

176.9
152.8
131.7
131.7
130.8
130.7
125.8
125.1
123.0
122.9
117.4
117.3
77.2 CDCl3
75.7
40.1
40.0
39.5
37.7
37.6
37.6
37.6
37.5
37.5
37.4
32.9
32.9
32.9
32.8
31.2
31.2
28.1
25.0
25.0
24.6
24.1
22.9
22.8
21.2
20.5
19.9
19.8
19.8
19.7
17.3
17.3
16.4
16.4
11.7
O
^{13}C
HO
O
^{13}C NMR (101 MHz, CDCl$_3$)
210 200 190 180 170 160 150 140 130 120 110 100 90 80 70 60 50 40 30 20 10 0
ppm

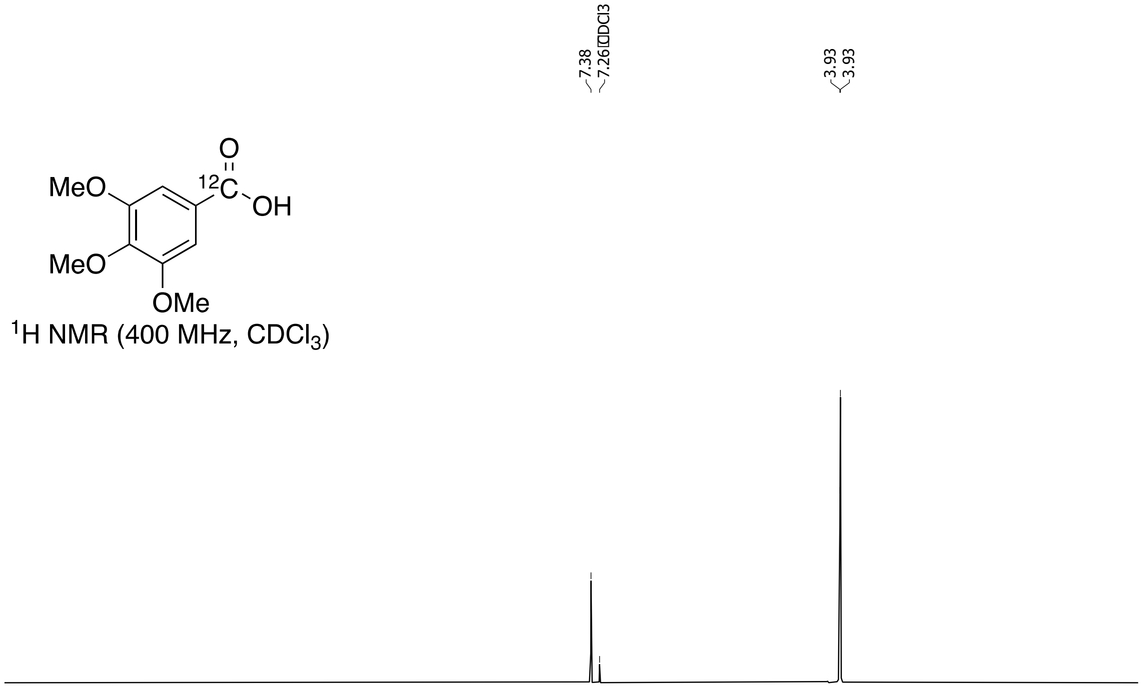

7.38
7.26 CDCl3
3.93
3.93
O
MeO
^{12}C
OH
MeO
OMe
^{1}H NMR (400 MHz, CDCl$_3$)
2.00
3.04
6.02
15 14 13 12 11 10 9 8 7 6 5 4 3 2 1 0
ppm

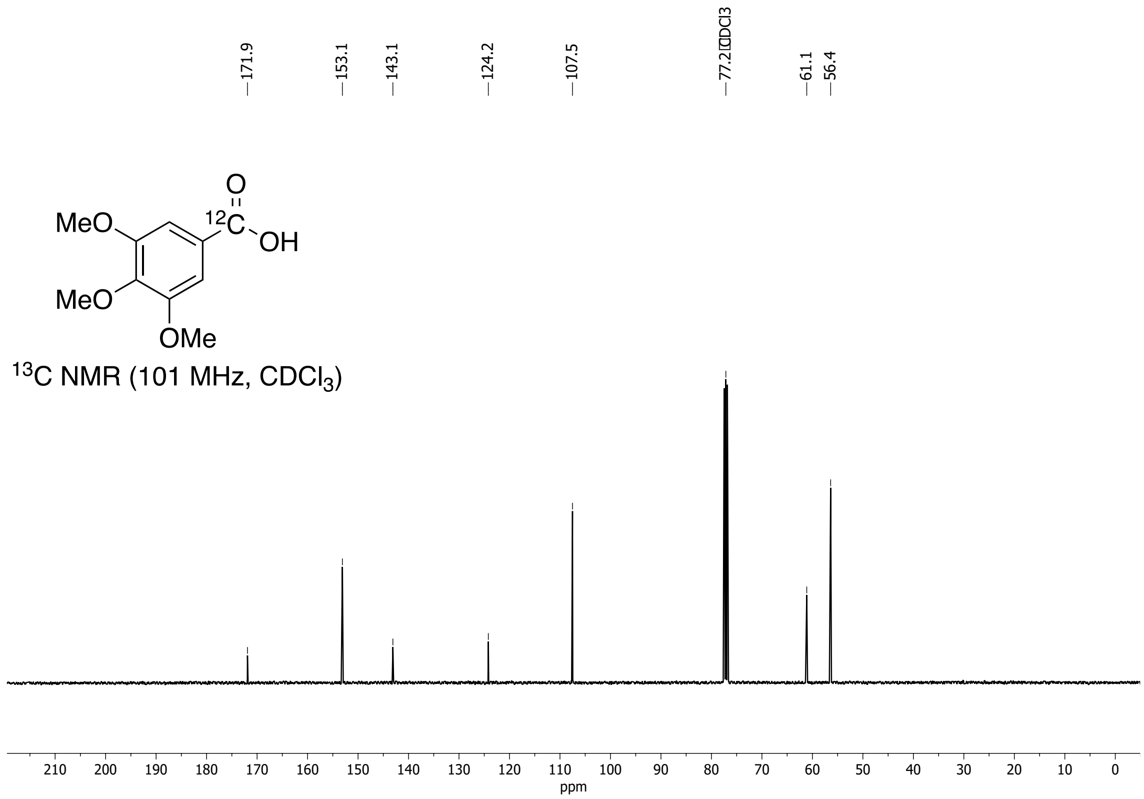
171.9
153.1
143.1
124.2
107.5
77.2 CDCl3
61.1
56.4
MeO
MeO
OMe
12C
O
OH
^{13}C NMR (101 MHz, CDCl$_3$)
210 200 190 180 170 160 150 140 130 120 110 100 90 80 70 60 50 40 30 20 10 0
ppm

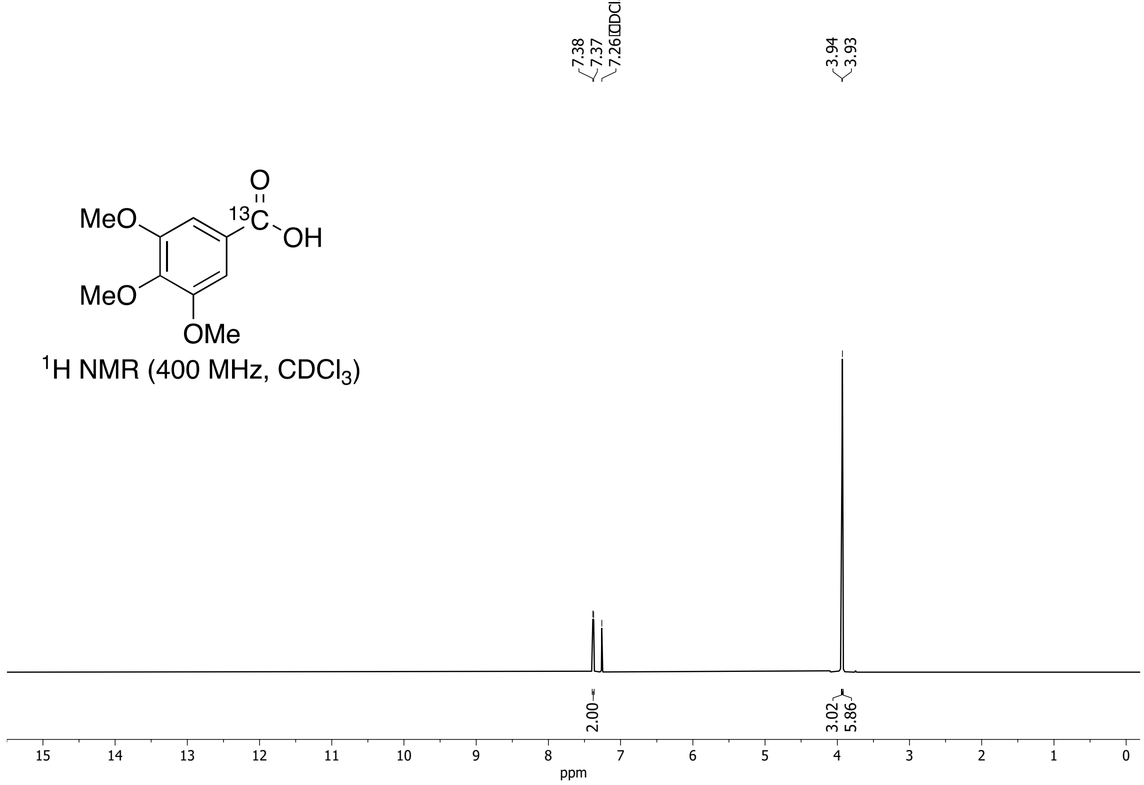
7.38
7.37
7.26 CDCl3
3.94
3.93
MeO
MeO
OMe
13C
O
OH
^{1}H NMR (400 MHz, CDCl$_3$)
2.00
3.02
5.86
15 14 13 12 11 10 9 8 7 6 5 4 3 2 1 0
ppm

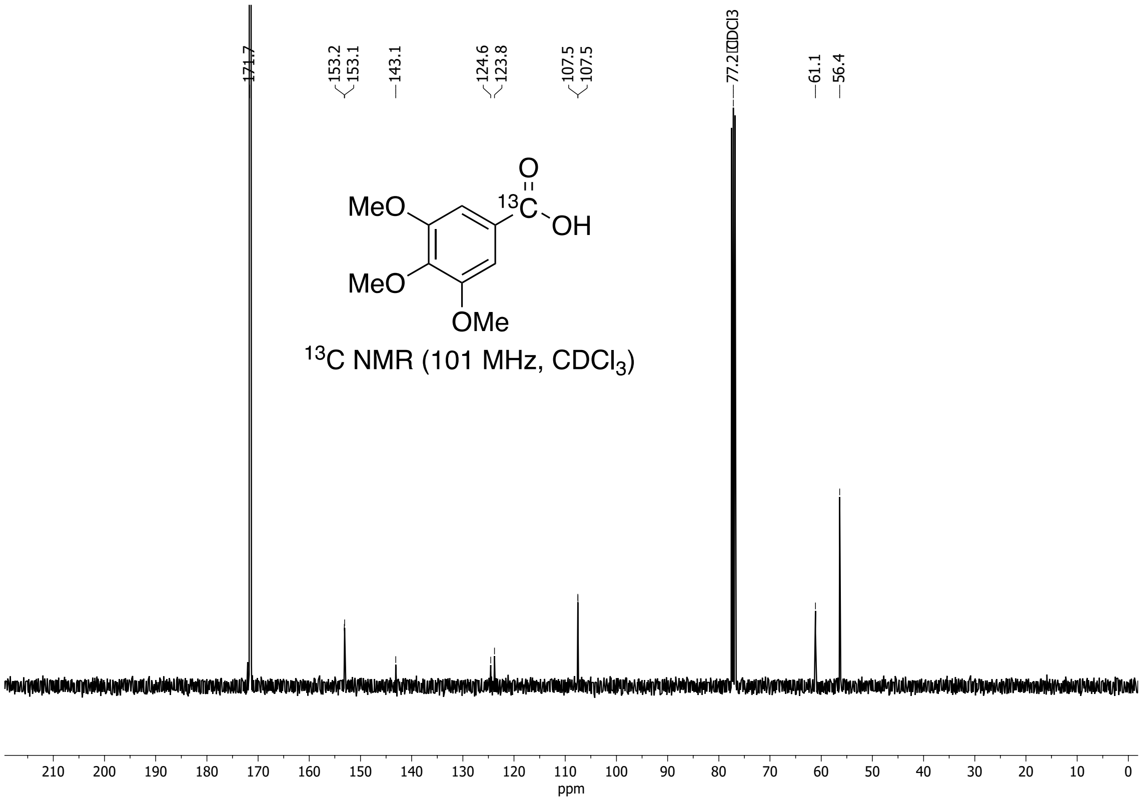
171.7
153.2
153.1
143.1
124.6
123.8
107.5
107.5
77.2 CDCl3
61.1
56.4
MeO
MeO
OMe
^{13}C
OH
O
^{13}C NMR (101 MHz, CDCl$_3$)
ppm

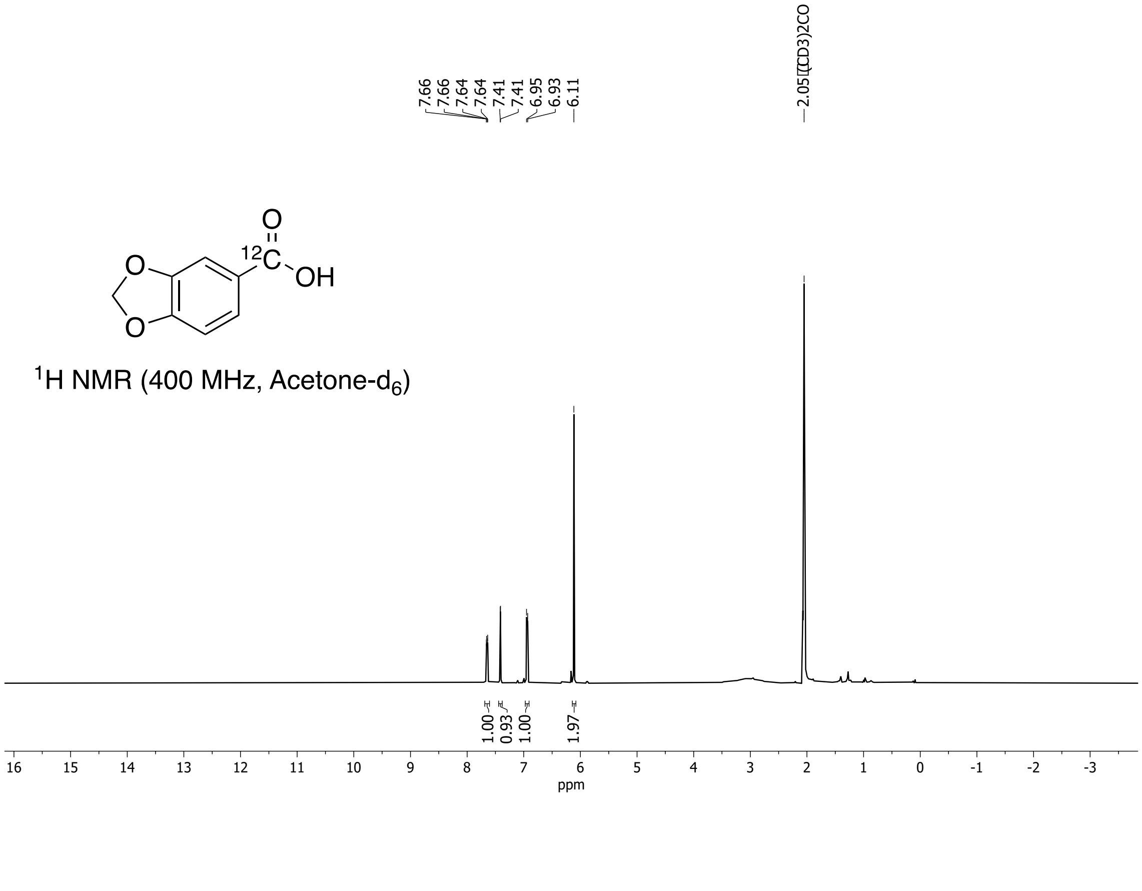
7.66
7.66
7.64
7.64
7.41
7.41
6.95
6.93
6.11
2.05 (CD3)2CO
O
O
^{12}C
OH
O
^{1}H NMR (400 MHz, Acetone-d$_6$)
1.00
0.93
1.00
1.97
ppm

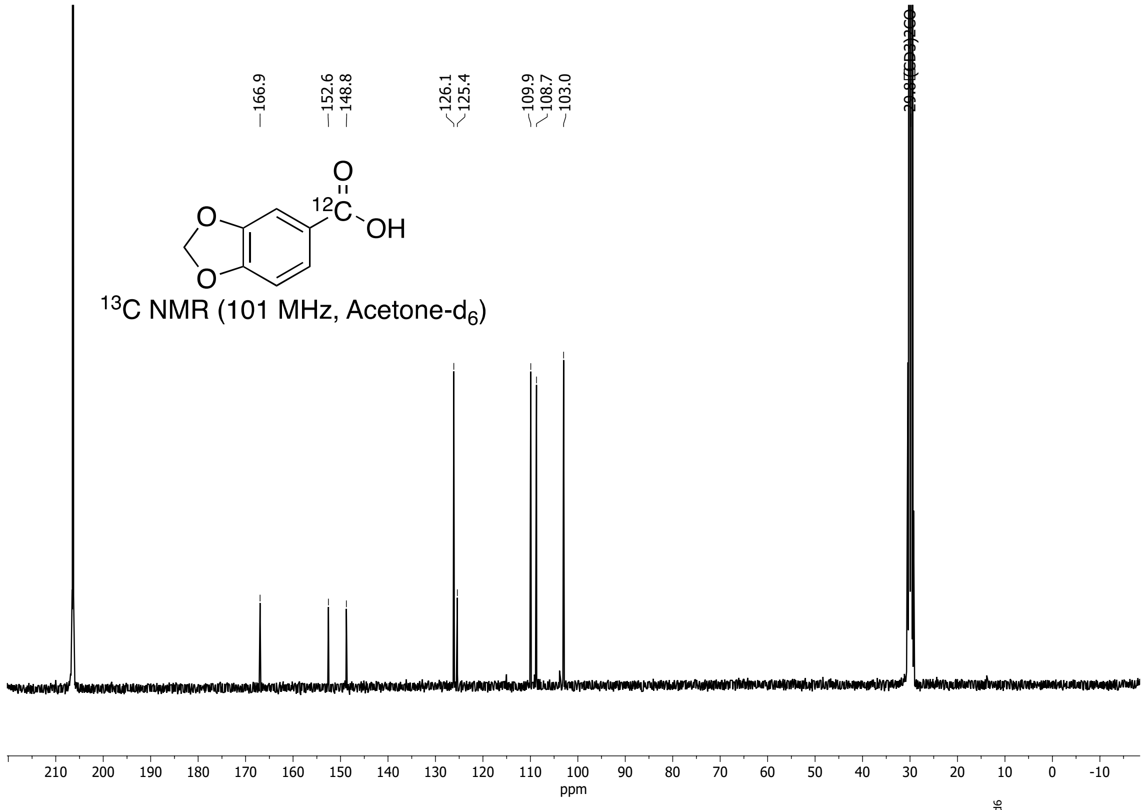

166.9
152.6
148.8
126.1
125.4
109.9
108.7
103.0
O
^{12}C
OH
O
O
^{13}C NMR (101 MHz, Acetone-d$_6$)
210 200 190 180 170 160 150 140 130 120 110 100 90 80 70 60 50 40 30 20 10 0 -10
ppm

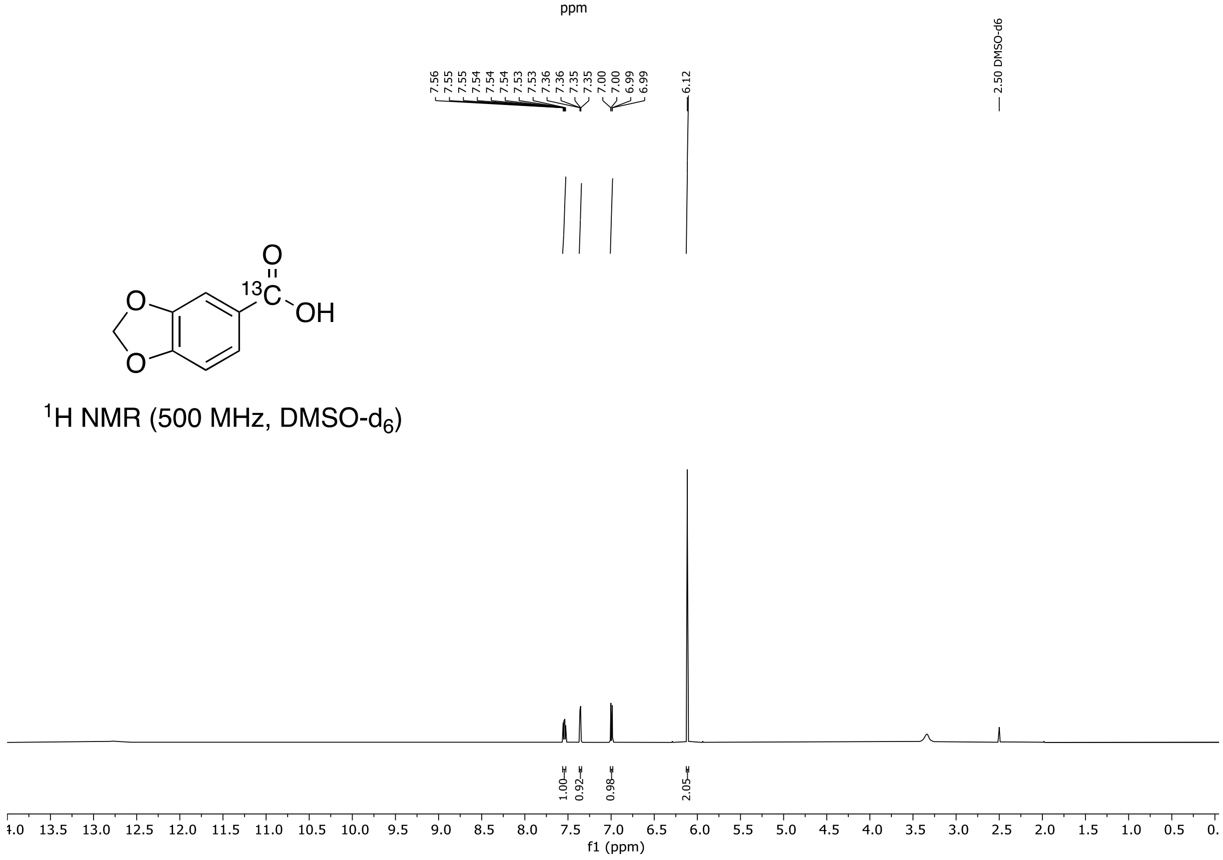

7.56 7.55 7.55 7.54 7.54 7.54 7.53 7.53 7.36 7.36 7.35 7.35 7.00 7.00 6.99 6.99
6.12
2.50 DMSO-d6
O
^{13}C
OH
O
O
^{1}H NMR (500 MHz, DMSO-d$_6$)
1.00
0.92
0.98
2.05
13.5 13.0 12.5 12.0 11.5 11.0 10.5 10.0 9.5 9.0 8.5 8.0 7.5 7.0 6.5 6.0 5.5 5.0 4.5 4.0 3.5 3.0 2.5 2.0 1.5 1.0 0.5
f1 (ppm)

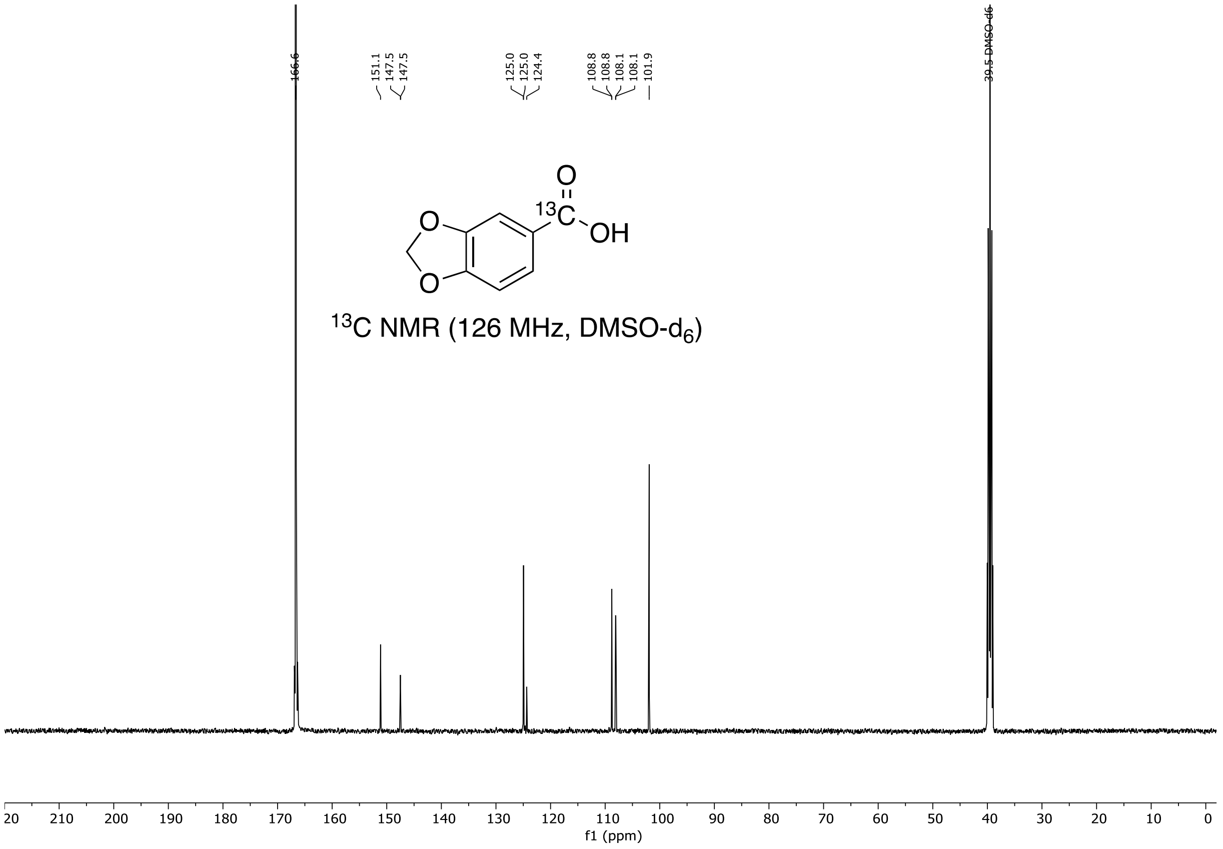
166.6
151.1
147.5
147.5
125.0
125.0
124.4
108.8
108.8
108.1
108.1
101.9
39.5 DMSO-d6
13C NMR (126 MHz, DMSO-d6)
f1 (ppm)

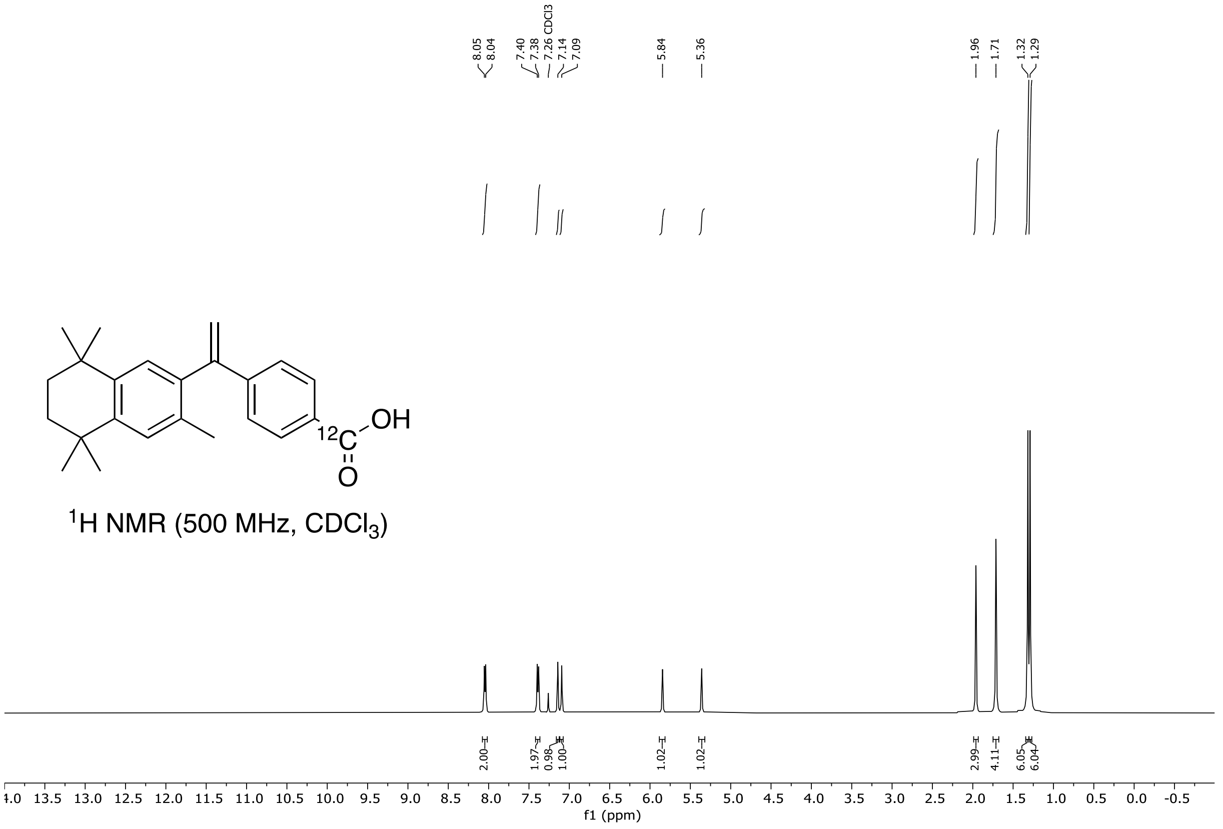
8.05
8.04
7.40
7.38
7.26 CDCl3
7.14
7.09
5.84
5.36
1.96
1.71
1.32
1.29
1H NMR (500 MHz, CDCl3)
2.00
1.97
0.98
1.00
1.02
1.02
2.99
4.11
6.05
6.04
f1 (ppm)

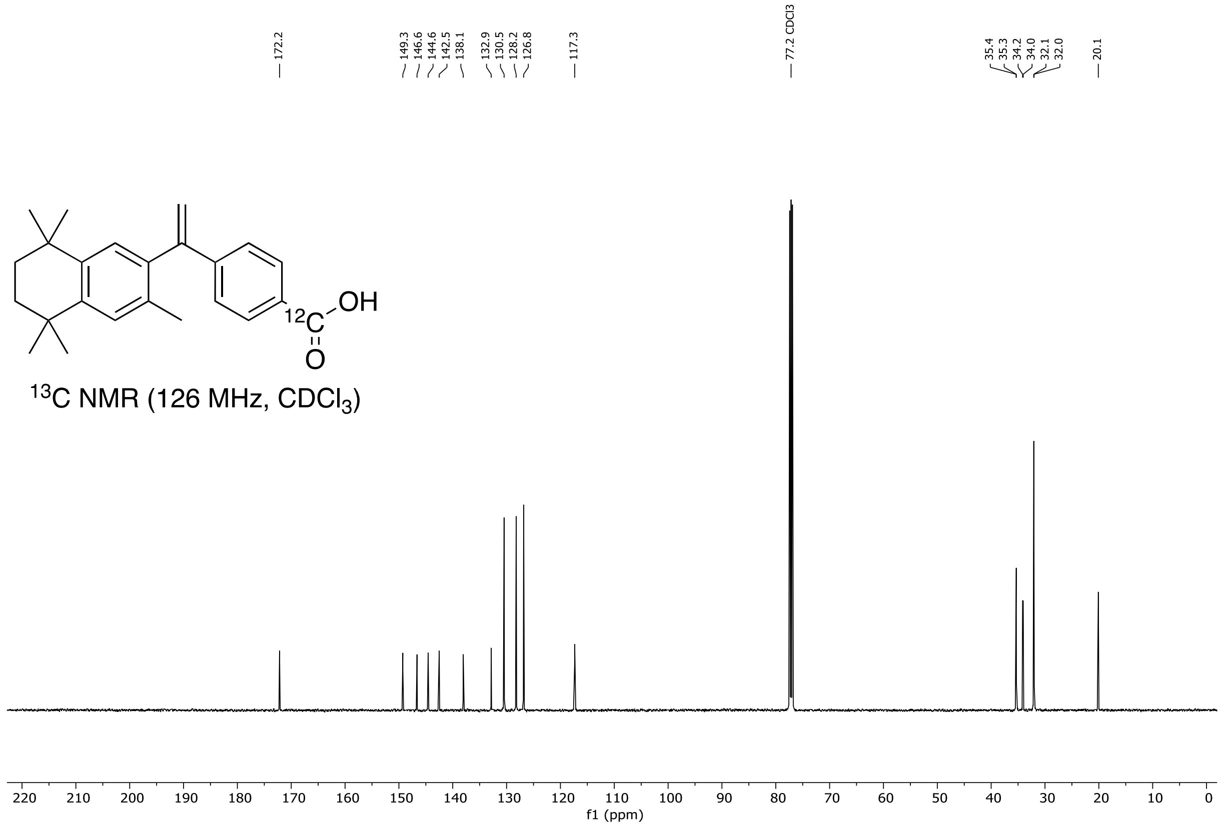
13C NMR (126 MHz, CDCl3)
172.2
149.3
146.6
144.6
142.5
138.1
132.9
130.5
128.2
126.8
117.3
77.2 CDCl3
35.4
35.3
34.2
34.0
32.1
32.0
20.1
f1 (ppm)

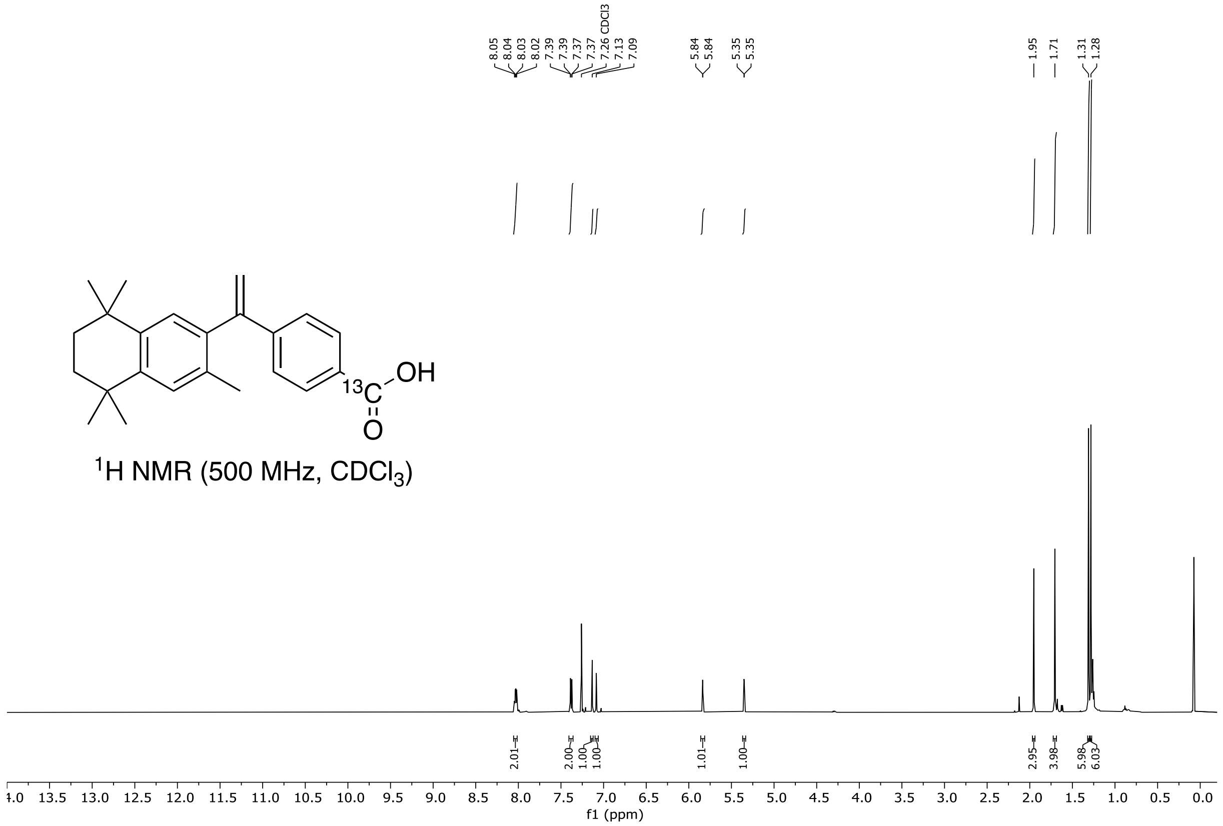
1H NMR (500 MHz, CDCl3)
8.05
8.04
8.03
8.02
7.39
7.39
7.37
7.37
7.26 CDCl3
7.13
7.09
5.84
5.84
5.35
5.35
1.95
1.71
1.31
1.28
2.01
2.00
1.00
1.00
1.01
1.00
2.95
3.98
5.98
6.03
f1 (ppm)

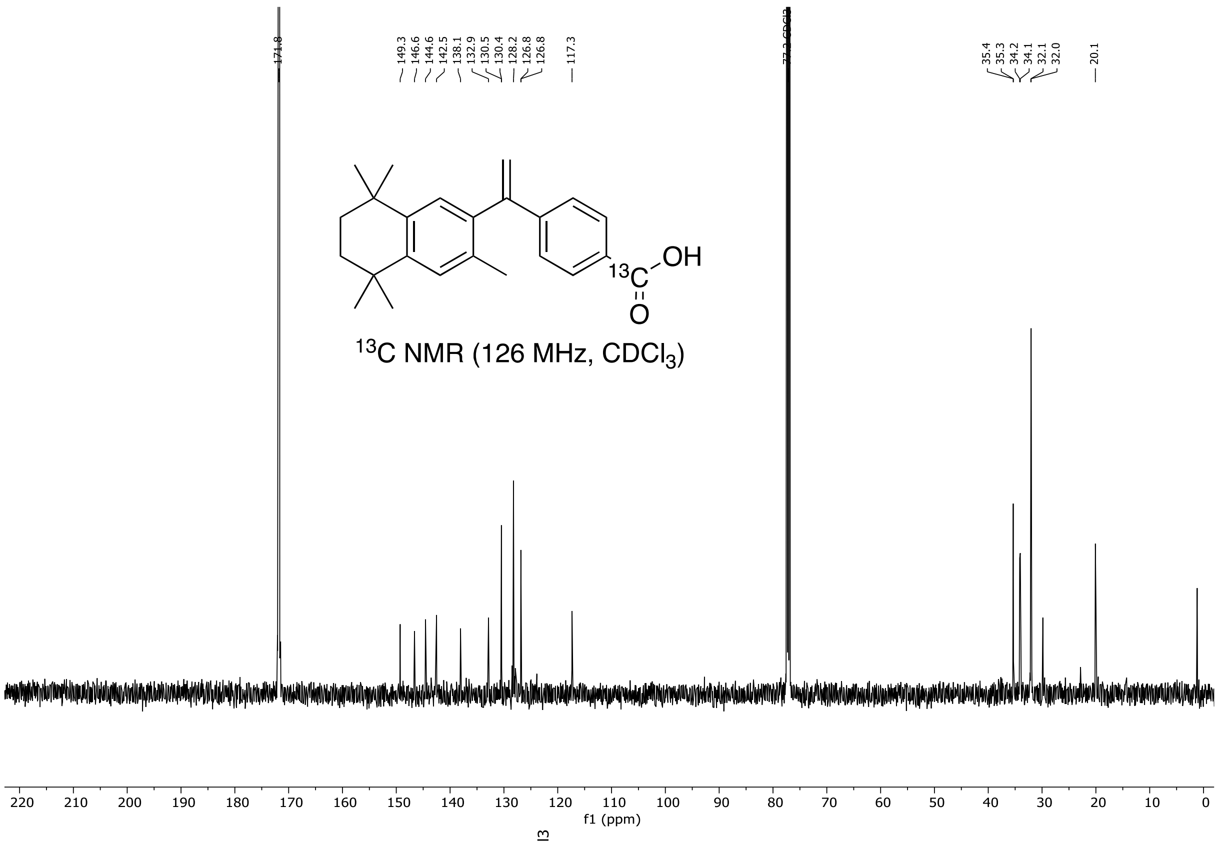
171.8
149.3
146.6
144.6
142.5
138.1
132.9
130.5
130.4
128.2
126.8
126.8
117.3
35.4
35.3
34.2
34.1
32.1
32.0
20.1
13C
OH
O
13C NMR (126 MHz, CDCl3)
220 210 200 190 180 170 160 150 140 130 120 110 100 90 80 70 60 50 40 30 20 10 0
f1 (ppm)

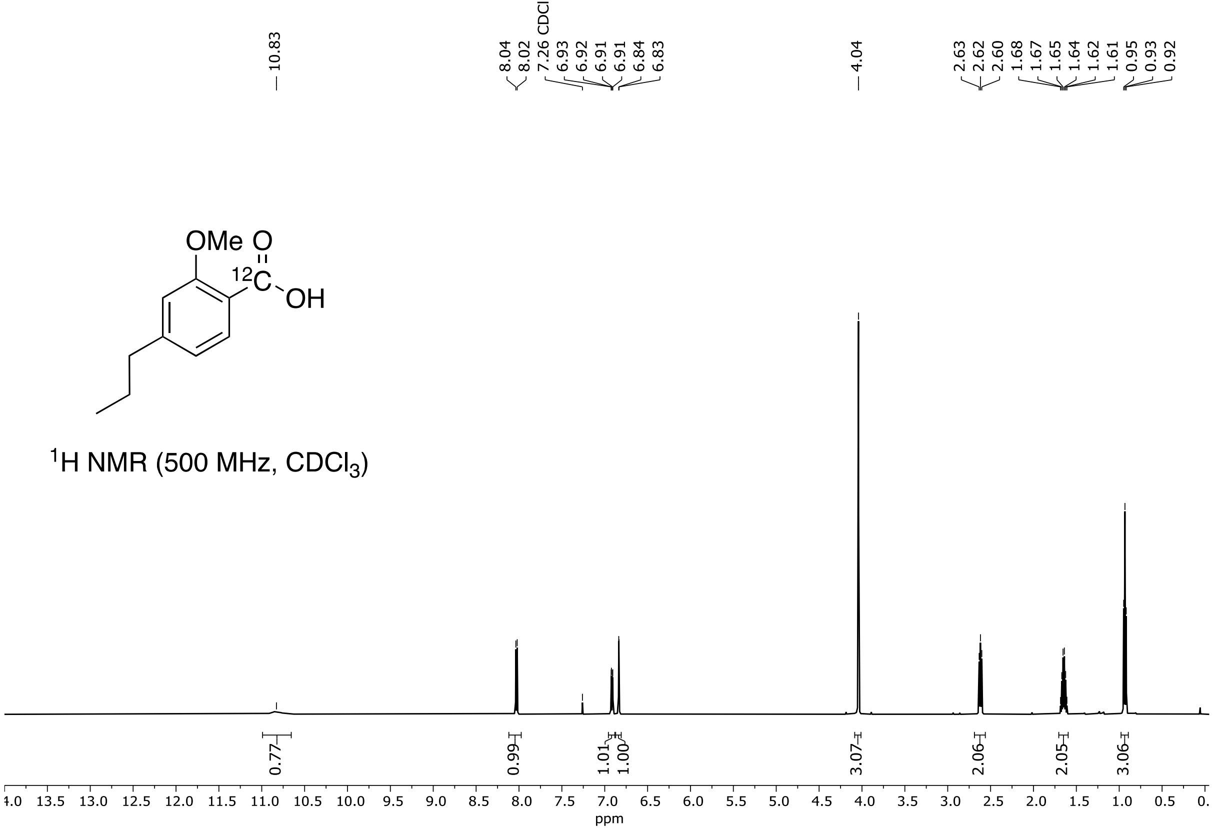
10.83
8.04
8.02
7.26 CDCl3
6.93
6.92
6.91
6.91
6.84
6.83
4.04
2.63
2.62
2.60
1.68
1.67
1.65
1.64
1.62
1.61
0.95
0.93
0.92
OMe
O
12C
OH
1H NMR (500 MHz, CDCl3)
0.77
0.99
1.01
1.00
3.07
2.06
2.05
3.06
13.5 13.0 12.5 12.0 11.5 11.0 10.5 10.0 9.5 9.0 8.5 8.0 7.5 7.0 6.5 6.0 5.5 5.0 4.5 4.0 3.5 3.0 2.5 2.0 1.5 1.0 0.5
ppm

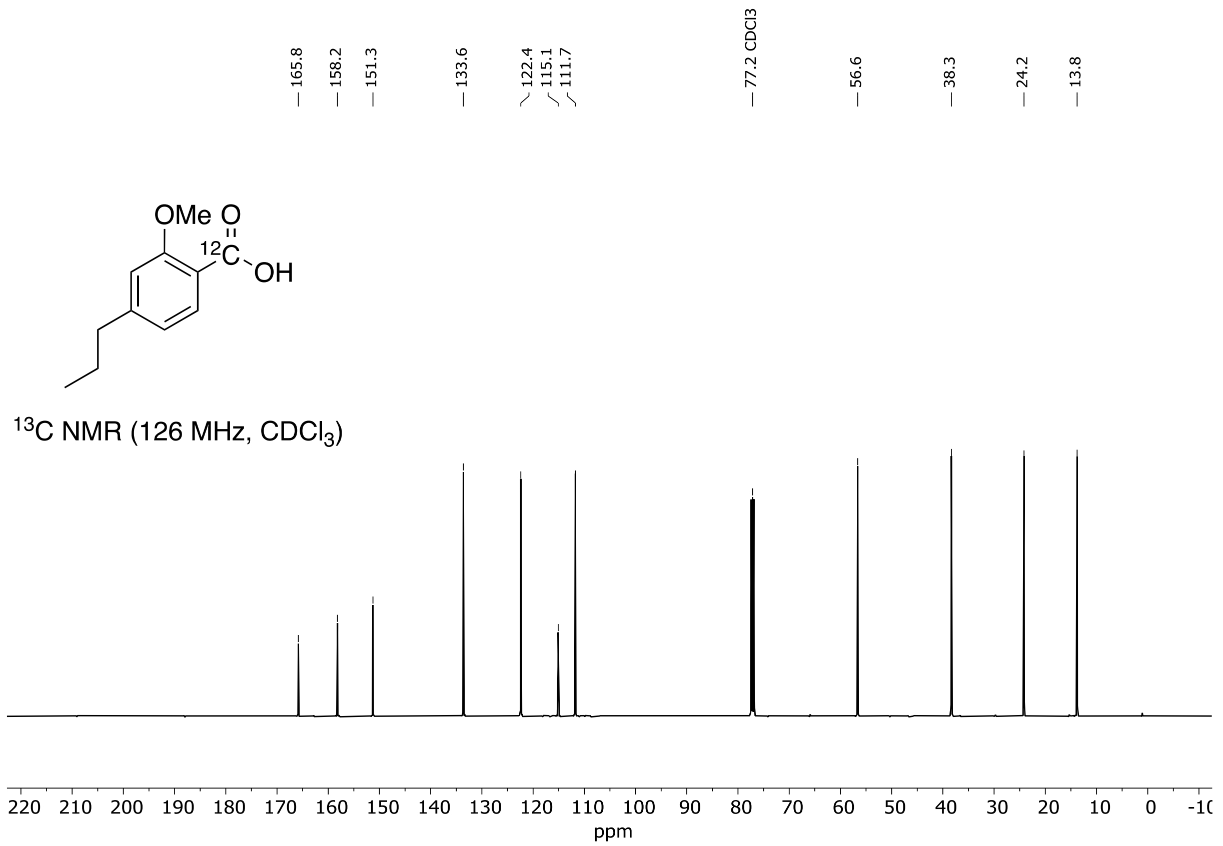
165.8
158.2
151.3
133.6
122.4
115.1
111.7
77.2 CDCl3
56.6
38.3
24.2
13.8
OMe O
12C
OH
13C NMR (126 MHz, CDCl3)
ppm

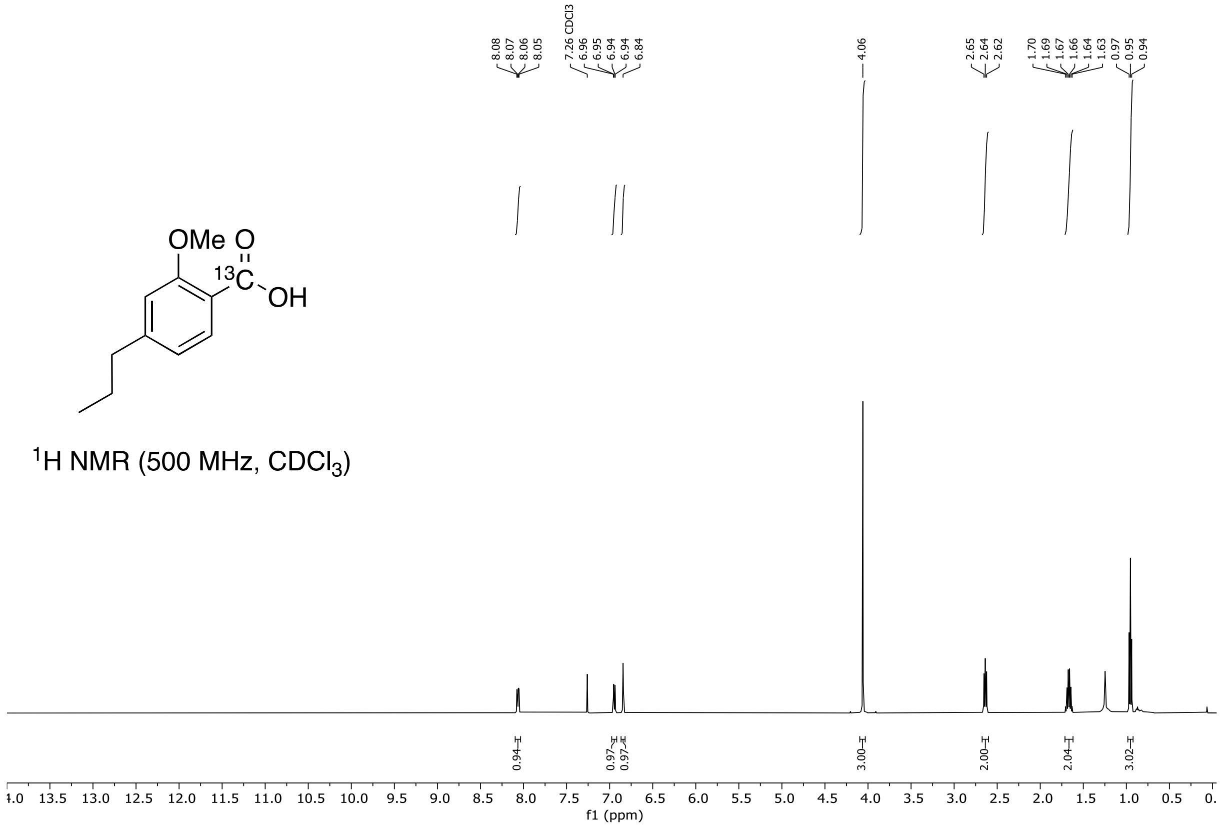
8.08
8.07
8.06
8.05
7.26 CDCl3
6.96
6.95
6.94
6.94
6.84
4.06
2.65
2.64
2.62
1.70
1.69
1.67
1.66
1.64
1.63
0.97
0.95
0.94
OMe O
13C
OH
1H NMR (500 MHz, CDCl3)
0.94
0.97
0.97
3.00
2.00
2.04
3.02
f1 (ppm)

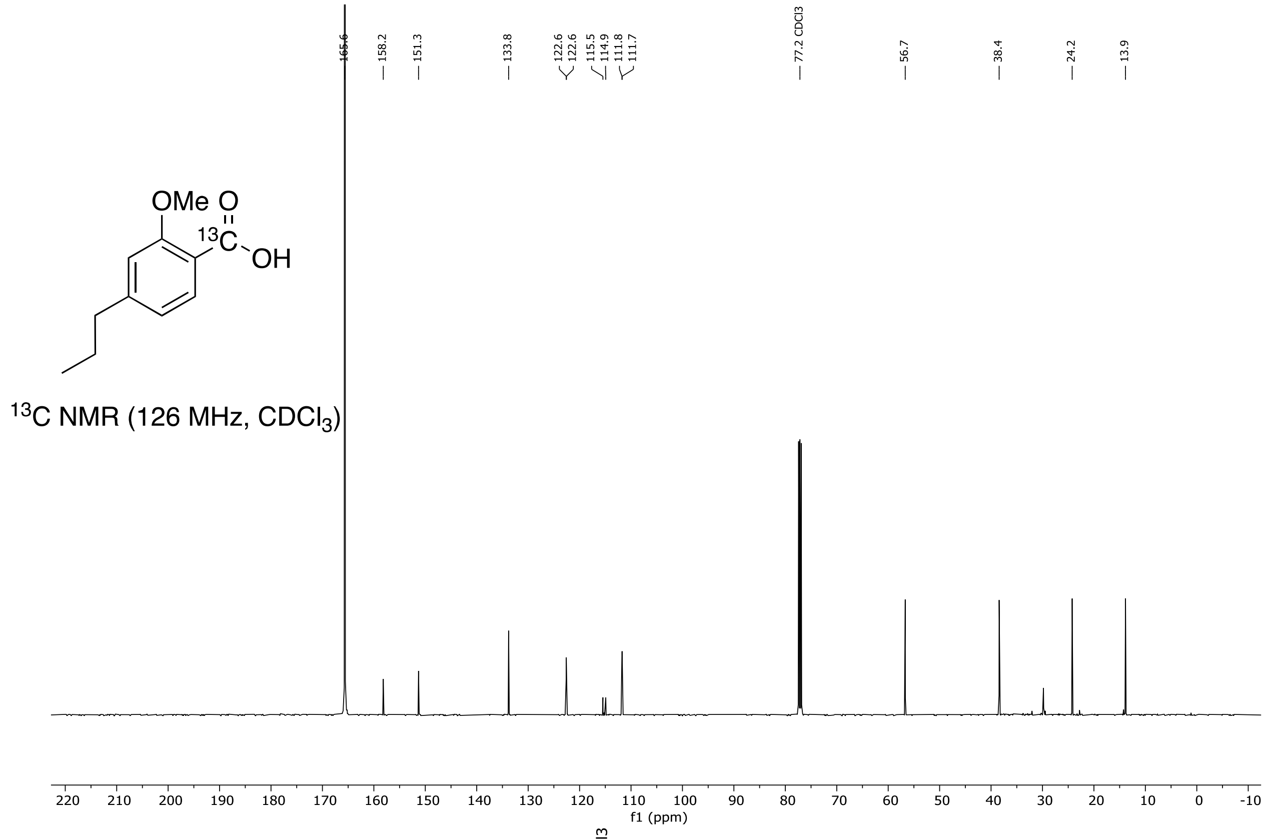
OMe
O
13C
OH
13C NMR (126 MHz, CDCl3)
165.6
158.2
151.3
133.8
122.6
122.6
115.5
114.9
111.8
111.7
77.2 CDCl3
56.7
38.4
24.2
13.9
f1 (ppm)

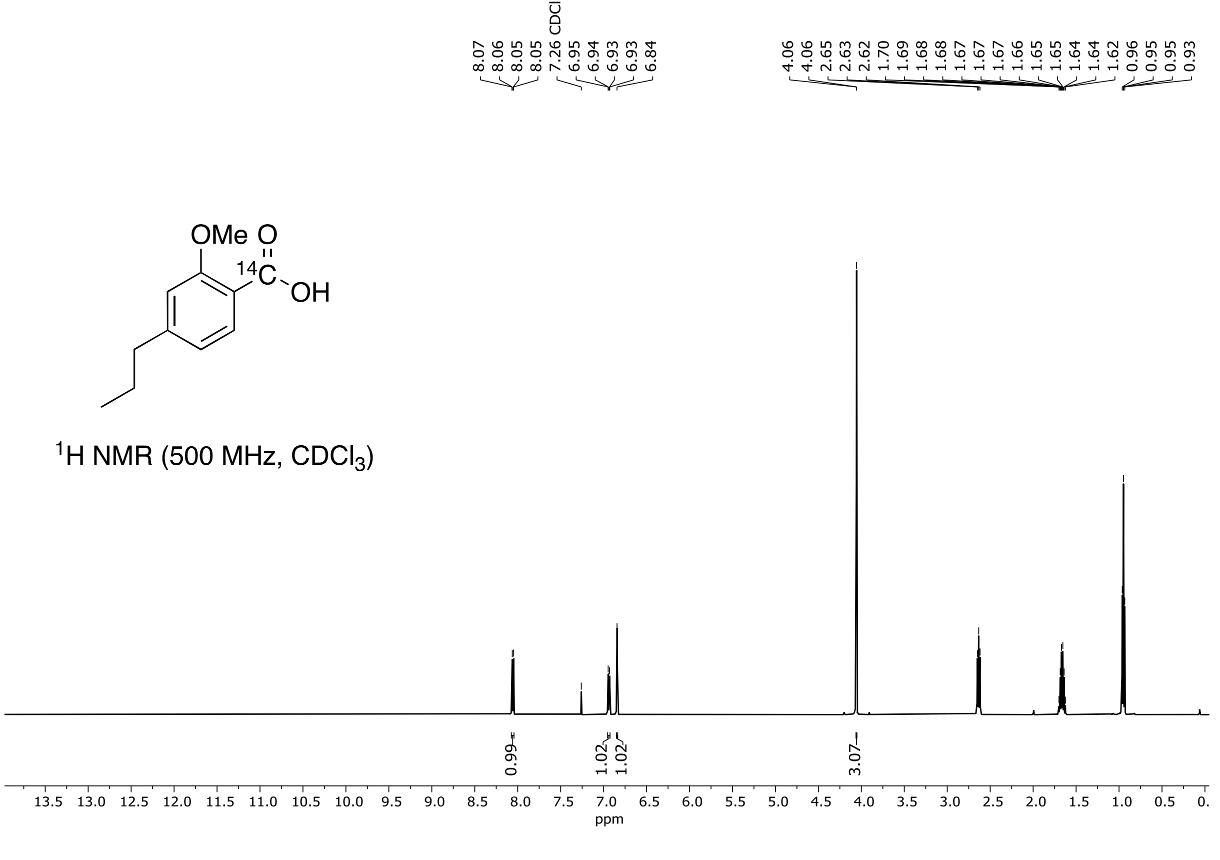
OMe
O
14C
OH
1H NMR (500 MHz, CDCl3)
8.07
8.06
8.05
8.05
7.26 CDCl3
6.95
6.94
6.93
6.93
6.84
4.06
4.06
2.65
2.63
2.62
1.70
1.69
1.68
1.68
1.67
1.67
1.66
1.65
1.65
1.64
1.64
1.62
0.96
0.95
0.95
0.93
0.99
1.02
1.02
3.07
ppm

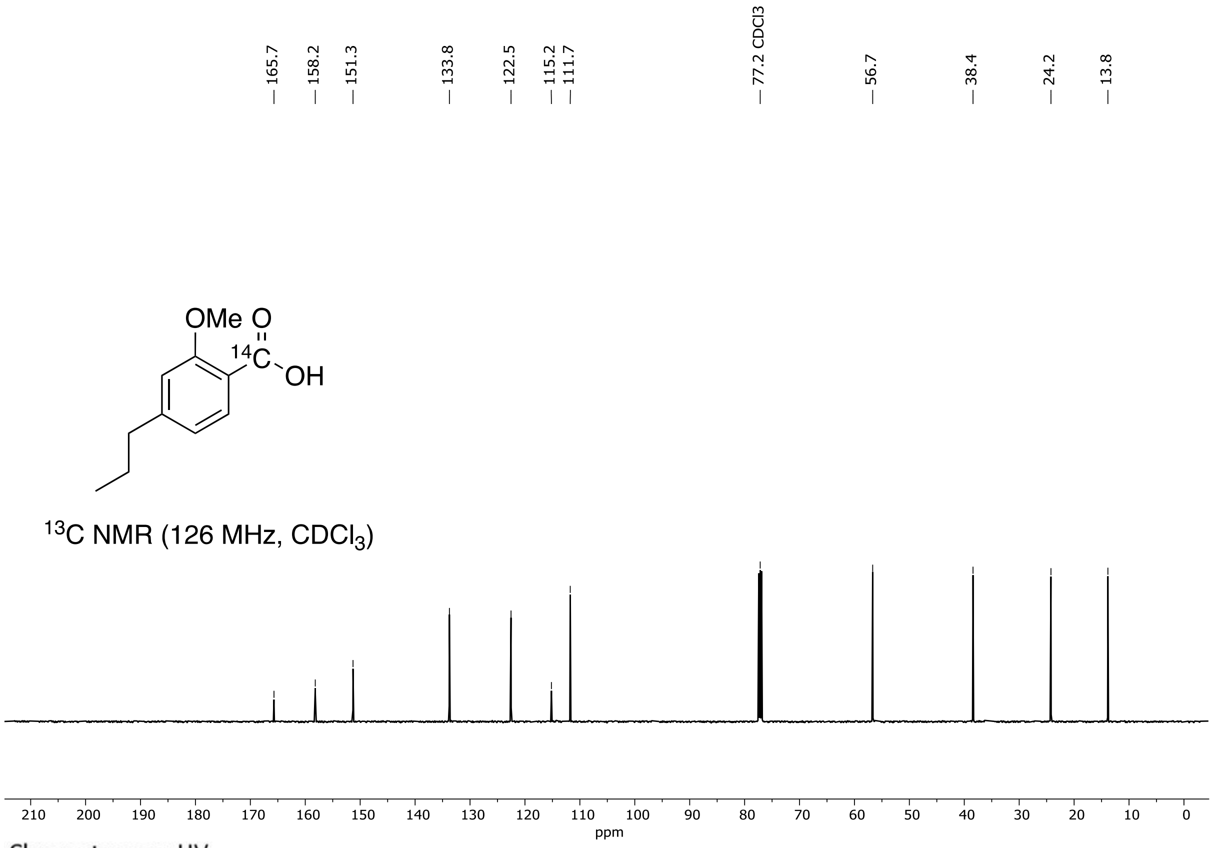

Chromatogram: UV

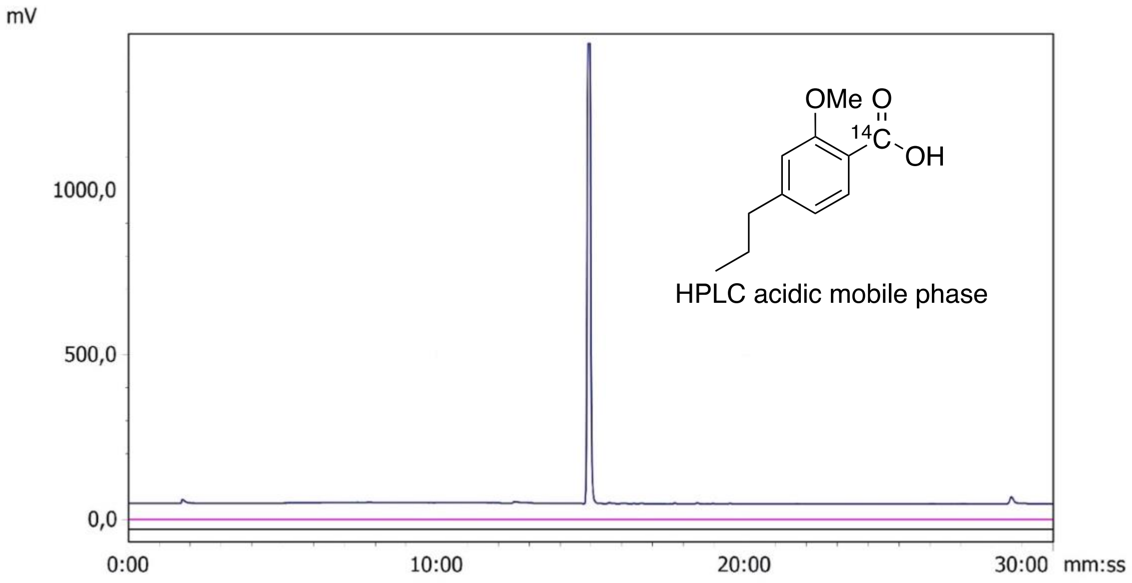

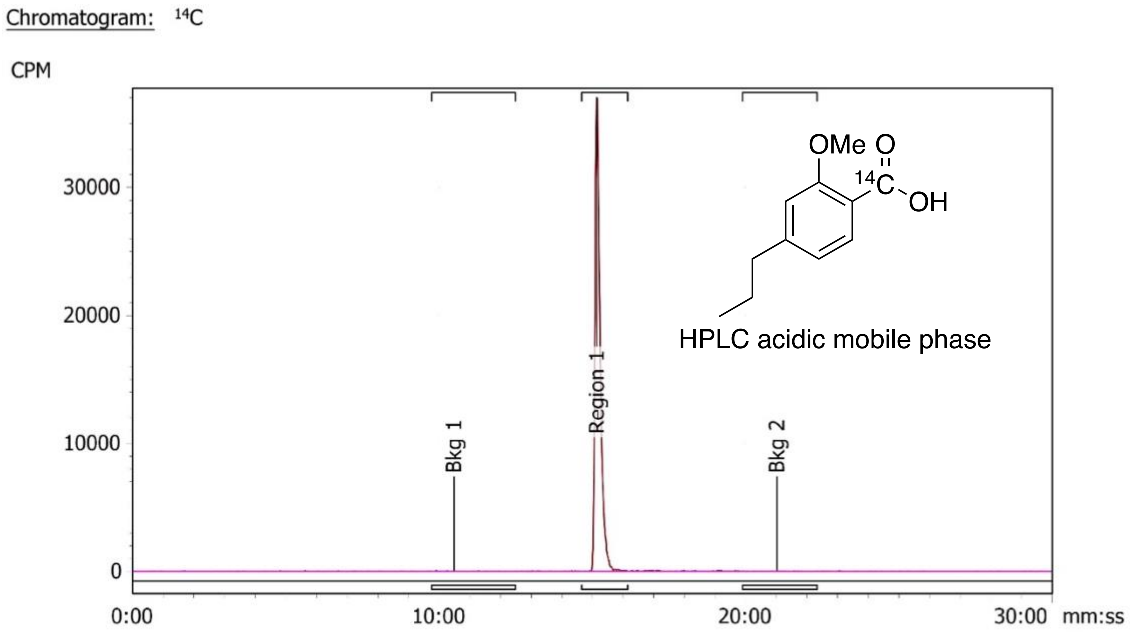
Chromatogram: ¹⁴C
CPM
30000
20000
10000
0
Bkg 1
Region 1
Bkg 2
OMe O
14C
OH
HPLC acidic mobile phase
0:00
10:00
20:00
30:00
mm:ss

RCP > 99%

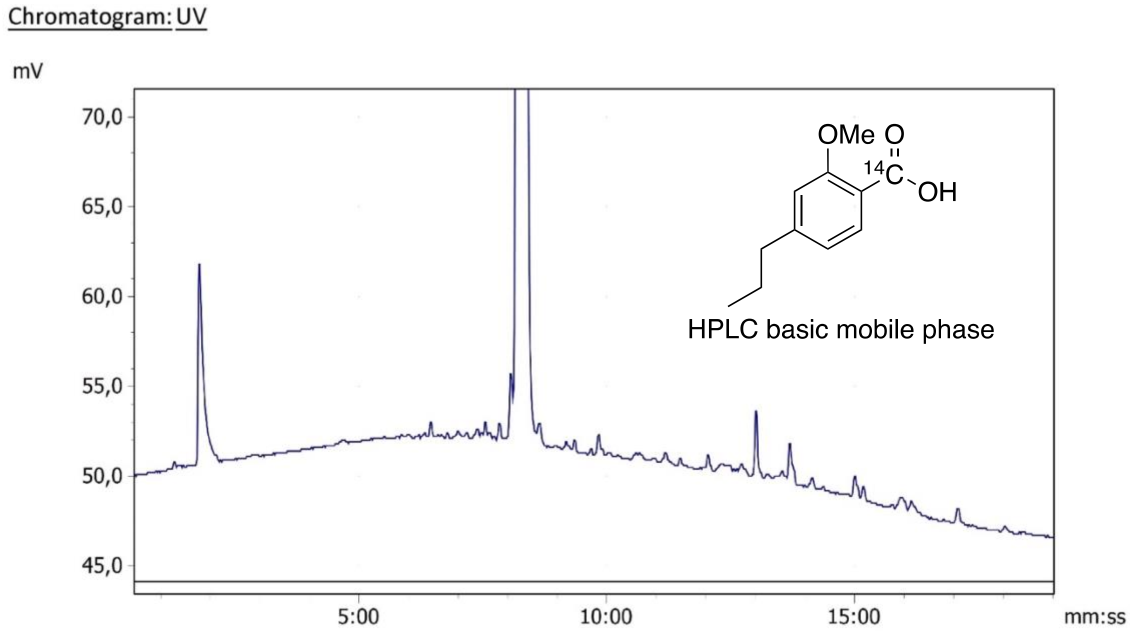
Chromatogram: UV
mV
70,0
65,0
60,0
55,0
50,0
45,0
OMe O
14C
OH
HPLC basic mobile phase
5:00
10:00
15:00
mm:ss

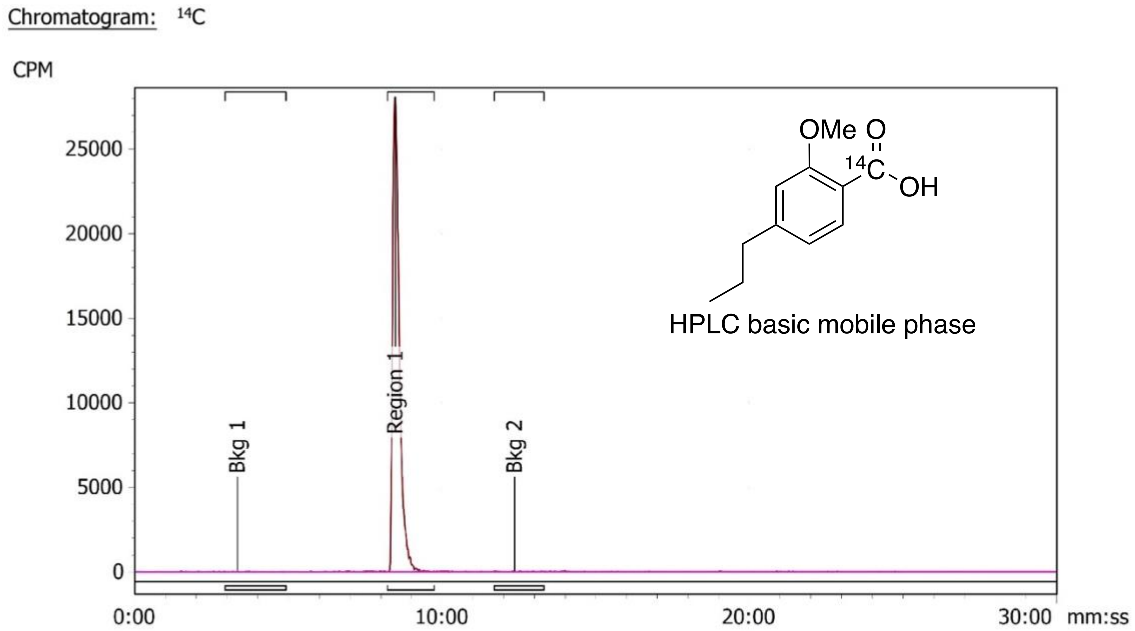
Chromatogram: 14C
CPM
25000
20000
15000
10000
5000
0
Bkg 1
Region 1
Bkg 2
OMe
O
14C
OH
HPLC basic mobile phase
0:00
10:00
20:00
30:00
mm:ss

RCP > 99%

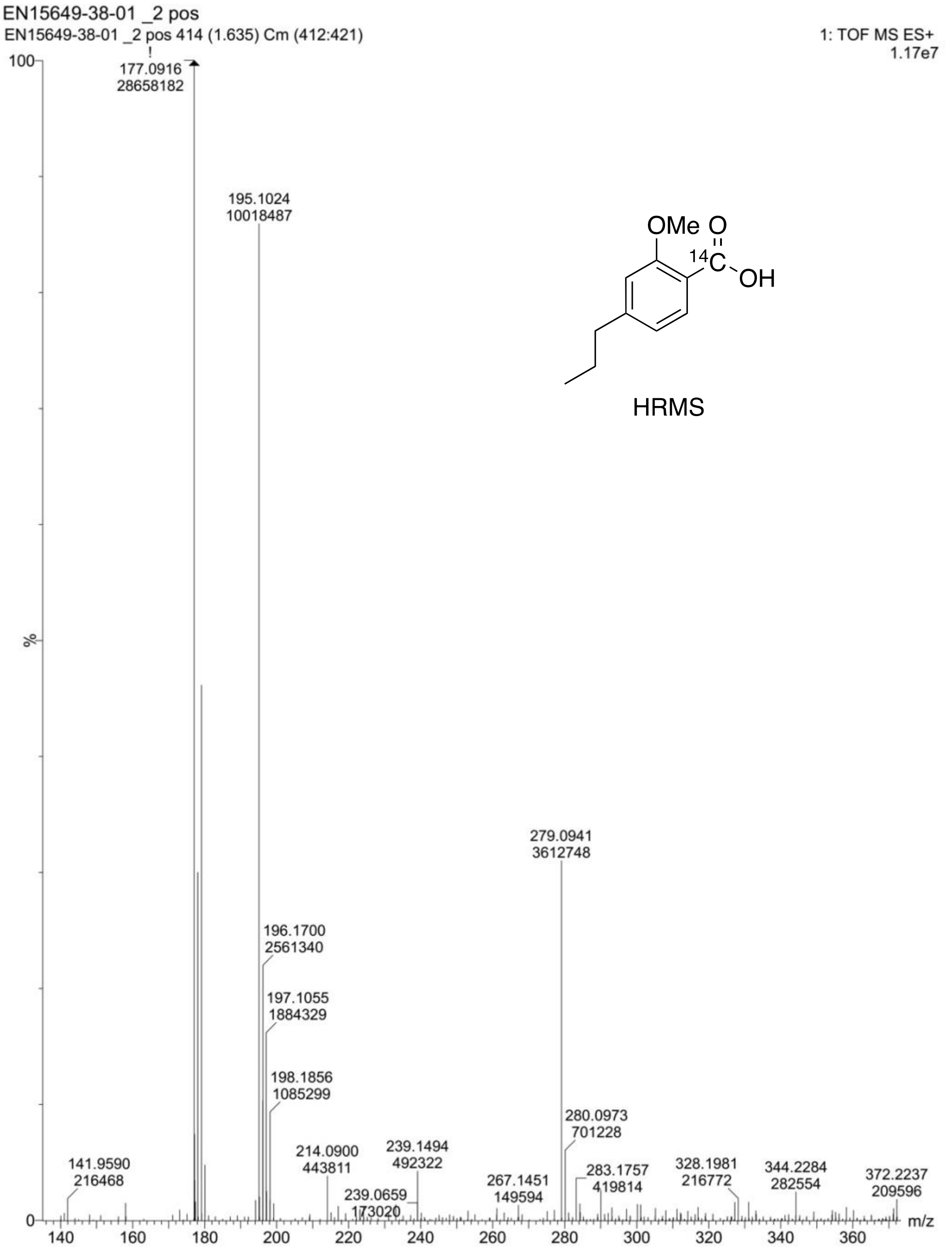
EN15649-38-01 _2 pos
EN15649-38-01 _2 pos 414 (1.635) Cm (412:421)
1: TOF MS ES+
1.17e7
100
%
0
177.0916
28658182
195.1024
10018487
196.1700
2561340
197.1055
1884329
198.1856
1085299
279.0941
3612748
280.0973
701228
141.9590
216468
214.0900
443811
239.1494
492322
239.0659
173020
267.1451
149594
283.1757
419814
328.1981
216772
344.2284
282554
372.2237
209596
140
160
180
200
220
240
260
280
300
320
340
360
m/z
OMe
O
14C
OH
HRMS

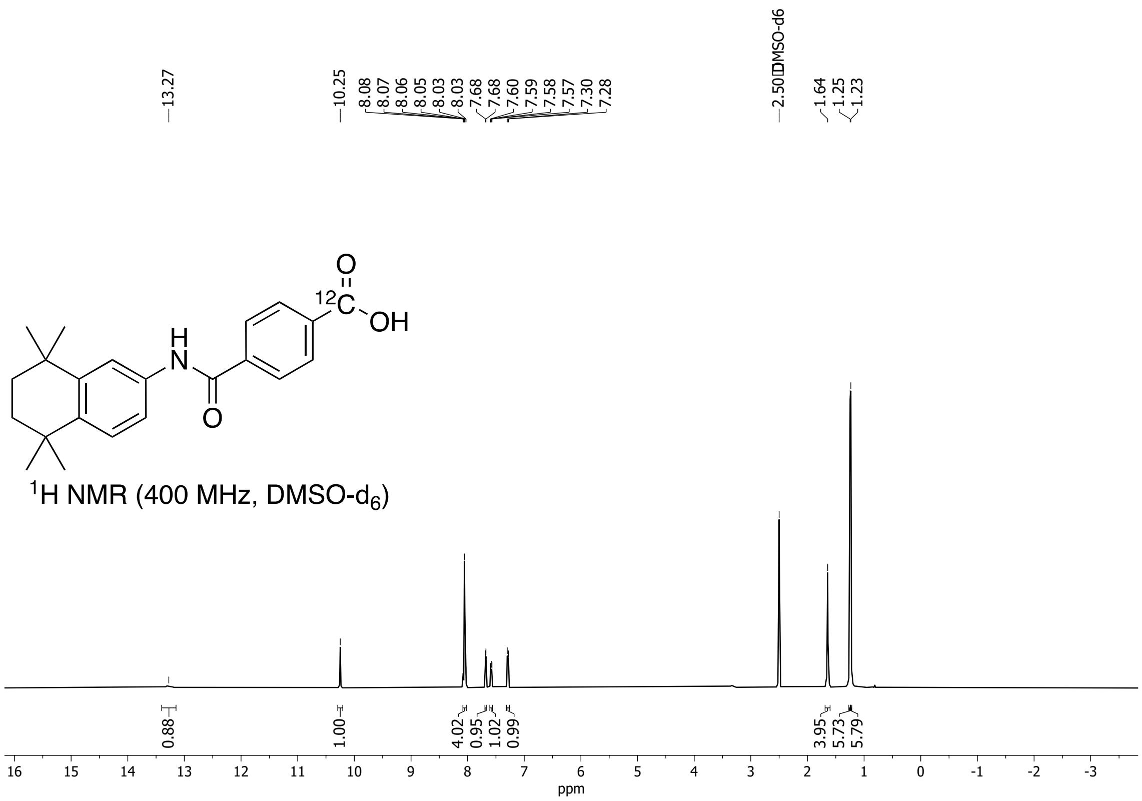
13.27
10.25
8.08
8.07
8.06
8.05
8.03
8.03
7.68
7.68
7.60
7.59
7.58
7.57
7.30
7.28
2.50 DMSO-d6
1.64
1.25
1.23
O
12C
OH
H
N
O
^{1}H NMR (400 MHz, DMSO-d$_6$)
0.88
1.00
4.02
0.95
1.02
0.99
3.95
5.73
5.79
ppm

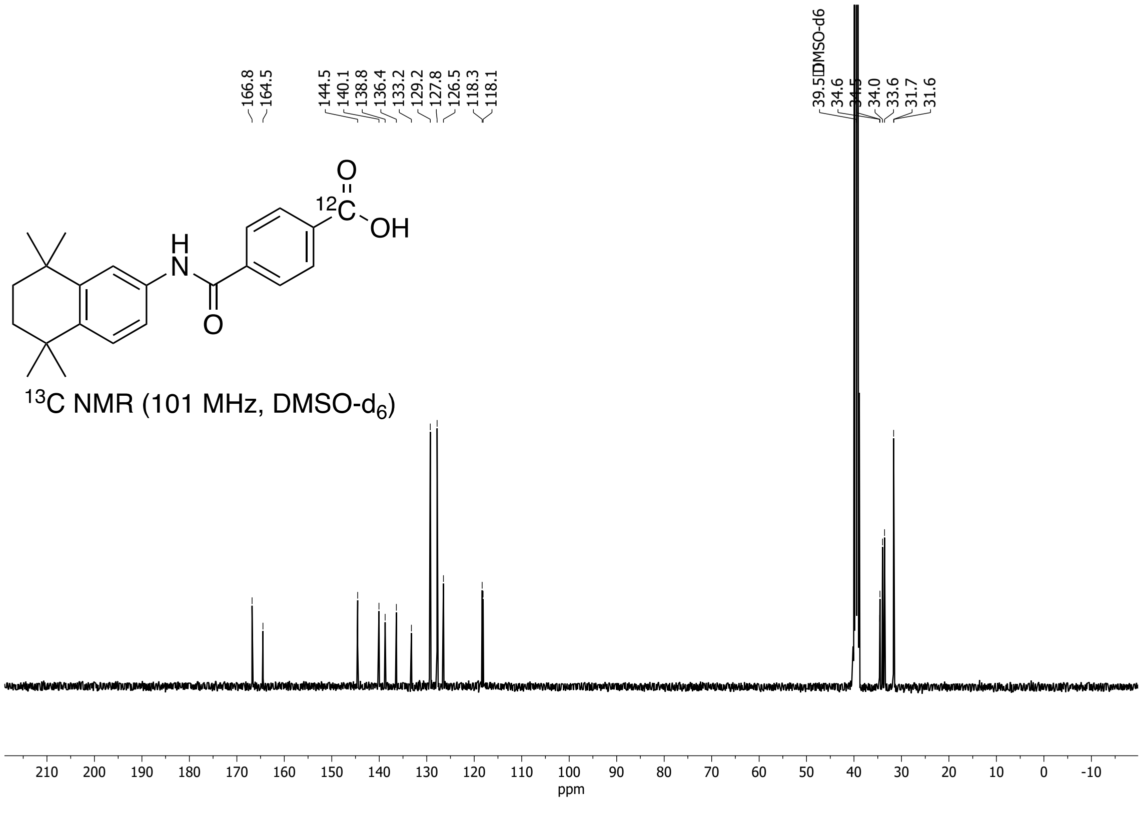
166.8
164.5
144.5
140.1
138.8
136.4
133.2
129.2
127.8
126.5
118.3
118.1
39.5 DMSO-d6
34.6
34.5
34.0
33.6
31.7
31.6
O
12C
OH
H
N
O
^{13}C NMR (101 MHz, DMSO-d$_6$)
ppm

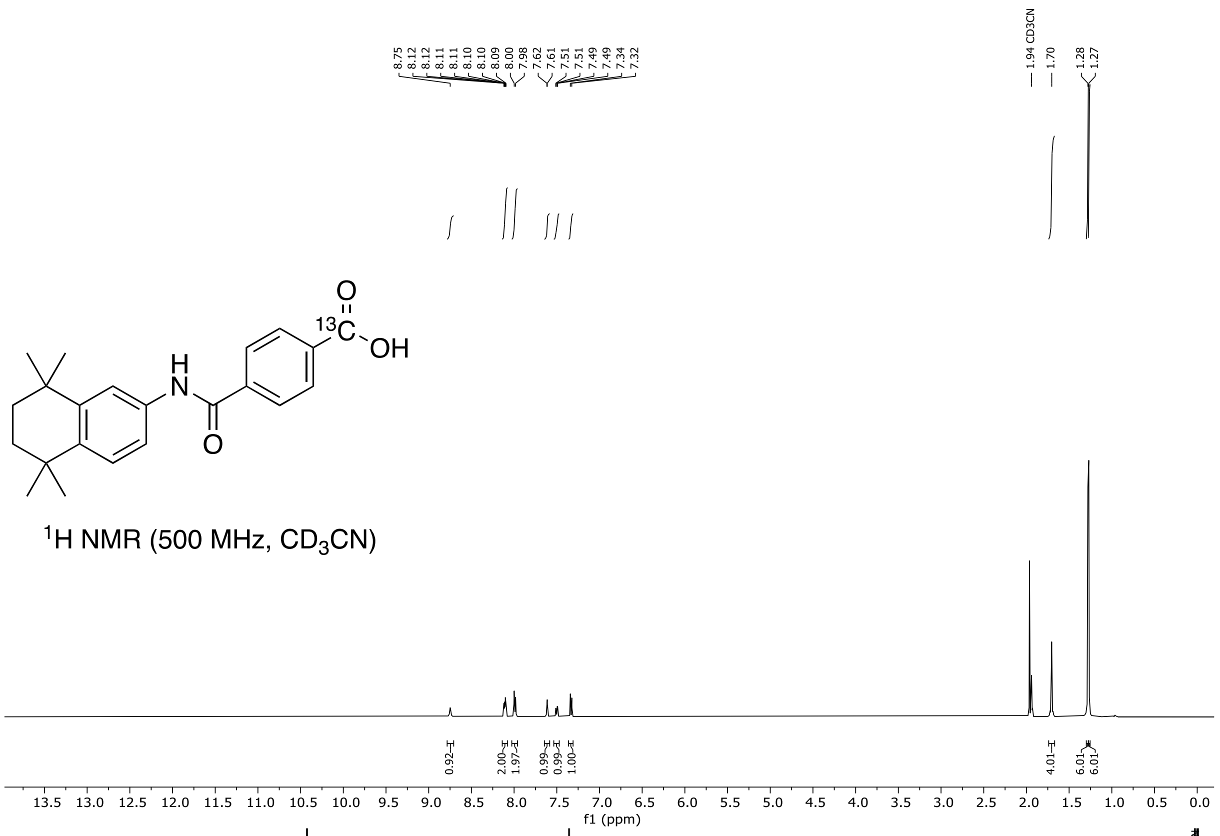

1H NMR (500 MHz, CD3CN)
f1 (ppm)

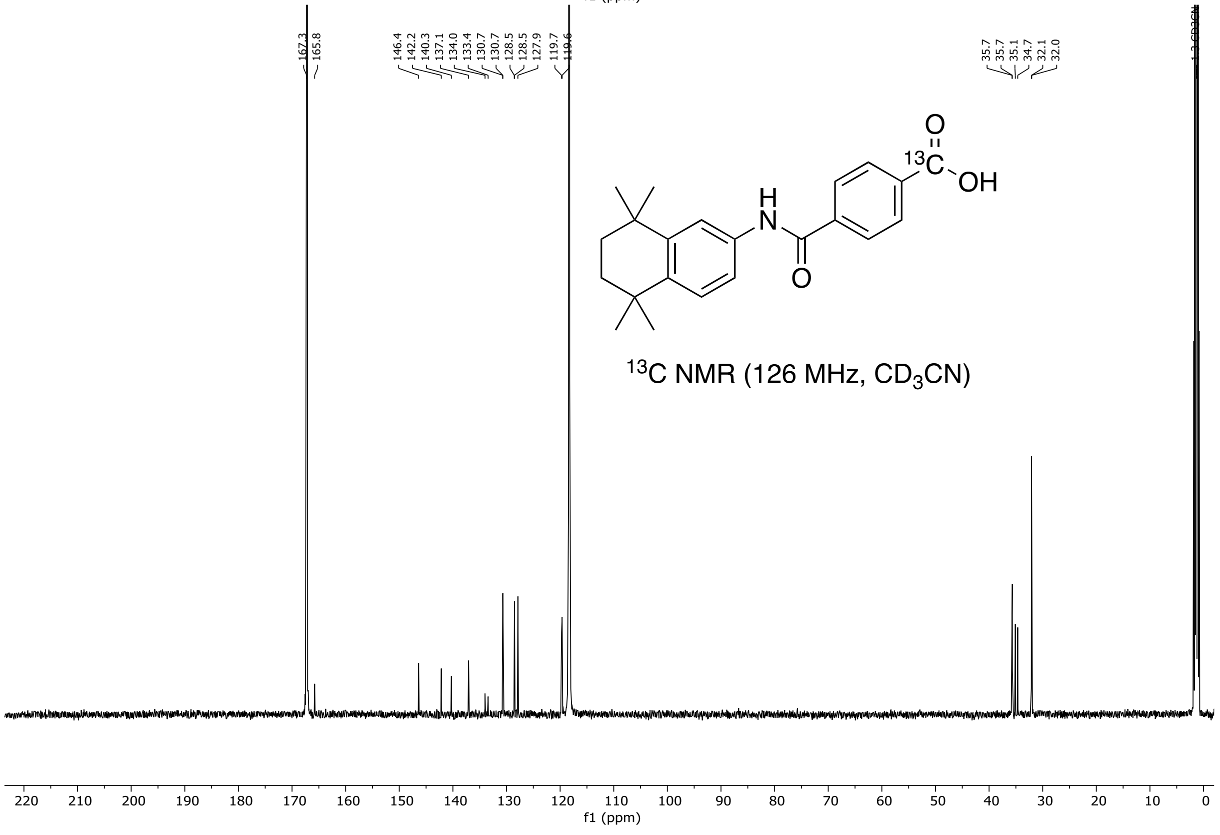

13C NMR (126 MHz, CD3CN)
f1 (ppm)

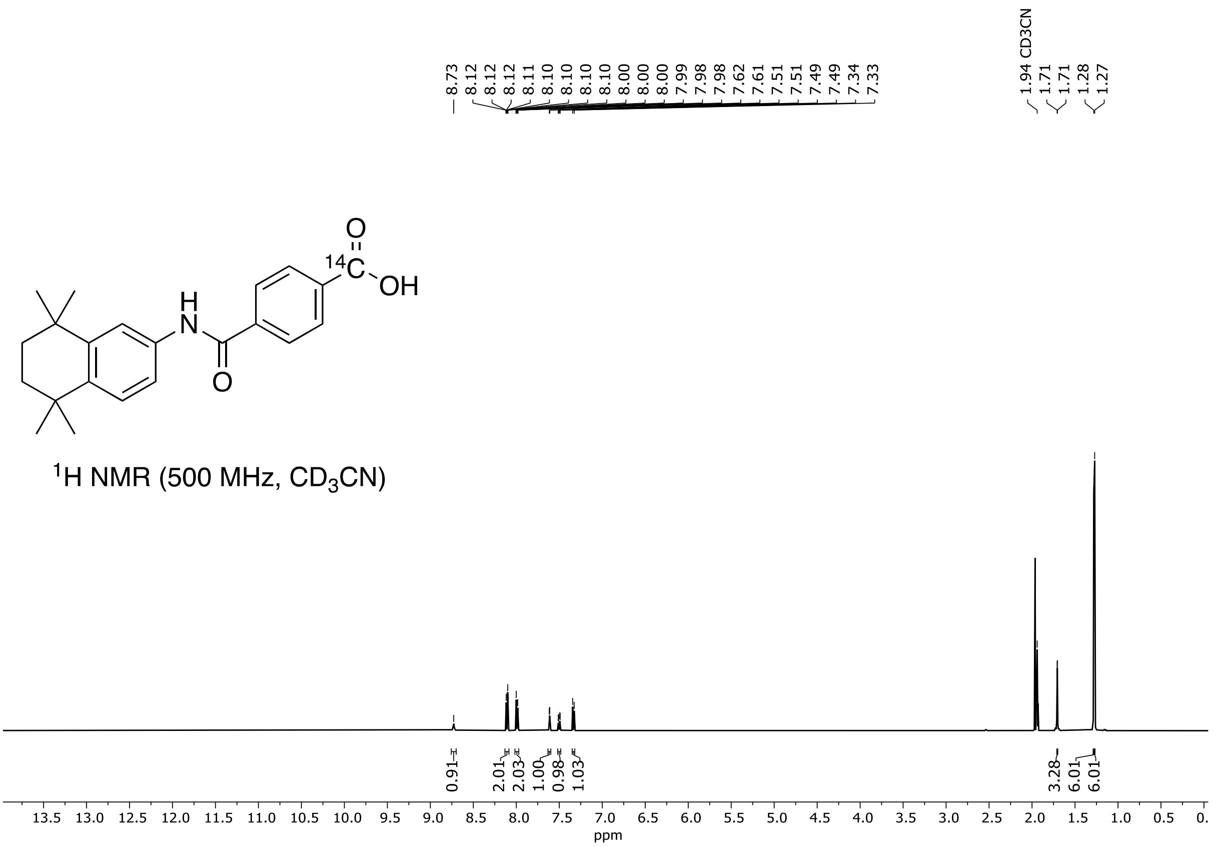
8.73
8.12
8.12
8.12
8.11
8.10
8.10
8.10
8.10
8.00
8.00
8.00
7.99
7.98
7.98
7.62
7.61
7.51
7.51
7.49
7.49
7.34
7.33
1.94 CD3CN
1.71
1.71
1.28
1.27
^{1}H NMR (500 MHz, CD_3CN)
0.91
2.01
2.03
1.00
0.98
1.03
3.28
6.01
6.01
ppm

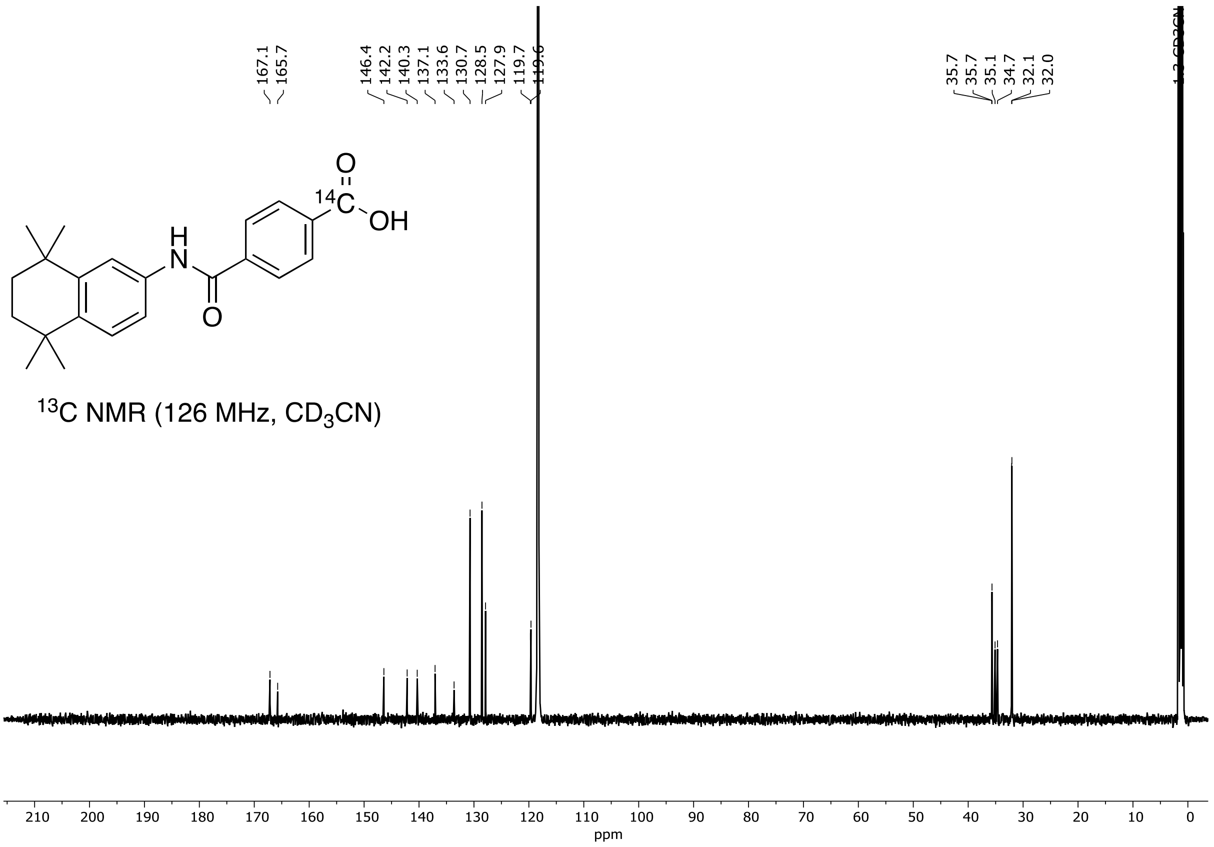
167.1
165.7
146.4
142.2
140.3
137.1
133.6
130.7
128.5
127.9
119.7
35.7
35.7
35.1
34.7
32.1
32.0
^{13}C NMR (126 MHz, CD_3CN)
ppm

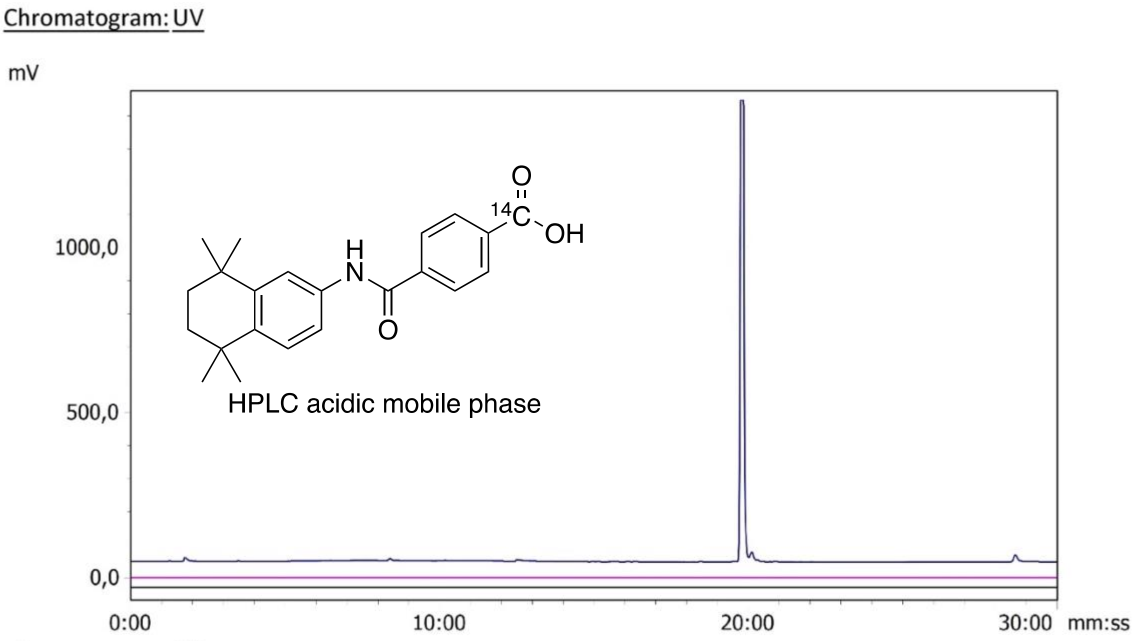
Chromatogram: UV
mV
1000,0
500,0
0,0
0:00
10:00
20:00
30:00
mm:ss
HPLC acidic mobile phase
OH

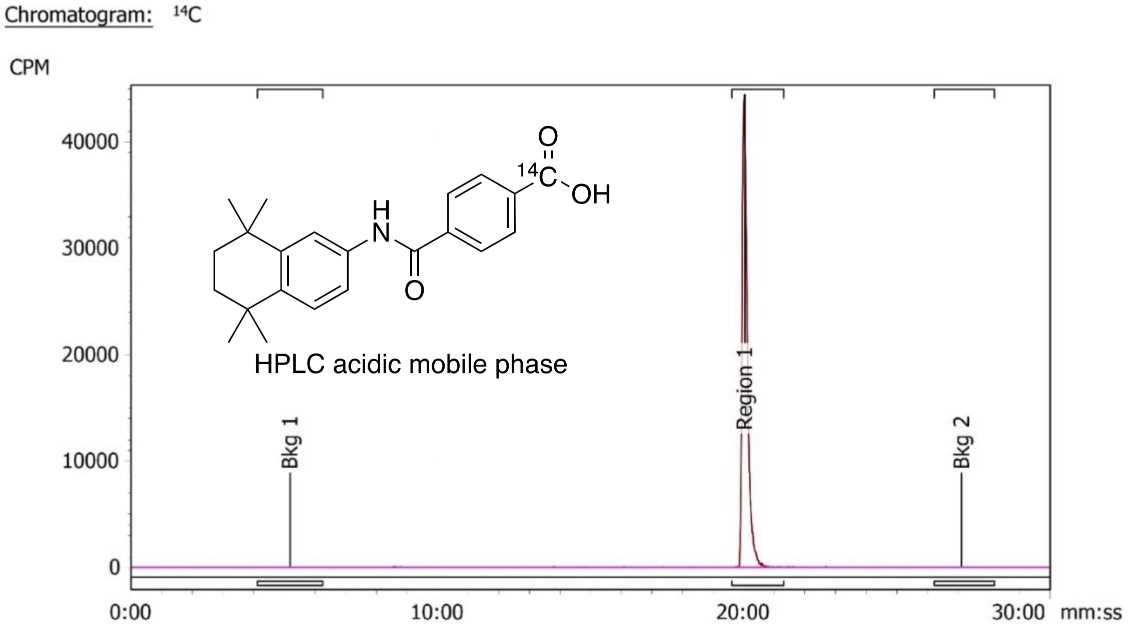
Chromatogram: 14C
CPM
40000
30000
20000
10000
0
0:00
10:00
20:00
30:00
mm:ss
HPLC acidic mobile phase
Bkg 1
Region 1
Bkg 2

RCP > 99%

Chromatogram: UV

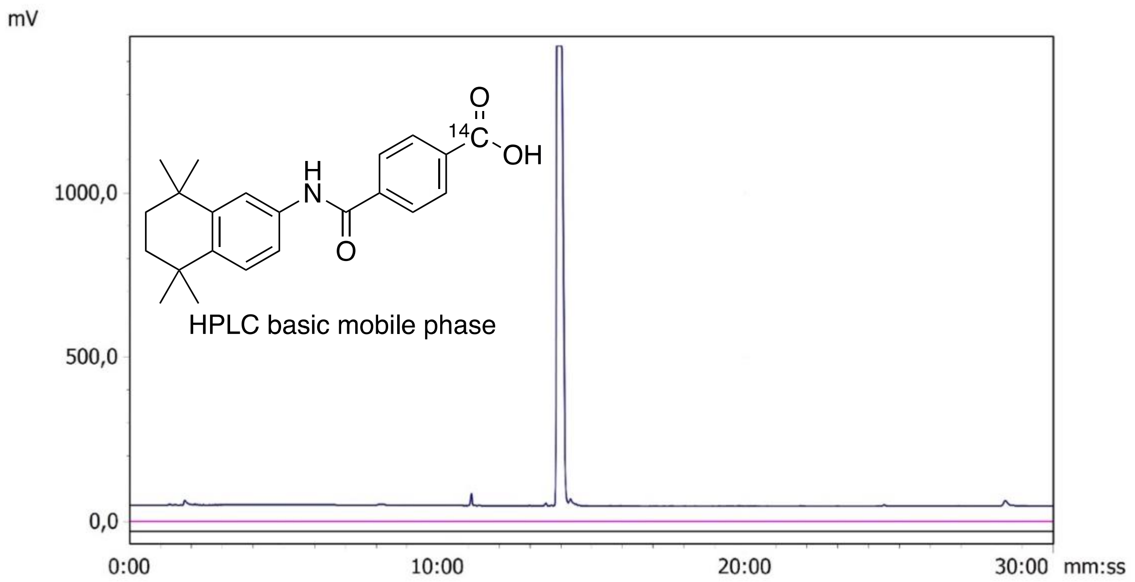

Chromatogram: ^{14}C

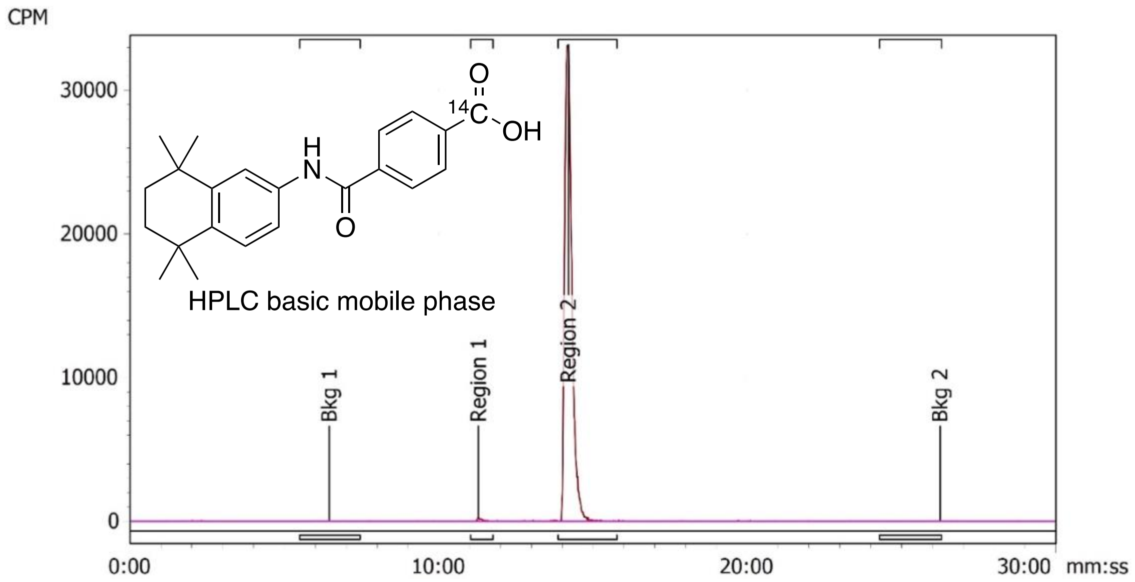

RCP > 99%

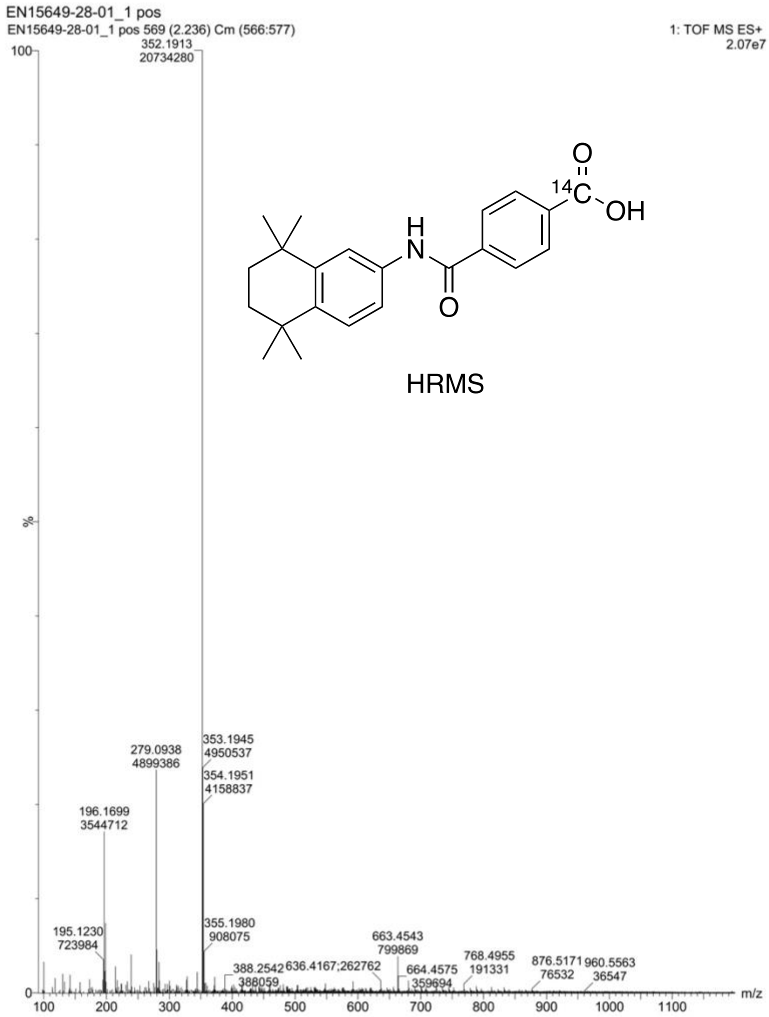
EN15649-28-01_1 pos
EN15649-28-01_1 pos 569 (2.236) Cm (566:577)
1: TOF MS ES+
2.07e7
352.1913
20734280
353.1945
4950537
354.1951
4158837
279.0938
4899386
196.1699
3544712
195.1230
723984
355.1980
908075
388.2542
388059
636.4167;262762
663.4543
799869
664.4575
359694
768.4955
191331
876.5171
76532
960.5563
36547
%
m/z
HRMS

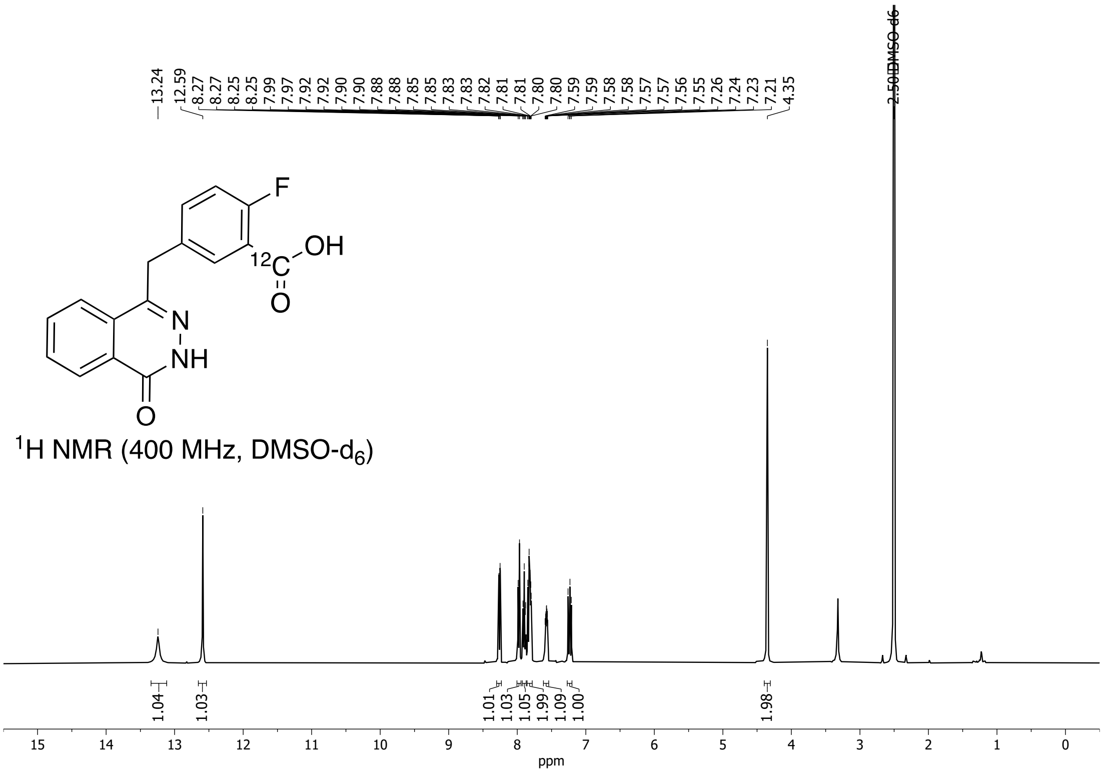

^1H NMR (400 MHz, DMSO-d_6)

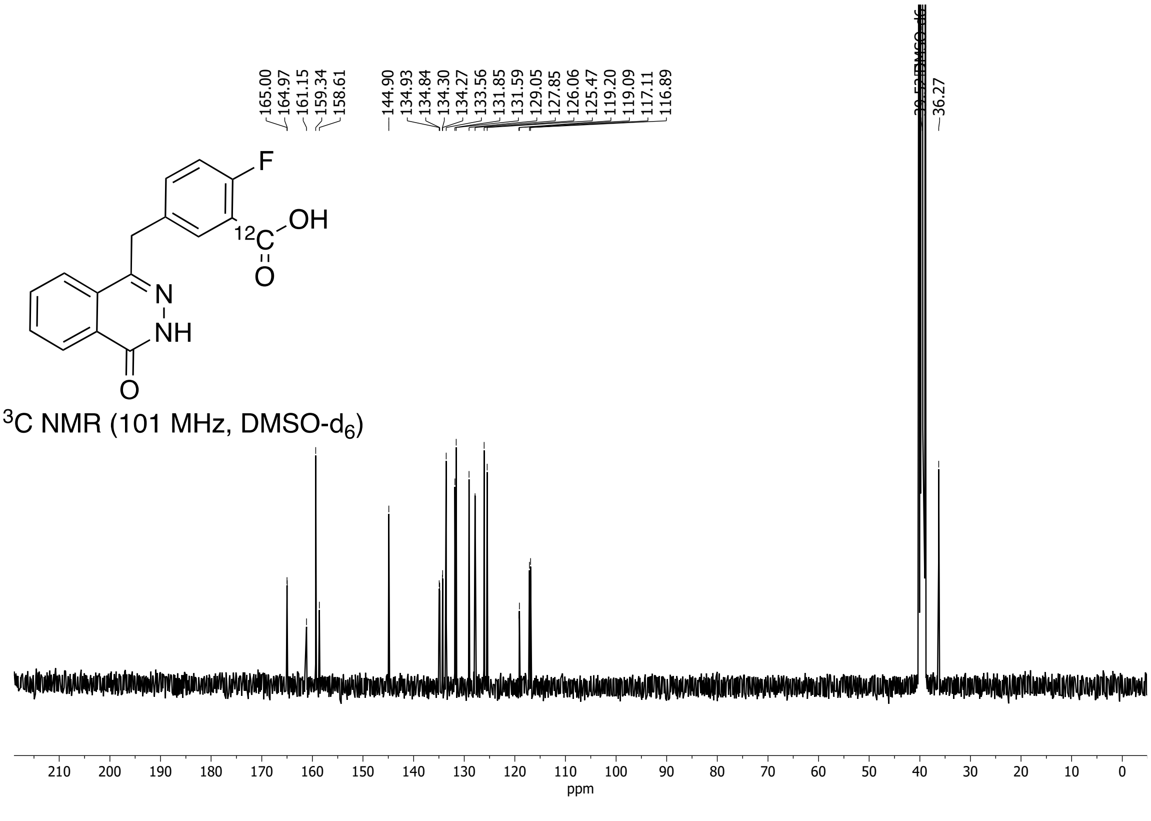

^{13}C NMR (101 MHz, DMSO-d_6)

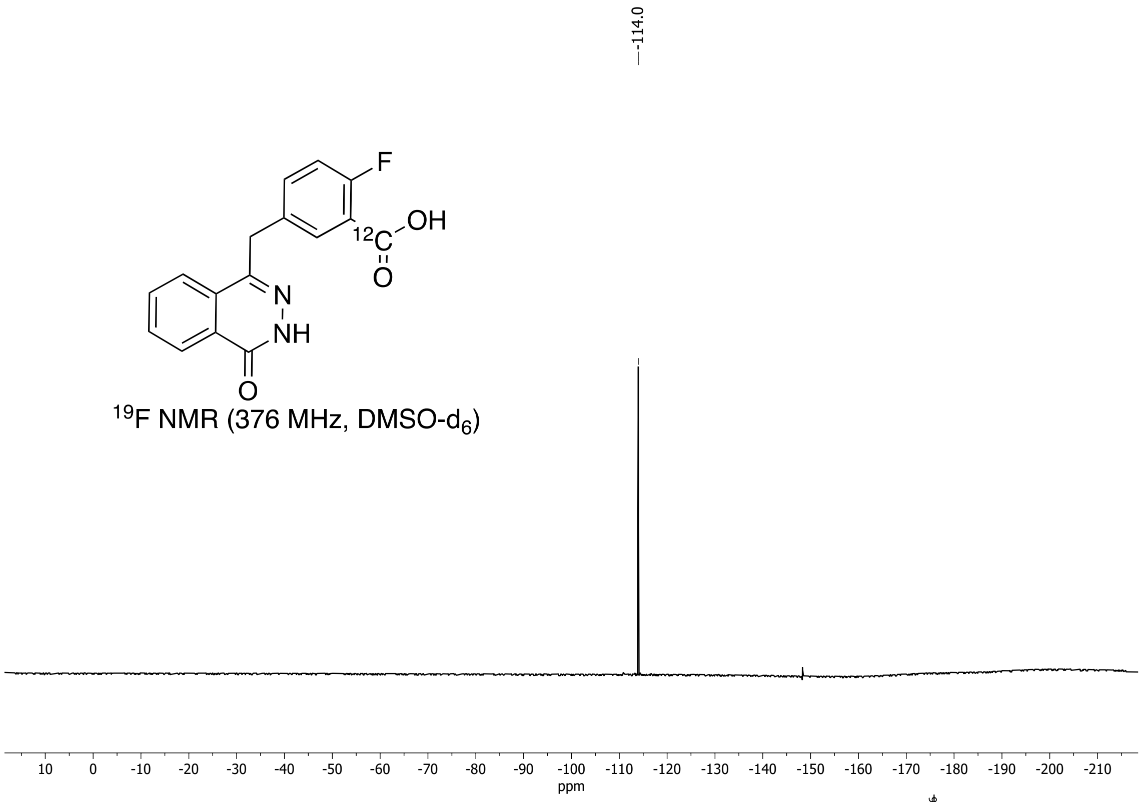

-114.0
F
^{12}C
OH
O
N
NH
O
^{19}F NMR (376 MHz, DMSO-d_6)
10 0 -10 -20 -30 -40 -50 -60 -70 -80 -90 -100 -110 -120 -130 -140 -150 -160 -170 -180 -190 -200 -210
ppm

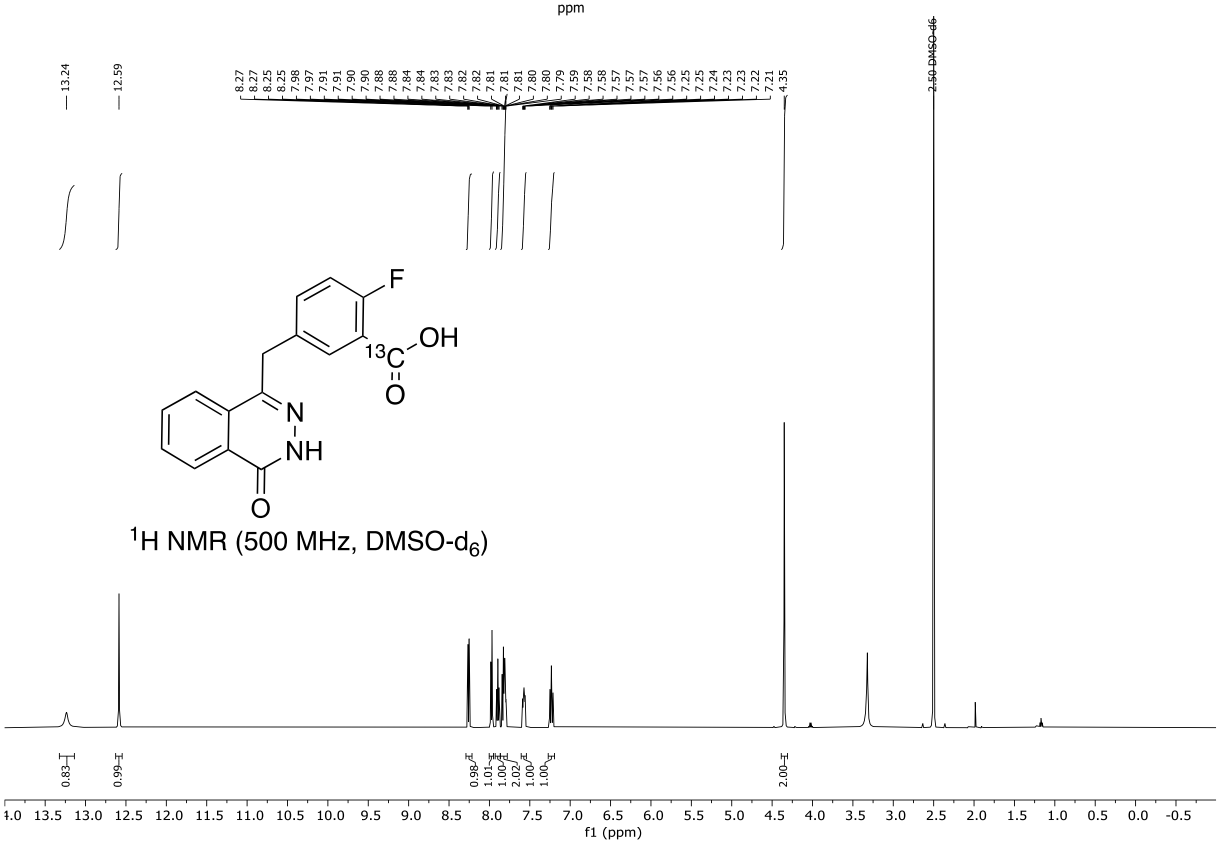

13.24
12.59
4.35
2.50 DMSO-d6
F
^{13}C
OH
O
N
NH
O
^{1}H NMR (500 MHz, DMSO-d_6)
0.83
0.99
2.00
13.5 13.0 12.5 12.0 11.5 11.0 10.5 10.0 9.5 9.0 8.5 8.0 7.5 7.0 6.5 6.0 5.5 5.0 4.5 4.0 3.5 3.0 2.5 2.0 1.5 1.0 0.5 0.0 -0.5
f1 (ppm)

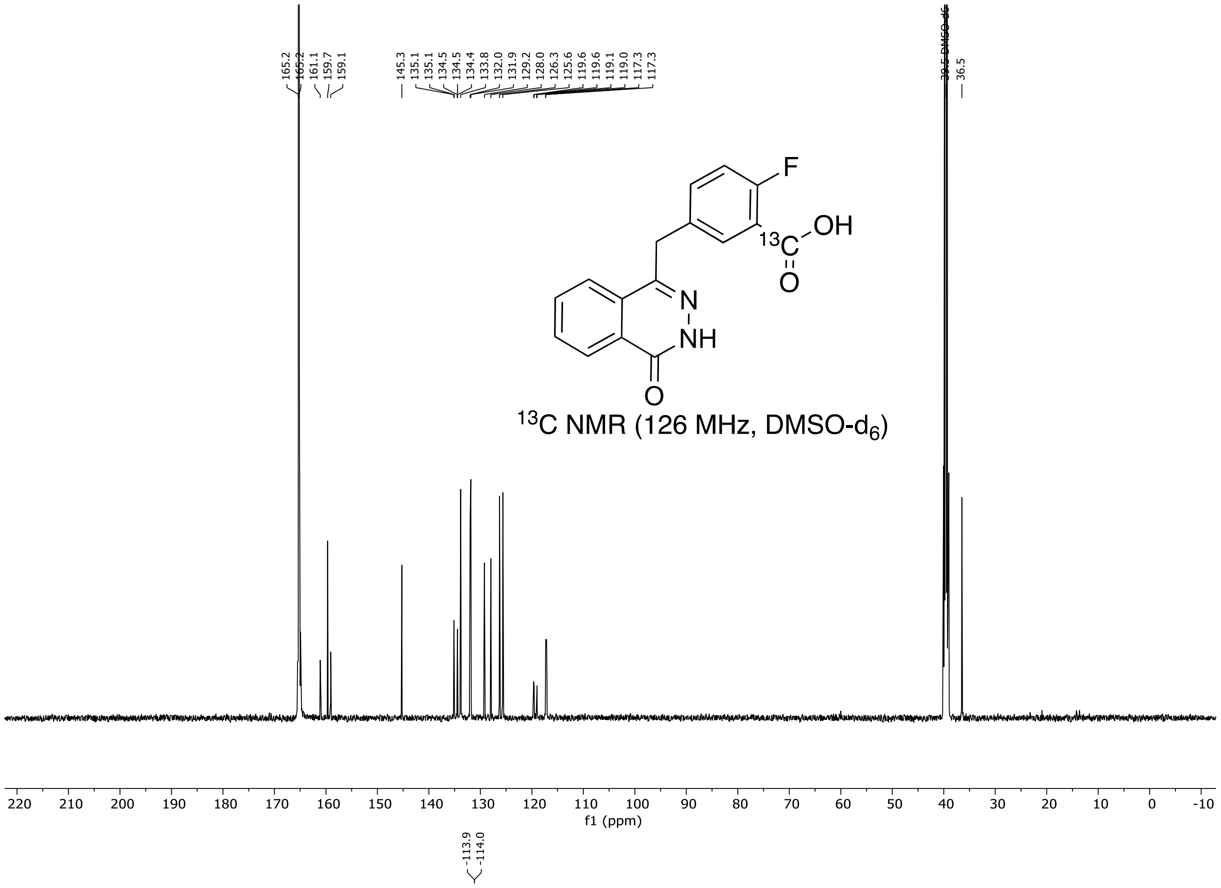
165.2
165.2
161.1
159.7
159.1
145.3
135.1
135.1
134.5
134.5
134.4
133.8
132.0
131.9
129.2
128.0
126.3
125.6
119.6
119.6
119.1
119.0
117.3
117.3
39.5 DMSO-d6
36.5
F
OH
^{13}C
O
N
NH
O
^{13}C NMR (126 MHz, DMSO-d_6)
f1 (ppm)
-113.9
-114.0

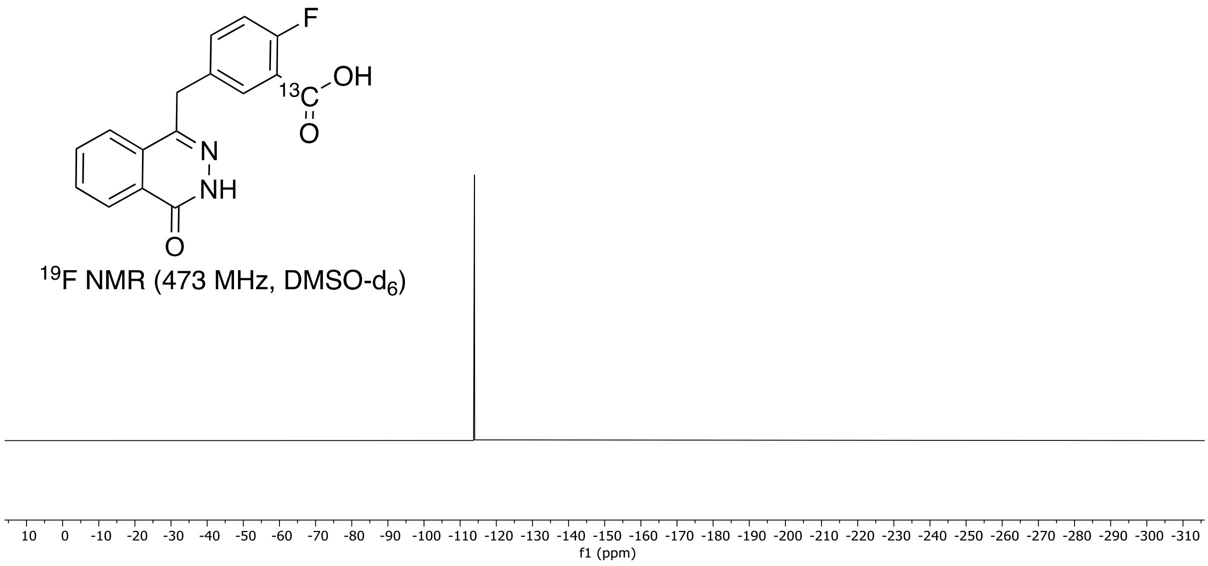
F
OH
^{13}C
O
N
NH
O
^{19}F NMR (473 MHz, DMSO-d_6)
f1 (ppm)

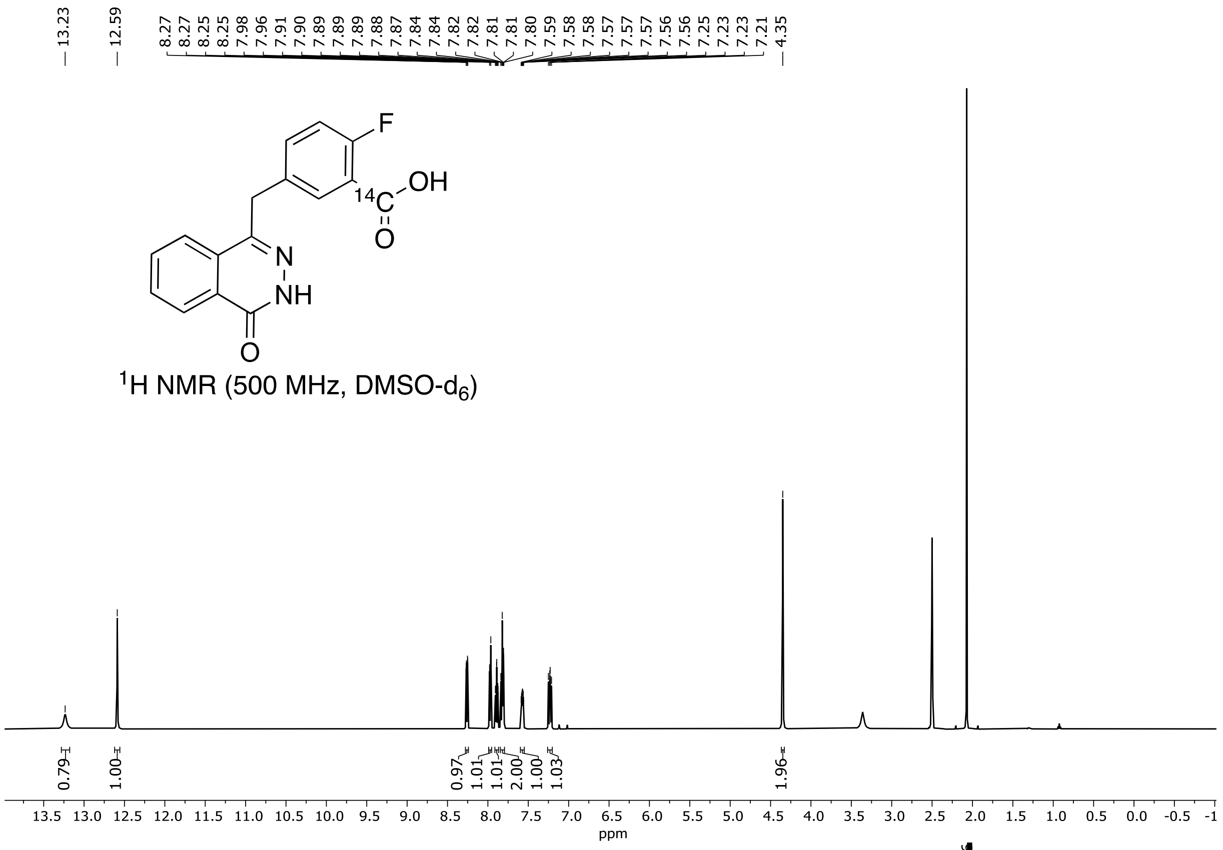
F
OH
14C
O
N
NH
O
1H NMR (500 MHz, DMSO-d6)
13.23
12.59
8.27 8.27 8.25 8.25 7.98 7.96 7.91 7.90 7.89 7.89 7.89 7.88 7.87 7.84 7.84 7.82 7.82 7.81 7.81 7.80 7.59 7.58 7.58 7.57 7.57 7.57 7.56 7.56 7.25 7.23 7.23 7.21
4.35
0.79
1.00
0.97 1.01 1.01 2.00 1.00 1.03
1.96
13.5 13.0 12.5 12.0 11.5 11.0 10.5 10.0 9.5 9.0 8.5 8.0 7.5 7.0 6.5 6.0 5.5 5.0 4.5 4.0 3.5 3.0 2.5 2.0 1.5 1.0 0.5 0.0 -0.5 -1
ppm

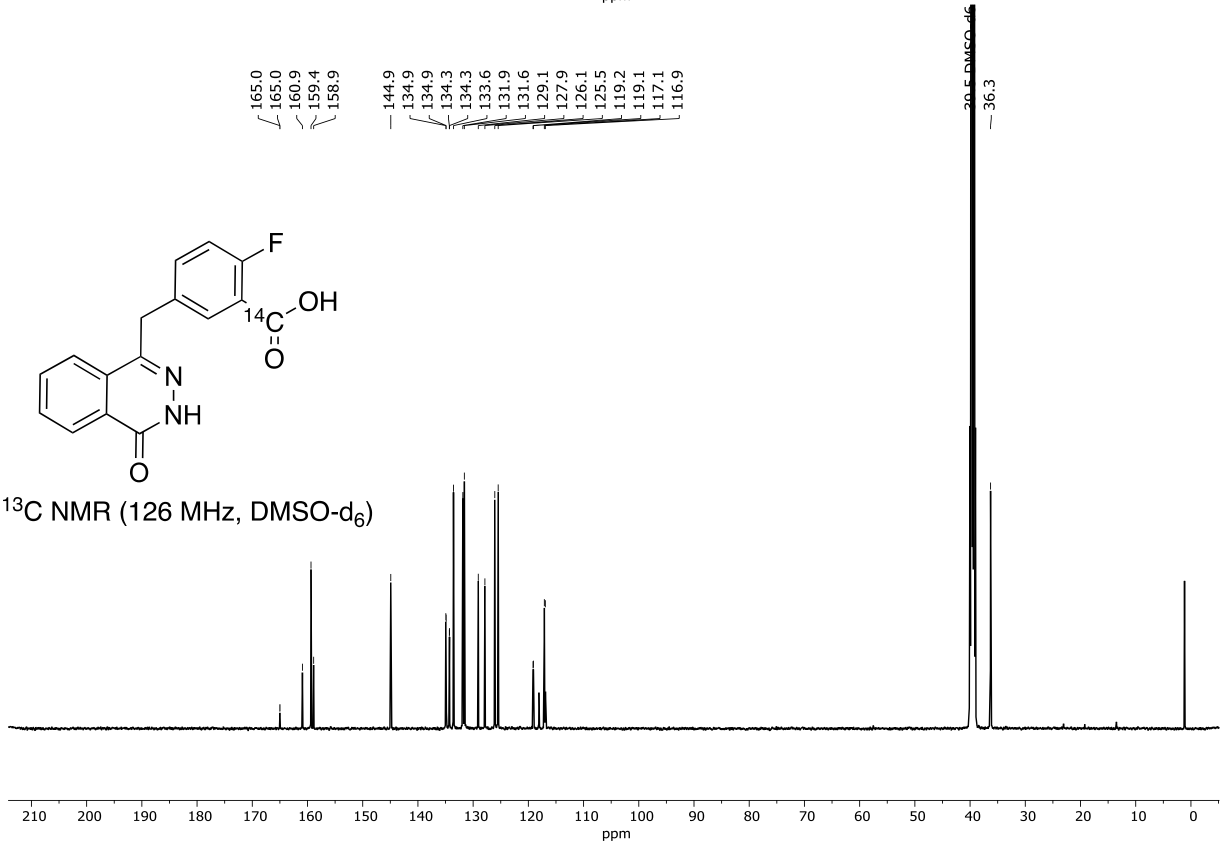
F
OH
14C
O
N
NH
O
13C NMR (126 MHz, DMSO-d6)
165.0 165.0 160.9 159.4 158.9
144.9 134.9 134.9 134.3 134.3 133.6 131.9 131.6 129.1 127.9 126.1 125.5 119.2 119.1 117.1 116.9
39.5 DMSO-d6
36.3
210 200 190 180 170 160 150 140 130 120 110 100 90 80 70 60 50 40 30 20 10 0
ppm

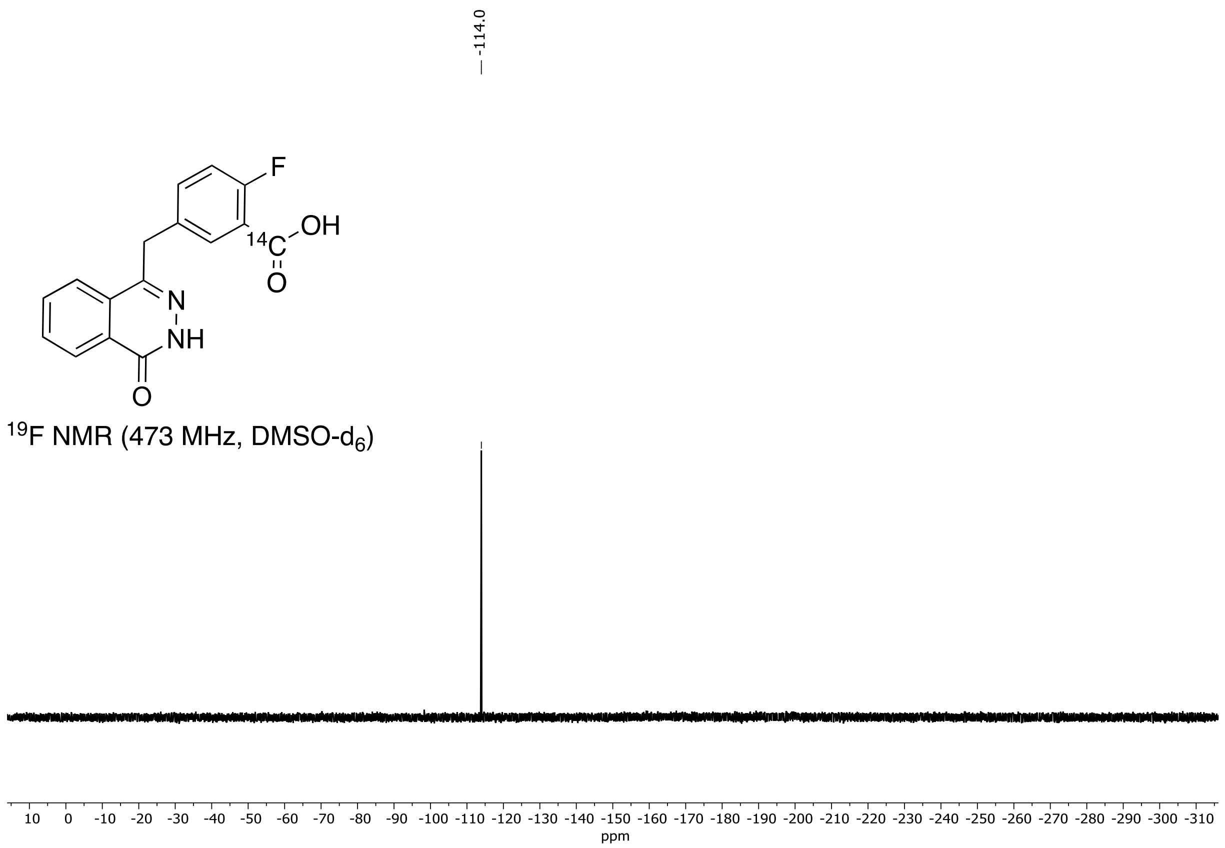

Chromatogram: UV

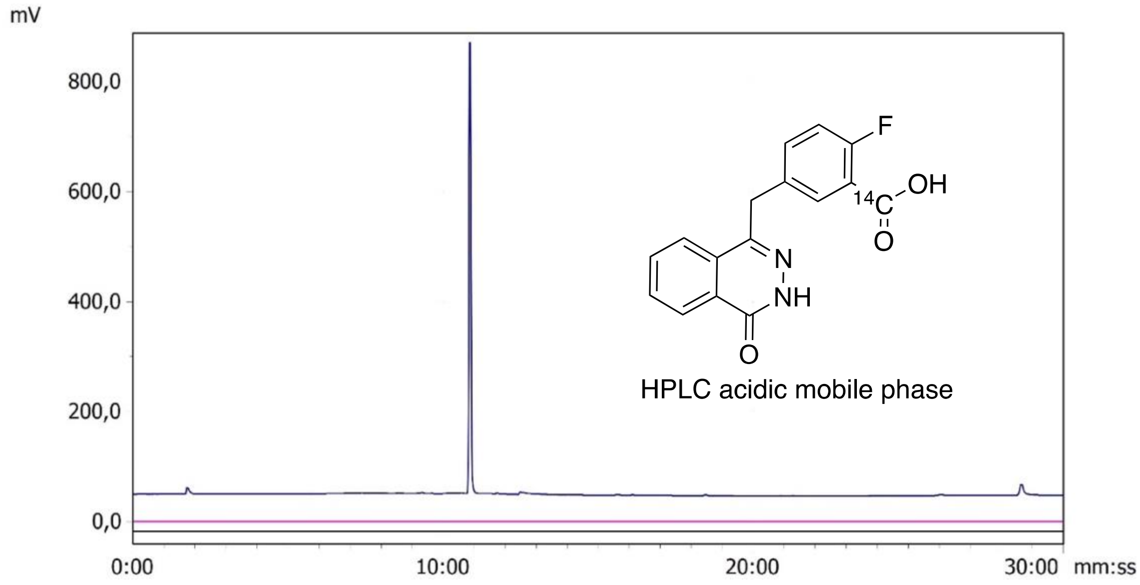

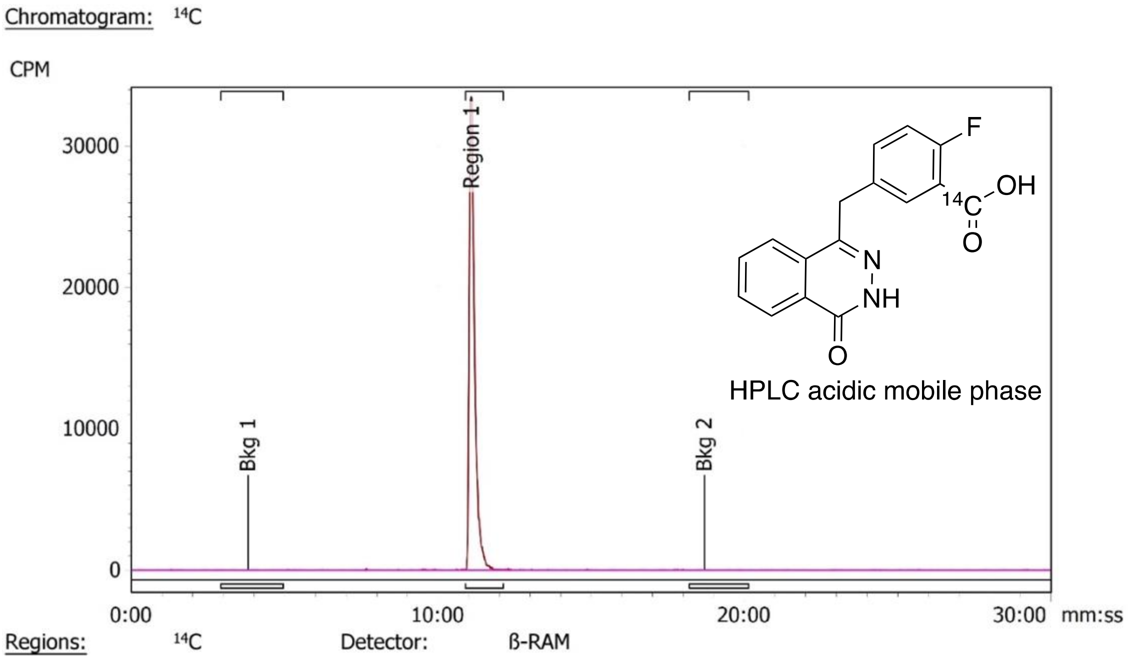
Chromatogram: 14C
CPM
30000
20000
10000
0
Region 1
Bkg 1
Bkg 2
0:00
10:00
20:00
30:00
mm:ss
HPLC acidic mobile phase
Regions: 14C
Detector: ß-RAM

RCP > 99%

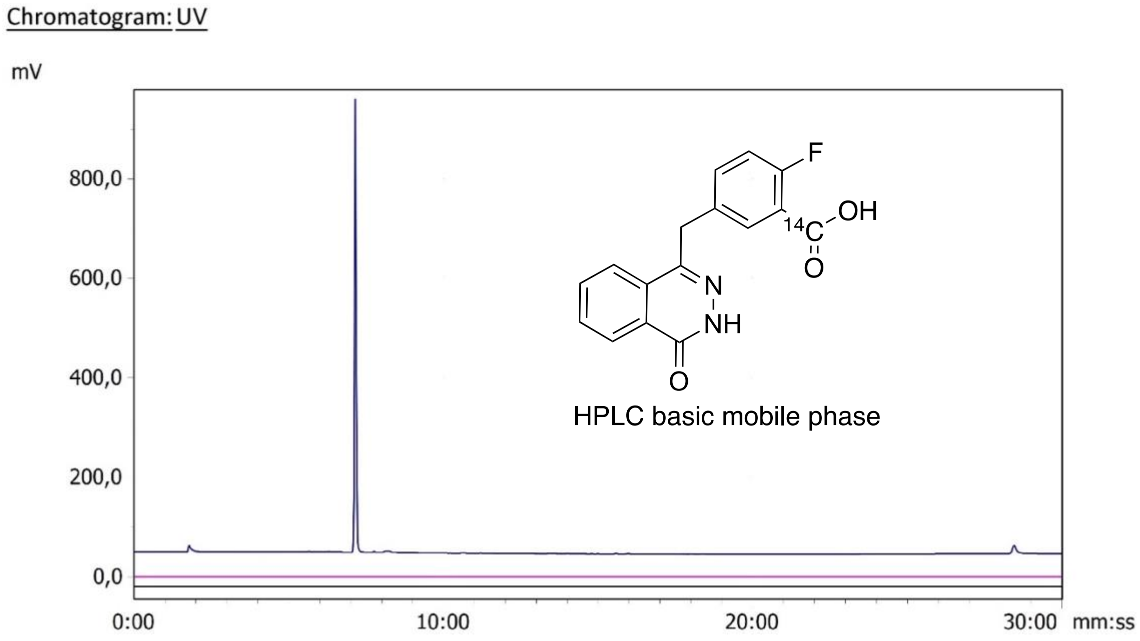
Chromatogram: UV
mV
800,0
600,0
400,0
200,0
0,0
0:00
10:00
20:00
30:00
mm:ss
HPLC basic mobile phase

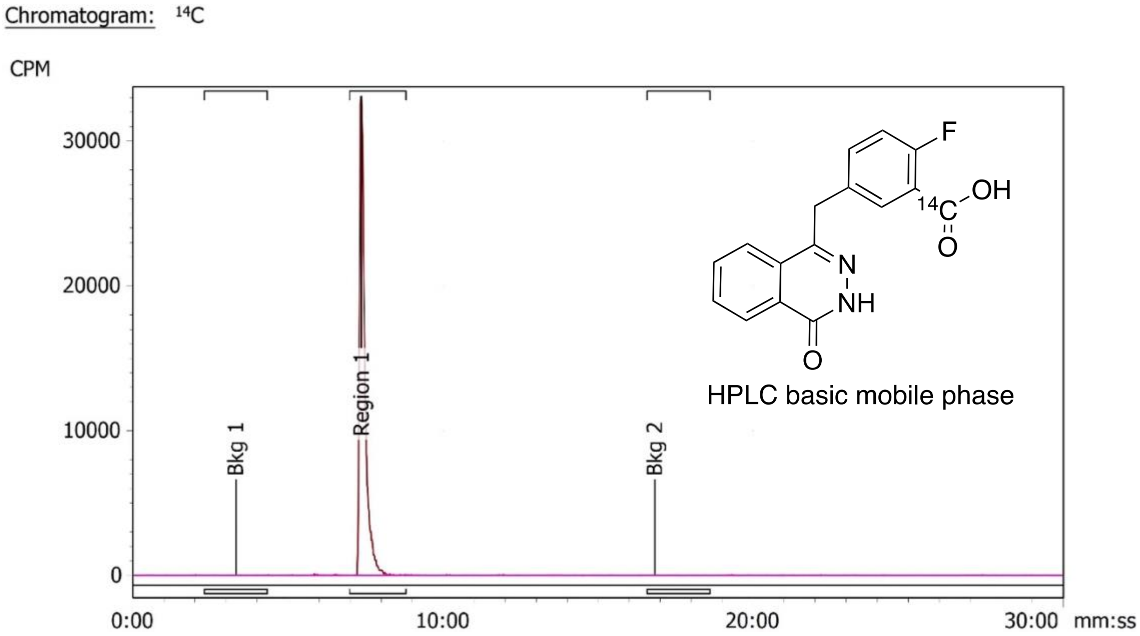
Chromatogram: 14C
CPM
30000
20000
10000
0
0:00
10:00
20:00
30:00
mm:ss
Bkg 1
Region 1
Bkg 2
F
OH
14C
O
N
NH
O
HPLC basic mobile phase

RCP > 99%

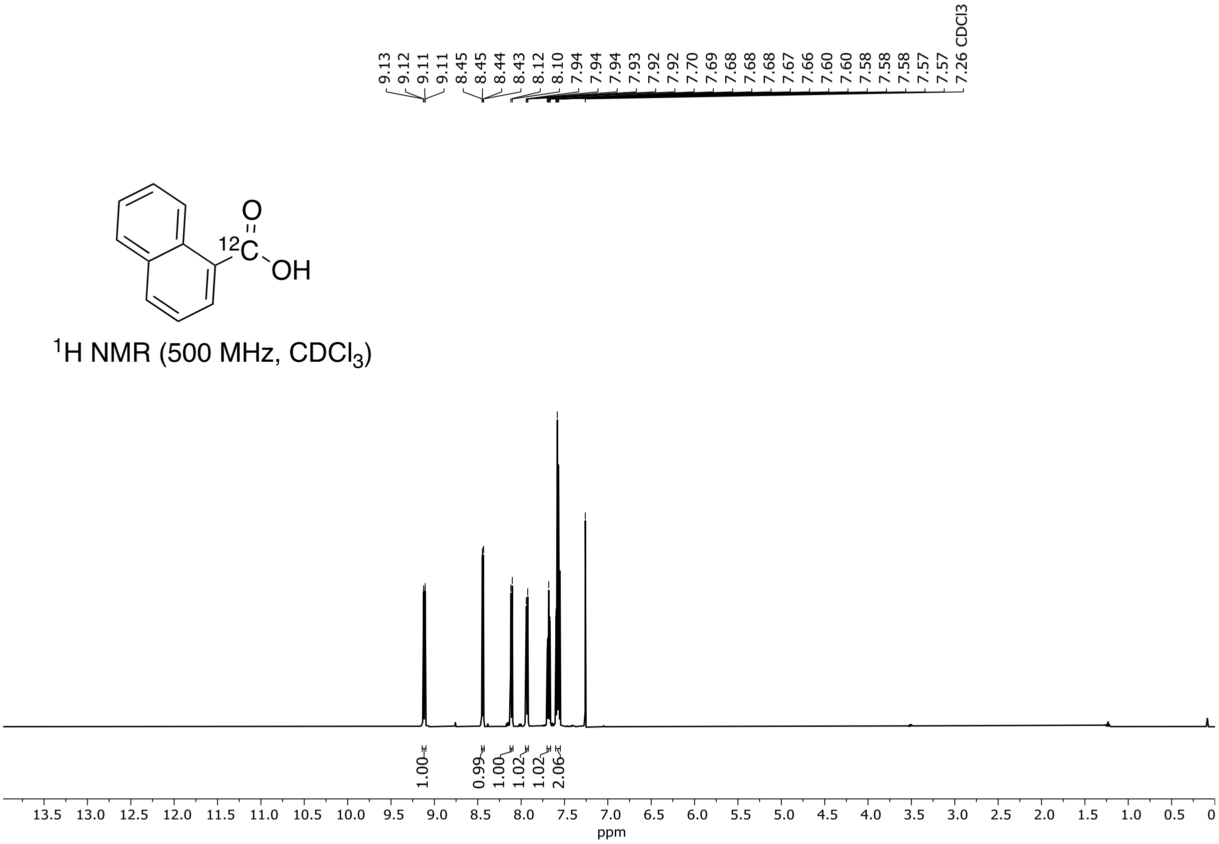
9.13
9.12
9.11
9.11
8.45
8.45
8.44
8.43
8.12
8.10
7.94
7.94
7.94
7.93
7.92
7.92
7.70
7.69
7.68
7.68
7.68
7.67
7.66
7.60
7.60
7.58
7.58
7.58
7.57
7.57
7.26 CDCl3
O
12C
OH
1H NMR (500 MHz, CDCl3)
1.00
0.99
1.00
1.02
1.02
2.06
13.5
13.0
12.5
12.0
11.5
11.0
10.5
10.0
9.5
9.0
8.5
8.0
7.5
7.0
6.5
6.0
5.5
5.0
4.5
4.0
3.5
3.0
2.5
2.0
1.5
1.0
0.5
0
ppm

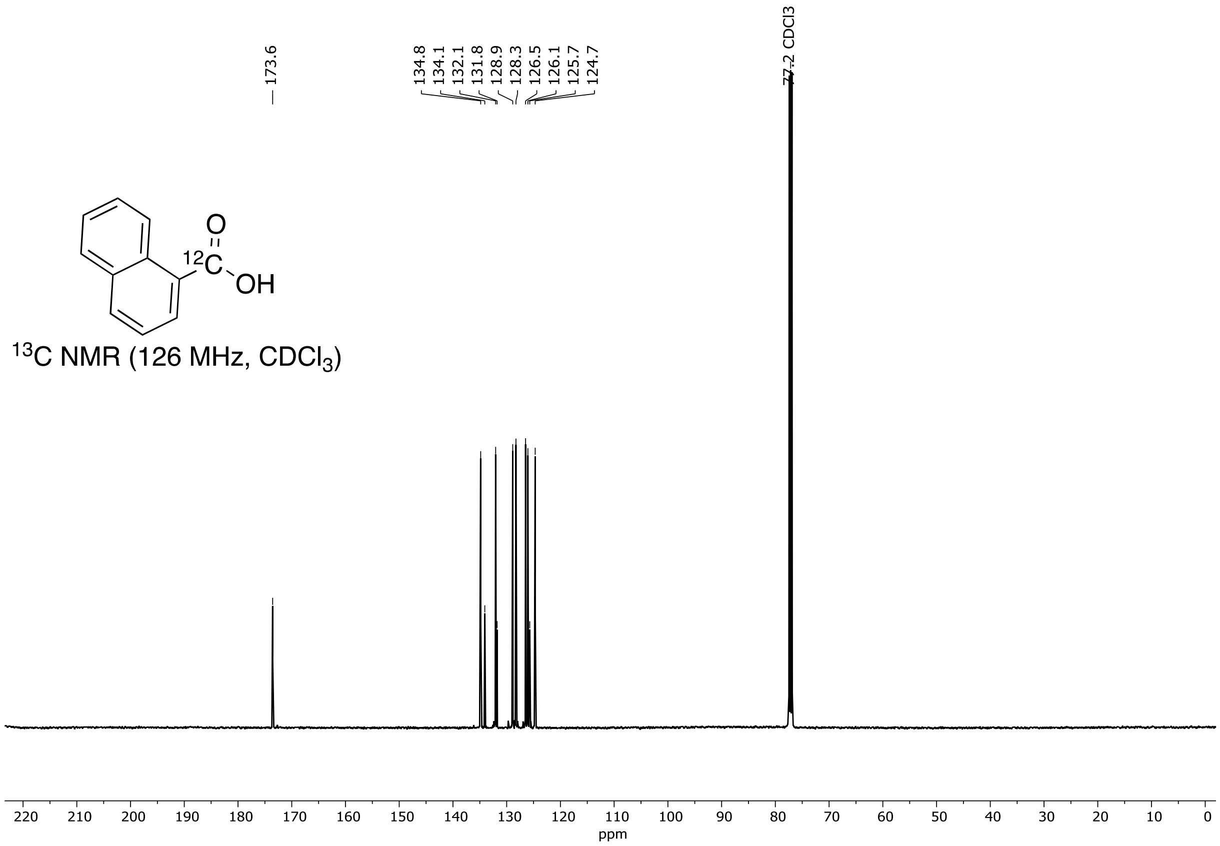
173.6
134.8
134.1
132.1
131.8
128.9
128.3
126.5
126.1
125.7
124.7
77.2 CDCl3
O
12C
OH
13C NMR (126 MHz, CDCl3)
220 210 200 190 180 170 160 150 140 130 120 110 100 90 80 70 60 50 40 30 20 10 0
ppm

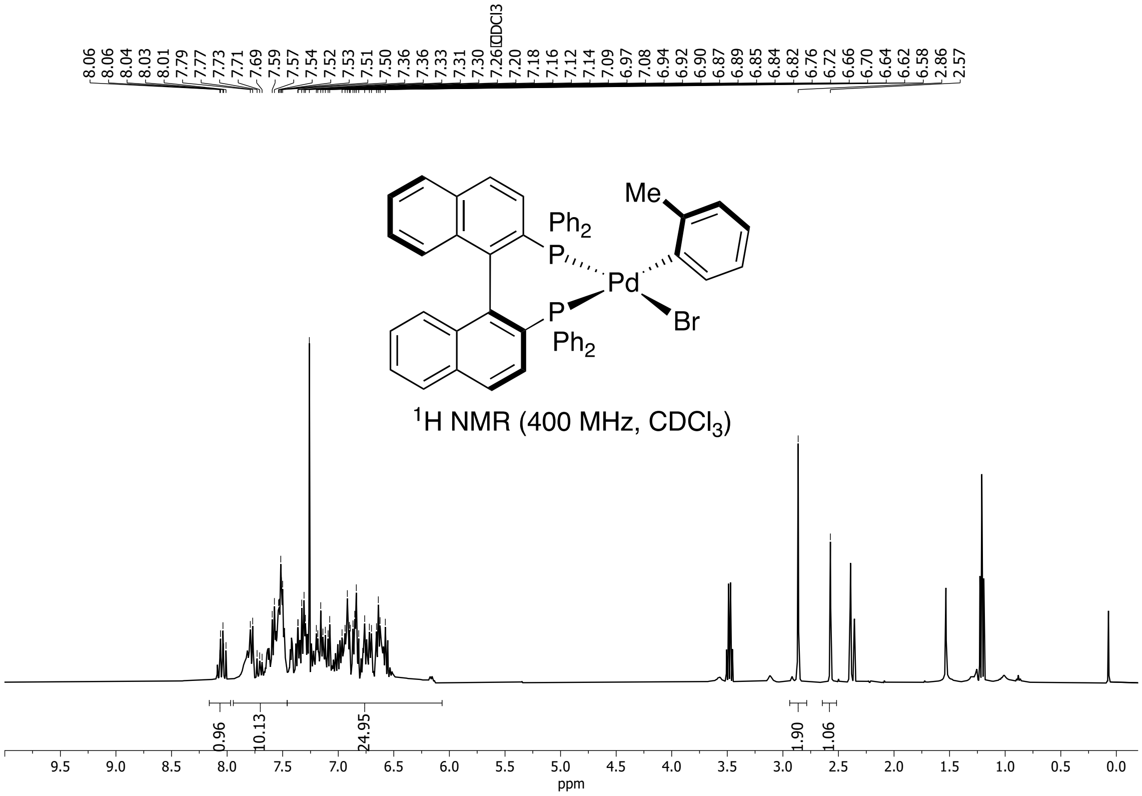
8.06 8.06 8.04 8.03 8.01 7.79 7.77 7.73 7.71 7.69 7.59 7.57 7.54 7.52 7.53 7.51 7.50 7.36 7.36 7.33 7.31 7.30 7.26 CDCl3 7.20 7.18 7.16 7.12 7.14 7.09 7.08 6.97 6.94 6.92 6.90 6.87 6.89 6.85 6.84 6.82 6.76 6.72 6.66 6.70 6.64 6.62 6.58 2.86 2.57
Me
Ph2
P
Pd
P
Br
Ph2
1H NMR (400 MHz, CDCl3)
0.96
10.13
24.95
1.90
1.06
9.5 9.0 8.5 8.0 7.5 7.0 6.5 6.0 5.5 5.0 4.5 4.0 3.5 3.0 2.5 2.0 1.5 1.0 0.5 0.0
ppm

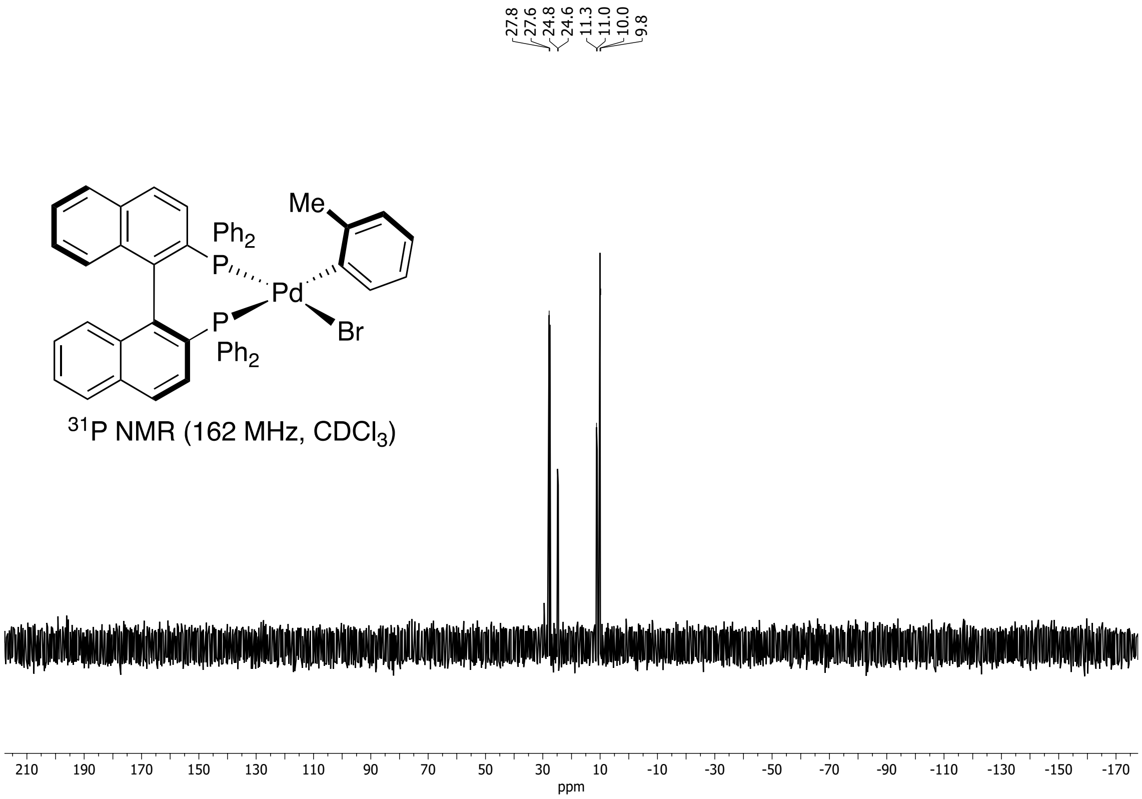
27.8
27.6
24.8
24.6
11.3
11.0
10.0
9.8
Me
Ph2
P
Pd
Br
Ph2
31P NMR (162 MHz, CDCl3)
210 190 170 150 130 110 90 70 50 30 10 -10 -30 -50 -70 -90 -110 -130 -150 -170
ppm

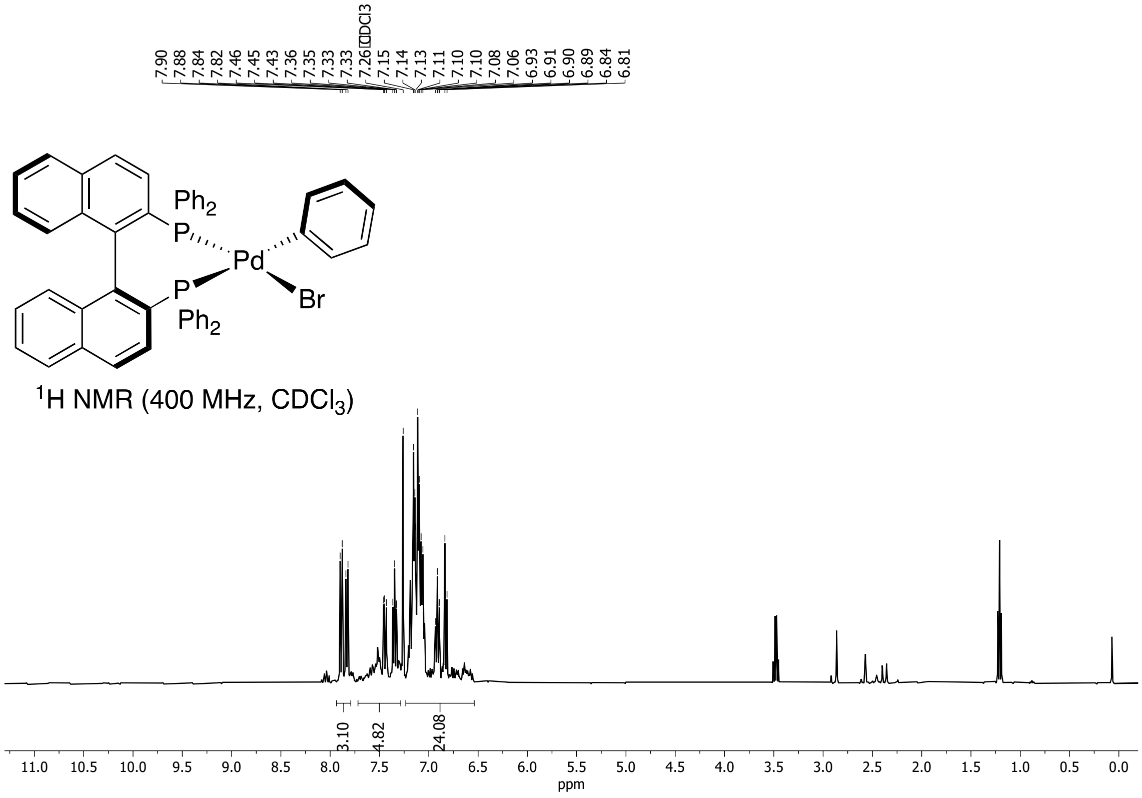
7.90
7.88
7.84
7.82
7.46
7.45
7.43
7.36
7.35
7.33
7.33
7.26 CDCl3
7.15
7.14
7.13
7.11
7.10
7.10
7.08
7.06
6.93
6.91
6.90
6.89
6.84
6.81
Ph2
P
Pd
Br
Ph2
1H NMR (400 MHz, CDCl3)
3.10
4.82
24.08
11.0 10.5 10.0 9.5 9.0 8.5 8.0 7.5 7.0 6.5 6.0 5.5 5.0 4.5 4.0 3.5 3.0 2.5 2.0 1.5 1.0 0.5 0.0
ppm

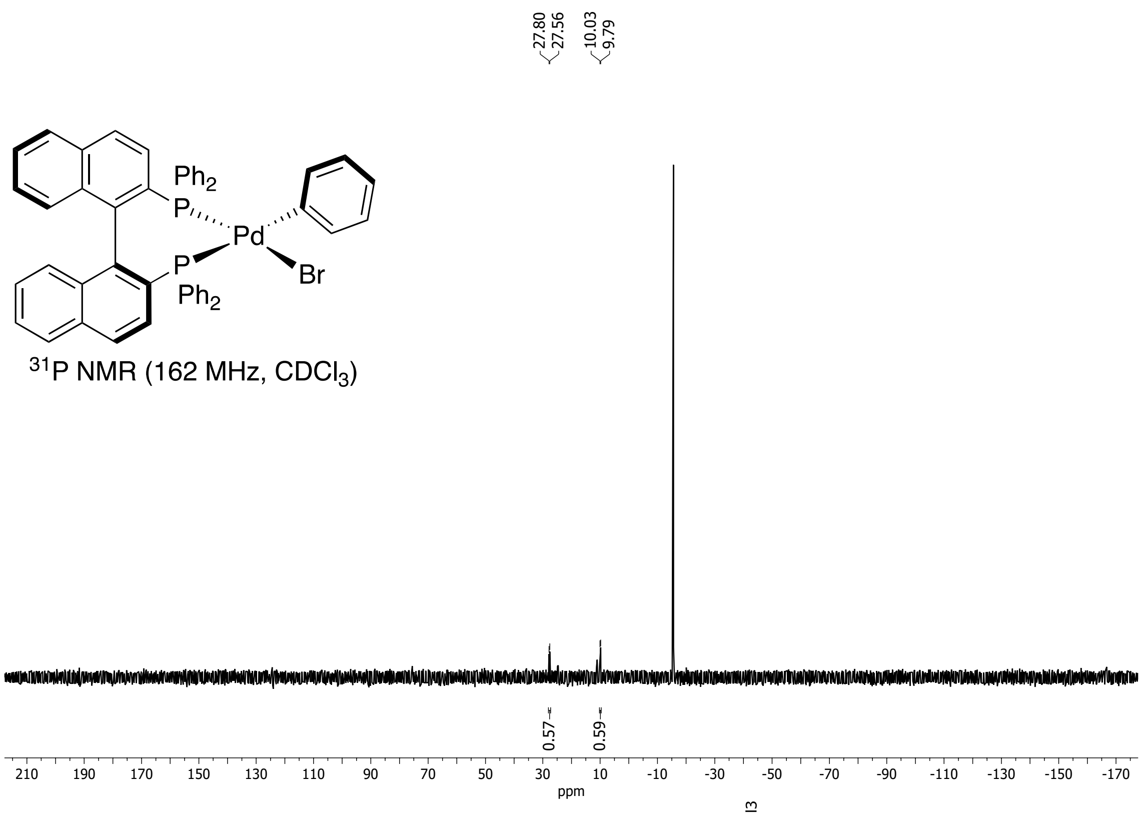

Ph2
P
Pd
Br
P
Ph2
31P NMR (162 MHz, CDCl3)
27.80
27.56
10.03
9.79
0.57
0.59
ppm

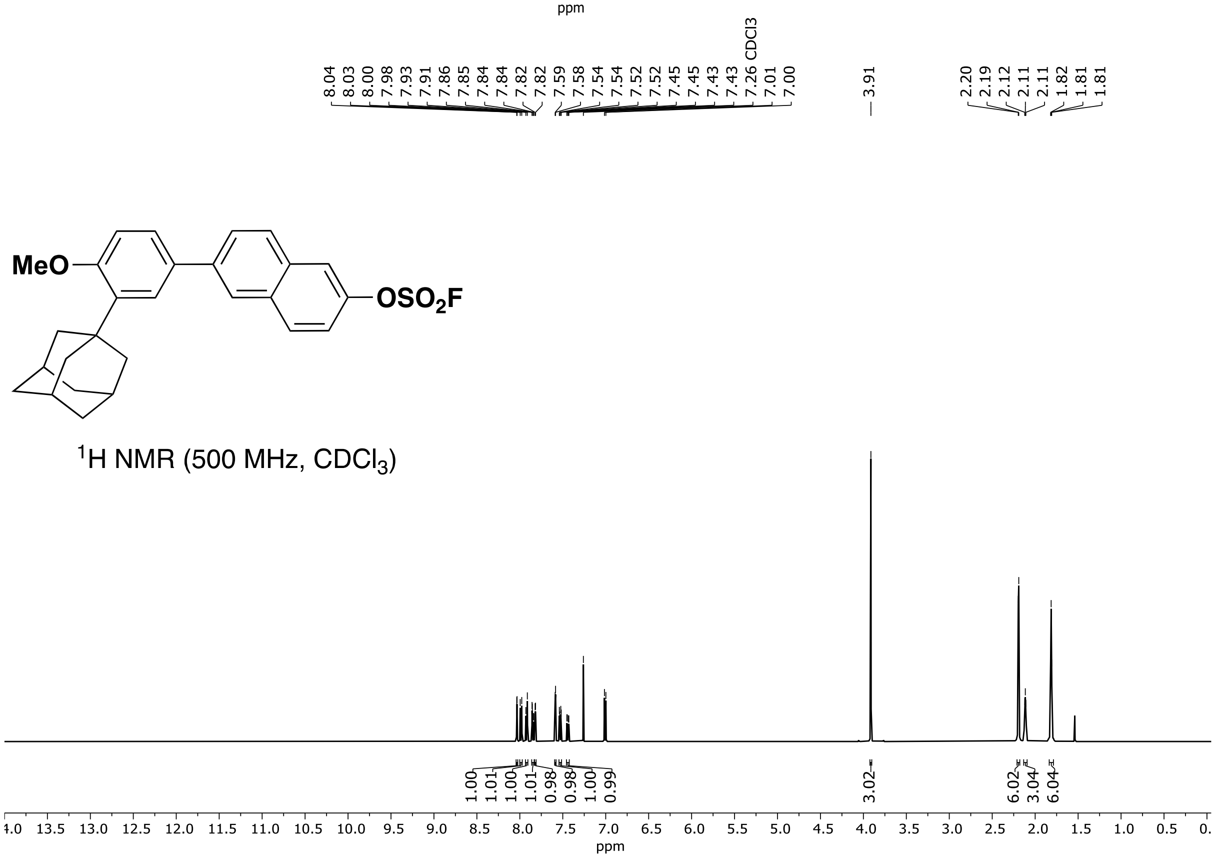

MeO
OSO2F
1H NMR (500 MHz, CDCl3)
CDCl3
3.91
3.02
6.02
3.04
6.04
ppm

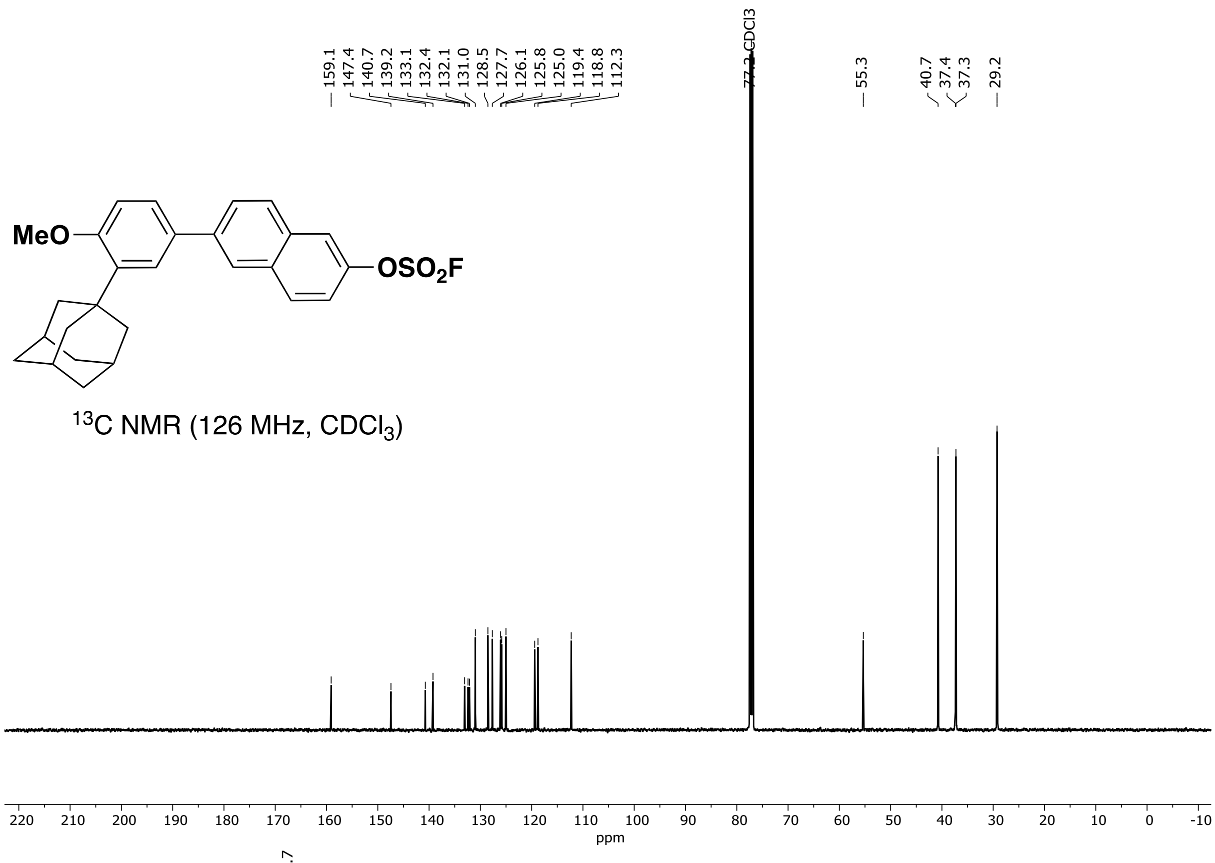
159.1
147.4
140.7
139.2
133.1
132.4
132.1
131.0
128.5
127.7
126.1
125.8
125.0
119.4
118.8
112.3
77.2 CDCl3
55.3
40.7
37.4
37.3
29.2
MeO
OSO_2F
^{13}C NMR (126 MHz, $CDCl_3$)
ppm

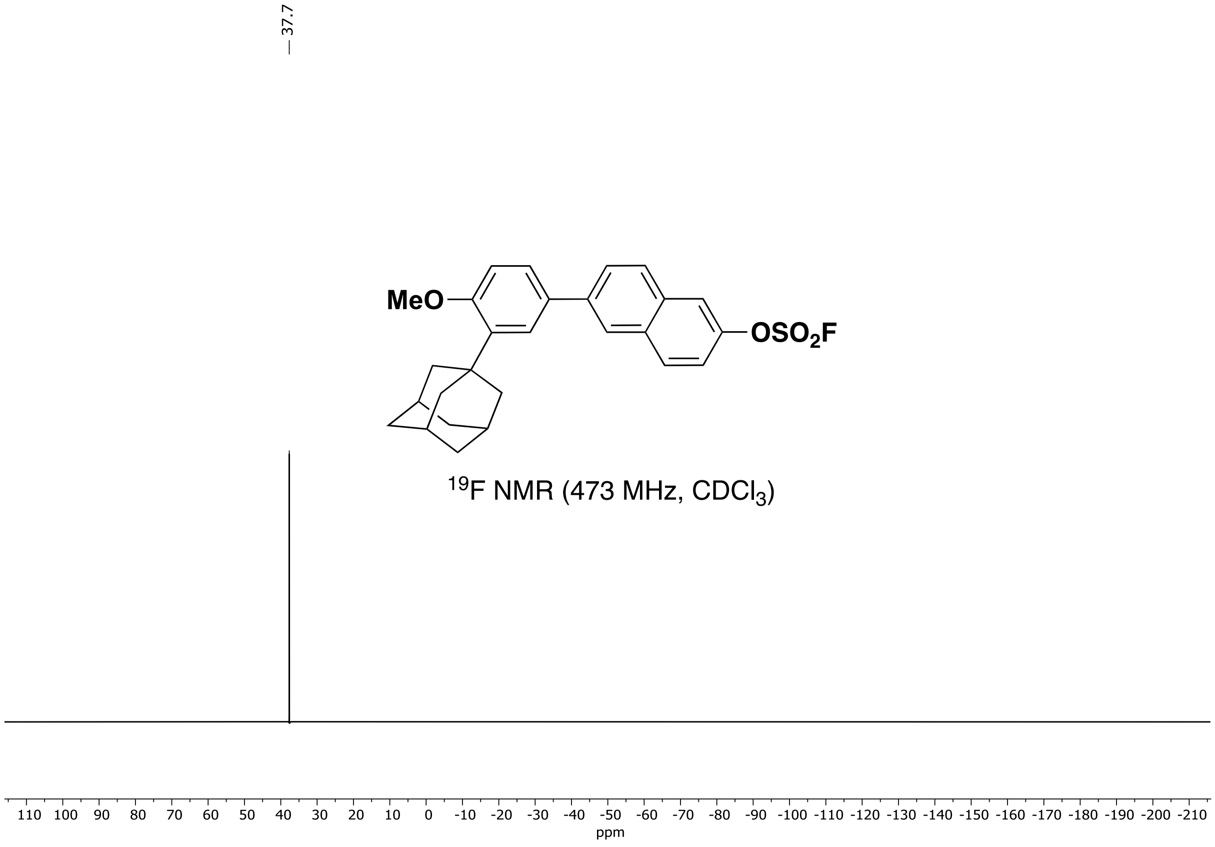
-37.7
MeO
OSO_2F
^{19}F NMR (473 MHz, $CDCl_3$)
ppm

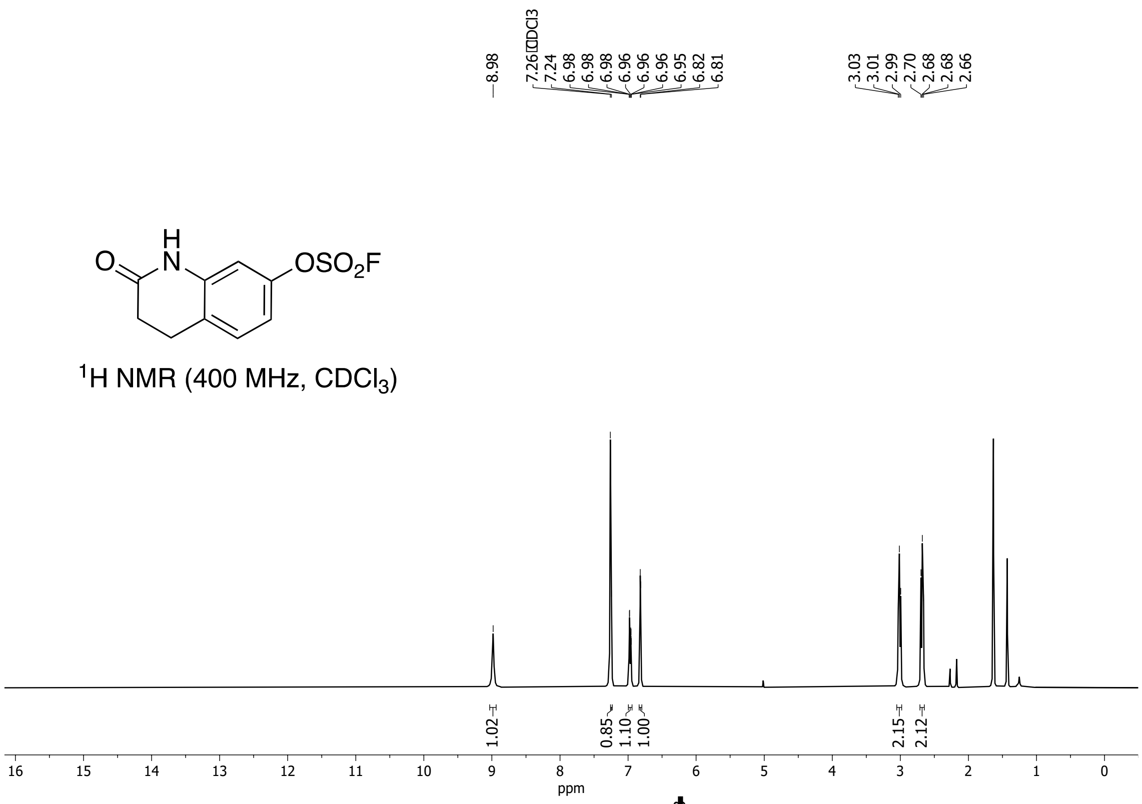

1H NMR (400 MHz, CDCl3)
OSO2F
8.98
7.26 CDCl3
7.24
6.98
6.98
6.98
6.96
6.96
6.96
6.95
6.82
6.81
3.03
3.01
2.99
2.70
2.68
2.68
2.66
1.02
0.85
1.10
1.00
2.15
2.12
ppm

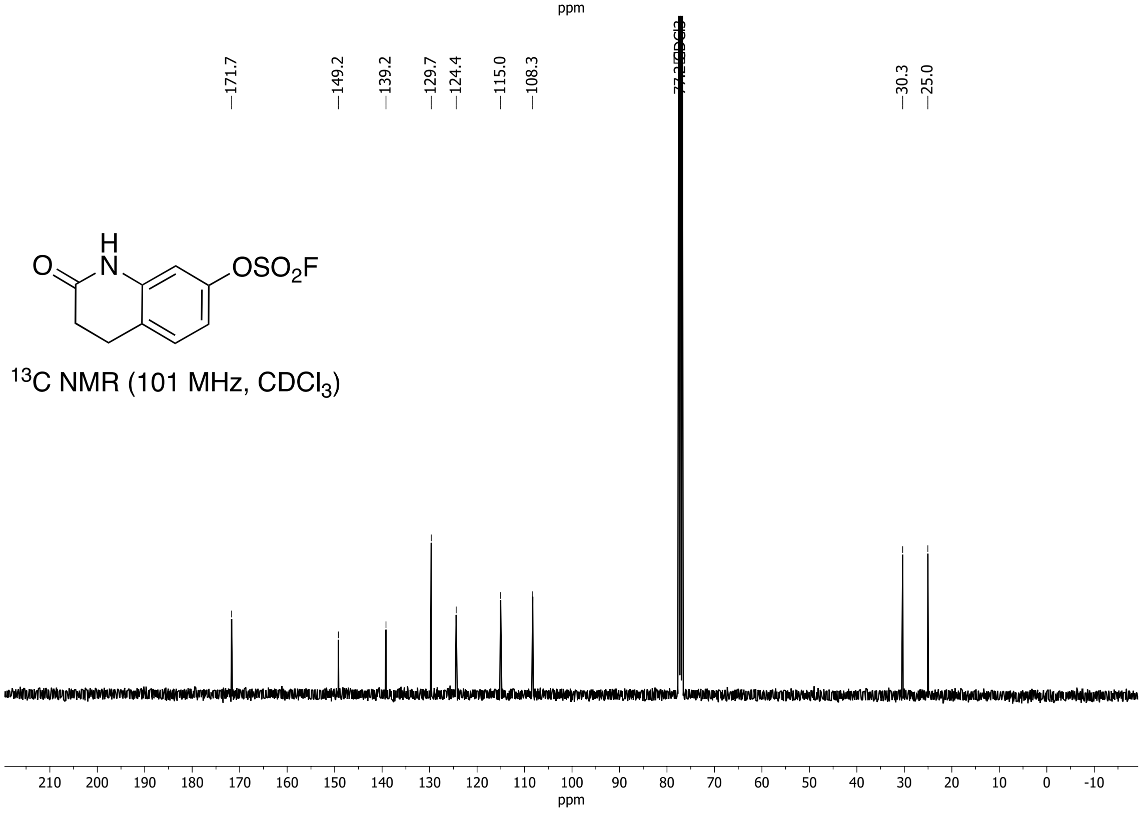

13C NMR (101 MHz, CDCl3)
OSO2F
171.7
149.2
139.2
129.7
124.4
115.0
108.3
30.3
25.0
ppm

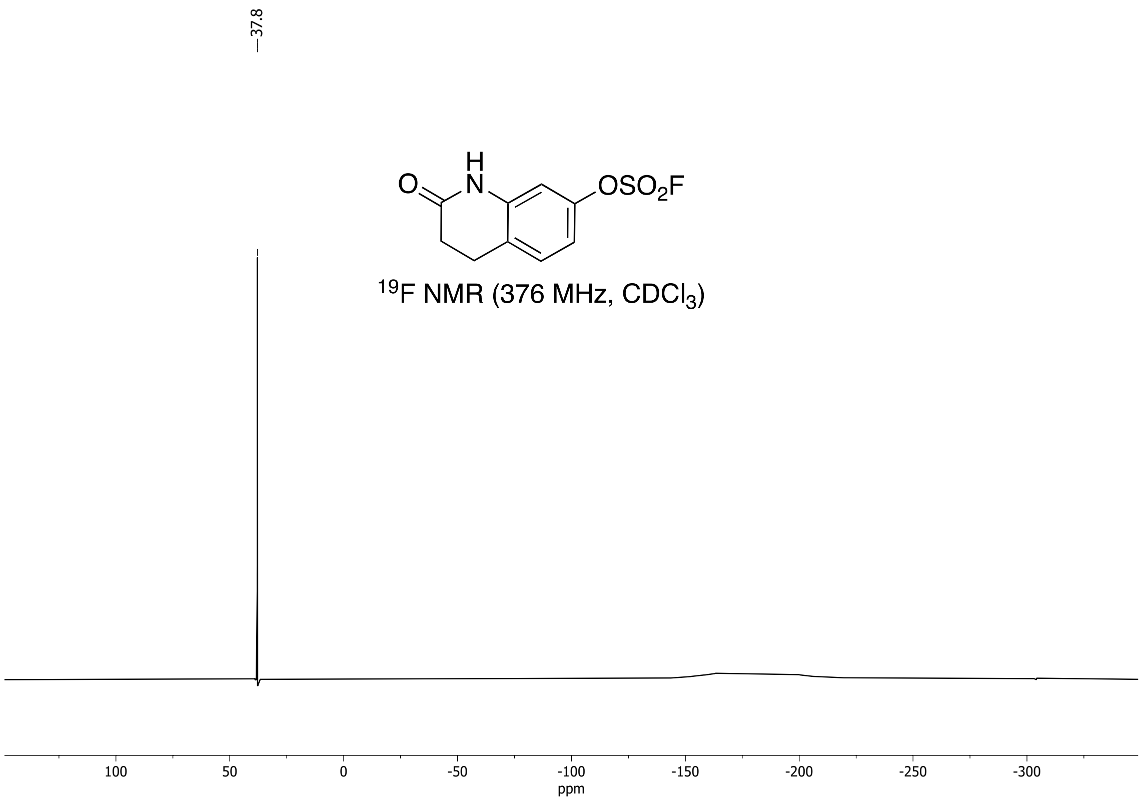

—37.8
H
N
O
OSO2F
19F NMR (376 MHz, CDCl3)
100
50
0
-50
-100
-150
-200
-250
-300
ppm

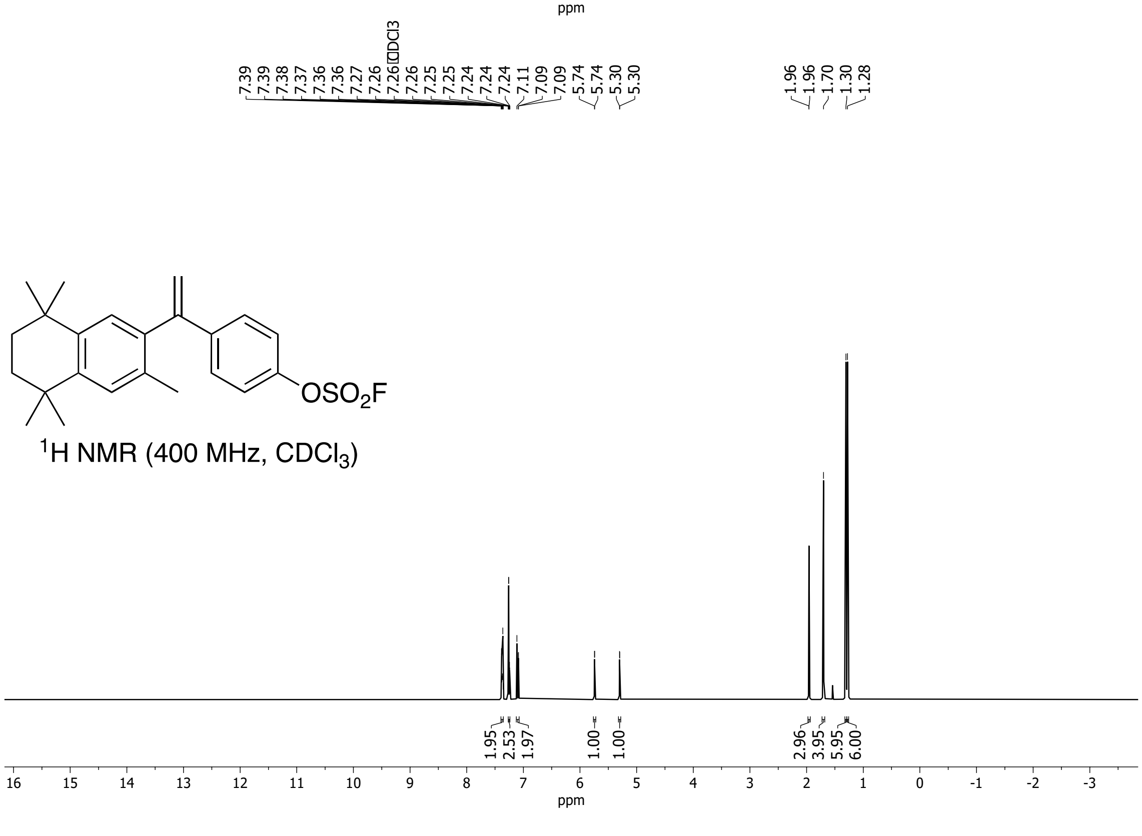

7.39
7.39
7.38
7.37
7.36
7.36
7.27
7.26
7.26 CDCl3
7.26
7.25
7.25
7.24
7.24
7.24
7.11
7.09
7.09
5.74
5.74
5.30
5.30
1.96
1.96
1.70
1.30
1.28
OSO2F
1H NMR (400 MHz, CDCl3)
1.95
2.53
1.97
1.00
1.00
2.96
3.95
5.95
6.00
16
15
14
13
12
11
10
9
8
7
6
5
4
3
2
1
0
-1
-2
-3
ppm

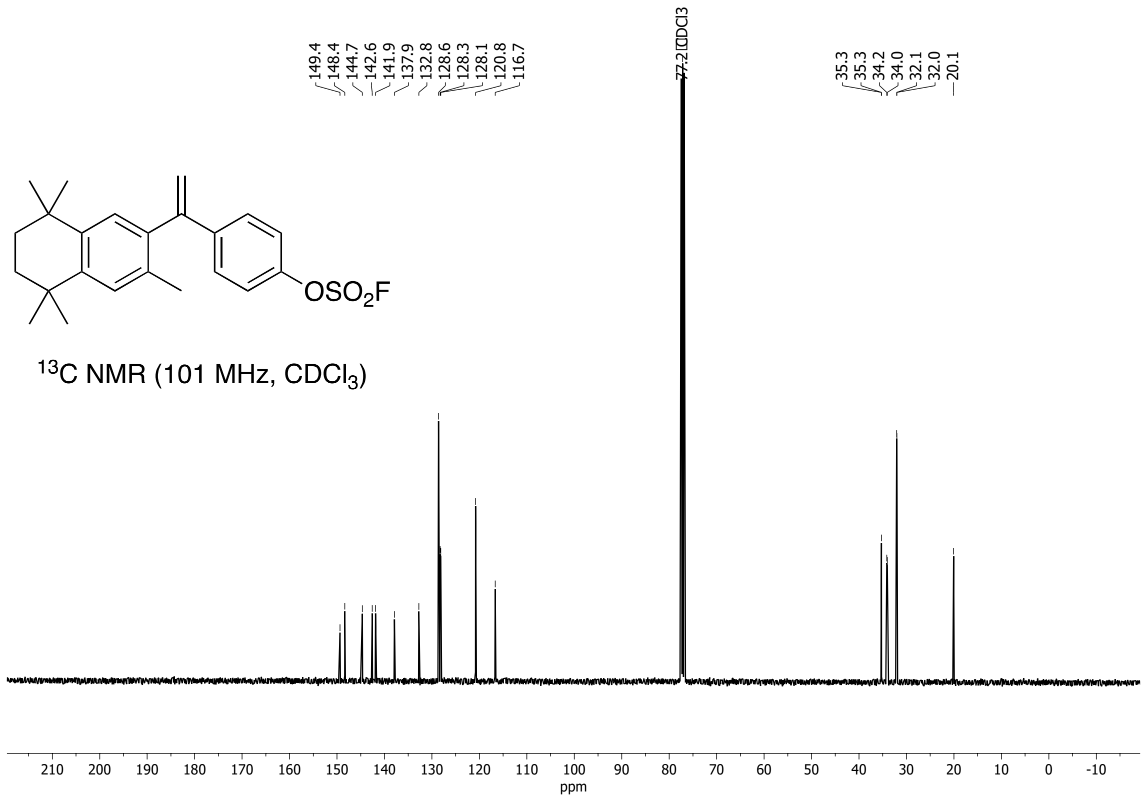
149.4
148.4
144.7
142.6
141.9
137.9
132.8
128.6
128.3
128.1
120.8
116.7
77.2 CDCl3
35.3
35.3
34.2
34.0
32.1
32.0
20.1
OSO2F
13C NMR (101 MHz, CDCl3)
210 200 190 180 170 160 150 140 130 120 110 100 90 80 70 60 50 40 30 20 10 0 -10
ppm

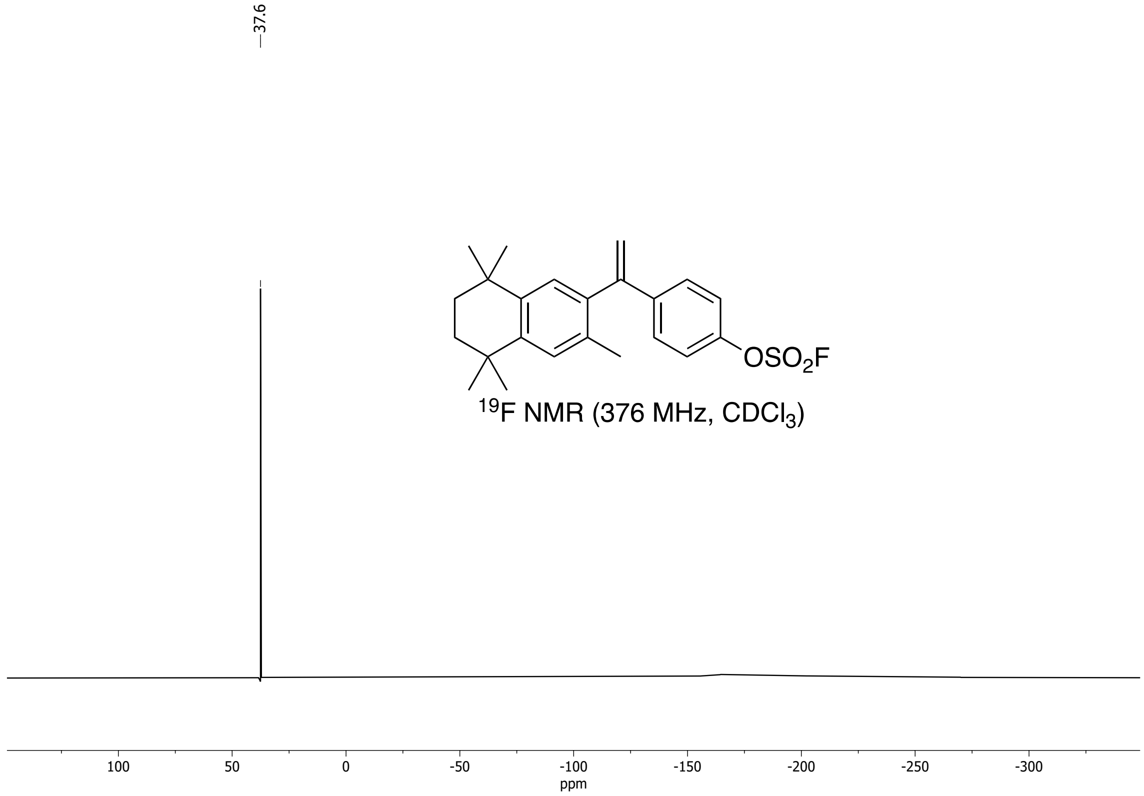
37.6
OSO2F
19F NMR (376 MHz, CDCl3)
100 50 0 -50 -100 -150 -200 -250 -300
ppm

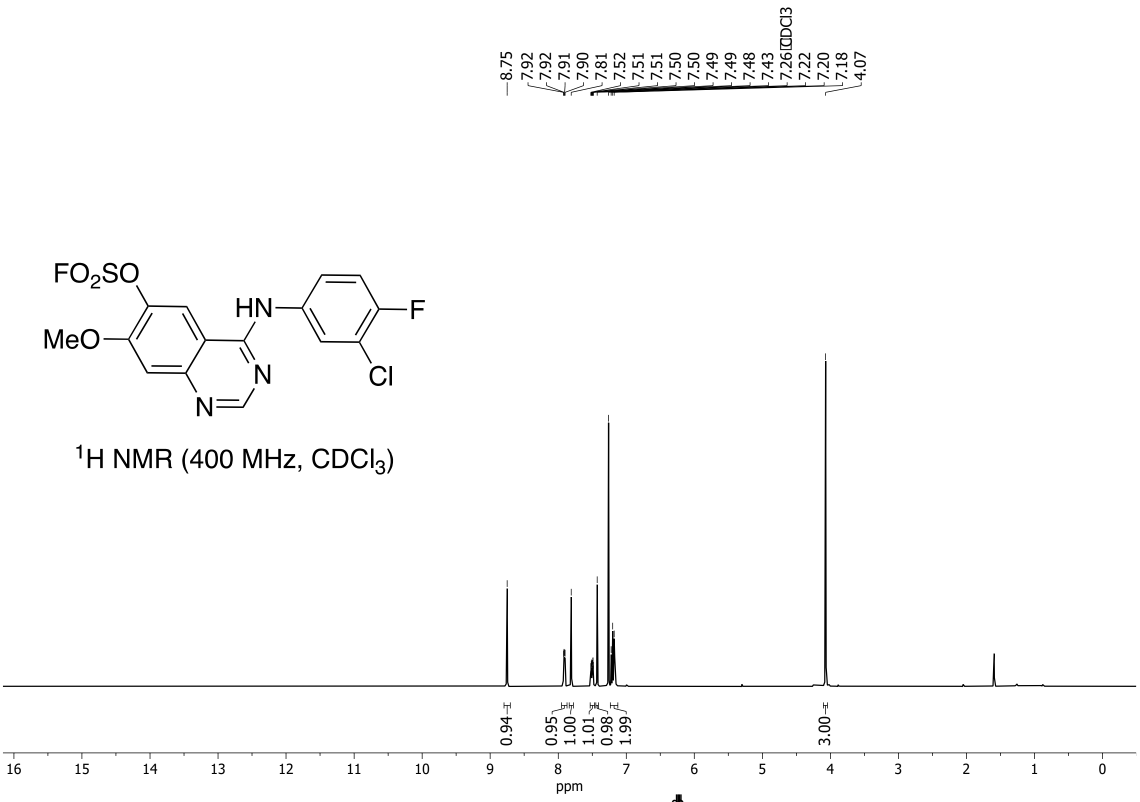
8.75
7.92
7.92
7.91
7.90
7.81
7.52
7.51
7.51
7.50
7.50
7.49
7.49
7.48
7.43
7.26 CDCl3
7.22
7.20
7.18
4.07
FO2SO
HN
F
MeO
N
Cl
N
1H NMR (400 MHz, CDCl3)
0.94
0.95
1.00
1.01
0.98
1.99
3.00
16 15 14 13 12 11 10 9 8 7 6 5 4 3 2 1 0
ppm

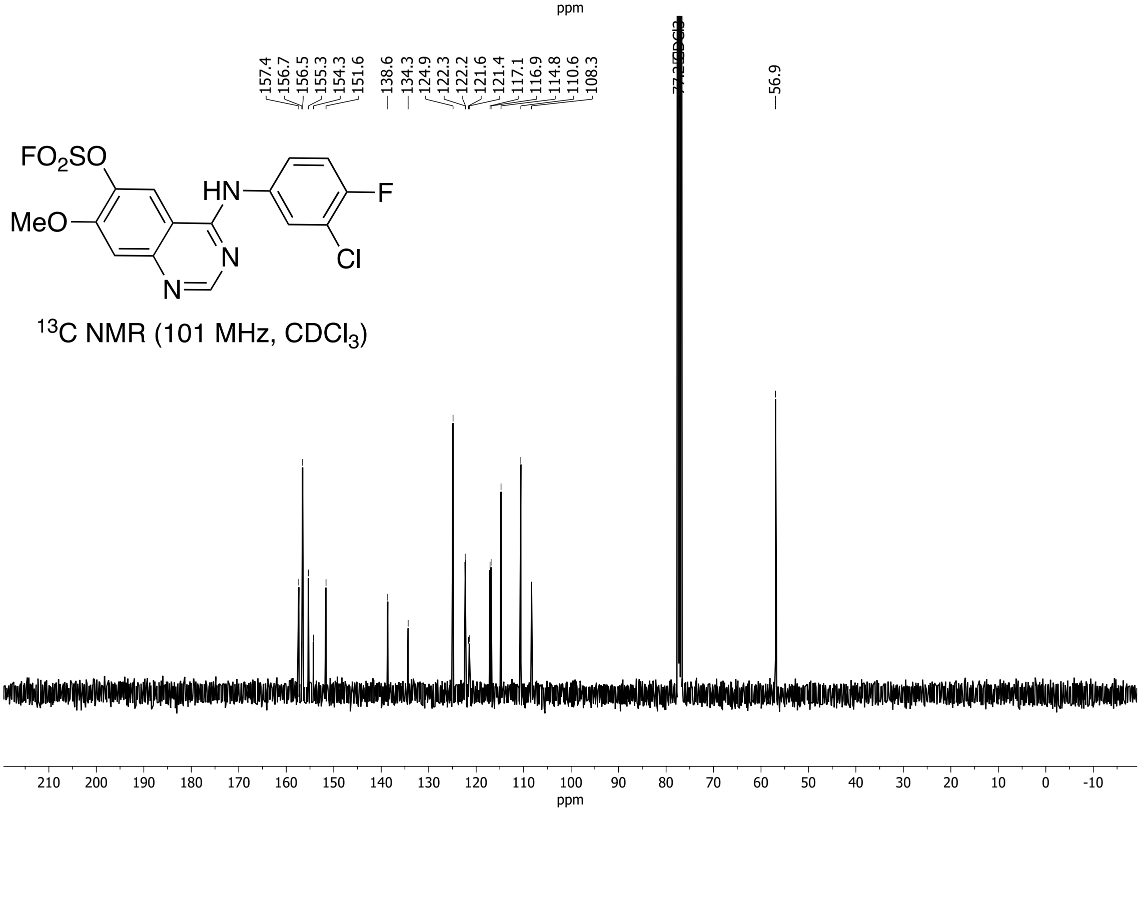
157.4
156.7
156.5
155.3
154.3
151.6
138.6
134.3
124.9
122.3
122.2
121.6
121.4
117.1
116.9
114.8
110.6
108.3
56.9
FO2SO
HN
F
MeO
N
Cl
N
13C NMR (101 MHz, CDCl3)
210 200 190 180 170 160 150 140 130 120 110 100 90 80 70 60 50 40 30 20 10 0 -10
ppm

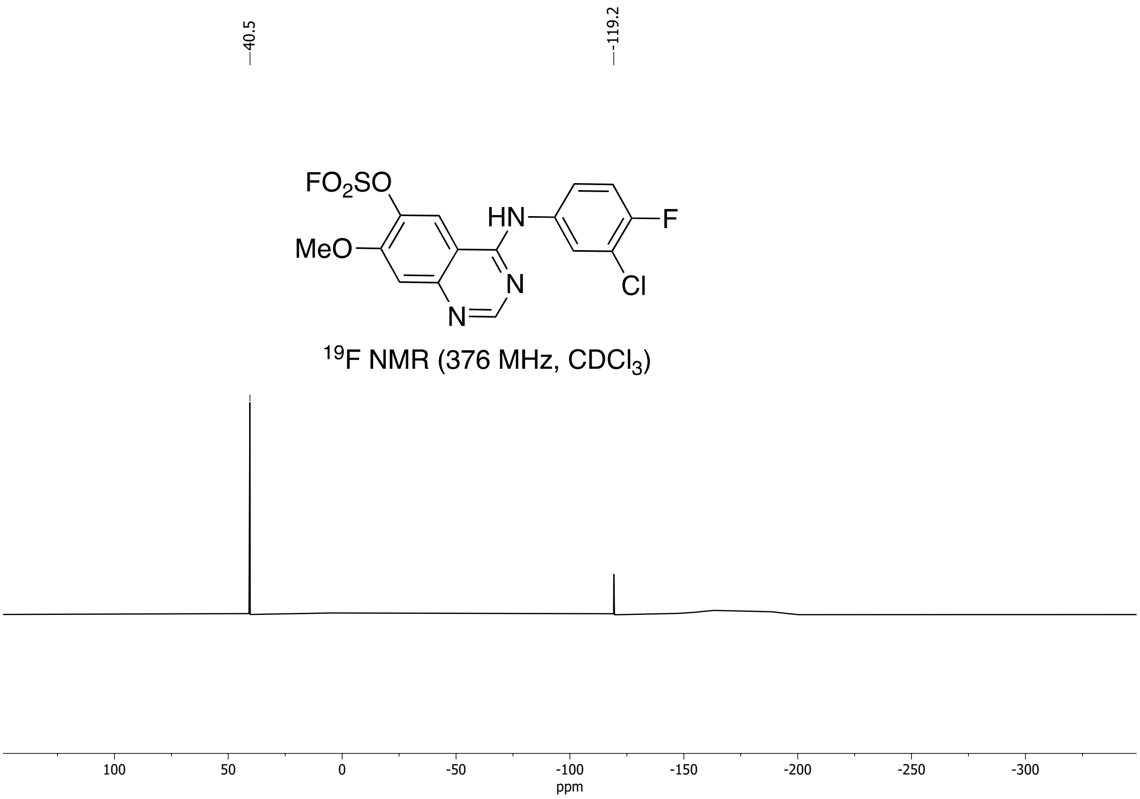
40.5
-119.2
FO2SO
HN
F
MeO
N
N
Cl
19F NMR (376 MHz, CDCl3)
100
50
0
-50
-100
-150
-200
-250
-300
ppm

8 References

1. Jensen, M. T. *et al.* Scalable carbon dioxide electroreduction coupled to carbonylation chemistry. *Nat Commun* **8**, 489 (2017).
2. Veryser, C., Demaerel, J., Bieliūnas, V., Gilles, P. & De Borggraeve, W. M. Ex Situ Generation of Sulfuryl Fluoride for the Synthesis of Aryl Fluorosulfates. *Org Lett* **19**, 5244–5247 (2017).
3. Lescot, C. *et al.* Efficient Fluoride-Catalyzed Conversion of CO2 to CO at Room Temperature. *J Am Chem Soc* **136**, 6142–6147 (2014).
4. Correa, A. & Martín, R. Palladium-catalyzed direct carboxylation of aryl bromides with carbon dioxide. *J Am Chem Soc* **131**, 15974–15975 (2009).
5. Fujihara, T., Nogi, K., Xu, T., Terao, J. & Tsuji, Y. Nickel-catalyzed carboxylation of aryl and vinyl chlorides employing carbon dioxide. *J Am Chem Soc* **134**, 9106–9109 (2012).
6. Ma, C. *et al.* Nickel-Catalyzed Carboxylation of Aryl and Heteroaryl Fluorosulfates Using Carbon Dioxide. *Org Lett* **21**, 2464–2467 (2019).
7. Shimomaki, K., Nakajima, T., Caner, J., Toriumi, N. & Iwasawa, N. Palladium-Catalyzed Visible-Light-Driven Carboxylation of Aryl and Alkenyl Triflates by Using Photoredox Catalysts. *Org Lett* **21**, 4486–4489 (2019).
8. Shimomaki, K., Murata, K., Martin, R. & Iwasawa, N. Visible-Light-Driven Carboxylation of Aryl Halides by the Combined Use of Palladium and Photoredox Catalysts. *J Am Chem Soc* **139**, 9467–9470 (2017).
9. Bhunia, S. K., Das, P., Nandi, S. & Jana, R. Carboxylation of Aryl Triflates with CO2 Merging Palladium and Visible-Light-Photoredox Catalysts. *Org Lett* **21**, 4632–4637 (2019).
10. Meng, Q. Y., Wang, S. & König, B. Carboxylation of Aromatic and Aliphatic Bromides and Triflates with CO2 by Dual Visible-Light–Nickel Catalysis. *Angewandte Chemie - International Edition* **56**, 13426–13430 (2017).
11. Sun, G. Q. *et al.* Nickel-catalyzed electrochemical carboxylation of unactivated aryl and alkyl halides with CO2. *Nat Commun* **12**, 2–11 (2021).
12. Wang, Y., Jiang, X. & Wang, B. Cobalt-catalyzed carboxylation of aryl and vinyl chlorides with CO_2. *Chemical Communications* **56**, 14416–14419 (2020).
13. Ang, N. W. J., Oliveira, J. C. A. & Ackermann, L. Electroreductive Cobalt-Catalyzed Carboxylation: Cross-Electrophile Electrocoupling with Atmospheric CO_2. *Angewandte Chemie International Edition* **59**, 12842–12847 (2020).
14. Amatore, C., Jutand, A., Khalil, F. & Nielsent, M. F. Carbon Dioxide as a C1 Building Block. Mechanism of Palladium-Catalyzed Carboxylation of Aromatic Halides. *J Am Chem Soc* **114**, 7076–7085 (1992).
15. Toriumi, N. *et al.* Mechanistic studies into visible light-driven carboxylation of aryl halides/triflates by the combined use of palladium and photoredox catalysts. *Bull Chem Soc Jpn* **94**, 1846–1853 (2021).
16. Weigend, F. Accurate Coulomb-fitting basis sets for H to Rn. *Physical Chemistry Chemical Physics* **8**, 1057 (2006).
17. Weigend, F. & Ahlrichs, R. Balanced basis sets of split valence, triple zeta valence and quadruple zeta valence quality for H to Rn: Design and assessment of accuracy. *Physical Chemistry Chemical Physics* **7**, 3297 (2005).

18. Grimme, S., Antony, J., Ehrlich, S. & Krieg, H. A consistent and accurate ab initio parametrization of density functional dispersion correction (DFT-D) for the 94 elements H-Pu. *J Chem Phys* **132**, 154104 (2010).
19. Lee, C., Yang, W. & Parr, R. G. Development of the Colle-Salvetti correlation-energy formula into a functional of the electron density. *Phys Rev B* **37**, 785–789 (1988).
20. Becke, A. D. Density-functional thermochemistry. III. The role of exact exchange. *J Chem Phys* **98**, 5648–5652 (1993).
21. Stephens, P. J., Devlin, F. J., Chabalowski, C. F. & Frisch, M. J. Ab Initio Calculation of Vibrational Absorption and Circular Dichroism Spectra Using Density Functional Force Fields. *J Phys Chem* **98**, 11623–11627 (1994).
22. Vosko, S. H., Wilk, L. & Nusair, M. Accurate spin-dependent electron liquid correlation energies for local spin density calculations: a critical analysis. *Can J Phys* **58**, 1200–1211 (1980).
23. Rudd, E. J., Finkelstein, M. & Ross, S. D. Anodic oxidations. VII. Reaction mechanism in the electrochemical oxidation of N,N-dimethylformamide in acetic acid and in methanol. *J Org Chem* **37**, 1763–1767 (1972).
