# Supplementary material for: Efficient palladium-catalyzed electrocarboxylation enables late-stage carbon isotope labelling
Source: Nat Commun. 2024 Mar 22;15:2592. doi: 10.1038/s41467-024-46820-9 (PMC10959938; doi:10.1038/s41467-024-46820-9)
Supplement: Supplementary file 2 — Description of Additional Supplementary Files [file 41467_2024_46820_MOESM2_ESM.pdf]

Description of Additional Supplementary Files

File Name: Supplementary Data 1
Description: This excel file contains the cartesian coordinates for all optimized structures (for the DFT study).
